# A Poorly Known High-Latitude Parasitoid Wasp Community: Unexpected Diversity and Dramatic Changes through Time

**DOI:** 10.1371/journal.pone.0023719

**Published:** 2011-08-29

**Authors:** Jose Fernandez-Triana, M. Alex Smith, Caroline Boudreault, Henri Goulet, Paul D. N. Hebert, Adam C. Smith, Rob Roughley

**Affiliations:** 1 Department of Integrative Biology and Biodiversity, Institute of Ontario, Guelph, Ontario, Canada; 2 Canadian National Collections of Insects, Agriculture and Agri-Food Canada, Ottawa, Ontario, Canada; 3 Environment Canada, Canadian Wildlife Service, National Wildlife Research Centre, Carleton University, Ottawa, Ontario, Canada; 4 Department of Entomology, University of Manitoba, Winnipeg, Manitoba, Canada; University of Western Ontario, Canada

## Abstract

Climate change will have profound and unanticipated effects on species distributions. The pace and nature of this change is largely unstudied, especially for the most diverse elements of terrestrial communities – the arthropods – here we have only limited knowledge concerning the taxonomy and the ecology of these groups. Because Arctic ecosystems have already experienced significant increases in temperature over the past half century, shifts in community structure may already be in progress. Here we utilise collections of a particularly hyperdiverse insect group – parasitoid wasps (Hymenoptera; Braconidae; Microgastrinae) – at Churchill, Manitoba, Canada in the early and mid-twentieth century to compare the composition of the contemporary community to that present 50–70 years ago. Morphological and DNA barcoding results revealed the presence of 79 species of microgastrine wasps in collections from Churchill, but we estimate that 20% of the local fauna awaits detection. Species composition and diversity between the two time periods differ significantly; species that were most common in historic collections were not found in contemporary collections and vice versa. Using barcodes we compared these collections to others from across North America; contemporary Churchill species are most affiliated with more south-western collections, while historic collections were more affiliated with eastern collections. The past five decades has clearly seen a dramatic change of species composition within the area studied coincident with rising temperature.

## Introduction

Changing global climate will affect species distributions, community composition and ecosystem function and these impacts are expected to be first evident in high elevation and high latitude communities [1]–[3]. Here, we expect extinction through attrition when a species cannot move higher or farther north [4]. However, there will be secondary impacts of climate changes, such as the disrupted phenological relationships between interacting species [5], including the altered synchronization between parasitoids and their host species [1]. Northerly species will be particularly prone to such alterations as the growing season is so short [6]. Accentuating this fear is our understanding of the ‘unknown unknowns’ of parasitoid biology: most species are undescribed – and therefore their interrelationships within an ecological community remain invisible to scientific enquiry [7].

Hymenopteran parasitoids are one of the most species-rich groups of animals, likely accounting for more than 20% of the world's insect diversity [8]. The braconid subfamily Microgastrinae is, in turn, one of the most diverse groups of parasitoid wasps – with over 2,000 described species and an estimated global diversity of 5,000–10,000 species [9]–[11]. Microgastrine wasps are parasitoids of Lepidoptera larvae and constitute an important group for biological control [11]–[13]. However, the taxonomic impediment [14] within this group is intense, and our current knowledge of this group's diversity is far from complete. In one recent example in northwestern Costa Rica (Area de Conservacion Guanacaste) a surprising diversity (313 provisional species within six genera) of microgastrine was revealed by integrating natural history, DNA barcoding, morphology and collections (http://janzen.sas.upenn.edu/) [15]. It is evident that further studies involving additional areas and regions will be necessary to better understand the diversity of parasitoid wasps.

We examined the diversity of the microgastrine wasps at Churchill, Manitoba, Canada – a sub-arctic locality with a legacy of scientific investigation – to document shifts in the diversity of this community of organisms. The Northern Insect Survey which was active in the 1940's and 50's made extensive collections in Churchill, which are now held in the Canadian National Collection of Insects, Nematodes and Arachnids (CNC) in Ottawa, Ontario, Canada. The CNC – like all contemporary natural history collections – is an invaluable tool in documenting changes in biodiversity [16], [17]. Comparing contemporary collections to this historical material makes it possible to test if there have been shifts in species composition linked to observed changes in climate [18], [19]. By incorporating DNA barcoding with traditional taxonomy (i.e. morphological studies), we found that the communities of parasitoids uncovered in the 20^th^ and 21^st^ centuries were markedly different and that the species currently found in Churchill have a greater affiliation to southern species than did the community in the historic collection. Furthermore, while the units of diversity independently identified using morphology or DNA barcodes were largely concordant (97.4%), our results strongly suggest that species composition has dramatically changed in this area over the 50–70 years between collecting efforts.

## Results

### Diversity of Microgastrinae in Churchill

Our study of both historical and contemporary collections revealed the presence of 79 species of microgastrine wasps belonging to 11 genera at Churchill (Table S1, Table S2). The list includes one new record for the Nearctic (*Microgaster deductor*) and 9 species recorded for the first time in Manitoba. Members of six genera (*Apanteles*, *Dolichogenidea*, *Cotesia*, *Glyptapanteles*, *Microgaster* and *Microplitis*) dominated the assemblage, comprising over 80% of the specimens and species collected.

**Figure 1:**
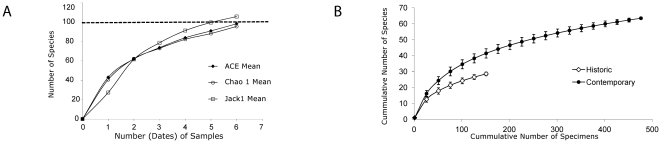
Churchill diversity patterns. **A**) Species richness estimators for Microgastrinae wasps at Churchill, Manitoba. The dashed line marks the estimated number of species (99.8) based in the average of the three estimators. **B**) Cumulative number of species for historical and contemporary collecting of Microgastrinae wasps at Churchill, Manitoba. Error bars show the standard deviation.

The six most abundant species (*Microplitis varicolor*, *Microplitis* jft04, *Glyptapanteles* jft05, *Microgaster deductor*, *Apanteles* jft02, *Pholetesor viminetorum*) represented 39.5% of all specimens collected (each with more than 30 specimens, Table S1). In contrast to these common taxa, 22 species (27.8%) were represented by just a single individual (singletons), while 13 (16.4%) were represented by two specimens (doubletons). The large proportion of singletons and doubletons in this collection makes it clear that many further species await detection at this locality. Several species richness estimators were calculated (Figure 1a) and estimates of total diversity ranged between 98.2–105.7 species, with an average of 99.8 species. Thus, about 20% of the microgastrine wasp species are still unknown and the species accumulation curve is far from asymptotic (Figure 1b) although it is starting to flatten after 500 specimens.

Diversity and evenness indices were high, with the lowest figures at the beginning and end of the season (Table S2). The peak of diversity was reached between July and the first half of August, while the beginning of the season (late May to early June) recorded the lowest number of specimens and species. While adults of some species flew from early June to late August, no species was collected throughout the entire season and 32 species (40.5%) were collected on just one sampling date (see Table S1). Where sample sizes were large enough to provide phenological information, males typically emerged about 2 weeks before females.

The recent collecting efforts (2005–2007) revealed a remarkable total of 64 species. Of these, only 15 taxa (23.4%) overlapped with species collected at Churchill before 1950. Fourteen of the 30 species (46.7%) collected before 1950 were absent from the contemporary collections, producing low similarity values (Sørensen Index = 0.32; Jaccard Similarity Coefficient = 0.19). Consider, for example, the most commonly collected species for each time period. Only one of the eight most abundant species from contemporary collections *(Microplitis varicolor)* was collected in the first half of the 20^th^ century and then, it was only represented by a single specimen. Conversely, only one of the five most common species in historic collections *(Pholetesor viminetorum)* was found in the contemporary collection. *Microgaster deductor*, the most abundant species in the historic collections and active throughout most of the season, was absent from contemporary sampling efforts.

The diversity and evenness indices and the rarefaction curves (Figure 1 and Table S2) provide additional evidence of the differences in species composition between the two time periods analyzed. It is clear from those data that regardless of sampling effort or number of specimens collected, the contemporary fauna is significantly more diverse. Altogether, our results strongly suggest that Churchill has been the site of a dramatic change in species composition over the last 50–70 years.

**Figure 2:**
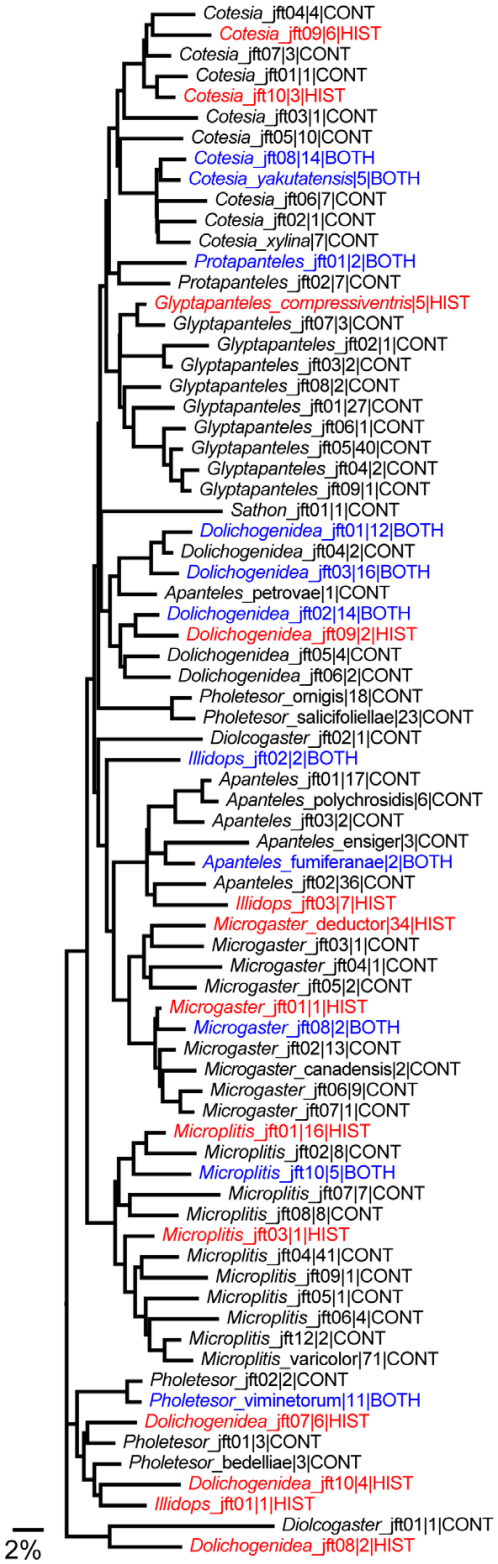
Neighbour-joining tree (K2P distance model) for Churchill Microgastrinae with single specimen per species. Tips are labelled: species name|sample size|collection period. Labels in red indicates specimens present only in historical collections (HIST), blue labels indicate a species present in both historical (BOTH) and contemporary collections, while labels in black indicate species only found in contemporary collections (CONT).

The barcode results revealed 25–30% more species than were recognized during a first sort to morphospecies by a taxonomist (JFT). Barcode sequence divergences within members of a species averaged 0.30% (Min = 0%, Max = 2.38%, SE = 0.006), while congeneric species had much higher divergences (average = 7.53% min = 1.33%, max = 20.24%, SE = 0.022). Nearly all species were clearly delimited from each other based on these values (Figure 2).

Sequence divergences within 28S were, as expected, lower than CO1 – intraspecific variation ranged from 0–1.83% (mean 0.14, SE = 0.01) while interspecific variation was higher and it did overlap with intraspecific (0–9.42%, mean 0.788, SE = 0.024). As such, it was not always possible to differentiate provisional species using a distance based measure of 28S. Character state analyses of 28S (as in [15]) have more utility when testing provisional species identified using barcode and morphology, and it was largely through a character based approach of the examination of apparently fixed nucleotide differences that these comparisons were made (see Table S3).

## Discussion

The diversity of the microgastrine wasps discovered at Churchill (79 species combined over each time period, 64 species in contemporary sampling) is extraordinary when compared to literature estimates of the Nearctic fauna north of 60° (30 species), Canada (140 species) or even the entire Nearctic region (300 species) [10], [13], [20], [21]. However, each of these estimates was considered preliminary [e.g. see [22] for updates estimates on Arctic and sub-Arctic North America species]. To contextualize our Churchill data we compared them with four other North American sites across a broad latitudinal gradient (39°–59°N) and from a wide range of habitats - apple orchards [23], tallgrass prairies [24] or forests [25], [26] (Figure S1). Although each study was made under different conditions and none (except ours) used a molecular approach, interesting patterns emerge. At all sites, the six most diverse genera accounted for 80–90% of the species. The most diverse genera at lower latitudes (*Cotesia* and *Apanteles*), were less dominant at northern localities where others increased in diversity (*Pholetesor* and *Microgaster*). Interestingly, *Glyptapanteles*, a genus typically considered to peak in diversity in the tropics, also increased its diversity in the north.

At first consideration, our results suggest that, despite its northern location, Churchill possesses the most diverse microgastrine fauna in North America. However, Churchill's diversity is a clear result of integrating morphology and DNA barcoding. Churchill's “hyperdiverse” status actually reflects the manner in which barcode data reveal species that would otherwise be overlooked. We are confident that integrating a barcoding component into studies at the aforementioned localities would also result in dramatic increases of diversity.

### Barcoding Analysis

We obtained sequence information from the barcode region for 98.2% (480/489) of the recently collected specimens with 87.1% of these records >500 bp (Figure 2). For specimens collected between 1930 and 1950, the barcode sequences were much shorter (usually around 150 bp) but we obtained sequences from 72.5% of the specimens (124/171). Altogether, we obtained barcodes or shorter sequences for 77 (97.5%) of the species and 604 (91.5%) of the specimens (Figure S2). Only two species (*Apanteles morrisi*, *Protapanteles* jft03), each represented by a single specimen, lacked a sequence record.

The 25–50% increase in diversity following barcoding was particularly significant in four genera (*Dolichogenidea*, *Glyptapanteles*, *Microplitis* and *Microgaster*). However, when specimens were re-examined in light of the molecular data, it was possible to find consistent, though subtle, morphological differences that supported the new species revealed through barcode data (Table S3, Table S4, Table S5). This parallels other experiences in Costa Rica [15] when barcoding suggested cryptic diversity within groups initially thought to be morphologically homogeneous.

For example, *Microplitis* jft12 and *Microplitis varicolor* possessed 1.97% CO1 divergence but we did not find morphological differences or nuclear 28S haplotypes that would reliably separate them. *M. varicolor* is a widespread species in North America [10] with considerable morphological variation, suggesting that it actually is a complex of cryptic species. Resolving *M. varicolor* taxonomy will require the study of more specimens from additional localities; a task beyond the scope of this paper.

In another case, the genetic distance between two species of *Apanteles* (*A. polychrosidis* (six specimens) and *Apanteles* jft01 (16 specimens)) was only 1.04%. Yet, each species had striking and consistent morphological differences, despite falling within the range of variation often recorded within single species.

Barcodes alone reliably separated 97.4% of the species recognized in this study. This result illustrates the role that DNA barcodes can play in providing a rapid and inexpensive way of first separating species when carrying out biodiversity inventories for complex, highly diverse, and taxonomically difficult groups. Similar results were reported in studies of tachinid flies [27], [28] and microgastrine wasps [15] in Costa Rica, as well as parasitoid wasps in temperate Canada [7] and Europe [29] It is unlikely that morphological investigations would separate as many species as barcoding reveals and, even if possible, it would require significant effort by an experienced researcher, a scarce resource. When barcoding flags specimens that may or may not constitute different species, it provides valuable insight to taxonomists. By concentrating on re-examining those specimens, the accuracy of the final work is improved.

Our results have one important element of concordance with earlier investigations on the microgastrine fauna of Costa Rica. The coupling of morphological taxonomy with DNA barcoding has revealed a far greater diversity of microgastrines than expected [15], [27], [28], [30]. Our study makes clear the critical value that historic regional collections in public institutions [31] have in providing insights into the impacts of climate change.

### Species Composition and Climate Change

Our results provide a novel perspective on the shifts in species composition against a backdrop of a changing climate. Our data indicate a radical shift in the composition of this subfamily of parasitoid wasps, likely reflecting both a direct response of the parasitoids and an indirect response to altered distributions of their hosts.

The striking differences in species composition between the historical and contemporary samples are not explained by differences in sampling methods or effort. If contemporary sampling effort was much greater than the historical, the species that were common historically should have been represented in the contemporary samples if they were still common. Indeed, the contemporary sampling used a more diverse suite of trapping methods, including the sweep collection methods that were used historically and more efficient passive-trapping methodologies (Malaise and yellow pan traps). This greater sampling effort produced a larger amount of material available for study (489 vs 171 individuals) that should have included representatives of the historically most common species, unless the species composition had dramatically changed.

By treating our sites and species as “random effects” (*sensu* Shaffer et al., 1998 - [32]), these broad patterns in species diversity can be compared across more than half a century, even though different sampling protocols were used [32]. Methods of collection [33] and natural fluctuations in abundance [34] affect ones capacity to compare any species in space and time. After thoroughly reviewing the field notes and collection records of the historic collections, we were unable to design a contemporary sampling protocol that would replicate the historic collections in sufficient detail to permit the use of highly-rigorous occupancy models [16] and thus our comparisons of space and species are not fixed [32]. The sampling locations in both the contemporary and historical surveys were designed to be a reasonably representative sample of the Churchill region and its microgastrine community. As such, they are perfectly suited to a random strategy focusing on different sites from the same broader area that are presumed to be equivalent [32]. In an analogous manner, “species” are here, also treated as random sampling units of the broader community – a strategy advisable when diversity is great and the goal is broad changes in diversity, rather than specific inferences regarding any specific taxa [32]. And indeed, our results show broad changes in diversity and community composition.

Any within-year fluctuations are likely to be reflected in our data. Consider that in 2007, we collected 33 species on multiple occasions over 51 collecting days (the insect ‘season’ may only be 3 months at Churchill). While 55% of these multiple occasions were within two weeks of each other (ephemeral), the remaining 45% occurred 20 and 50 days apart (long-lasting). Therefore, two months of collecting in Churchill captured both ephemeral and long-lasting parasitoid species and while parasitoid populations do fluctuate [34], we feel that it is unlikely that the differences we have uncovered are due to all species fluctuating in synchrony. We conclude that the absence of the most common species from contemporary collections (using both active and passive sampling technologies), and vice versa, is meaningful evidence [32] of a dramatic change in parasitoid species composition.

**Figure 3:**
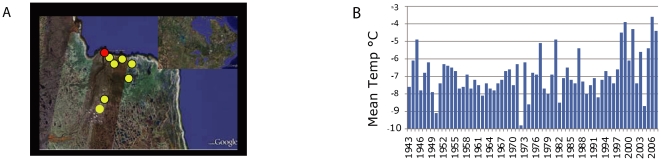
Churchill collection localities and temperature data. **A**) Map of Churchill (red circle) Manitoba and its surroundings. Yellow circles mark contemporary and historical collecting sites; orange circles mark contemporary only collecting sites. **B**) Average mean temperature at Churchill airport (data: Environment Canada, National Climate Data and Information Archive [58]). The decade between 1997 and 2007 saw 6/10 of the hottest years, and all of the top five.

We note that in other northern regions, where much longer-term data exists, community compositional changes evident in the past several decades were found to be unique at that site when contextualised over 200,000 years (See Axford et al [35]). Using paleoecological data from lake sediments their results supported the conclusion that the north is entering a period of extraordinary biotic change. In the portions of the north where our study occurred, air temperatures over western Hudson Bay have increased by 2–3°C over the past 50 years [36], [37]. When our results, gathered between the late 1930's and as recently as 2007, are contextualized by demonstrated increases in temperature (Figure 3b) they reinforce recent findings [35], [38] of striking changes in biodiversity within the most recent past. The dramatic changes noted in aquatic environments and communities will be paralleled by dramatic biological shifts in terrestrial communities.

Varied climate change models predict that species ranges should experience northward latitudinal drift [18], [19]. As such, collections from sites several hundred kilometers to the north of Churchill should yield those species that were present at this locality 50 years ago, though not today. Additionally, it would be interesting to test whether those species found in Churchill today are also found to be continuously distributed in southern Manitoba. Species adapted to yet colder conditions may already be facing increasing range restriction and extinction through habitat attrition as shifts in their preferred climatic envelope outstrip the dispersal capacity of the organism. Danks [39] proposed that inter-specific ratios of host∶parasitoid in the Arctic could be used to demonstrate changes to ecosystem structure and responses to environmental change and recently, it was predicted that the Churchill area would experience a net increase in butterfly species richness [40]. However, the predicted impacts of climate change on the space and time relations between Lepidoptera and their host plant as well as between the Lepidoptera and their parasitoids are difficult and remain unanswered. Are parasitoids tracking the movements of their associated host species, or is there a broader than expected host range – both questions remain to be answered.

**Figure 4:**
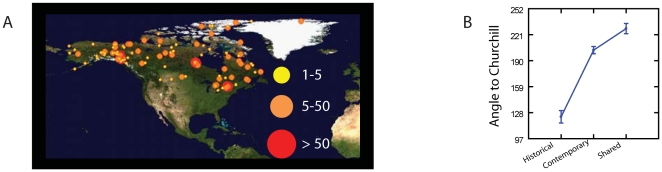
Broad collection localities and barcode affiliations. **A**) Map of collection localities where barcode comparisons suggested faunal affiliations to Churchill (contemporary and historical). **B**) Mean and SE of directions of that faunal affiliation to Churchill.

For instance, comparing the Churchill species to Microgastrinae collected across North America (and housed at the CNC) (Figure 4) we found that 1/3^rd^ of both historic and contemporary Churchill specimens have a very restricted geographic affiliation (affiliation measured as containing barcode similarities of less than 2% K2P genetic divergence and that were are not shared with any other geographic regions currently represented in CNC collections that have been barcoded). However, while each temporal category of Churchill microgastrine community has affiliations to other regions (Table S1), the historic Churchill collections have an affiliation to a comparatively small number of CNC specimens from eastern North America, while contemporary Churchill specimens have strong and wide affiliations to the west and the south (Figure 4, Figure S3). Thus, while Churchill's microgastine community appears unique, our findings re-enforce the view that the biota in Churchill has changed from what was there historically. What is there now has much wider, and more southerly, regional affiliations.

### Conclusions

The revelation of these trends, in a group of insects that lies at the leading edge of the “taxonomic impediment”, was critically dependent on the existence of a curated collection, the accuracy of morphological investigations, and the amenability of those specimens to DNA barcode analysis. No one of these elements alone would have been sufficient to capture this pattern. Species declines and range changes are critically dependent on the data harboured in museum and herbarium collections – maintaining these collections with a view towards DNA sampling in the future is critically important. Our results revealed that the microgastrine fauna at Churchill has undergone a dramatic change in species composition compared to collections made in the early 20^th^ century. It is unlikely that natural fluctuations in abundance and differences in methods of collection account for this variation. Instead, it is likely that the demonstrated patterns of climate change occurring during the same time period have resulted in the dramatic compositional alterations in this terrestrial insect species assemblage.

## Methods

Churchill is located in the Coastal Hudson Bay Lowland Ecoregion, which belongs to the Hudson Plains Ecozone [41]. This Ecoregion has a sub-arctic climate and is part of the broad area of tundra and boreal forest transition. The vegetation is characterized by open stands of stunted black spruce and tamarack with lesser quantities of white spruce to a shrub layer of dwarf birch, willow or ericaceous shrubs, and a ground cover of cottongrass or lichen and moss. Poorly drained sites support tussock vegetation of sedge, cottongrass, and sphagnum moss [41]. Climate measurements (mean average temperature) were generated by Environment Canada continuously between 1943 and 2007 (Figure 3b).

Collection sites were scattered over 100 km^2^, extending 30 km east of the Churchill town, 15 km to the south, limited to the west by the Churchill River and to the north by Hudson Bay (Figure 3a). Here, specimens were collected using standard entomological methods, including sweep nets for the historical collections. For the modern collections pitfall traps, yellow pan traps, Malaise traps and sweep nets were used (for a thorough review of standard techniques see [42]).

The locations of the modern collections comprised all of the ones sampled during the first half of the 20^th^ century, with some additional localities added especially to the west of town and extending up to Wapusk National Park. Exact trap locations are detailed in the SI Table S5.

The majority of the contemporary collections were made by undergraduate students participating in an Arctic Ecology field course. Collection microhabitats mirrored that of the historical collections (e.g. patches of flowering plants, river banks, willows, marshes and road edges). Photographs and collection records [43] from the 1950's suggest that historical active collections were completed in as wide a variety of habitats as the modern collections. Afterwards, we consulted the field notes of the entomologists who carried out the historical sampling (those notebooks are kept in the Hymenoptera Unit of the CNC), as well as literature which detailed the way sampling sites in the Arctic (though not Churchill) to ensure that the contemporary collection localities mirrored those made by the earlier researchers (e.g. [43], [44]). While our analysis takes advantage of each of these period-specific collecting events, the synthesis presented here was not part of the experimental design by the contemporary, nor the historical collectors. As such, there are some details that we would prefer to include, but we are unable due to the *a posteriori* nature of our analysis (e.g. comparable GPS precision of collection localities for historical collections, and per/specimen details regarding the exact collection method for the contemporary specimens).

We studied historical holdings in the Canadian National Collection (CNC), comprising 171 specimens, mostly from expeditions between 1940 and 1950 as part of the Northern Insect Survey effort, but also including a few specimens collected as early as 1937. We examined 489 specimens recently collected (mostly in 2007, with some specimens from 2005 and 2006) which were deposited in the CNC, the J. B. Wallis Museum, and the Biodiversity Institute of Ontario. In total, 660 specimens were examined. Until now, little information was available on the diversity of Microgastrinae at Churchill as only three species were recorded [43].

We computed non-parametric species richness estimators using the software EstimateS (version 8.0.0, October 26, 2006) and calculated Shannon-Wiener (H′) and the Evenness (J) indices following [45]. Because the data from the historical and recent collections had different sample sizes, we computed rarefactions curves using RAREFACT.FOR [46] to compare the cumulative number of species, as well as to estimate and compare its diversity. The Shannon-Wiener index (H′) and the Evenness (J) indices were calculated as described by Magurran [45]. The Shannon-Wiener index is a measure of the likelihood that the next individual will be the same species as the previous sample. It is based on the number of species and captures. The Evenness index is derived from the Shannon-Wiener index. It is the Shannon-Wiener index divided by the log of the total number of species. It is a measure of similarity for the abundance of different species. Rarefaction calculations and the Chao-2 index were also calculated to estimate the diversity of Microgastrinae at the two sites (times) [47]–[49]. Rarefaction calculations compare the cumulative distribution of species at two sites with different sample sizes. It is based on data from a large sample to estimate the number of species to be found in a small sample [50]–[52].

DNA extracts were prepared from single legs using a glass fibre protocol [53]. Extracts were re-suspended in 30 µl of dH2O, and a 658-bp region near the 5′ terminus of the COI gene was amplified using primers (LepF1–LepR1) following standard protocols. Composite sequences were generated using internal primers when initial amplification was not successful. Sequence divergences were calculated using the K2P distance model and a NJ tree of K2P distance was created to provide a graphic representation of the among-species divergences. Full details of methodology are as in [15], [28]. All sequence data are available on BOLD (www.barcodinglife.org). New sequences from this analysis are in the public projects: Temporal Biological Shifts in Microgastrinae Wasps (CNCAS, ASCON, CNCBM), while the 160 already published sequences can be retrieved from BOLD by searching sample accession numbers in SI File 4. GenBank accessions for all sequences are in SI File 4.

Each individual was assigned to a barcode cluster using barcode divergences between 1–3% using the Barcode of Life Datasystem (BOLD [54]). Those results were then compared with the species delineated by a comprehensive morphological study (entailing more than 70 characters (SI Tables 2 & 3)), employing approaches from previous studies on Microgastrinae [9], [15], [55]–[57]. Species that could only be identified to genus level were assigned numbers following the acronym of the identifier (e.g. *Apanteles* jft01). Historic and contemporary barcoded specimens were compared to other, broadly distributed microgastrine collections from the CNC (Figure 4 and Figure S3).

When barcode analyses suggested morphologically cryptic mitochondrial diversity we amplified portions of the rRNA gene region for a portion of the large subunit (LSU or 28S – the variable D2 region) in addition to the CO1 barcode region. rDNA sequences facilitated our interpretation of morphologically cryptic and geographically sympatric deep mtDNA splits (correlated splitting within the independent rDNA sequence supports the hypothesis of morphologically cryptic species, while the lack of a split suggests mtDNA variation within a species). The 28S amplicon forward primer corresponds to positions 3549–3568 in the *Drosophila melanogaster* reference sequence (GenBank M21017). GenBank accessions for all sequences are in Table S5.

## Supporting Information

Figure S1 Proportion of species within genera of Microgastrinae from five different study sites within the Nearctic: 1) Arkansas forests, 34°N and 65 species [18]; 2) Midwestern US tall grass prairies, 39°N and 55 species [17]; 3) Forests of Yellowstone National Park, 44°N and 35 species[19]; 4) Quebec apple orchards, 45°N and 36 species [16]; 5) Boreal forest/tundra in Churchill, 59°N and 79 species (present work).
(PDF)

Figure S2 Length of CO1 sequences (bp) obtained from specimens (contemporary and historical collections) of Microgastrinae wasps at Churchill, Manitoba.
(PDF)

Figure S3 Neighbor-joining tree (K2P) constructed with Churchill specimens (N = 1037) based on all codon positions and sequence lengths. Tip labels include: Taxonomy|BOLD Process ID|Collection Date|Country and Province.
(PDF)

Table S1 Species of Microgastrinae from Churchill, Manitoba, Canada. Data comprise the first half of the 20th century (1930–1950's), referred as “Hist” in the heading of the second column; and recent collecting (2005–2007), referred as “Cont” in the heading of the third column. Species presence (+) or absence (−) is shown for Old and New data. Collecting dates were grouped in first and second half of every month sampled, indicated in the table headings with Arabic numbers (1 or 2) followed by the corresponding month (June = Jun, July = Jul, August = Aug). New records for Manitoba (*) or the Nearctic (**) are marked in the table. N/B marks the species with no barcodes available from this study.
(PDF)

Table S2 Shannon-Wiener (H′) and Evenness (J) diversity indices of Microgastrinae from Churchill, Manitoba, Canada. Collecting dates were grouped in first and second half of every month sampled, indicated in the table headings with Arabic numbers (1 or 2) followed by the corresponding month (June = Jun, July = Jul, August = Aug).The last column refers to all dates and specimens combined.
(PDF)

Table S3 Taxonomic commentary and context for selected species of Microgastrinae from Churchill, Manitoba, Canada.
(PDF)

Table S4 Morphological characters used to study the species of Microgastrine from Churchill, Manitoba, Canada; and its definition. Based on Mason, 1981 [1], Whitfield et al., 2002 [2], Smith et al., 2008 [3], Fernandez-Triana and Goulet, 2009 [4], Valerio and Whitfield, 2009 [5], and unpublished data from JFT.
(PDF)

Table S5 All collection and accession information associated with specimens analysed here.
(PDF)
