# Supplementary material for: A Poorly Known High-Latitude Parasitoid Wasp Community: Unexpected Diversity and Dramatic Changes through Time
Source: PLoS One. 2011 Aug 29;6(8):e23719. doi: 10.1371/journal.pone.0023719 (PMC3163582; doi:10.1371/journal.pone.0023719)
Supplement: Figure S1 — Proportion of species within genera of Microgastrinae from five different study sites within the Nearctic: 1) Arkansas forests, 34°N and 65 species [18]; 2) Midwestern US tall grass prairies, 39°N and 55 species [17]; 3) Forests of Yellowstone National Park, 44°N and 35 species[19]; 4) Quebec apple orchards, 45°N and 36 species [16]; 5) Boreal forest/tundra in Churchill, 59°N and 79 species (present work). (PDF) [file pone.0023719.s001.pdf]

Figure S1: Proportion of species within genera of Microgastrinae from five different study sites within the Nearctic: 1) Arkansas forests, 34° N and 65 species [18]; 2) Midwestern US tall grass prairies, 39° N and 55 species [17]; 3) Forests of Yellowstone National Park, 44° N and 35 species[19]; 4) Quebec apple orchards, 45° N and 36 species [16]; 5) Boreal forest/tundra in Churchill, 59° N and 79 species (present work).

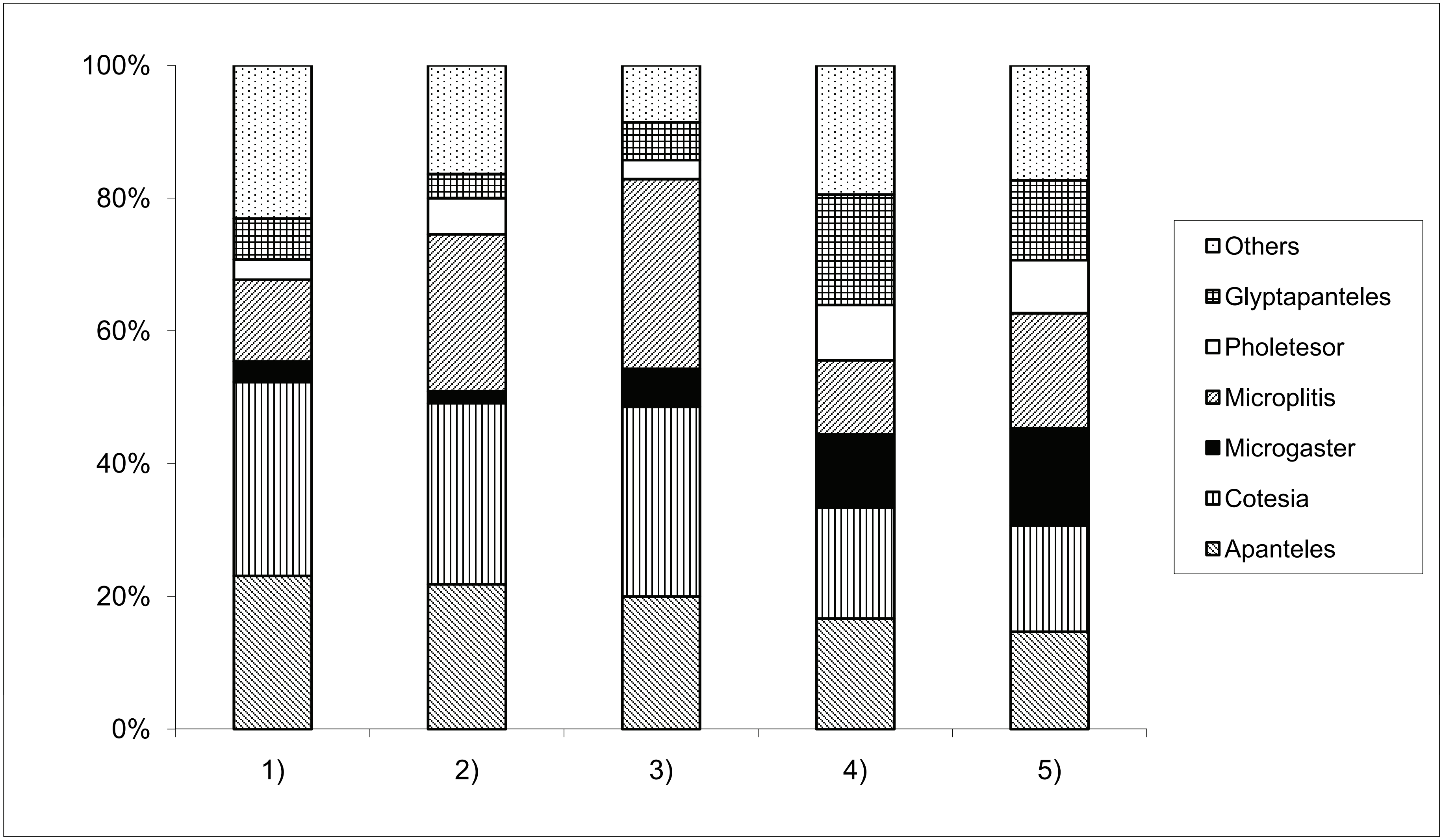

100%
80%
60%
40%
20%
0%
1)
2)
3)
4)
5)
Others
Glyptapanteles
Pholetesor
Microplitis
Microgaster
Cotesia
Apanteles
