# Supplementary material for: A Poorly Known High-Latitude Parasitoid Wasp Community: Unexpected Diversity and Dramatic Changes through Time
Source: PLoS One. 2011 Aug 29;6(8):e23719. doi: 10.1371/journal.pone.0023719 (PMC3163582; doi:10.1371/journal.pone.0023719)
Supplement: Figure S2 — Length of CO1 sequences (bp) obtained from specimens (contemporary and historical collections) of Microgastrinae wasps at Churchill, Manitoba. (PDF) [file pone.0023719.s002.pdf]

Figure S2: Length of CO1 sequences (bp) obtained from specimens (contemporary and historical collections) of Microgastrinae wasps at Churchill, Manitoba.

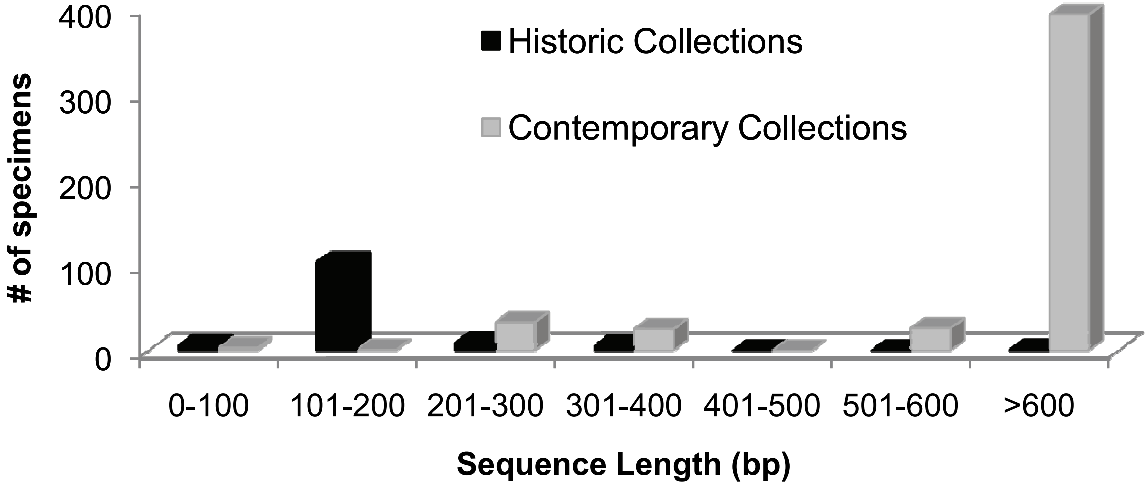

400
300
200
100
0
# of specimens
Historic Collections
Contemporary Collections
0-100
101-200
201-300
301-400
401-500
501-600
>600
Sequence Length (bp)
