# Supplementary material for: A Poorly Known High-Latitude Parasitoid Wasp Community: Unexpected Diversity and Dramatic Changes through Time
Source: PLoS One. 2011 Aug 29;6(8):e23719. doi: 10.1371/journal.pone.0023719 (PMC3163582; doi:10.1371/journal.pone.0023719)
Supplement: Figure S3 — Neighbor-joining tree (K2P) constructed with Churchill specimens (N = 1037) based on all codon positions and sequence lengths. Tip labels include: Taxonomy|BOLD Process ID|Collection Date|Country and Province. (PDF) [file pone.0023719.s003.pdf]

# BOLD TaxonID Tree

| | | |
|---|---|---|
| Project | : | MERGED: {ASCON,CNCBM,CNCAS} |
| Subprojects | : | TEMPORAL BIOLOGICAL SHIFTS OF MICROGASTRINE WASPS AT CHURCHILL-Contemporary |
| | | TEMPORAL BIOLOGICAL SHIFTS OF MICROGASTRINE WASPS AT CHURCHILL- BIOLOGICALLY |
| | | TEMPORAL BIOLOGICAL SHIFTS OF MICROGASTRINE WASPS - Historical[CNCAS] |
| Date | : | 10-August-2011 |
| Data Type | : | Nucleotide |
| Distance Model | : | Kimura 2 Parameter |
| Marker | : | COI-5P |
| Codon Positions | : | 1st, 2nd, 3rd |
| Labels | : | Country & Province, ProcessID, |
| Filters | : | Length > 50 |

| | | |
|---|---|---|
| Sequence Count | : | 1037 |
| Species count | : | 100 |
| Genus count | : | 11 |
| Family count | : | 1 |
| Unidentified | : | 26 |

10 %

Diolcogaster jft01|ASWA1033-08|17-Jul-2007|Canada.Manitoba
Diolcogaster|CNCHZ130-09|20-Jul-1959|United States.Alaska
Apanteles jft11|CNCHZ077-09|13-Jul-1960|Canada.Northwest Territories
Apanteles jft11|CNCHZ078-09|13-Jul-1955|Canada.Northwest Territories
Microplitis varicolor|CNCHZ439-09|16-May-2007|Canada.Ontario
Microplitis varicolor|CNCHZ440-09|16-May-2007|Canada.Ontario
Microplitis varicolor|CNCHX219-09||Canada.Ontario
Microplitis varicolor|CNCHX224-09||Canada.Ontario
Microplitis varicolor|CNCHX197-09||Canada.Ontario
Microplitis varicolor|CNCHX203-09||Canada.Ontario
Microplitis varicolor|CNCHX195-09||Canada.Ontario
Microplitis varicolor|CNCHZ370-09|30-Jul-2007|Canada.Ontario
Microplitis varicolor|CNCHZ425-09|30-Jul-2007|Canada.Ontario
Microplitis varicolor|CNCHZ433-09|30-Jul-2007|Canada.Ontario
Microplitis varicolor|CNCHZ434-09|30-Jul-2007|Canada.Ontario
Microplitis varicolor|CNCHZ430-09|30-Jul-2007|Canada.Ontario
Microplitis varicolor|CNCHZ431-09|30-Jul-2007|Canada.Ontario
Microplitis varicolor|CNCHZ427-09|30-Jul-2007|Canada.Ontario
Microplitis varicolor|CNCHZ429-09|30-Jul-2007|Canada.Ontario
Microplitis varicolor|CNCHZ423-09|13-Jul-2007|Canada.Ontario
Microplitis varicolor|CNCHZ426-09|30-Jul-2007|Canada.Ontario
Microplitis varicolor|CNCHZ412-09|30-May-2007|Canada.Ontario
Microplitis varicolor|CNCHZ413-09|30-May-2007|Canada.Ontario
Microplitis varicolor|CNCHZ401-09|30-May-2007|Canada.Ontario
Microplitis varicolor|CNCHZ406-09|30-May-2007|Canada.Ontario
Microplitis varicolor|CNCHZ396-09|19-Oct-2007|Canada.Ontario
Microplitis varicolor|CNCHZ399-09|19-Oct-2007|Canada.Ontario
Microplitis varicolor|CNCHZ387-09|19-Sep-2007|Canada.Ontario
Microplitis varicolor|CNCHZ391-09|19-Sep-2007|Canada.Ontario
Microplitis varicolor|CNCHZ384-09|19-Sep-2007|Canada.Ontario
Microplitis varicolor|CNCHZ386-09|19-Sep-2007|Canada.Ontario
Microplitis varicolor|CNCHZ381-09|16-Jun-2007|Canada.Ontario
Microplitis varicolor|CNCHZ383-09|19-Sep-2007|Canada.Ontario
Microplitis varicolor|CNCHZ379-09|30-Jul-2007|Canada.Ontario
Microplitis varicolor|CNCHZ380-09|30-Jul-2007|Canada.Ontario
Microplitis varicolor|CNCHZ377-09|30-Jul-2007|Canada.Ontario
Microplitis varicolor|CNCHZ378-09|30-Jul-2007|Canada.Ontario
Microplitis varicolor|CNCHZ375-09|30-Jul-2007|Canada.Ontario
Microplitis varicolor|CNCHZ376-09|30-Jul-2007|Canada.Ontario
Microplitis varicolor|CNCHZ372-09|30-Jul-2007|Canada.Ontario
Microplitis varicolor|CNCHZ373-09|30-Jul-2007|Canada.Ontario
Microplitis varicolor|CNCHZ366-09|10-Aug-2007|Canada.Ontario
Microplitis varicolor|CNCHZ368-09|10-Aug-2007|Canada.Ontario
Microplitis varicolor|CNCHZ363-09|10-Aug-2007|Canada.Ontario
Microplitis varicolor|CNCHZ365-09|10-Aug-2007|Canada.Ontario
Microplitis varicolor|CNCHZ361-09|10-Aug-2007|Canada.Ontario
Microplitis varicolor|CNCHZ362-09|10-Aug-2007|Canada.Ontario
Microplitis varicolor|CNCHZ285-09|10-Aug-2007|Canada.Ontario
Microplitis varicolor|CNCHZ360-09|10-Aug-2007|Canada.Ontario
Microplitis varicolor|CNCHZ283-09|10-Aug-2007|Canada.Ontario
Microplitis varicolor|CNCHZ284-09|10-Aug-2007|Canada.Ontario
Microplitis varicolor|CNCHX193-09||Canada.Ontario
Microplitis varicolor|CNCHX209-09||Canada.Ontario
Microplitis varicolor|ASWAY008-08|28-Jun-2007|Canada.Manitoba
Microplitis varicolor|ASWA1070-08|07-Jul-2007|Canada.Manitoba
Microplitis varicolor|CNCHY218-07|19-Jul-2006|Canada.Yukon Territory
Microplitis varicolor|CNCHY224-07|19-Jul-2006|Canada.Yukon Territory
Microplitis varicolor|CNCHX225-09||Canada.Ontario
Microplitis varicolor|CNCHX194-09||Canada.Ontario
Microplitis varicolor|CNCHX222-09||Canada.Ontario
Microplitis varicolor|CNCHZ281-09|10-Aug-2007|Canada.Ontario
Microplitis varicolor|CNCHZ367-09|10-Aug-2007|Canada.Ontario
Microplitis varicolor|CNCHZ382-09|19-Sep-2007|Canada.Ontario
Microplitis varicolor|CNCHZ398-09|19-Oct-2007|Canada.Ontario
Microplitis varicolor|CNCHZ408-09|30-May-2007|Canada.Ontario
Microplitis varicolor|CNCHZ410-09|30-May-2007|Canada.Ontario
Microplitis varicolor|CNCHZ392-09|19-Sep-2007|Canada.Ontario
Microplitis varicolor|CNCHZ113-09|13-Jul-1962|United States.Alaska
Microplitis varicolor|CNCHY217-07|15-Jul-2006|Canada.Yukon Territory
Microplitis varicolor|CNCHY221-07|19-Jul-2006|Canada.Yukon Territory
Microplitis varicolor|ASWAV959-08|16-Jul-2007|Canada.Manitoba
Microplitis varicolor|CNCHY259-07|15-Jul-2006|Canada.Yukon Territory
Microplitis varicolor|ASWAV970-08|16-Jul-2007|Canada.Manitoba
Microplitis varicolor|ASWAX980-08|23-Jul-2007|Canada.Manitoba
Microplitis varicolor|ASWAX978-08|23-Jul-2007|Canada.Manitoba
Microplitis varicolor|CNCHY255-07|19-Jul-2006|Canada.Yukon Territory
Microplitis varicolor|ASWA1036-08||Canada.Manitoba
Microplitis varicolor|ASWA1078-08||Canada.Manitoba
Microplitis varicolor|ASWAT764-08|07-Jul-2007|Canada.Manitoba
Microplitis varicolor|ASWA945-08|03-Jul-2007|Canada.Manitoba
Microplitis varicolor|CNCHY262-07|15-Jul-2006|Canada.Yukon Territory
Microplitis varicolor|ASWAV969-08|16-Jul-2007|Canada.Manitoba
Microplitis varicolor|CNCHY264-07|13-Jul-2006|Canada.Yukon Territory
Microplitis varicolor|CNCHZ102-09|12-Jul-1954|Canada.Quebec
Microplitis varicolor|CNCHZ158-09|10-Jul-1951|United States.Alaska
Microplitis varicolor|ASWAY610-08|05-Aug-1952|Canada.Manitoba
Microplitis varicolor|ASWAV908-08|11-Aug-2007|Canada.Manitoba
Microplitis varicolor|ASWAV902-08|11-Aug-2007|Canada.Manitoba
Microplitis varicolor|ASWAV901-08|11-Aug-2007|Canada.Manitoba
Microplitis varicolor|ASWAV887-08|11-Aug-2007|Canada.Manitoba
Microplitis varicolor|ASWAV900-08|11-Aug-2007|Canada.Manitoba
Microplitis varicolor|ASWAV882-08|11-Aug-2007|Canada.Manitoba
Microplitis varicolor|ASWAY004-08|07-Aug-2007|Canada.Manitoba
Microplitis varicolor|ASWAV899-08|11-Aug-2007|Canada.Manitoba

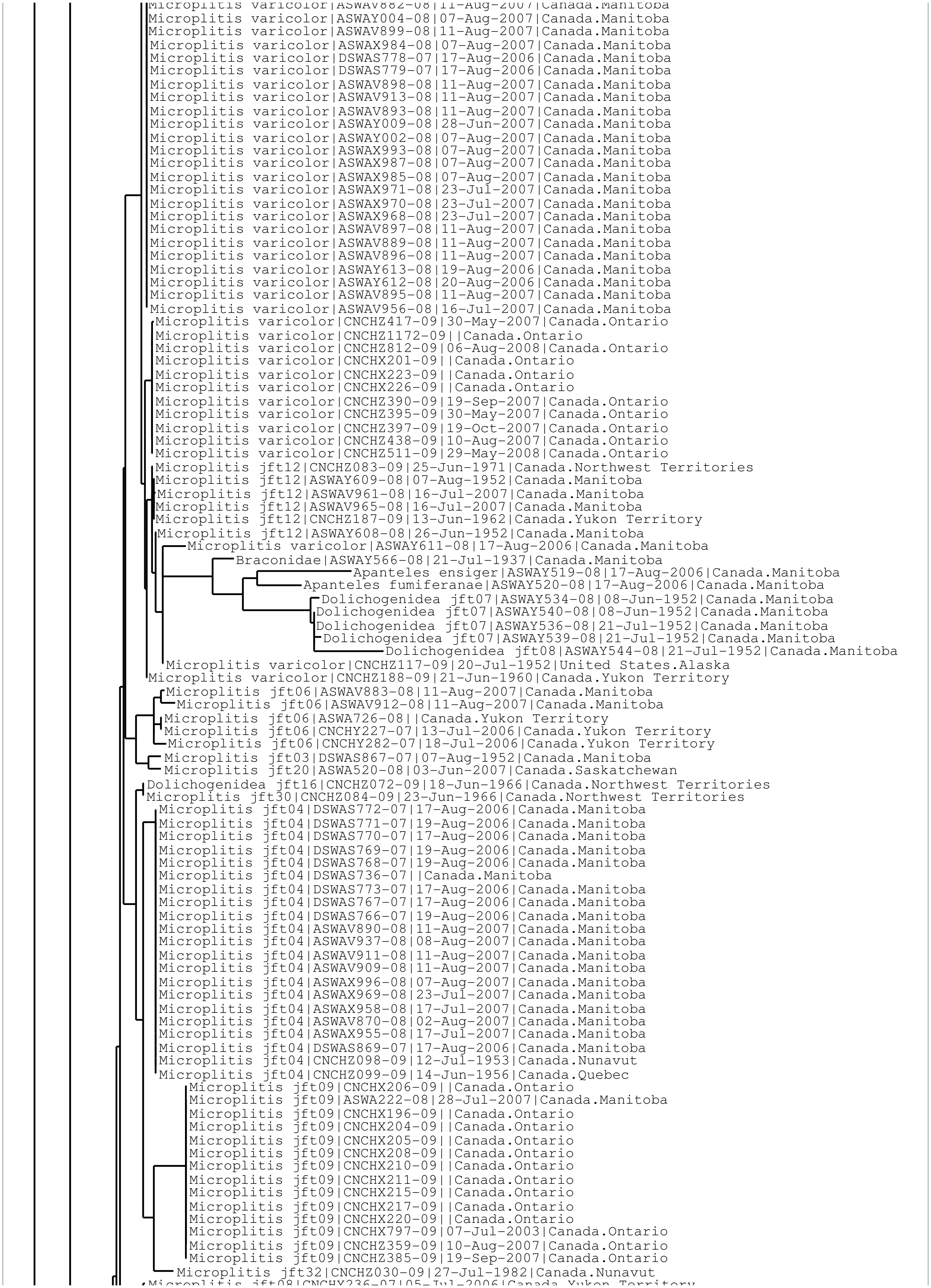
Microplitis varicolor|ASWAV882-08|11-Aug-2007|Canada.Manitoba
Microplitis varicolor|ASWAY004-08|07-Aug-2007|Canada.Manitoba
Microplitis varicolor|ASWAV899-08|11-Aug-2007|Canada.Manitoba
Microplitis varicolor|ASWAX984-08|07-Aug-2007|Canada.Manitoba
Microplitis varicolor|DSWAS778-07|17-Aug-2006|Canada.Manitoba
Microplitis varicolor|DSWAS779-07|17-Aug-2006|Canada.Manitoba
Microplitis varicolor|ASWAV898-08|11-Aug-2007|Canada.Manitoba
Microplitis varicolor|ASWAV913-08|11-Aug-2007|Canada.Manitoba
Microplitis varicolor|ASWAV893-08|11-Aug-2007|Canada.Manitoba
Microplitis varicolor|ASWAY009-08|28-Jun-2007|Canada.Manitoba
Microplitis varicolor|ASWAY002-08|07-Aug-2007|Canada.Manitoba
Microplitis varicolor|ASWAX993-08|07-Aug-2007|Canada.Manitoba
Microplitis varicolor|ASWAX987-08|07-Aug-2007|Canada.Manitoba
Microplitis varicolor|ASWAX985-08|07-Aug-2007|Canada.Manitoba
Microplitis varicolor|ASWAX971-08|23-Jul-2007|Canada.Manitoba
Microplitis varicolor|ASWAX970-08|23-Jul-2007|Canada.Manitoba
Microplitis varicolor|ASWAX968-08|23-Jul-2007|Canada.Manitoba
Microplitis varicolor|ASWAV897-08|11-Aug-2007|Canada.Manitoba
Microplitis varicolor|ASWAV889-08|11-Aug-2007|Canada.Manitoba
Microplitis varicolor|ASWAV896-08|11-Aug-2007|Canada.Manitoba
Microplitis varicolor|ASWAY613-08|19-Aug-2006|Canada.Manitoba
Microplitis varicolor|ASWAY612-08|20-Aug-2006|Canada.Manitoba
Microplitis varicolor|ASWAV895-08|11-Aug-2007|Canada.Manitoba
Microplitis varicolor|ASWAV956-08|16-Jul-2007|Canada.Manitoba
Microplitis varicolor|CNCHZ417-09|30-May-2007|Canada.Ontario
Microplitis varicolor|CNCHZ1172-09||Canada.Ontario
Microplitis varicolor|CNCHZ812-09|06-Aug-2008|Canada.Ontario
Microplitis varicolor|CNCHX201-09||Canada.Ontario
Microplitis varicolor|CNCHX223-09||Canada.Ontario
Microplitis varicolor|CNCHX226-09||Canada.Ontario
Microplitis varicolor|CNCHZ390-09|19-Sep-2007|Canada.Ontario
Microplitis varicolor|CNCHZ395-09|30-May-2007|Canada.Ontario
Microplitis varicolor|CNCHZ397-09|19-Oct-2007|Canada.Ontario
Microplitis varicolor|CNCHZ438-09|10-Aug-2007|Canada.Ontario
Microplitis varicolor|CNCHZ511-09|29-May-2008|Canada.Ontario
Microplitis jft12|CNCHZ083-09|25-Jun-1971|Canada.Northwest Territories
Microplitis jft12|ASWAY609-08|07-Aug-1952|Canada.Manitoba
Microplitis jft12|ASWAV961-08|16-Jul-2007|Canada.Manitoba
Microplitis jft12|ASWAV965-08|16-Jul-2007|Canada.Manitoba
Microplitis jft12|CNCHZ187-09|13-Jun-1962|Canada.Yukon Territory
Microplitis jft12|ASWAY608-08|26-Jun-1952|Canada.Manitoba
Microplitis varicolor|ASWAY611-08|17-Aug-2006|Canada.Manitoba
Braconidae|ASWAY566-08|21-Jul-1937|Canada.Manitoba
Apanteles ensiger|ASWAY519-08|17-Aug-2006|Canada.Manitoba
Apanteles fumiferanae|ASWAY520-08|17-Aug-2006|Canada.Manitoba
Dolichogenidea jft07|ASWAY534-08|08-Jun-1952|Canada.Manitoba
Dolichogenidea jft07|ASWAY540-08|08-Jun-1952|Canada.Manitoba
Dolichogenidea jft07|ASWAY536-08|21-Jul-1952|Canada.Manitoba
Dolichogenidea jft07|ASWAY539-08|21-Jul-1952|Canada.Manitoba
Dolichogenidea jft08|ASWAY544-08|21-Jul-1952|Canada.Manitoba
Microplitis varicolor|CNCHZ117-09|20-Jul-1952|United States.Alaska
Microplitis varicolor|CNCHZ188-09|21-Jun-1960|Canada.Yukon Territory
Microplitis jft06|ASWAV883-08|11-Aug-2007|Canada.Manitoba
Microplitis jft06|ASWAV912-08|11-Aug-2007|Canada.Manitoba
Microplitis jft06|ASWA726-08||Canada.Yukon Territory
Microplitis jft06|CNCHY227-07|13-Jul-2006|Canada.Yukon Territory
Microplitis jft06|CNCHY282-07|18-Jul-2006|Canada.Yukon Territory
Microplitis jft03|DSWAS867-07|07-Aug-1952|Canada.Manitoba
Microplitis jft20|ASWA520-08|03-Jun-2007|Canada.Saskatchewan
Dolichogenidea jft16|CNCHZ072-09|18-Jun-1966|Canada.Northwest Territories
Microplitis jft30|CNCHZ084-09|23-Jun-1966|Canada.Northwest Territories
Microplitis jft04|DSWAS772-07|17-Aug-2006|Canada.Manitoba
Microplitis jft04|DSWAS771-07|19-Aug-2006|Canada.Manitoba
Microplitis jft04|DSWAS770-07|17-Aug-2006|Canada.Manitoba
Microplitis jft04|DSWAS769-07|19-Aug-2006|Canada.Manitoba
Microplitis jft04|DSWAS768-07|19-Aug-2006|Canada.Manitoba
Microplitis jft04|DSWAS736-07||Canada.Manitoba
Microplitis jft04|DSWAS773-07|17-Aug-2006|Canada.Manitoba
Microplitis jft04|DSWAS767-07|17-Aug-2006|Canada.Manitoba
Microplitis jft04|DSWAS766-07|19-Aug-2006|Canada.Manitoba
Microplitis jft04|ASWAV890-08|11-Aug-2007|Canada.Manitoba
Microplitis jft04|ASWAV937-08|08-Aug-2007|Canada.Manitoba
Microplitis jft04|ASWAV911-08|11-Aug-2007|Canada.Manitoba
Microplitis jft04|ASWAV909-08|11-Aug-2007|Canada.Manitoba
Microplitis jft04|ASWAX996-08|07-Aug-2007|Canada.Manitoba
Microplitis jft04|ASWAX969-08|23-Jul-2007|Canada.Manitoba
Microplitis jft04|ASWAX958-08|17-Jul-2007|Canada.Manitoba
Microplitis jft04|ASWAV870-08|02-Aug-2007|Canada.Manitoba
Microplitis jft04|ASWAX955-08|17-Jul-2007|Canada.Manitoba
Microplitis jft04|DSWAS869-07|17-Aug-2006|Canada.Manitoba
Microplitis jft04|CNCHZ098-09|12-Jul-1953|Canada.Nunavut
Microplitis jft04|CNCHZ099-09|14-Jun-1956|Canada.Quebec
Microplitis jft09|CNCHX206-09||Canada.Ontario
Microplitis jft09|ASWA222-08|28-Jul-2007|Canada.Manitoba
Microplitis jft09|CNCHX196-09||Canada.Ontario
Microplitis jft09|CNCHX204-09||Canada.Ontario
Microplitis jft09|CNCHX205-09||Canada.Ontario
Microplitis jft09|CNCHX208-09||Canada.Ontario
Microplitis jft09|CNCHX210-09||Canada.Ontario
Microplitis jft09|CNCHX211-09||Canada.Ontario
Microplitis jft09|CNCHX215-09||Canada.Ontario
Microplitis jft09|CNCHX217-09||Canada.Ontario
Microplitis jft09|CNCHX220-09||Canada.Ontario
Microplitis jft09|CNCHX797-09|07-Jul-2003|Canada.Ontario
Microplitis jft09|CNCHZ359-09|10-Aug-2007|Canada.Ontario
Microplitis jft09|CNCHZ385-09|19-Sep-2007|Canada.Ontario
Microplitis jft32|CNCHZ030-09|27-Jul-1982|Canada.Nunavut
Microplitis jft08|CNCHY236-07|05-Jul-2006|Canada.Yukon Territory

[illegible]
Microplitis jft09|CNCHZ385-09|19-Sep-2007|Canada.Ontario
Microplitis jft32|CNCHZ030-09|27-Jul-1982|Canada.Nunavut
Microplitis jft08|CNCHY236-07|05-Jul-2006|Canada.Yukon Territory
Microplitis jft08|ASWA970-08||Canada.Manitoba
Microplitis jft08|ASWA983-08||Canada.Manitoba
Microplitis jft08|ASWA1032-08|17-Jul-2007|Canada.Manitoba
Microplitis jft08|CNCHY076-07|11-Jul-2006|Canada.Yukon Territory
Microplitis jft08|CNCHY078-07|11-Jul-2006|Canada.Yukon Territory
Microplitis jft08|ASWA1035-08|17-Jul-2007|Canada.Manitoba
Microplitis jft08|ASWA944-08|03-Jul-2007|Canada.Manitoba
Microplitis jft08|ASWA942-08|03-Jul-2007|Canada.Manitoba
Microplitis jft08|ASWAV973-08|16-Jul-2007|Canada.Manitoba
Microplitis jft08|CNCHY248-07|05-Jul-2006|Canada.Yukon Territory
Microplitis jft08|CNCHZ079-09|18-Jun-1966|Canada.Northwest Territories
Microplitis jft08|CNCHZ103-09|08-Jul-1959|Canada.British Columbia
Microplitis jft07|CNCHZ1171-09||Canada.Ontario
Microplitis jft07|ASWAX976-08|23-Jul-2007|Canada.Manitoba
Microplitis jft07|ASWAX973-08|23-Jul-2007|Canada.Manitoba
Microplitis jft07|CNCHZ837-09|26-Jul-2008|Canada.Quebec
Microplitis jft07|CNCHZ838-09|26-Jul-2008|Canada.Quebec
Microplitis jft07|CNCHZ839-09|26-Jul-2008|Canada.Quebec
Microplitis jft41|CNCHZ032-09|15-Jul-1971|Canada.Nunavut
Microplitis jft41|CNCHZ089-09|14-Aug-1950|Canada.Northwest Territories
Microplitis jft38|CNCHZ185-09|27-Jul-1971|Canada.Yukon Territory
Microplitis jft38|CNCHZ186-09|27-Jul-1971|Canada.Yukon Territory
Microplitis jft11|ASWAY607-08|05-Aug-1952|Canada.Manitoba
Microplitis jft11|CNCHZ097-09|20-Jun-1955|Canada.Quebec
Microplitis jft17|CNCHZ157-09|03-Jul-1959|United States.Alaska
Microplitis jft10|ASWAT763-08|07-Jul-2007|Canada.Manitoba
Microplitis jft10|ASWAV858-08|12-Jul-2007|Canada.Manitoba
Microplitis jft10|ASWAY654-08|21-Jun-1952|Canada.Manitoba
Microplitis jft40|CNCHZ096-09|12-Jul-1959|Canada.British Columbia
Microplitis jft02|DSWAS866-07|17-Aug-2006|Canada.Manitoba
Microplitis jft02|ASWAX962-08|23-Jul-2007|Canada.Manitoba
Microplitis jft02|ASWAX981-08|23-Jul-2007|Canada.Manitoba
Microplitis jft02|ASWAX974-08|23-Jul-2007|Canada.Manitoba
Microplitis jft02|ASWAX972-08|23-Jul-2007|Canada.Manitoba
Microplitis jft02|ASWAX960-08|23-Jul-2007|Canada.Manitoba
Microplitis jft02|ASWAV940-08|08-Aug-2007|Canada.Manitoba
Microplitis jft02|ASWAV960-08|16-Jul-2007|Canada.Manitoba
Microplitis jft02|CNCHZ125-09|31-Aug-1968|United States.Alaska
Microplitis auripes|CNCHY242-07|10-Jul-2006|Canada.Yukon Territory
Microplitis auripes|CNCHY070-07|10-Jul-2006|Canada.Yukon Territory
Microplitis auripes|ASWA518-08|10-Jun-2007|Canada.Ontario
Microplitis auripes|CNCHY243-07|16-Jul-2006|Canada.Yukon Territory
Microplitis auripes|CNCHY244-07|16-Jul-2006|Canada.Yukon Territory
Microplitis jft01|ASWAY622-08|23-Jun-1952|Canada.Manitoba
Microplitis jft01|ASWAY638-08|23-Jun-1952|Canada.Manitoba
Microplitis jft01|CNCHZ029-09|11-Jul-1980|Canada.Nunavut
Microplitis jft01|ASWAY635-08|16-Jun-1952|Canada.Manitoba
Microplitis jft01|ASWAY632-08|23-Jun-1952|Canada.Manitoba
Microplitis jft01|ASWAY623-08|23-Jun-1952|Canada.Manitoba
Microplitis jft01|ASWAY627-08|23-Jun-1952|Canada.Manitoba
Microplitis jft01|ASWAY628-08|23-Jun-1952|Canada.Manitoba
Microplitis jft01|ASWAY630-08|23-Jun-1952|Canada.Manitoba
Microplitis jft01|ASWAY631-08|23-Jun-1952|Canada.Manitoba
Microplitis jft01|ASWAY640-08|23-Jun-1952|Canada.Manitoba
Microplitis jft01|CNCHZ031-09|19-Jul-1966|Greenland
Microplitis jft01|ASWAS973-07|23-Jun-1952|Canada.Manitoba
Microplitis jft01|ASWAY637-08|23-Jun-1952|Canada.Manitoba
Microplitis jft01|ASWAY636-08|23-Jun-1952|Canada.Manitoba
Microplitis jft01|ASWAY633-08|18-Jun-1952|Canada.Manitoba
Microplitis jft01|ASWAY625-08|23-Jun-1952|Canada.Manitoba
Microplitis jft01|ASWAY639-08|29-Jun-1952|Canada.Manitoba
Microplitis jft01|CNCHZ081-09|20-Jun-1971|Canada.Northwest Territories
Microplitis jft01|CNCHZ090-09|22-Jul-1951|Canada.Northwest Territories
Microplitis jft01|CNCHZ119-09|18-Jul-1962|United States.Alaska
Microplitis jft45|CNCHZ080-09|30-Jun-1953|Canada.Northwest Territories
Glyptapanteles|CNCHZ144-09|31-Jul-1958|Canada.Quebec
Glyptapanteles|CNCHZ135-09|29-Jul-1958|Canada.Quebec
Glyptapanteles|CNCHZ143-09|09-Aug-1958|Canada.Quebec
Glyptapanteles|CNCHZ147-09|22-Jul-1954|Canada.Quebec
Glyptapanteles|CNCHZ057-09|09-Jul-1949|Canada.Northwest Territories
Glyptapanteles|CNCHZ162-09|14-Aug-1961|United States.Alaska
Glyptapanteles jft01|ASWAY010-08|25-Jul-2007|Canada.Manitoba
Glyptapanteles jft01|DSWAS815-07|17-Aug-2006|Canada.Manitoba
Glyptapanteles jft01|DSWAS819-07|17-Aug-2006|Canada.Manitoba
Glyptapanteles jft01|DSWAS826-07|17-Aug-2006|Canada.Manitoba
Glyptapanteles jft01|DSWAS813-07|17-Aug-2006|Canada.Manitoba
Glyptapanteles jft01|DSWAS830-07|17-Aug-2006|Canada.Manitoba
Glyptapanteles jft01|DSWAS828-07|17-Aug-2006|Canada.Manitoba
Glyptapanteles jft01|DSWAS820-07|17-Aug-2006|Canada.Manitoba
Glyptapanteles jft01|DSWAS827-07|17-Aug-2006|Canada.Manitoba
Glyptapanteles jft01|DSWAS823-07|17-Aug-2006|Canada.Manitoba
Glyptapanteles jft01|DSWAS821-07|17-Aug-2006|Canada.Manitoba
Glyptapanteles jft01|DSWAS811-07|17-Aug-2006|Canada.Manitoba
Glyptapanteles jft01|ASWAV962-08|16-Jul-2007|Canada.Manitoba
Glyptapanteles jft01|ASWAY034-08|05-Jul-2007|Canada.Manitoba
Glyptapanteles jft01|ASWAY026-08|05-Jul-2007|Canada.Manitoba
Glyptapanteles jft01|ASWAY025-08|05-Jul-2007|Canada.Manitoba
Glyptapanteles jft01|ASWAY022-08|05-Jul-2007|Canada.Manitoba
Glyptapanteles jft01|ASWAY006-08|28-Jun-2007|Canada.Manitoba
Glyptapanteles jft01|ASWAY644-08|20-Aug-2006|Canada.Manitoba
Glyptapanteles jft01|ASWA941-08|03-Jul-2007|Canada.Manitoba
Glyptapanteles jft01|ASWAT766-08|07-Jul-2007|Canada.Manitoba
Glyptapanteles jft01|ASWAV931-08|08-Aug-2007|Canada.Manitoba
Glyptapanteles jft01|ASWA1028-08|17-Jul-2007|Canada.Manitoba

Glyptapanteles jft01|ASWAT766-08|07-Jul-2007|Canada.Manitoba
Glyptapanteles jft01|ASWAV931-08|08-Aug-2007|Canada.Manitoba
Glyptapanteles jft01|ASWA1028-08|17-Jul-2007|Canada.Manitoba
Glyptapanteles jft01|CNCHZ051-09|09-Jul-1971|Canada.Northwest Territories
Glyptapanteles jft01|CNCHZ053-09|30-Jul-1979|Canada.Northwest Territories
Glyptapanteles jft01|CNCHZ054-09|22-Jun-1971|Canada.Northwest Territories
Glyptapanteles jft01|CNCHZ055-09|04-Jul-1953|Canada.Northwest Territories
Diolcogaster|CNCHZ065-09|13-Jul-1971|Canada.Northwest Territories
Diolcogaster|CNCHZ066-09|17-Jul-1955|Canada.Newfoundland and Labrador
Diolcogaster|CNCHZ108-09|13-Jun-1948|Canada.British Columbia
Glyptapanteles jft05|CNCHZ023-09|30-Jul-1968|Canada.Northwest Territories
Glyptapanteles jft05|DSWAS818-07|17-Aug-2006|Canada.Manitoba
Glyptapanteles jft05|DSWAS829-07|17-Aug-2006|Canada.Manitoba
Glyptapanteles jft05|DSWAS825-07|17-Aug-2006|Canada.Manitoba
Glyptapanteles jft05|DSWAS824-07|17-Aug-2006|Canada.Manitoba
Glyptapanteles jft05|DSWAS807-07|17-Aug-2006|Canada.Manitoba
Glyptapanteles jft06|ASWAV910-08|11-Aug-2007|Canada.Manitoba
Glyptapanteles jft05|DSWAS806-07|17-Aug-2006|Canada.Manitoba
Glyptapanteles jft05|DSWAS822-07|17-Aug-2006|Canada.Manitoba
Glyptapanteles jft05|DSWAS816-07|17-Aug-2006|Canada.Manitoba
Glyptapanteles jft05|CNCHZ017-09|07-Aug-1935|Canada.Nunavut
Glyptapanteles jft05|DSWAS817-07|17-Aug-2006|Canada.Manitoba
Glyptapanteles jft05|DSWAS805-07|17-Aug-2006|Canada.Manitoba
Glyptapanteles jft05|CNCHZ094-09|16-Aug-1954|Canada.Quebec
Glyptapanteles jft05|CNCHZ027-09|14-Aug-1963|Canada.Nunavut
Glyptapanteles jft05|CNCHZ020-09|31-Jul-1948|Canada.Nunavut
Glyptapanteles jft05|CNCHZ026-09|03-Aug-1966|Greenland
Glyptapanteles jft05|ASWAY651-08|20-Aug-2006|Canada.Manitoba
Glyptapanteles jft05|ASWAY652-08|20-Aug-2006|Canada.Manitoba
Glyptapanteles jft05|ASWAY650-08|20-Aug-2006|Canada.Manitoba
Glyptapanteles jft05|ASWAY648-08|14-Aug-2006|Canada.Manitoba
Glyptapanteles jft05|ASWAY647-08|20-Aug-2006|Canada.Manitoba
Glyptapanteles jft05|ASWAY646-08|20-Aug-2006|Canada.Manitoba
Glyptapanteles jft05|ASWAY645-08|14-Aug-2006|Canada.Manitoba
Glyptapanteles jft05|ASWAY643-08|20-Aug-2006|Canada.Manitoba
Glyptapanteles jft05|ASWAY642-08|20-Aug-2006|Canada.Manitoba
Glyptapanteles jft05|ASWAY641-08|20-Aug-2006|Canada.Manitoba
Glyptapanteles jft05|ASWAY653-08|19-Aug-2006|Canada.Manitoba
Glyptapanteles jft05|CNCHZ013-09|27-Jul-1982|Canada.Nunavut
Glyptapanteles jft05|CNCHZ148-09|17-Aug-1959|Canada.Quebec
Glyptapanteles|CNCHZ012-09|19-Jun-1951|Canada.Nunavut
Glyptapanteles|CNCHZ018-09|20-Jul-1963|Canada.Nunavut
Glyptapanteles|CNCHZ022-09|19-Jun-1951|Canada.Nunavut
Glyptapanteles jft09|ASWAY649-08|08-Sep-2006|Canada.Manitoba
Glyptapanteles jft09|CNCHZ015-09|16-Jul-1968|Canada.Northwest Territories
Glyptapanteles jft09|CNCHZ021-09|07-Aug-1958|Canada.Nunavut
Glyptapanteles jft07|ASWAY033-08|05-Jul-2007|Canada.Manitoba
Glyptapanteles jft07|ASWA1034-08|17-Jul-2007|Canada.Manitoba
Glyptapanteles jft07|CNCHZ058-09|02-Jul-1966|Canada.Northwest Territories
Glyptapanteles jft07|CNCHZ091-09|02-Jul-1966|Canada.Northwest Territories
Glyptapanteles jft07|CNCHZ019-09|03-Jul-1975|Canada.Northwest Territories
Glyptapanteles jft07|CNCHZ011-09|03-Jul-1958|Canada.Nunavut
Glyptapanteles jft07|CNCHZ014-09|13-Jul-1975|Canada.Northwest Territories
Glyptapanteles jft07|CNCHZ050-09|03-Aug-1950|Canada.Nunavut
Sathon|CNCHZ161-09|11-Jul-1951|United States.Alaska
Sathon|CNCHZ156-09|05-Aug-1961|United States.Alaska
Sathon|CNCHZ165-09|25-Jul-1952|United States.Alaska
Glyptapanteles compressiventris|ASWAY618-08|11-Jun-1952|Canada.Manitoba
Glyptapanteles compressiventris|ASWAY617-08|17-Jun-1952|Canada.Manitoba
Glyptapanteles compressiventris|ASWAY616-08|07-Aug-1952|Canada.Manitoba
Glyptapanteles compressiventris|ASWAY614-08|11-Jun-1952|Canada.Manitoba
Glyptapanteles compressiventris|ASWAY619-08|11-Jun-1952|Canada.Manitoba
Sathon|CNCHZ163-09|21-Jul-1952|United States.Alaska
Glyptapanteles jft03|WSPAH043-06|29-Jul-2005|Canada.Manitoba
Glyptapanteles jft03|ASWAV881-08|11-Aug-2007|Canada.Manitoba
Sathon|CNCHZ166-09|21-Jul-1952|United States.Alaska
Dolichogenidea jft02|CNCHZ116-09|03-Aug-1952|United States.Alaska
Dolichogenidea jft02|CNCHZ168-09|24-Jun-1949|Canada.Yukon Territory
Dolichogenidea jft02|CNCHZ115-09|30-Jun-1951|United States.Alaska
Dolichogenidea jft02|CNCHZ076-09|28-Jun-1966|Canada.Northwest Territories
Dolichogenidea jft02|CNCHZ159-09|30-Jun-1951|United States.Alaska
Dolichogenidea jft02|ASWAY027-08|05-Jul-2007|Canada.Manitoba
Dolichogenidea jft02|CNCHZ841-09|26-Jul-2008|Canada.Quebec
Dolichogenidea jft02|ASWAU498-08|13-Jul-2006|Canada.Yukon Territory
Dolichogenidea jft02|CNCHY345-07|14-Jul-2006|Canada.Yukon Territory
Dolichogenidea jft02|ASWAV951-08|08-Aug-2007|Canada.Manitoba
Dolichogenidea jft02|CNCHZ1154-09||Canada.Ontario
Dolichogenidea jft02|ASWAV952-08|16-Jul-2007|Canada.Manitoba
Dolichogenidea jft02|ASWAV948-08|08-Aug-2007|Canada.Manitoba
Dolichogenidea jft02|ASWAV927-08|08-Aug-2007|Canada.Manitoba
Dolichogenidea jft02|ASWAY016-08|25-Jul-2007|Canada.Manitoba
Dolichogenidea jft02|ASWAV949-08|08-Aug-2007|Canada.Manitoba
Dolichogenidea jft02|ASWAV917-08|08-Aug-2007|Canada.Manitoba
Dolichogenidea jft02|ASWAV943-08|08-Aug-2007|Canada.Manitoba
Dolichogenidea jft02|CNCHY344-07|19-Jul-2006|Canada.Yukon Territory
Dolichogenidea jft02|CNCHY340-07|19-Jul-2006|Canada.Yukon Territory
Dolichogenidea jft02|ASWAU334-08|15-Jul-2006|Canada.Yukon Territory
Dolichogenidea jft02|CNCHY346-07|08-Jul-2006|Canada.Yukon Territory
Dolichogenidea jft02|CNCHY314-07|16-Jul-2006|Canada.Yukon Territory
Dolichogenidea jft02|ASWAU315-08|15-Jul-2006|Canada.Yukon Territory
Dolichogenidea jft02|CNCHY358-07|08-Jul-2006|Canada.Yukon Territory
Dolichogenidea jft02|CNCHZ175-09|06-Aug-1973|Canada.Yukon Territory
Dolichogenidea jft02|ASWAY528-08|29-Jun-1937|Canada.Manitoba
Dolichogenidea jft02|ASWAY529-08|16-Aug-1952|Canada.Manitoba
Dolichogenidea jft02|ASWAY531-08|29-Jun-1937|Canada.Manitoba
Dolichogenidea jft02|ASWAY593-08|05-Aug-1952|Canada.Manitoba
Dolichogenidea jft03|CNCHY061-07|10-Jul-2006|Canada.Yukon Territory
Dolichogenidea jft03|ASWAY549-08|03-Aug-1952|Canada.Manitoba

[illegible]
Dolichogenidea jft02|ASWAY593-08|05-Aug-1952|Canada.Manitoba
Dolichogenidea jft03|CNCHY061-07|10-Jul-2006|Canada.Yukon Territory
Dolichogenidea jft03|ASWAY549-08|03-Aug-1952|Canada.Manitoba
Dolichogenidea jft03|CNCHZ169-09|19-Aug-1962|Canada.Yukon Territory
Dolichogenidea jft03|CNCHZ145-09|13-Jun-1961|United States.Alaska
Dolichogenidea jft03|CNCHZ086-09|16-Aug-1949|Canada.Northwest Territories
Dolichogenidea jft03|CNCHZ074-09|02-Jul-1957|Canada.Northwest Territories
Dolichogenidea jft03|CNCHZ170-09|16-Aug-1956|Canada.Yukon Territory
Dolichogenidea jft03|ASWAV884-08|11-Aug-2007|Canada.Manitoba
Dolichogenidea jft03|ASWAV891-08|11-Aug-2007|Canada.Manitoba
Dolichogenidea jft03|ASWAV905-08|11-Aug-2007|Canada.Manitoba
Dolichogenidea jft03|ASWAV886-08|11-Aug-2007|Canada.Manitoba
Dolichogenidea jft03|ASWAX989-08|07-Aug-2007|Canada.Manitoba
Dolichogenidea jft03|ASWAV906-08|11-Aug-2007|Canada.Manitoba
Dolichogenidea jft03|CNCHY351-07|15-Jul-2006|Canada.Yukon Territory
Dolichogenidea jft03|CNCHY317-07|15-Jul-2006|Canada.Yukon Territory
Dolichogenidea jft03|CNCHY304-07|15-Jul-2006|Canada.Yukon Territory
Dolichogenidea jft03|CNCHY295-07|15-Jul-2006|Canada.Yukon Territory
Dolichogenidea jft03|CNCHY291-07|15-Jul-2006|Canada.Yukon Territory
Dolichogenidea jft03|CNCHY297-07|15-Jul-2006|Canada.Yukon Territory
Dolichogenidea jft03|ASWAY601-08|15-Aug-2006|Canada.Manitoba
Dolichogenidea jft03|ASWAY694-08|17-Aug-2006|Canada.Manitoba
Dolichogenidea jft03|ASWAY693-08|15-Aug-2006|Canada.Manitoba
Dolichogenidea jft03|ASWAY692-08|15-Aug-2006|Canada.Manitoba
Dolichogenidea jft03|CNCHY349-07|15-Jul-2006|Canada.Yukon Territory
Dolichogenidea jft03|CNCHY348-07|15-Jul-2006|Canada.Yukon Territory
Dolichogenidea jft03|CNCHY347-07|05-Jul-2006|Canada.Yukon Territory
Dolichogenidea jft03|CNCHY341-07|19-Jul-2006|Canada.Yukon Territory
Dolichogenidea jft03|CNCHY335-07|18-Jul-2006|Canada.Yukon Territory
Dolichogenidea jft03|CNCHY334-07|05-Jul-2006|Canada.Yukon Territory
Dolichogenidea jft03|CNCHY306-07|06-Jul-2006|Canada.Yukon Territory
Dolichogenidea jft03|CNCHY305-07|15-Jul-2006|Canada.Yukon Territory
Dolichogenidea jft03|CNCHY303-07|15-Jul-2006|Canada.Yukon Territory
Dolichogenidea jft03|CNCHY302-07|15-Jul-2006|Canada.Yukon Territory
Dolichogenidea jft03|CNCHY300-07|15-Jul-2006|Canada.Yukon Territory
Dolichogenidea jft03|CNCHY294-07|15-Jul-2006|Canada.Yukon Territory
Dolichogenidea jft03|CNCHY292-07|15-Jul-2006|Canada.Yukon Territory
Dolichogenidea jft03|CNCHY286-07|15-Jul-2006|Canada.Yukon Territory
Dolichogenidea jft03|CNCHY284-07|15-Jul-2006|Canada.Yukon Territory
Dolichogenidea jft03|ASWAU491-08|12-Jul-2006|Canada.Yukon Territory
Dolichogenidea jft03|ASWAU323-08|15-Jul-2006|Canada.Yukon Territory
Dolichogenidea jft03|ASWAU332-08|15-Jul-2006|Canada.Yukon Territory
Dolichogenidea jft03|ASWAU337-08|15-Jul-2006|Canada.Yukon Territory
Dolichogenidea jft03|ASWAU343-08|15-Jul-2006|Canada.Yukon Territory
Dolichogenidea jft03|ASWAU312-08|15-Jul-2006|Canada.Yukon Territory
Dolichogenidea jft03|ASWAU340-08|15-Jul-2006|Canada.Yukon Territory
Dolichogenidea jft03|ASWAU328-08|15-Jul-2006|Canada.Yukon Territory
Dolichogenidea jft03|ASWAU314-08|10-Jul-2006|Canada.Yukon Territory
Dolichogenidea jft03|ASWAU333-08|15-Jul-2006|Canada.Yukon Territory
Dolichogenidea jft03|ASWAU344-08|15-Jul-2006|Canada.Yukon Territory
Dolichogenidea jft03|ASWAU346-08|15-Jul-2006|Canada.Yukon Territory
Dolichogenidea jft03|ASWAU351-08|15-Jul-2006|Canada.Yukon Territory
Dolichogenidea jft03|CNCHY350-07|15-Jul-2006|Canada.Yukon Territory
Dolichogenidea jft03|CNCHY352-07|15-Jul-2006|Canada.Yukon Territory
Dolichogenidea jft03|CNCHY353-07|16-Jul-2006|Canada.Yukon Territory
Dolichogenidea jft03|DSWAS959-07|15-Jul-2006|Canada.Yukon Territory
Dolichogenidea jft03|CNCHZ177-09|17-Jul-1948|Canada.British Columbia
Dolichogenidea jft03|ASWAY548-08|05-Sep-1948|Canada.Manitoba
Dolichogenidea jft03|ASWAY551-08|08-Jul-1937|Canada.Manitoba
Dolichogenidea jft03|ASWAY559-08|29-Jun-1952|Canada.Manitoba
Dolichogenidea jft03|CNCHZ001-09|21-Jul-1963|Canada.Nunavut
Dolichogenidea jft03|CNCHZ006-09|25-Jul-1962|Canada.Nunavut
Dolichogenidea jft03|CNCHZ009-09|07-Aug-1958|Canada.Nunavut
Dolichogenidea jft01|DSWAS848-07|04-Jun-1952|Canada.Manitoba
Dolichogenidea jft01|CNCHZ172-09|02-Aug-1962|Canada.Yukon Territory
Dolichogenidea jft01|ASWAY554-08|23-Jul-1952|Canada.Manitoba
Dolichogenidea jft01|ASWAY552-08|11-Jun-1952|Canada.Manitoba
Dolichogenidea jft01|ASWAY513-08|05-Aug-1952|Canada.Manitoba
Dolichogenidea jft01|ASWAY512-08|23-Jul-1952|Canada.Manitoba
Dolichogenidea jft01|CNCHY324-07|06-Jul-2006|Canada.Yukon Territory
Dolichogenidea jft01|ASWAV946-08|08-Aug-2007|Canada.Manitoba
Dolichogenidea jft01|ASWAY017-08|25-Jul-2007|Canada.Manitoba
Dolichogenidea jft01|ASWAV922-08|08-Aug-2007|Canada.Manitoba
Dolichogenidea jft01|ASWAV942-08|08-Aug-2007|Canada.Manitoba
Dolichogenidea jft01|ASWAY553-08|11-Jun-1952|Canada.Manitoba
Dolichogenidea jft01|ASWAY556-08|03-Aug-1952|Canada.Manitoba
Dolichogenidea jft01|ASWAY557-08|18-Jun-1952|Canada.Manitoba
Dolichogenidea jft04|ASWAX998-08|07-Aug-2007|Canada.Manitoba
Dolichogenidea jft04|CNCHZ075-09|21-Jul-1951|Canada.Northwest Territories
Dolichogenidea jft11|CNCHZ007-09|29-Jul-1968|Canada.Northwest Territories
Cotesia jft03|ASWA224-08|28-Jul-2007|Canada.Manitoba
Cotesia jft03|ASWA491-08|15-Jun-2004|Canada.Ontario
Cotesia jft03|ASWA492-08|15-Jun-2004|Canada.Ontario
Cotesia jft24|CNCHZ111-09|12-Jul-1959|Canada.British Columbia
Cotesia jft05|ASWAY018-08|25-Jul-2007|Canada.Manitoba
Cotesia jft05|ASWAY015-08|25-Jul-2007|Canada.Manitoba
Cotesia jft05|ASWAY011-08|25-Jul-2007|Canada.Manitoba
Cotesia jft05|ASWAX963-08|23-Jul-2007|Canada.Manitoba
Cotesia jft05|ASWAV915-08|08-Aug-2007|Canada.Manitoba
Cotesia jft05|ASWAV935-08|08-Aug-2007|Canada.Manitoba
Cotesia jft05|ASWAY014-08|25-Jul-2007|Canada.Manitoba
Cotesia jft05|ASWAY568-08|17-Aug-2006|Canada.Manitoba
Cotesia jft05|ASWAY569-08|01-Aug-2005|Canada.Manitoba
Cotesia jft05|ASWAV966-08|16-Jul-2007|Canada.Manitoba
Cotesia jft25|CNCHZ149-09|09-Aug-1954|Canada.Newfoundland and Labrador
Cotesia jft08|CNCHZ034-09|06-Jul-1953|Canada.Nunavut
Cotesia jft08|CNCHZ036-09|15-Jul-1982|Canada.Nunavut

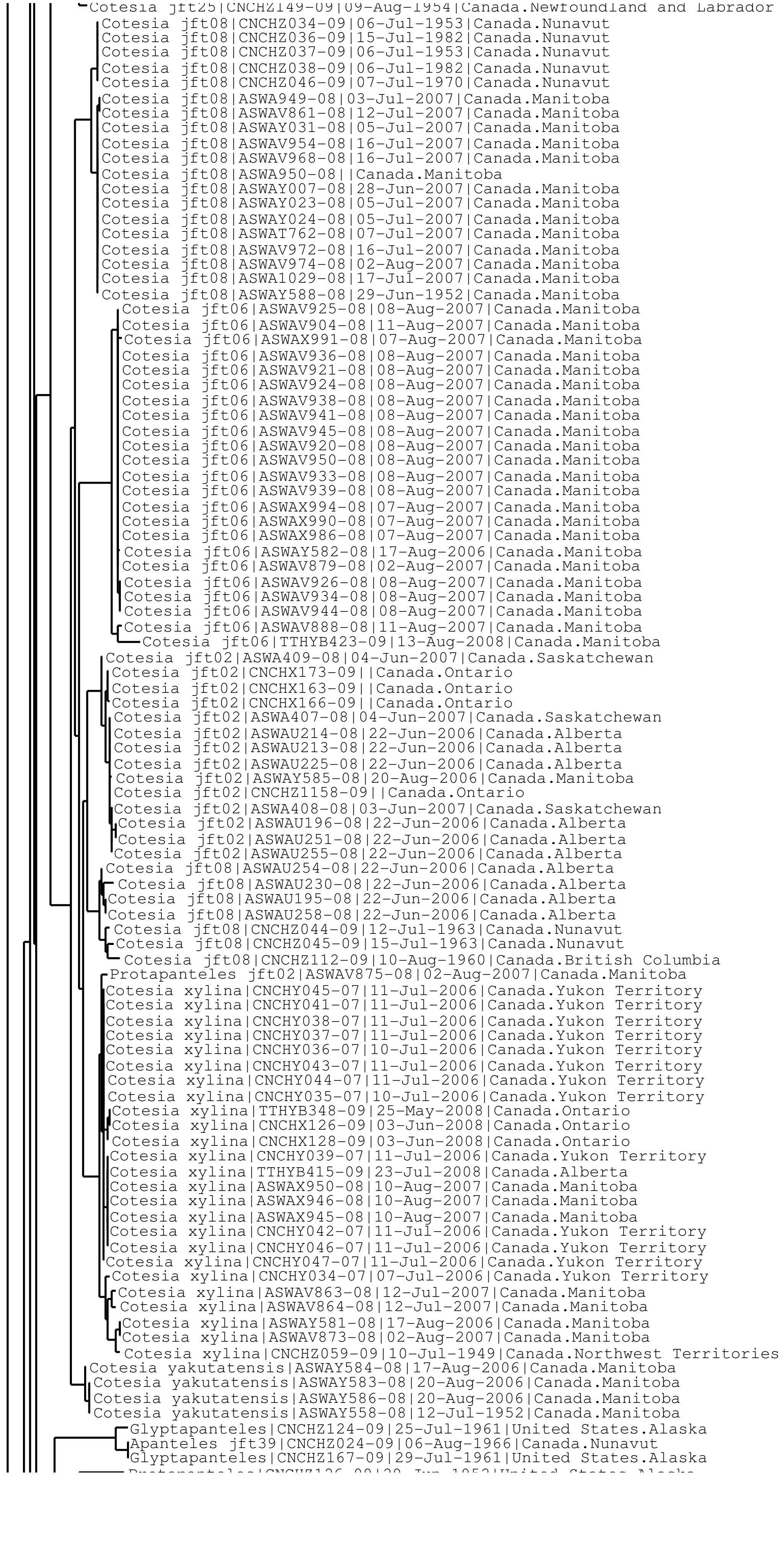
Cotesia jft25|CNCHZ149-09|09-Aug-1954|Canada.Newfoundland and Labrador
Cotesia jft08|CNCHZ034-09|06-Jul-1953|Canada.Nunavut
Cotesia jft08|CNCHZ036-09|15-Jul-1982|Canada.Nunavut
Cotesia jft08|CNCHZ037-09|06-Jul-1953|Canada.Nunavut
Cotesia jft08|CNCHZ038-09|06-Jul-1982|Canada.Nunavut
Cotesia jft08|CNCHZ046-09|07-Jul-1970|Canada.Nunavut
Cotesia jft08|ASWA949-08|03-Jul-2007|Canada.Manitoba
Cotesia jft08|ASWAV861-08|12-Jul-2007|Canada.Manitoba
Cotesia jft08|ASWAY031-08|05-Jul-2007|Canada.Manitoba
Cotesia jft08|ASWAV954-08|16-Jul-2007|Canada.Manitoba
Cotesia jft08|ASWAV968-08|16-Jul-2007|Canada.Manitoba
Cotesia jft08|ASWA950-08||Canada.Manitoba
Cotesia jft08|ASWAY007-08|28-Jun-2007|Canada.Manitoba
Cotesia jft08|ASWAY023-08|05-Jul-2007|Canada.Manitoba
Cotesia jft08|ASWAY024-08|05-Jul-2007|Canada.Manitoba
Cotesia jft08|ASWAT762-08|07-Jul-2007|Canada.Manitoba
Cotesia jft08|ASWAV972-08|16-Jul-2007|Canada.Manitoba
Cotesia jft08|ASWAV974-08|02-Aug-2007|Canada.Manitoba
Cotesia jft08|ASWA1029-08|17-Jul-2007|Canada.Manitoba
Cotesia jft08|ASWAY588-08|29-Jun-1952|Canada.Manitoba
Cotesia jft06|ASWAV925-08|08-Aug-2007|Canada.Manitoba
Cotesia jft06|ASWAV904-08|11-Aug-2007|Canada.Manitoba
Cotesia jft06|ASWAX991-08|07-Aug-2007|Canada.Manitoba
Cotesia jft06|ASWAV936-08|08-Aug-2007|Canada.Manitoba
Cotesia jft06|ASWAV921-08|08-Aug-2007|Canada.Manitoba
Cotesia jft06|ASWAV924-08|08-Aug-2007|Canada.Manitoba
Cotesia jft06|ASWAV938-08|08-Aug-2007|Canada.Manitoba
Cotesia jft06|ASWAV941-08|08-Aug-2007|Canada.Manitoba
Cotesia jft06|ASWAV945-08|08-Aug-2007|Canada.Manitoba
Cotesia jft06|ASWAV920-08|08-Aug-2007|Canada.Manitoba
Cotesia jft06|ASWAV950-08|08-Aug-2007|Canada.Manitoba
Cotesia jft06|ASWAV933-08|08-Aug-2007|Canada.Manitoba
Cotesia jft06|ASWAV939-08|08-Aug-2007|Canada.Manitoba
Cotesia jft06|ASWAX994-08|07-Aug-2007|Canada.Manitoba
Cotesia jft06|ASWAX990-08|07-Aug-2007|Canada.Manitoba
Cotesia jft06|ASWAX986-08|07-Aug-2007|Canada.Manitoba
Cotesia jft06|ASWAY582-08|17-Aug-2006|Canada.Manitoba
Cotesia jft06|ASWAV879-08|02-Aug-2007|Canada.Manitoba
Cotesia jft06|ASWAV926-08|08-Aug-2007|Canada.Manitoba
Cotesia jft06|ASWAV934-08|08-Aug-2007|Canada.Manitoba
Cotesia jft06|ASWAV944-08|08-Aug-2007|Canada.Manitoba
Cotesia jft06|ASWAV888-08|11-Aug-2007|Canada.Manitoba
Cotesia jft06|TTHYB423-09|13-Aug-2008|Canada.Manitoba
Cotesia jft02|ASWA409-08|04-Jun-2007|Canada.Saskatchewan
Cotesia jft02|CNCHX173-09||Canada.Ontario
Cotesia jft02|CNCHX163-09||Canada.Ontario
Cotesia jft02|CNCHX166-09||Canada.Ontario
Cotesia jft02|ASWA407-08|04-Jun-2007|Canada.Saskatchewan
Cotesia jft02|ASWAU214-08|22-Jun-2006|Canada.Alberta
Cotesia jft02|ASWAU213-08|22-Jun-2006|Canada.Alberta
Cotesia jft02|ASWAU225-08|22-Jun-2006|Canada.Alberta
Cotesia jft02|ASWAY585-08|20-Aug-2006|Canada.Manitoba
Cotesia jft02|CNCHZ1158-09||Canada.Ontario
Cotesia jft02|ASWA408-08|03-Jun-2007|Canada.Saskatchewan
Cotesia jft02|ASWAU196-08|22-Jun-2006|Canada.Alberta
Cotesia jft02|ASWAU251-08|22-Jun-2006|Canada.Alberta
Cotesia jft02|ASWAU255-08|22-Jun-2006|Canada.Alberta
Cotesia jft08|ASWAU254-08|22-Jun-2006|Canada.Alberta
Cotesia jft08|ASWAU230-08|22-Jun-2006|Canada.Alberta
Cotesia jft08|ASWAU195-08|22-Jun-2006|Canada.Alberta
Cotesia jft08|ASWAU258-08|22-Jun-2006|Canada.Alberta
Cotesia jft08|CNCHZ044-09|12-Jul-1963|Canada.Nunavut
Cotesia jft08|CNCHZ045-09|15-Jul-1963|Canada.Nunavut
Cotesia jft08|CNCHZ112-09|10-Aug-1960|Canada.British Columbia
Protapanteles jft02|ASWAV875-08|02-Aug-2007|Canada.Manitoba
Cotesia xylina|CNCHY045-07|11-Jul-2006|Canada.Yukon Territory
Cotesia xylina|CNCHY041-07|11-Jul-2006|Canada.Yukon Territory
Cotesia xylina|CNCHY038-07|11-Jul-2006|Canada.Yukon Territory
Cotesia xylina|CNCHY037-07|11-Jul-2006|Canada.Yukon Territory
Cotesia xylina|CNCHY036-07|10-Jul-2006|Canada.Yukon Territory
Cotesia xylina|CNCHY043-07|11-Jul-2006|Canada.Yukon Territory
Cotesia xylina|CNCHY044-07|11-Jul-2006|Canada.Yukon Territory
Cotesia xylina|CNCHY035-07|10-Jul-2006|Canada.Yukon Territory
Cotesia xylina|TTHYB348-09|25-May-2008|Canada.Ontario
Cotesia xylina|CNCHX126-09|03-Jun-2008|Canada.Ontario
Cotesia xylina|CNCHX128-09|03-Jun-2008|Canada.Ontario
Cotesia xylina|CNCHY039-07|11-Jul-2006|Canada.Yukon Territory
Cotesia xylina|TTHYB415-09|23-Jul-2008|Canada.Alberta
Cotesia xylina|ASWAX950-08|10-Aug-2007|Canada.Manitoba
Cotesia xylina|ASWAX946-08|10-Aug-2007|Canada.Manitoba
Cotesia xylina|ASWAX945-08|10-Aug-2007|Canada.Manitoba
Cotesia xylina|CNCHY042-07|11-Jul-2006|Canada.Yukon Territory
Cotesia xylina|CNCHY046-07|11-Jul-2006|Canada.Yukon Territory
Cotesia xylina|CNCHY047-07|11-Jul-2006|Canada.Yukon Territory
Cotesia xylina|CNCHY034-07|07-Jul-2006|Canada.Yukon Territory
Cotesia xylina|ASWAV863-08|12-Jul-2007|Canada.Manitoba
Cotesia xylina|ASWAV864-08|12-Jul-2007|Canada.Manitoba
Cotesia xylina|ASWAY581-08|17-Aug-2006|Canada.Manitoba
Cotesia xylina|ASWAV873-08|02-Aug-2007|Canada.Manitoba
Cotesia xylina|CNCHZ059-09|10-Jul-1949|Canada.Northwest Territories
Cotesia yakutatensis|ASWAY584-08|17-Aug-2006|Canada.Manitoba
Cotesia yakutatensis|ASWAY583-08|20-Aug-2006|Canada.Manitoba
Cotesia yakutatensis|ASWAY586-08|20-Aug-2006|Canada.Manitoba
Cotesia yakutatensis|ASWAY558-08|12-Jul-1952|Canada.Manitoba
Glyptapanteles|CNCHZ124-09|25-Jul-1961|United States.Alaska
Apanteles jft39|CNCHZ024-09|06-Aug-1966|Canada.Nunavut
Glyptapanteles|CNCHZ167-09|29-Jul-1961|United States.Alaska

Glyptapanteles|CNCHZ121-09|29-Jul-1961|United States.Alaska
Apanteles jft39|CNCHZ024-09|06-Aug-1966|Canada.Nunavut
Glyptapanteles|CNCHZ167-09|29-Jul-1961|United States.Alaska
Protapanteles|CNCHZ126-09|29-Jun-1952|United States.Alaska
Protapanteles jft02|CNCHZ461-09|01-Nov-2007|Canada.Ontario
Protapanteles jft02|ASCNC014-09|10-Jul-2007|Canada.Ontario
Protapanteles jft02|CNCHX172-09||Canada.Ontario
Protapanteles jft02|CNCHZ459-09|01-Sep-2007|Canada.Ontario
Protapanteles jft02|ASCNC015-09|10-Jul-2007|Canada.Ontario
Protapanteles jft02|ASWAV872-08|02-Aug-2007|Canada.Manitoba
Protapanteles jft02|ASWAV855-08|12-Jul-2007|Canada.Manitoba
Protapanteles jft02|ASWAX952-08|17-Jul-2007|Canada.Manitoba
Protapanteles jft02|ASWAV857-08|12-Jul-2007|Canada.Manitoba
Protapanteles jft02|ASWAV856-08|12-Jul-2007|Canada.Manitoba
Protapanteles jft02|ASWAV866-08|02-Aug-2007|Canada.Manitoba
Protapanteles jft02|CNCHZ465-09|30-Jul-2007|Canada.Ontario
Protapanteles jft02|ASCNC016-09|15-Jun-2007|Canada.Ontario
Protapanteles jft02|CNCHZ122-09|09-Jul-1954|Canada.Quebec
Pholetesor salicifoliellae|CNCHZ545-09|29-May-2008|Canada.Ontario
Pholetesor salicifoliellae|CNCHZ533-09|29-May-2008|Canada.Ontario
Pholetesor salicifoliellae|CNCHZ552-09|29-May-2008|Canada.Ontario
Pholetesor salicifoliellae|CNCHZ610-09|29-May-2008|Canada.Ontario
Pholetesor salicifoliellae|CNCHZ615-09|29-May-2008|Canada.Ontario
Pholetesor salicifoliellae|ASWAX941-08|10-Aug-2007|Canada.Manitoba
Pholetesor salicifoliellae|ASWAX948-08|10-Aug-2007|Canada.Manitoba
Pholetesor salicifoliellae|ASWAV865-08|12-Jul-2007|Canada.Manitoba
Pholetesor salicifoliellae|ASWAV892-08|11-Aug-2007|Canada.Manitoba
Pholetesor salicifoliellae|ASWAT782-08|07-Jul-2007|Canada.Manitoba
Pholetesor salicifoliellae|ASWAX956-08|17-Jul-2007|Canada.Manitoba
Pholetesor salicifoliellae|ASWAX949-08|10-Aug-2007|Canada.Manitoba
Pholetesor salicifoliellae|ASWAY028-08|05-Jul-2007|Canada.Manitoba
Pholetesor salicifoliellae|ASWAX997-08|07-Aug-2007|Canada.Manitoba
Pholetesor salicifoliellae|ASWAX942-08|10-Aug-2007|Canada.Manitoba
Pholetesor salicifoliellae|ASWAT768-08|07-Jul-2007|Canada.Manitoba
Pholetesor salicifoliellae|ASWAX982-08|23-Jul-2007|Canada.Manitoba
Pholetesor salicifoliellae|ASWAX944-08|10-Aug-2007|Canada.Manitoba
Pholetesor salicifoliellae|ASWAT770-08|07-Jul-2007|Canada.Manitoba
Pholetesor salicifoliellae|ASWAV903-08|11-Aug-2007|Canada.Manitoba
Pholetesor salicifoliellae|ASWAX947-08|10-Aug-2007|Canada.Manitoba
Pholetesor salicifoliellae|ASWAX954-08|17-Jul-2007|Canada.Manitoba
Pholetesor salicifoliellae|DSWAS928-07|08-Jul-2006|Canada.Yukon Territory
Pholetesor salicifoliellae|DSWAS963-07|06-Jul-2006|Canada.Yukon Territory
Pholetesor ornigis|ASWA610-08|02-Jun-2007|Canada.Saskatchewan
Pholetesor ornigis|CNCHZ614-09|29-May-2008|Canada.Ontario
Pholetesor ornigis|CNCHZ623-09|29-May-2008|Canada.Ontario
Pholetesor ornigis|ASWAX966-08|23-Jul-2007|Canada.Manitoba
Pholetesor ornigis|ASWA609-08|24-May-2007|Canada.Quebec
Pholetesor ornigis|ASWA611-08|02-Jun-2007|Canada.Saskatchewan
Pholetesor ornigis|ASWAT772-08|07-Jul-2007|Canada.Manitoba
Pholetesor ornigis|ASWA1027-08|17-Jul-2007|Canada.Manitoba
Pholetesor ornigis|ASWAT776-08|07-Jul-2007|Canada.Manitoba
Pholetesor ornigis|ASWAT769-08|07-Jul-2007|Canada.Manitoba
Pholetesor ornigis|ASWAT781-08|07-Jul-2007|Canada.Manitoba
Pholetesor ornigis|ASWA1026-08|17-Jul-2007|Canada.Manitoba
Pholetesor ornigis|ASWAT777-08|07-Jul-2007|Canada.Manitoba
Pholetesor ornigis|ASWAT774-08|07-Jul-2007|Canada.Manitoba
Pholetesor ornigis|ASWAT779-08|07-Jul-2007|Canada.Manitoba
Pholetesor ornigis|ASWAY029-08|05-Jul-2007|Canada.Manitoba
Pholetesor ornigis|ASWAT773-08|07-Jul-2007|Canada.Manitoba
Pholetesor ornigis|ASWAV967-08|16-Jul-2007|Canada.Manitoba
Pholetesor ornigis|ASWAT775-08|07-Jul-2007|Canada.Manitoba
Pholetesor ornigis|ASWAT767-08|07-Jul-2007|Canada.Manitoba
Pholetesor ornigis|ASWAV868-08|02-Aug-2007|Canada.Manitoba
Pholetesor ornigis|ASWAY695-08|07-Jul-2007|Canada.Manitoba
Pholetesor ornigis|CNCHY338-07|05-Jul-2006|Canada.Yukon Territory
Pholetesor ornigis|DSWAS955-07|06-Jul-2006|Canada.Yukon Territory
Pholetesor ornigis|CNCHZ093-09|25-Jun-1955|Canada.Quebec
Pholetesor bedelliae|DSWAS762-07|19-Aug-2006|Canada.Manitoba
Pholetesor bedelliae|DSWAS761-07|19-Aug-2006|Canada.Manitoba
Pholetesor bedelliae|DSWAS943-07|08-Jul-2006|Canada.Yukon Territory
Pholetesor bedelliae|DSWAS940-07|08-Jul-2006|Canada.Yukon Territory
Pholetesor bedelliae|DSWAS924-07|15-Jul-2006|Canada.Yukon Territory
Pholetesor bedelliae|DSWAS863-07|17-Aug-2006|Canada.Manitoba
Pholetesor bedelliae|ASWA606-08|04-Jun-2007|Canada.Saskatchewan
Pholetesor bedelliae|ASWA608-08|04-Jun-2007|Canada.Saskatchewan
Pholetesor bedelliae|CNCHY059-07|10-Jul-2006|Canada.Yukon Territory
Pholetesor bedelliae|DSWAS929-07|05-Jul-2006|Canada.Yukon Territory
Pholetesor bedelliae|CNCHZ468-09|30-May-2007|Canada.Ontario
Pholetesor bedelliae|CNCHZ627-09|29-May-2008|Canada.Ontario
Pholetesor bedelliae|DSWAS958-07|08-Jul-2006|Canada.Yukon Territory
Pholetesor bedelliae|DSWAS902-07|15-Jul-2006|Canada.Yukon Territory
Pholetesor bedelliae|DSWAS905-07|15-Jul-2006|Canada.Yukon Territory
Pholetesor bedelliae|DSWAS906-07|15-Jul-2006|Canada.Yukon Territory
Pholetesor bedelliae|DSWAS907-07|15-Jul-2006|Canada.Yukon Territory
Pholetesor bedelliae|DSWAS909-07|08-Jul-2006|Canada.Yukon Territory
Pholetesor bedelliae|DSWAS911-07|08-Jul-2006|Canada.Yukon Territory
Pholetesor bedelliae|DSWAS912-07|08-Jul-2006|Canada.Yukon Territory
Pholetesor bedelliae|DSWAS913-07|08-Jul-2006|Canada.Yukon Territory
Pholetesor bedelliae|DSWAS914-07|08-Jul-2006|Canada.Yukon Territory
Pholetesor bedelliae|DSWAS919-07|15-Jul-2006|Canada.Yukon Territory
Pholetesor bedelliae|DSWAS921-07|15-Jul-2006|Canada.Yukon Territory
Pholetesor bedelliae|DSWAS922-07|15-Jul-2006|Canada.Yukon Territory
Pholetesor bedelliae|DSWAS926-07|15-Jul-2006|Canada.Yukon Territory
Pholetesor bedelliae|DSWAS939-07|08-Jul-2006|Canada.Yukon Territory
Pholetesor bedelliae|DSWAS941-07|08-Jul-2006|Canada.Yukon Territory
Pholetesor bedelliae|DSWAS942-07|08-Jul-2006|Canada.Yukon Territory
Pholetesor bedelliae|DSWAS944-07|08-Jul-2006|Canada.Yukon Territory
Pholetesor bedelliae|DSWAS945-07|08-Jul-2006|Canada.Yukon Territory

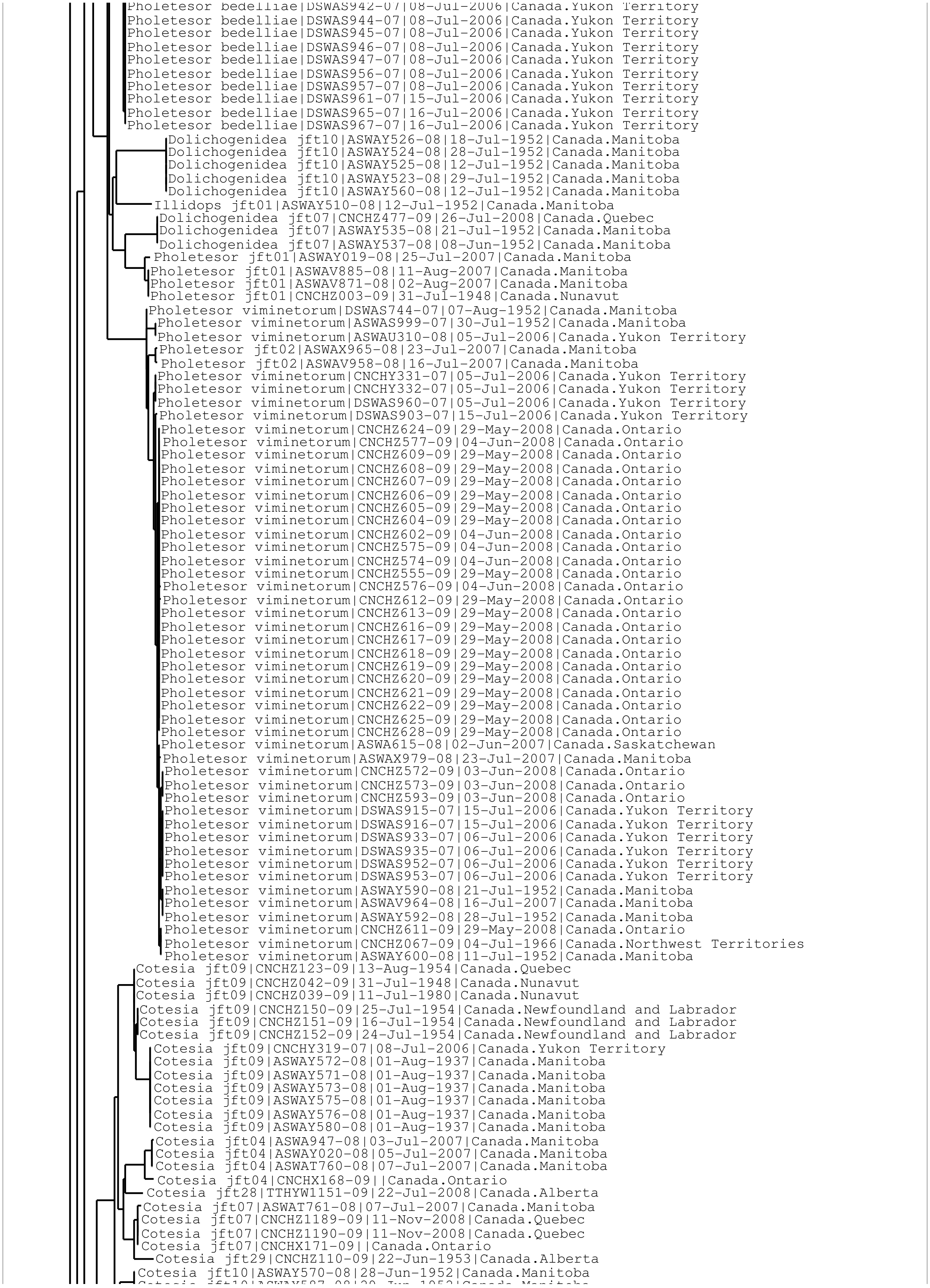
Pholetesor bedelliae|DSWAS942-07|08-Jul-2006|Canada.Yukon Territory
Pholetesor bedelliae|DSWAS944-07|08-Jul-2006|Canada.Yukon Territory
Pholetesor bedelliae|DSWAS945-07|08-Jul-2006|Canada.Yukon Territory
Pholetesor bedelliae|DSWAS946-07|08-Jul-2006|Canada.Yukon Territory
Pholetesor bedelliae|DSWAS947-07|08-Jul-2006|Canada.Yukon Territory
Pholetesor bedelliae|DSWAS956-07|08-Jul-2006|Canada.Yukon Territory
Pholetesor bedelliae|DSWAS957-07|08-Jul-2006|Canada.Yukon Territory
Pholetesor bedelliae|DSWAS961-07|15-Jul-2006|Canada.Yukon Territory
Pholetesor bedelliae|DSWAS965-07|16-Jul-2006|Canada.Yukon Territory
Pholetesor bedelliae|DSWAS967-07|16-Jul-2006|Canada.Yukon Territory
Dolichogenidea jft10|ASWAY526-08|18-Jul-1952|Canada.Manitoba
Dolichogenidea jft10|ASWAY524-08|28-Jul-1952|Canada.Manitoba
Dolichogenidea jft10|ASWAY525-08|12-Jul-1952|Canada.Manitoba
Dolichogenidea jft10|ASWAY523-08|29-Jul-1952|Canada.Manitoba
Dolichogenidea jft10|ASWAY560-08|12-Jul-1952|Canada.Manitoba
Illidops jft01|ASWAY510-08|12-Jul-1952|Canada.Manitoba
Dolichogenidea jft07|CNCHZ477-09|26-Jul-2008|Canada.Quebec
Dolichogenidea jft07|ASWAY535-08|21-Jul-1952|Canada.Manitoba
Dolichogenidea jft07|ASWAY537-08|08-Jun-1952|Canada.Manitoba
Pholetesor jft01|ASWAY019-08|25-Jul-2007|Canada.Manitoba
Pholetesor jft01|ASWAV885-08|11-Aug-2007|Canada.Manitoba
Pholetesor jft01|ASWAV871-08|02-Aug-2007|Canada.Manitoba
Pholetesor jft01|CNCHZ003-09|31-Jul-1948|Canada.Nunavut
Pholetesor viminetorum|DSWAS744-07|07-Aug-1952|Canada.Manitoba
Pholetesor viminetorum|ASWAS999-07|30-Jul-1952|Canada.Manitoba
Pholetesor viminetorum|ASWAU310-08|05-Jul-2006|Canada.Yukon Territory
Pholetesor jft02|ASWAX965-08|23-Jul-2007|Canada.Manitoba
Pholetesor jft02|ASWAV958-08|16-Jul-2007|Canada.Manitoba
Pholetesor viminetorum|CNCHY331-07|05-Jul-2006|Canada.Yukon Territory
Pholetesor viminetorum|CNCHY332-07|05-Jul-2006|Canada.Yukon Territory
Pholetesor viminetorum|DSWAS960-07|05-Jul-2006|Canada.Yukon Territory
Pholetesor viminetorum|DSWAS903-07|15-Jul-2006|Canada.Yukon Territory
Pholetesor viminetorum|CNCHZ624-09|29-May-2008|Canada.Ontario
Pholetesor viminetorum|CNCHZ577-09|04-Jun-2008|Canada.Ontario
Pholetesor viminetorum|CNCHZ609-09|29-May-2008|Canada.Ontario
Pholetesor viminetorum|CNCHZ608-09|29-May-2008|Canada.Ontario
Pholetesor viminetorum|CNCHZ607-09|29-May-2008|Canada.Ontario
Pholetesor viminetorum|CNCHZ606-09|29-May-2008|Canada.Ontario
Pholetesor viminetorum|CNCHZ605-09|29-May-2008|Canada.Ontario
Pholetesor viminetorum|CNCHZ604-09|29-May-2008|Canada.Ontario
Pholetesor viminetorum|CNCHZ602-09|04-Jun-2008|Canada.Ontario
Pholetesor viminetorum|CNCHZ575-09|04-Jun-2008|Canada.Ontario
Pholetesor viminetorum|CNCHZ574-09|04-Jun-2008|Canada.Ontario
Pholetesor viminetorum|CNCHZ555-09|29-May-2008|Canada.Ontario
Pholetesor viminetorum|CNCHZ576-09|04-Jun-2008|Canada.Ontario
Pholetesor viminetorum|CNCHZ612-09|29-May-2008|Canada.Ontario
Pholetesor viminetorum|CNCHZ613-09|29-May-2008|Canada.Ontario
Pholetesor viminetorum|CNCHZ616-09|29-May-2008|Canada.Ontario
Pholetesor viminetorum|CNCHZ617-09|29-May-2008|Canada.Ontario
Pholetesor viminetorum|CNCHZ618-09|29-May-2008|Canada.Ontario
Pholetesor viminetorum|CNCHZ619-09|29-May-2008|Canada.Ontario
Pholetesor viminetorum|CNCHZ620-09|29-May-2008|Canada.Ontario
Pholetesor viminetorum|CNCHZ621-09|29-May-2008|Canada.Ontario
Pholetesor viminetorum|CNCHZ622-09|29-May-2008|Canada.Ontario
Pholetesor viminetorum|CNCHZ625-09|29-May-2008|Canada.Ontario
Pholetesor viminetorum|CNCHZ628-09|29-May-2008|Canada.Ontario
Pholetesor viminetorum|ASWA615-08|02-Jun-2007|Canada.Saskatchewan
Pholetesor viminetorum|ASWAX979-08|23-Jul-2007|Canada.Manitoba
Pholetesor viminetorum|CNCHZ572-09|03-Jun-2008|Canada.Ontario
Pholetesor viminetorum|CNCHZ573-09|03-Jun-2008|Canada.Ontario
Pholetesor viminetorum|CNCHZ593-09|03-Jun-2008|Canada.Ontario
Pholetesor viminetorum|DSWAS915-07|15-Jul-2006|Canada.Yukon Territory
Pholetesor viminetorum|DSWAS916-07|15-Jul-2006|Canada.Yukon Territory
Pholetesor viminetorum|DSWAS933-07|06-Jul-2006|Canada.Yukon Territory
Pholetesor viminetorum|DSWAS935-07|06-Jul-2006|Canada.Yukon Territory
Pholetesor viminetorum|DSWAS952-07|06-Jul-2006|Canada.Yukon Territory
Pholetesor viminetorum|DSWAS953-07|06-Jul-2006|Canada.Yukon Territory
Pholetesor viminetorum|ASWAY590-08|21-Jul-1952|Canada.Manitoba
Pholetesor viminetorum|ASWAV964-08|16-Jul-2007|Canada.Manitoba
Pholetesor viminetorum|ASWAY592-08|28-Jul-1952|Canada.Manitoba
Pholetesor viminetorum|CNCHZ611-09|29-May-2008|Canada.Ontario
Pholetesor viminetorum|CNCHZ067-09|04-Jul-1966|Canada.Northwest Territories
Pholetesor viminetorum|ASWAY600-08|11-Jul-1952|Canada.Manitoba
Cotesia jft09|CNCHZ123-09|13-Aug-1954|Canada.Quebec
Cotesia jft09|CNCHZ042-09|31-Jul-1948|Canada.Nunavut
Cotesia jft09|CNCHZ039-09|11-Jul-1980|Canada.Nunavut
Cotesia jft09|CNCHZ150-09|25-Jul-1954|Canada.Newfoundland and Labrador
Cotesia jft09|CNCHZ151-09|16-Jul-1954|Canada.Newfoundland and Labrador
Cotesia jft09|CNCHZ152-09|24-Jul-1954|Canada.Newfoundland and Labrador
Cotesia jft09|CNCHY319-07|08-Jul-2006|Canada.Yukon Territory
Cotesia jft09|ASWAY572-08|01-Aug-1937|Canada.Manitoba
Cotesia jft09|ASWAY571-08|01-Aug-1937|Canada.Manitoba
Cotesia jft09|ASWAY573-08|01-Aug-1937|Canada.Manitoba
Cotesia jft09|ASWAY575-08|01-Aug-1937|Canada.Manitoba
Cotesia jft09|ASWAY576-08|01-Aug-1937|Canada.Manitoba
Cotesia jft09|ASWAY580-08|01-Aug-1937|Canada.Manitoba
Cotesia jft04|ASWA947-08|03-Jul-2007|Canada.Manitoba
Cotesia jft04|ASWAY020-08|05-Jul-2007|Canada.Manitoba
Cotesia jft04|ASWAT760-08|07-Jul-2007|Canada.Manitoba
Cotesia jft04|CNCHX168-09||Canada.Ontario
Cotesia jft28|TTHYW1151-09|22-Jul-2008|Canada.Alberta
Cotesia jft07|ASWAT761-08|07-Jul-2007|Canada.Manitoba
Cotesia jft07|CNCHZ1189-09|11-Nov-2008|Canada.Quebec
Cotesia jft07|CNCHZ1190-09|11-Nov-2008|Canada.Quebec
Cotesia jft07|CNCHX171-09||Canada.Ontario
Cotesia jft29|CNCHZ110-09|22-Jun-1953|Canada.Alberta
Cotesia jft10|ASWAY570-08|28-Jun-1952|Canada.Manitoba

Cotesia jft07|CNCHX171-09||Canada.Ontario
Cotesia jft29|CNCHZ110-09|22-Jun-1953|Canada.Alberta
Cotesia jft10|ASWAY570-08|28-Jun-1952|Canada.Manitoba
Cotesia jft10|ASWAY587-08|29-Jun-1952|Canada.Manitoba
Cotesia jft10|ASWAY597-08|11-Jul-1952|Canada.Manitoba
Cotesia halli|CNCHZ043-09|01-Jul-1966|Greenland
Cotesia halli|CNCHZ048-09|19-Jul-1963|Canada.Nunavut
Cotesia jft52|CNCHZ047-09|29-Jul-1968|Canada.Northwest Territories
Glyptapanteles jft08|ASWA948-08|03-Jul-2007|Canada.Manitoba
Cotesia jft23|CNCHZ041-09|28-Jun-1950|Canada.Nunavut
Cotesia jft23|CNCHZ070-09|05-Jul-1956|Canada.Nunavut
Cotesia jft23|CNCHZ071-09|05-Jul-1956|Canada.Nunavut
Cotesia jft22|CNCHZ035-09|28-Jul-1968|Canada.Northwest Territories
Dolichogenidea jft09|CNCHZ483-09|26-Jul-2008|Canada.Quebec
Dolichogenidea jft09|ASWAY538-08|27-Aug-1950|Canada.Manitoba
Dolichogenidea jft09|CNCHZ489-09|26-Jul-2008|Canada.Quebec
Dolichogenidea jft09|CNCHZ488-09|26-Jul-2008|Canada.Quebec
Dolichogenidea jft09|CNCHZ487-09|26-Jul-2008|Canada.Quebec
Dolichogenidea jft09|CNCHZ486-09|26-Jul-2008|Canada.Quebec
Dolichogenidea jft09|CNCHZ484-09|26-Jul-2008|Canada.Quebec
Dolichogenidea jft09|CNCHZ482-09|26-Jul-2008|Canada.Quebec
Dolichogenidea jft09|CNCHZ485-09|26-Jul-2008|Canada.Quebec
Dolichogenidea jft09|ASWAY599-08|24-Jul-1952|Canada.Manitoba
Dolichogenidea jft06|ASWAV916-08|08-Aug-2007|Canada.Manitoba
Dolichogenidea jft06|ASWAV947-08|08-Aug-2007|Canada.Manitoba
Dolichogenidea jft06|CNCHY318-07|13-Jul-2006|Canada.Yukon Territory
Dolichogenidea jft06|CNCHY368-07|16-Jul-2006|Canada.Yukon Territory
Dolichogenidea jft06|CNCHY374-07|16-Jul-2006|Canada.Yukon Territory
Dolichogenidea jft06|CNCHZ134-09|17-Aug-1959|Canada.Quebec
Dolichogenidea jft06|ASWAU338-08|15-Jul-2006|Canada.Yukon Territory
Dolichogenidea jft06|ASWAU345-08|15-Jul-2006|Canada.Yukon Territory
Dolichogenidea jft08|ASWAY527-08|05-Aug-1952|Canada.Manitoba
Dolichogenidea jft08|ASWAY541-08|08-Jun-1952|Canada.Manitoba
Pholetesor jft11|CNCHZ056-09|17-Jul-1966|Canada.Nunavut
Dolichogenidea jft05|ASWAY546-08|15-Aug-2006|Canada.Manitoba
Dolichogenidea jft05|ASWAV930-08|08-Aug-2007|Canada.Manitoba
Dolichogenidea jft05|ASWAY547-08|15-Aug-2006|Canada.Manitoba
Dolichogenidea jft05|ASCNC021-09|09-Jul-2007|Canada.Ontario
Apanteles petrovae|CNCHZ263-09|26-Jun-2007|Canada.Ontario
Apanteles petrovae|CNCHX136-09||Canada.Ontario
Apanteles petrovae|CNCHX138-09||Canada.Ontario
Apanteles petrovae|CNCHZ209-09|30-May-2007|Canada.Ontario
Apanteles petrovae|CNCHX132-09||Canada.Ontario
Apanteles petrovae|CNCHX130-09||Canada.Ontario
Apanteles petrovae|ASWAV928-08|08-Aug-2007|Canada.Manitoba
Apanteles petrovae|CNCHX139-09||Canada.Ontario
Apanteles petrovae|CNCHZ118-09|11-Jul-1951|United States.Alaska
Protapanteles jft01|ASWAU194-08|22-Jun-2006|Canada.Alberta
Protapanteles jft01|CNCHY270-07|15-Jul-2006|Canada.Yukon Territory
Protapanteles jft01|ASWAY620-08|29-Jun-1952|Canada.Manitoba
Microgaster jft10|DSWAS857-07|17-Aug-2006|Canada.Manitoba
Microgaster jft10|DSWAS858-07|17-Aug-2006|Canada.Manitoba
Microgaster jft10|CNCHY064-07|10-Jul-2006|Canada.Yukon Territory
Microgaster jft10|ASWAU288-08|10-Jul-2006|Canada.Yukon Territory
Microgaster jft10|ASWA619-08|02-Jun-2007|Canada.Saskatchewan
Microgaster jft10|ASWAX957-08|17-Jul-2007|Canada.Manitoba
Microgaster jft10|ASWAU293-08|19-Jul-2006|Canada.Yukon Territory
Microgaster jft08|ASWAT757-08|07-Jul-2007|Canada.Manitoba
Microgaster jft08|CNCHZ028-09|08-Jul-1968|Canada.Northwest Territories
Microgaster jft08|CNCHZ132-09|27-Jul-1951|United States.Alaska
Microgaster jft10|ASWAX988-08|07-Aug-2007|Canada.Manitoba
Microgaster jft10|ASWAX992-08|07-Aug-2007|Canada.Manitoba
Microgaster jft10|ASWAY003-08|07-Aug-2007|Canada.Manitoba
Microgaster jft10|CNCHX052-09||Canada.Ontario
Microgaster jft10|CNCHX057-09||Canada.Ontario
Microgaster jft02|CNCHX056-09||Canada.Ontario
Microgaster jft10|CNCHX053-09||Canada.Ontario
Microgaster jft10|CNCHX055-09||Canada.Ontario
Microgaster jft10|CNCHX058-09||Canada.Ontario
Microgaster jft10|ASWAY030-08|05-Jul-2007|Canada.Manitoba
Microgaster jft10|CNCHY063-07|10-Jul-2006|Canada.Yukon Territory
Microgaster jft10|CNCHY210-07|06-Jul-2006|Canada.Yukon Territory
Microgaster jft10|CNCHY215-07|06-Jul-2006|Canada.Yukon Territory
Microgaster jft08|ASWAY567-08|11-Jul-1952|Canada.Manitoba
Microgaster jft10|CNCHZ139-09|26-Jul-1956|Canada.Quebec
Microgaster jft01|DSWAS859-07|29-Jun-1952|Canada.Manitoba
Microgaster jft06|ASWAY021-08|05-Jul-2007|Canada.Manitoba
Microgaster jft06|ASWAV932-08|08-Aug-2007|Canada.Manitoba
Microgaster jft06|ASWA943-08|03-Jul-2007|Canada.Manitoba
Microgaster jft06|ASWA946-08|03-Jul-2007|Canada.Manitoba
Microgaster jft06|ASWAX953-08|17-Jul-2007|Canada.Manitoba
Microgaster jft07|ASWAY032-08|05-Jul-2007|Canada.Manitoba
Microgaster canadensis|ASWAV929-08|08-Aug-2007|Canada.Manitoba
Microgaster canadensis|TTHYW990-09|19-Jul-2008|Canada.Saskatchewan
Microgaster jft10|CNCHZ160-09|28-Jun-1952|United States.Alaska
Microgaster deductor|ASWAY669-08|12-Jul-1952|Canada.Manitoba
Microgaster deductor|ASWAY668-08|12-Jul-1952|Canada.Manitoba
Microgaster deductor|ASWAY663-08|07-Jul-1952|Canada.Manitoba
Microgaster deductor|ASWAY661-08|12-Jul-1952|Canada.Manitoba
Microgaster deductor|ASWAY656-08|29-Jun-1952|Canada.Manitoba
Microgaster deductor|ASWAY655-08|12-Jul-1952|Canada.Manitoba
Microgaster deductor|ASWAY690-08|23-Jul-1952|Canada.Manitoba
Microgaster deductor|ASWAY689-08|03-Aug-1952|Canada.Manitoba
Microgaster deductor|ASWAY687-08|12-Jul-1952|Canada.Manitoba
Microgaster deductor|ASWAY684-08|03-Aug-1952|Canada.Manitoba
Microgaster deductor|ASWAY682-08|12-Jul-1952|Canada.Manitoba
Microgaster deductor|ASWAY678-08|12-Jul-1952|Canada.Manitoba
Microgaster deductor|ASWAY674-08|28-Jul-1952|Canada.Manitoba

Microgaster deductor|ASWAY682-08|12-Jul-1952|Canada.Manitoba
Microgaster deductor|ASWAY678-08|12-Jul-1952|Canada.Manitoba
Microgaster deductor|ASWAY674-08|28-Jul-1952|Canada.Manitoba
Microgaster deductor|ASWAY683-08|18-Jul-1952|Canada.Manitoba
Microgaster deductor|ASWAY680-08|03-Aug-1952|Canada.Manitoba
Microgaster deductor|ASWAY677-08|12-Jul-1952|Canada.Manitoba
Microgaster deductor|ASWAY685-08|29-Jul-1952|Canada.Manitoba
Microgaster deductor|ASWAY670-08|12-Jul-1952|Canada.Manitoba
Microgaster deductor|ASWAY671-08|29-Jul-1952|Canada.Manitoba
Microgaster deductor|ASWAY589-08|18-Jul-1952|Canada.Manitoba
Microgaster deductor|ASWAY667-08|23-Jul-1952|Canada.Manitoba
Microgaster deductor|ASWAY662-08|12-Jul-1952|Canada.Manitoba
Microgaster deductor|ASWAY660-08|12-Jul-1952|Canada.Manitoba
Microgaster deductor|ASWAY658-08|12-Jul-1952|Canada.Manitoba
Microgaster deductor|ASWAY657-08|29-Jun-1952|Canada.Manitoba
Microgaster deductor|ASWAY691-08|28-Jul-1952|Canada.Manitoba
Microgaster deductor|ASWAY681-08|23-Jul-1952|Canada.Manitoba
Microgaster deductor|ASWAY679-08|28-Jul-1952|Canada.Manitoba
Microgaster deductor|ASWAY676-08|03-Aug-1952|Canada.Manitoba
Microgaster deductor|ASWAY686-08|28-Jul-1952|Canada.Manitoba
Microgaster deductor|ASWAY673-08|03-Aug-1952|Canada.Manitoba
Microgaster deductor|ASWAY664-08|23-Jul-1952|Canada.Manitoba
Microgaster deductor|ASWAY666-08|12-Jul-1952|Canada.Manitoba
Microgaster deductor|ASWAY672-08|23-Jul-1952|Canada.Manitoba
Microgaster jft05|ASWAU291-08|05-Jul-2006|Canada.Yukon Territory
Microgaster jft05|ASWAT756-08|07-Jul-2007|Canada.Manitoba
Microgaster jft05|ASWAT758-08|07-Jul-2007|Canada.Manitoba
Microgaster jft05|ASWA638-08|02-Jun-2007|Canada.Saskatchewan
Microgaster jft05|ASWA644-08|02-Jun-2007|Canada.Saskatchewan
Microgaster jft05|ASWA629-08|02-Jun-2007|Canada.Saskatchewan
Microgaster jft05|ASWA621-08|02-Jun-2007|Canada.Saskatchewan
Microgaster jft05|ASWA630-08|02-Jun-2007|Canada.Saskatchewan
Microgaster jft05|ASWA622-08|02-Jun-2007|Canada.Saskatchewan
Microgaster jft05|ASWA637-08|02-Jun-2007|Canada.Saskatchewan
Microgaster jft05|ASWA645-08|02-Jun-2007|Canada.Saskatchewan
Microgaster jft05|ASWA631-08|02-Jun-2007|Canada.Saskatchewan
Microgaster jft05|CNCHZ138-09|21-Jun-1948|Canada.Quebec
Microgaster jft03|ASWAU284-08|10-Jul-2006|Canada.Yukon Territory
Microgaster jft03|ASWAU365-08|06-Jul-2006|Canada.Yukon Territory
Microgaster jft03|ASWAU374-08|06-Jul-2006|Canada.Yukon Territory
Microgaster jft03|ASWA1071-08||Canada.Manitoba
Microgaster jft03|ASWAU368-08|06-Jul-2006|Canada.Yukon Territory
Microgaster jft03|ASWAU370-08|06-Jul-2006|Canada.Yukon Territory
Microgaster jft03|CNCHY193-07|06-Jul-2006|Canada.Yukon Territory
Microgaster jft03|ASWAU367-08|06-Jul-2006|Canada.Yukon Territory
Microgaster jft03|CNCHY189-07|08-Jul-2006|Canada.Yukon Territory
Microgaster jft03|CNCHY205-07|06-Jul-2006|Canada.Yukon Territory
Microgaster jft03|ASWAU373-08|06-Jul-2006|Canada.Yukon Territory
Microgaster jft03|CNCHY190-07|08-Jul-2006|Canada.Yukon Territory
Microgaster jft03|CNCHY208-07|08-Jul-2006|Canada.Yukon Territory
Microgaster jft03|CNCHY195-07|06-Jul-2006|Canada.Yukon Territory
Microgaster jft03|ASWAU357-08|10-Jul-2006|Canada.Yukon Territory
Microgaster jft03|CNCHY204-07|08-Jul-2006|Canada.Yukon Territory
Microgaster jft03|CNCHY216-07|14-Jul-2006|Canada.Yukon Territory
Microgaster jft03|CNCHY209-07|06-Jul-2006|Canada.Yukon Territory
Microgaster jft03|CNCHY206-07|06-Jul-2006|Canada.Yukon Territory
Microgaster jft03|CNCHY203-07|08-Jul-2006|Canada.Yukon Territory
Microgaster jft03|CNCHY194-07|06-Jul-2006|Canada.Yukon Territory
Microgaster jft03|ASWAU369-08|06-Jul-2006|Canada.Yukon Territory
Microgaster jft03|ASWAU354-08|10-Jul-2006|Canada.Yukon Territory
Microgaster jft03|CNCHY192-07|06-Jul-2006|Canada.Yukon Territory
Microgaster jft03|ASWAU294-08|10-Jul-2006|Canada.Yukon Territory
Microgaster jft03|ASWAU376-08|06-Jul-2006|Canada.Yukon Territory
Microgaster jft03|ASWAU295-08|10-Jul-2006|Canada.Yukon Territory
Microgaster jft03|CNCHY207-07|06-Jul-2006|Canada.Yukon Territory
Microgaster jft03|CNCHY202-07|08-Jul-2006|Canada.Yukon Territory
Microgaster jft03|CNCHY212-07|08-Jul-2006|Canada.Yukon Territory
Microgaster jft03|ASWAU375-08|06-Jul-2006|Canada.Yukon Territory
Microgaster jft03|ASWAU363-08|06-Jul-2006|Canada.Yukon Territory
Microgaster jft03|ASWAU283-08|10-Jul-2006|Canada.Yukon Territory
Microgaster jft03|ASWAU364-08|06-Jul-2006|Canada.Yukon Territory
Microgaster jft03|ASWAU371-08|06-Jul-2006|Canada.Yukon Territory
Microgaster jft03|ASWAU366-08|06-Jul-2006|Canada.Yukon Territory
Microgaster jft03|ASWAU358-08|10-Jul-2006|Canada.Yukon Territory
Microgaster jft03|CNCHY191-07|06-Jul-2006|Canada.Yukon Territory
Microgaster jft03|ASWAU372-08|06-Jul-2006|Canada.Yukon Territory
Microgaster jft03|ASWAU361-08|06-Jul-2006|Canada.Yukon Territory
Microgaster jft03|ASWAU285-08|10-Jul-2006|Canada.Yukon Territory
Microgaster jft09|CNCHY214-07|19-Jul-2006|Canada.Yukon Territory
Microgaster jft09|CNCHZ133-09|28-Jul-1951|United States.Alaska
Microgaster jft09|ASWAY565-08|21-Jun-1952|Canada.Manitoba
Microgaster jft13|CNCHZ106-09|08-Jun-1987|Canada.Quebec
Microgaster jft13|CNCHZ129-09|26-Jul-1951|United States.Alaska
Microgaster jft16|CNCHZ128-09|20-Jul-1952|United States.Alaska
Microgaster jft21|CNCHZ131-09|16-Jul-1952|United States.Alaska
Microgaster jft21|CNCHZ140-09|08-Jul-1953|Canada.Quebec
Sathon jft01|ASWAY696-08|17-Aug-2006|Canada.Manitoba
Sathon|CNCHZ095-09|23-Jul-1954|Canada.Quebec
Sathon|CNCHZ107-09|11-Aug-1954|Canada.Quebec
Illidops jft02|CNCHZ064-09|07-Jul-1958|Canada.Quebec
Illidops jft02|ASWAY005-08|25-Jun-2007|Canada.Manitoba
Illidops jft02|CNCHZ002-09|25-Jul-1965|Canada.Northwest Territories
Illidops jft02|CNCHZ008-09|13-Jul-1975|Canada.Northwest Territories
Illidops jft02|CNCHZ062-09|08-Aug-1950|Canada.Northwest Territories
Illidops jft02|CNCHZ068-09|18-Jul-1966|Canada.Nunavut
Illidops jft02|ASWAY508-08|25-Jun-1952|Canada.Manitoba
Apanteles jft02|ASWAX995-08|07-Aug-2007|Canada.Manitoba
[illegible]

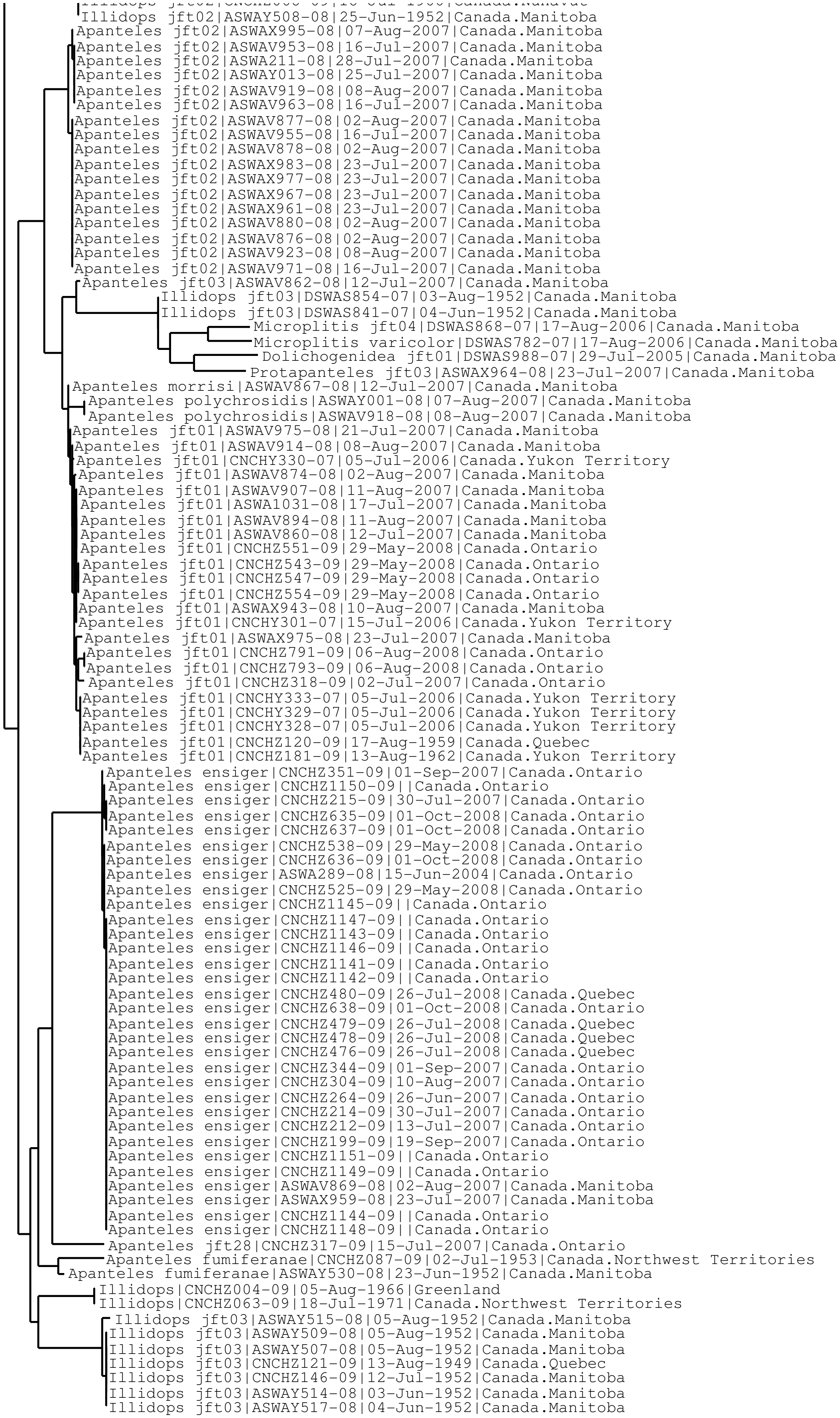
Illidops jft02|ASWAY508-08|25-Jun-1952|Canada.Manitoba
Apanteles jft02|ASWAX995-08|07-Aug-2007|Canada.Manitoba
Apanteles jft02|ASWAV953-08|16-Jul-2007|Canada.Manitoba
Apanteles jft02|ASWA211-08|28-Jul-2007|Canada.Manitoba
Apanteles jft02|ASWAY013-08|25-Jul-2007|Canada.Manitoba
Apanteles jft02|ASWAV919-08|08-Aug-2007|Canada.Manitoba
Apanteles jft02|ASWAV963-08|16-Jul-2007|Canada.Manitoba
Apanteles jft02|ASWAV877-08|02-Aug-2007|Canada.Manitoba
Apanteles jft02|ASWAV955-08|16-Jul-2007|Canada.Manitoba
Apanteles jft02|ASWAV878-08|02-Aug-2007|Canada.Manitoba
Apanteles jft02|ASWAX983-08|23-Jul-2007|Canada.Manitoba
Apanteles jft02|ASWAX977-08|23-Jul-2007|Canada.Manitoba
Apanteles jft02|ASWAX967-08|23-Jul-2007|Canada.Manitoba
Apanteles jft02|ASWAX961-08|23-Jul-2007|Canada.Manitoba
Apanteles jft02|ASWAV880-08|02-Aug-2007|Canada.Manitoba
Apanteles jft02|ASWAV876-08|02-Aug-2007|Canada.Manitoba
Apanteles jft02|ASWAV923-08|08-Aug-2007|Canada.Manitoba
Apanteles jft02|ASWAV971-08|16-Jul-2007|Canada.Manitoba
Apanteles jft03|ASWAV862-08|12-Jul-2007|Canada.Manitoba
Illidops jft03|DSWAS854-07|03-Aug-1952|Canada.Manitoba
Illidops jft03|DSWAS841-07|04-Jun-1952|Canada.Manitoba
Microplitis jft04|DSWAS868-07|17-Aug-2006|Canada.Manitoba
Microplitis varicolor|DSWAS782-07|17-Aug-2006|Canada.Manitoba
Dolichogenidea jft01|DSWAS988-07|29-Jul-2005|Canada.Manitoba
Protapanteles jft03|ASWAX964-08|23-Jul-2007|Canada.Manitoba
Apanteles morrisi|ASWAV867-08|12-Jul-2007|Canada.Manitoba
Apanteles polychrosidis|ASWAY001-08|07-Aug-2007|Canada.Manitoba
Apanteles polychrosidis|ASWAV918-08|08-Aug-2007|Canada.Manitoba
Apanteles jft01|ASWAV975-08|21-Jul-2007|Canada.Manitoba
Apanteles jft01|ASWAV914-08|08-Aug-2007|Canada.Manitoba
Apanteles jft01|CNCHY330-07|05-Jul-2006|Canada.Yukon Territory
Apanteles jft01|ASWAV874-08|02-Aug-2007|Canada.Manitoba
Apanteles jft01|ASWAV907-08|11-Aug-2007|Canada.Manitoba
Apanteles jft01|ASWA1031-08|17-Jul-2007|Canada.Manitoba
Apanteles jft01|ASWAV894-08|11-Aug-2007|Canada.Manitoba
Apanteles jft01|ASWAV860-08|12-Jul-2007|Canada.Manitoba
Apanteles jft01|CNCHZ551-09|29-May-2008|Canada.Ontario
Apanteles jft01|CNCHZ543-09|29-May-2008|Canada.Ontario
Apanteles jft01|CNCHZ547-09|29-May-2008|Canada.Ontario
Apanteles jft01|CNCHZ554-09|29-May-2008|Canada.Ontario
Apanteles jft01|ASWAX943-08|10-Aug-2007|Canada.Manitoba
Apanteles jft01|CNCHY301-07|15-Jul-2006|Canada.Yukon Territory
Apanteles jft01|ASWAX975-08|23-Jul-2007|Canada.Manitoba
Apanteles jft01|CNCHZ791-09|06-Aug-2008|Canada.Ontario
Apanteles jft01|CNCHZ793-09|06-Aug-2008|Canada.Ontario
Apanteles jft01|CNCHZ318-09|02-Jul-2007|Canada.Ontario
Apanteles jft01|CNCHY333-07|05-Jul-2006|Canada.Yukon Territory
Apanteles jft01|CNCHY329-07|05-Jul-2006|Canada.Yukon Territory
Apanteles jft01|CNCHY328-07|05-Jul-2006|Canada.Yukon Territory
Apanteles jft01|CNCHZ120-09|17-Aug-1959|Canada.Quebec
Apanteles jft01|CNCHZ181-09|13-Aug-1962|Canada.Yukon Territory
Apanteles ensiger|CNCHZ351-09|01-Sep-2007|Canada.Ontario
Apanteles ensiger|CNCHZ1150-09||Canada.Ontario
Apanteles ensiger|CNCHZ215-09|30-Jul-2007|Canada.Ontario
Apanteles ensiger|CNCHZ635-09|01-Oct-2008|Canada.Ontario
Apanteles ensiger|CNCHZ637-09|01-Oct-2008|Canada.Ontario
Apanteles ensiger|CNCHZ538-09|29-May-2008|Canada.Ontario
Apanteles ensiger|CNCHZ636-09|01-Oct-2008|Canada.Ontario
Apanteles ensiger|ASWA289-08|15-Jun-2004|Canada.Ontario
Apanteles ensiger|CNCHZ525-09|29-May-2008|Canada.Ontario
Apanteles ensiger|CNCHZ1145-09||Canada.Ontario
Apanteles ensiger|CNCHZ1147-09||Canada.Ontario
Apanteles ensiger|CNCHZ1143-09||Canada.Ontario
Apanteles ensiger|CNCHZ1146-09||Canada.Ontario
Apanteles ensiger|CNCHZ1141-09||Canada.Ontario
Apanteles ensiger|CNCHZ1142-09||Canada.Ontario
Apanteles ensiger|CNCHZ480-09|26-Jul-2008|Canada.Quebec
Apanteles ensiger|CNCHZ638-09|01-Oct-2008|Canada.Ontario
Apanteles ensiger|CNCHZ479-09|26-Jul-2008|Canada.Quebec
Apanteles ensiger|CNCHZ478-09|26-Jul-2008|Canada.Quebec
Apanteles ensiger|CNCHZ476-09|26-Jul-2008|Canada.Quebec
Apanteles ensiger|CNCHZ344-09|01-Sep-2007|Canada.Ontario
Apanteles ensiger|CNCHZ304-09|10-Aug-2007|Canada.Ontario
Apanteles ensiger|CNCHZ264-09|26-Jun-2007|Canada.Ontario
Apanteles ensiger|CNCHZ214-09|30-Jul-2007|Canada.Ontario
Apanteles ensiger|CNCHZ212-09|13-Jul-2007|Canada.Ontario
Apanteles ensiger|CNCHZ199-09|19-Sep-2007|Canada.Ontario
Apanteles ensiger|CNCHZ1151-09||Canada.Ontario
Apanteles ensiger|CNCHZ1149-09||Canada.Ontario
Apanteles ensiger|ASWAV869-08|02-Aug-2007|Canada.Manitoba
Apanteles ensiger|ASWAX959-08|23-Jul-2007|Canada.Manitoba
Apanteles ensiger|CNCHZ1144-09||Canada.Ontario
Apanteles ensiger|CNCHZ1148-09||Canada.Ontario
Apanteles jft28|CNCHZ317-09|15-Jul-2007|Canada.Ontario
Apanteles fumiferanae|CNCHZ087-09|02-Jul-1953|Canada.Northwest Territories
Apanteles fumiferanae|ASWAY530-08|23-Jun-1952|Canada.Manitoba
Illidops|CNCHZ004-09|05-Aug-1966|Greenland
Illidops|CNCHZ063-09|18-Jul-1971|Canada.Northwest Territories
Illidops jft03|ASWAY515-08|05-Aug-1952|Canada.Manitoba
Illidops jft03|ASWAY509-08|05-Aug-1952|Canada.Manitoba
Illidops jft03|ASWAY507-08|05-Aug-1952|Canada.Manitoba
Illidops jft03|CNCHZ121-09|13-Aug-1949|Canada.Quebec
Illidops jft03|CNCHZ146-09|12-Jul-1952|Canada.Manitoba
Illidops jft03|ASWAY514-08|03-Jun-1952|Canada.Manitoba
Illidops jft03|ASWAY517-08|04-Jun-1952|Canada.Manitoba
