# Supplementary material for: A Poorly Known High-Latitude Parasitoid Wasp Community: Unexpected Diversity and Dramatic Changes through Time
Source: PLoS One. 2011 Aug 29;6(8):e23719. doi: 10.1371/journal.pone.0023719 (PMC3163582; doi:10.1371/journal.pone.0023719)
Supplement: Table S1 — Species of Microgastrinae from Churchill, Manitoba, Canada. Data comprise the first half of the 20th century (1930–1950's), referred as “Hist” in the heading of the second column; and recent collecting (2005–2007), referred as “Cont” in the heading of the third column. Species presence (+) or absence (−) is shown for Old and New data. Collecting dates were grouped in first and second half of every month sampled, indicated in the table headings with Arabic numbers (1 or 2) followed by the corresponding month (June = Jun, July = Jul, August = Aug). New records for Manitoba (*) or the Nearctic (**) are marked in the table. N/B marks the species with no barcodes available from this study. (PDF) [file pone.0023719.s004.pdf]

Table S1: Species of Microgastrinae from Churchill, Manitoba, Canada. Data comprise the first half of the 20th century (1930-1950's), referred as "Hist" in the heading of the second column; and recent collecting (2005-2007), referred as "Cont" in the heading of the third column. Species presence (+) or absence (-) is shown for Old and New data. Collecting dates were grouped in first and second half of every month sampled, indicated in the table headings with Arabic numbers (1 or 2) followed by the corresponding month (June=Jun, July=Jul, August=Aug). New records for Manitoba (*) or the Nearctic (**) are marked in the table. N/B marks the species with no barcodes available from this study.

ON=Ontario, QC=Quebec, NWT=Northwest Territories, SK=Saskatchewan, AB=Alberta, NU=Nunavat, BC=British Columbia, NFLD=Newfoundland, Greenland=GR

| | Hist | Cont | Affinities | 01-Jun | 02-Jun | 01-Jul | 02-Jul | 01-Aug | 02-Aug | Tot |
|---|---|---|---|---|---|---|---|---|---|---|
| *Apanteles ensiger* | - | + | ON, QC | 0 | 0 | 0 | 1 | 1 | 1 | 3 |
| *Apanteles fumiferanae* * | + | + | Only Churchill | 0 | 1 | 0 | 0 | 0 | 1 | 2 |
| *Apanteles morrisi* N/B | - | + | | 0 | 0 | 1 | 0 | 0 | 0 | 1 |
| *Apanteles petrovae* * | - | + | ON, QC | 0 | 0 | 0 | 0 | 1 | 0 | 1 |
| *Apanteles polychrosidis* * | - | + | Only Churchill | 0 | 0 | 0 | 0 | 2 | 4 | 6 |
| *Apanteles* jft01 | - | + | ON, QC, YK | 0 | 7 | 1 | 2 | 6 | 2 | 18 |
| *Apanteles* jft02 | - | + | Only Churchill | 0 | 1 | 0 | 28 | 7 | 1 | 37 |
| *Apanteles* jft03 | - | + | Only Churchill | 0 | 0 | 2 | 0 | 0 | 0 | 2 |
| *Cotesia xylina* | + | + | YK, ON, AB, NWT | 1 | 1 | 2 | 0 | 7 | 0 | 11 |
| *Cotesia yakutatensis* * | + | + | Only Churchill | 0 | 0 | 1 | 0 | 1 | 4 | 6 |
| *Cotesia* jft01 | - | + | Only Churchill | 0 | 0 | 0 | 0 | 0 | 1 | 1 |
| *Cotesia* jft02 | - | + | ON, AB, SK | 0 | 0 | 0 | 0 | 0 | 1 | 1 |
| *Cotesia* jft03 | - | + | ON | 0 | 0 | 0 | 1 | 0 | 0 | 1 |
| *Cotesia* jft04 | - | + | ON | 0 | 0 | 4 | 0 | 0 | 0 | 4 |
| *Cotesia* jft05 | - | + | ON | 0 | 0 | 0 | 6 | 3 | 1 | 10 |
| *Cotesia* jft06 | - | + | Only Churchill | 0 | 1 | 0 | 0 | 6 | 1 | 8 |
| *Cotesia* jft07 | - | + | QC, ON | 0 | 2 | 1 | 0 | 0 | 0 | 3 |
| *Cotesia* jft08 | + | + | NU | 0 | 2 | 7 | 5 | 0 | 0 | 14 |
| *Cotesia* jft09 | + | - | QC, NWT, NU, NFLD | 0 | 0 | 0 | 0 | 10 | 0 | 10 |
| *Cotesia* jft10 | + | - | Only Churchill | 0 | 2 | 1 | 0 | 0 | 0 | 3 |
| *Diolcogaster* jft01 | - | + | Only Churchill | 0 | 0 | 0 | 0 | 0 | 1 | 1 |
| *Diolcogaster* jft02 | - | + | Only Churchill | 0 | 0 | 0 | 1 | 0 | 0 | 1 |
| *Dolichogenidea* jft01 | + | + | YK | 3 | 1 | 1 | 4 | 6 | 0 | 15 |
| *Dolichogenidea* jft02 | + | + | YK, ON, QC, NWT, AL | 0 | 2 | 1 | 1 | 9 | 1 | 14 |
| *Dolichogenidea* jft03 | + | + | YK, AL, NWT, BC, NU | 0 | 1 | 2 | 0 | 11 | 4 | 18 |
| *Dolichogenidea* jft04 | - | + | NWT | 0 | 0 | 2 | 0 | 1 | 0 | 3 |
| *Dolichogenidea* jft05 | - | + | ON | 0 | 0 | 1 | 0 | 1 | 2 | 4 |
| *Dolichogenidea* jft06 | - | + | YK, QC | 0 | 0 | 0 | 0 | 2 | 0 | 2 |
| *Dolichogenidea* jft07 | + | - | Only Churchill | 3 | 0 | 0 | 4 | 0 | 0 | 7 |
| *Dolichogenidea* jft08 | + | - | Only Churchill | 0 | 0 | 0 | 1 | 2 | 0 | 3 |

Table S1: Species of Microgastrinae from Churchill, Manitoba, Canada. Data comprise the first half of the 20th century (1930-1950's), referred as "Hist" in the heading of the second column; and recent collecting (2005-2007), referred as "Cont" in the heading of the third column. Species presence (+) or absence (-) is shown for Old and New data. Collecting dates were grouped in first and second half of every month sampled, indicated in the table headings with Arabic numbers (1 or 2) followed by the corresponding month (June=Jun, July=Jul, August=Aug). New records for Manitoba (*) or the Nearctic (**) are marked in the table. N/B marks the species with no barcodes available from this study.

ON=Ontario, QC=Quebec, NWT=Northwest Territories, SK=Saskatchewan, AB=Alberta, NU=Nunavat, BC=British Columbia, NFLD=Newfoundland, Greenland=GR

| | Hist | Cont | Affinities | 01-Jun | 02-Jun | 01-Jul | 02-Jul | 01-Aug | 02-Aug | Tot |
|---|---|---|---|---|---|---|---|---|---|---|
| *Dolichogenidea* jft09 | + | - | QC | 0 | 0 | 0 | 1 | 0 | 1 | 2 |
| *Dolichogenidea* jft10 | + | - | Only Churchill | 0 | 0 | 2 | 5 | 0 | 0 | 7 |
| *Glyptapanteles compressiventris* * | + | - | Only Churchill | 3 | 0 | 3 | 0 | 0 | 0 | 6 |
| *Glyptapanteles* jft01 | - | + | NWT | 0 | 0 | 6 | 3 | 1 | 17 | 27 |
| *Glyptapanteles* jft02 | - | + | Only Churchill | 0 | 0 | 0 | 0 | 0 | 1 | 1 |
| *Glyptapanteles* jft03 | - | + | Only Churchill | 0 | 0 | 0 | 1 | 1 | 0 | 2 |
| *Glyptapanteles* jft04 | - | + | Only Churchill | 0 | 0 | 0 | 0 | 0 | 2 | 2 |
| *Glyptapanteles* jft05 | - | + | NU, QC, GR | 0 | 1 | 0 | 0 | 2 | 37 | 40 |
| *Glyptapanteles* jft06 | - | + | Only Churchill | 0 | 0 | 0 | 0 | 1 | 0 | 1 |
| *Glyptapanteles* jft07 | - | + | NU, NWT | 0 | 0 | 2 | 1 | 0 | 0 | 3 |
| *Glyptapanteles* jft08 | - | + | Only Churchill | 0 | 0 | 2 | 0 | 0 | 0 | 2 |
| *Glyptapanteles* jft09 | - | + | NU, NWT | 0 | 0 | 0 | 0 | 0 | 1 | 1 |
| *Illidops* jft01 | + | - | Only Churchill | 0 | 0 | 1 | 0 | 0 | 0 | 1 |
| *Illidops* jft02 | + | + | QC, NWT, NU | 0 | 2 | 0 | 0 | 0 | 0 | 2 |
| *Illidops* jft03 | + | - | QC | 4 | 0 | 2 | 0 | 4 | 1 | 11 |
| *Microgaster canadensis* | - | + | SK | 0 | 0 | 0 | 0 | 1 | 1 | 2 |
| *Microgaster deductor* ** | + | - | Only Churchill | 0 | 2 | 16 | 13 | 7 | 0 | 38 |
| *Microgaster* jft01 | + | - | Only Churchill | 0 | 1 | 0 | 0 | 0 | 0 | 1 |
| *Microgaster* jft02 | - | + | same as Microgaster jft02 | 0 | 6 | 2 | 1 | 3 | 2 | 14 |
| *Microgaster* jft03 | - | + | YK | 0 | 0 | 1 | 0 | 0 | 0 | 1 |
| *Microgaster* jft04 | - | + | Only Churchill | 0 | 0 | 1 | 0 | 0 | 0 | 1 |
| *Microgaster* jft05 | - | + | SK, YU | 0 | 0 | 2 | 0 | 0 | 0 | 2 |
| *Microgaster* jft06 | - | + | Only Churchill | 0 | 4 | 3 | 1 | 1 | 0 | 9 |
| *Microgaster* jft07 | - | + | Only Churchill | 0 | 0 | 1 | 0 | 0 | 0 | 1 |
| *Microgaster* jft08 | + | + | NWT | 0 | 0 | 2 | 0 | 0 | 0 | 2 |
| *Microgaster* jft09 | + | - | YU, AL | 0 | 1 | 0 | 0 | 0 | 0 | 1 |
| *Microplitis varicolor* * | + | + | YU, ON, AL, QC | 0 | 15 | 9 | 12 | 22 | 15 | 73 |
| *Microplitis* jft01 | + | - | YU, GR, NWT | 1 | 18 | 1 | 0 | 0 | 0 | 20 |
| *Microplitis* jft02 | - | + | AL | 0 | 0 | 0 | 6 | 1 | 1 | 8 |
| *Microplitis* jft03 | + | - | SK | 0 | 0 | 1 | 0 | 0 | 0 | 1 |

Table S1: Species of Microgastrinae from Churchill, Manitoba, Canada. Data comprise the first half of the 20th century (1930-1950's), referred as "Hist" in the heading of the second column; and recent collecting (2005-2007), referred as "Cont" in the heading of the third column. Species presence (+) or absence (-) is shown for Old and New data. Collecting dates were grouped in first and second half of every month sampled, indicated in the table headings with Arabic numbers (1 or 2) followed by the corresponding month (June=Jun, July=Jul, August=Aug). New records for Manitoba (*) or the Nearctic (**) are marked in the table. N/B marks the species with no barcodes available from this study.

ON=Ontario, QC=Quebec, NWT=Northwest Territories, SK=Saskatchewan, AB=Alberta, NU=Nunavat, BC=British Columbia, NFLD=Newfoundland, Greenland=GR

| | Hist | Cont | Affinities | 01-Jun | 02-Jun | 01-Jul | 02-Jul | 01-Aug | 02-Aug | Tot |
|---|---|---|---|---|---|---|---|---|---|---|
| *Microplitis* jft04 | - | + | NU, QC | 0 | 9 | 5 | 4 | 6 | 18 | 42 |
| *Microplitis* jft05 | - | + | ON, NWFLD | 0 | 1 | 0 | 0 | 0 | 0 | 1 |
| *Microplitis* jft06 | - | + | NWT | 0 | 0 | 1 | 1 | 2 | 0 | 4 |
| *Microplitis* jft07 | - | + | QC, ON | 0 | 0 | 0 | 7 | 0 | 0 | 7 |
| *Microplitis* jft08 | - | + | YU, BC, NWT | 0 | 0 | 4 | 4 | 0 | 0 | 8 |
| *Microplitis* jft09 | - | + | ON | 0 | 0 | 0 | 1 | 0 | 0 | 1 |
| *Microplitis* jft10 | + | + | Only Churchill | 0 | 2 | 3 | 0 | 0 | 0 | 5 |
| *Microplitis* jft11 | + | - | QC | 0 | 0 | 0 | 0 | 1 | 0 | 1 |
| *Microplitis* jft12 | + | + | YU, NWT | 0 | 1 | 0 | 2 | 1 | 0 | 4 |
| *Pholetesor bedelliae* * | + | + | YU, SK, ON | 0 | 0 | 1 | 2 | 0 | 3 | 6 |
| *Pholetesor ornigis* | - | + | SK, ON, QC, YU | 0 | 0 | 12 | 5 | 1 | 0 | 18 |
| *Pholetesor salicifoliellae* * | - | + | ON, YU | 0 | 3 | 6 | 4 | 9 | 1 | 23 |
| *Pholetesor viminetorum* * | + | + | YU, ON, SK, NWT | 0 | 2 | 9 | 17 | 3 | 0 | 31 |
| *Pholetesor* jft01 | - | + | NU | 0 | 0 | 0 | 1 | 2 | 0 | 3 |
| *Pholetesor* jft02 | - | + | Only Churchill | 0 | 0 | 0 | 2 | 0 | 0 | 2 |
| *Protapanteles* jft01 | + | + | YU, AB | 0 | 1 | 0 | 1 | 0 | 0 | 2 |
| *Protapanteles* jft02 | - | + | ON | 0 | 0 | 5 | 0 | 3 | 0 | 8 |
| *Protapanteles* jft03 N/B | - | + | Only Churchill | 0 | 0 | 0 | 1 | 0 | 0 | 1 |
| *Sathon* jft01 | - | + | Only Churchill | 0 | 0 | 0 | 0 | 0 | 1 | 1 |
| **Total number of specimens** | **171** | **489** | | **15** | **90** | **129** | **151** | **148** | **127** | **660** |
| **Total number of species** | **30** | **64** | | **6** | **27** | **41** | **36** | **37** | **29** | **79** |
