# Supplementary material for: A Poorly Known High-Latitude Parasitoid Wasp Community: Unexpected Diversity and Dramatic Changes through Time
Source: PLoS One. 2011 Aug 29;6(8):e23719. doi: 10.1371/journal.pone.0023719 (PMC3163582; doi:10.1371/journal.pone.0023719)
Supplement: Table S2 — Shannon-Wiener (H′) and Evenness (J) diversity indices of Microgastrinae from Churchill, Manitoba, Canada. Collecting dates were grouped in first and second half of every month sampled, indicated in the table headings with Arabic numbers (1 or 2) followed by the corresponding month (June = Jun, July = Jul, August = Aug).The last column refers to all dates and specimens combined. (PDF) [file pone.0023719.s005.pdf]

Table S2: Shannon-Wiener (H') and Evenness (J) diversity indices of Microgastrinae from Churchill, Manitoba, Canada. Collecting dates were grouped in first and second half of every month sampled, indicated in the table headings with Arabic numbers (1 or 2) followed by the corresponding month (June=Jun, July=Jul, August=Aug).The last column refers to all dates and specimens combined.

	1/Jun	2/Jun	1/Jul	2/Jul	1/Aug	2/Aug	TOTAL
H’	1.679204	2.758412	3.311068	3.027475	3.187291	2.482828	3.66664
J	0.937182	0.836938	0.891613	0.844833	0.882682	0.737336	0.839154
