# Supplementary material for: A Poorly Known High-Latitude Parasitoid Wasp Community: Unexpected Diversity and Dramatic Changes through Time
Source: PLoS One. 2011 Aug 29;6(8):e23719. doi: 10.1371/journal.pone.0023719 (PMC3163582; doi:10.1371/journal.pone.0023719)
Supplement: Table S3 — Taxonomic commentary and context for selected species of Microgastrinae from Churchill, Manitoba, Canada. (PDF) [file pone.0023719.s006.pdf]

Table S3: Taxonomic commentary and context for selected species of Microgastrinae from Churchill, Manitoba, Canada.

Apanteles fumiferanae Viereck, 1912. First record for Manitoba. The specimens observed have yellowish brown tegulae instead of blackish as stated by Mason ((1)), but otherwise share the characteristics of the species. Ongoing research suggests that *A. fumiferanae* may be a species complex, but resolving this point is beyond the scope of this work.

Apanteles polychrosidis Viereck, 1912. First record for Manitoba. This species shows a genetic dissimilarity of just 1.04 % compared to *Apanteles* jft01, however the two species are easily separated morphologically and by a single base pair 28S haplotype.

Apanteles jft01. One specimen (07PROBE-23049) has slight morphological differences (legs slightly darker, tergite 2 more sculptured). It also has over 1% of CO1 divergence but no 28S variation and without further sampling we feel that neither line of evidence is sufficient to warrant that singleton a different status.

Apanteles jft02. This species is similar to *A. fumiferanae* but slight morphological differences (in tegula colour and metatibial spines length) as well as consistent and large genetic dissimilarities (over 7 %) support their specific distinctiveness.

Cotesia xylina (Say, 1836). Whether this species is valid has been questioned by Muesebeck ((2)) and Papp ((3)) who considered it as synonym of *C. yakutatensis* or *C. tibialis* (Curtis)

respectively. Indeed the specimens from Churchill seem closely related to those of *C. yakutatensis. C. xylina* forms three distinct barcode clusters separated by up to 1.9 % (28S data is only available for one of these barcode clusters and so a comparison is not possible), however we refrained from changing the present status until more material and/or different localities are available for study. Based on the keys of Muesebeck ((2)), Papp ((3-5)) and van Achterberg ((6)), some of these specimens might be *C. eliniae*, *C. halli*, *C. melanoscela*, *C. tetricus* or none of those, but they are most likely new species.

Cotesia yakutatensis (Ashmead, 1902). First record for Manitoba. Within the Churchill species, it is morphologically close to *Cotesia xylina* and *Cotesia* jft02, with the CO1 divergences between 3.6 and 4.1% respectively. It is likely that *Cotesia yakutatensis* and related species in this work (*C. xylina*, *Cotesia* jft02, *Cotesia* jft06 and *Cotesia* jft08) form a species complex.

Cotesia jft01. The only available specimen, a male, runs to *C. nemoriae* in Muesebeck ((7)) and to *C. subordinanius* in Papp ((3)) keys but it is neither of those species. Its huge metacoxae (half the length of the metasoma) are very distinctive and seem a good character to consider it as a new species.

Cotesia jft04. This species is near to *Cotesia brevicornis* (Wesmael,) (the latter known from the Palearctic region as well as Alberta in Canada) and appears similar to that species.

Cotesia jft05. Belongs to the *glomerata* species-group and runs through couplet number 206 in the key from Papp ((4)). We checked specimens of *C. acauda*, *C. mahoniae, C. phobetri* and *C.*

pyrophilae (mentioned in Papp's key as closely related) but the Churchill specimens have longer ovipositor sheaths and a more blackish coloration than the above mentioned species. While morphologically similar (especially to *C. acauda*), its status should be established when the *glomerata* species-group is revised within the Nearctic.

Cotesia jft09. Related to Holoarctic species of *Cotesia* with short antennae —e.g. *C. tenebrosa* (Wesmael) and *C. arctica* (Thompson) — but differing from all of the species keyed by Papp (1976). It is very likely a new species.

Dolichogenidea jft02. This species is near to *D. clavata*, differing mostly in the ovipositor shape.

Dolichogenidea jft05. The specimens run through Papp ((8)) key to *D. laevigata* and seem to be similar, but differ from that species by being darker –especially legs and antenna– and also with thinner stigma in forewing and shorter antennal segments at apex. It may also be related to *D. clavata*.

Dolichogenidea jft03 and jft06. These two species are morphologically related to D. *banksi*, differing between each other in palpi colour, elongation of the malar space and body colour pattern.

Glyptapanteles compressiventris (Muesebeck, 1921). First record for Manitoba.

Glyptapanteles spp. (*Glyptapanteles* jft01, jft04, jft05, jft06, jft08, jft09). Several species from Churchill are related to *G. alticola* Ashmead, 1902, and one of them is possibly that species, with the rest being differentiated by the molecular data as well as slight morphological differences (e.g. antennae colour, relative length of the last flagellomeres, puncture density of head, hair density and length on the mesoscutum, scutellum punctures, wing base colour, propodeal carination, metathoracic leg colour —especially tibia & tarsi— sculpture of medio tergites 1 and 2). Barcoding data formed well defined clusters with greater than 5 % interspecific divergence values, and we have no doubts that they are valid species. Without having molecular data from type material and/or a comprehensive taxonomical review of the genus within the Nearctic unequivocal assignment to *G. alticola* is not possible. This example clearly illustrates how advantageous it would be to have barcode data from type material, something that at present is not easy to accomplish (due both to technological and regulatory issues). After studying the material housed at the CNC, we agree with Papp's ((9)) statement that *G. alticola* is not different from *G. fulvipes* (Haliday).

Microgaster deductor Nixon 1968. New record for the Nearctic region, previously known from the Western Palearctic. The presence of a basal lobe on the claws is a very distinctive and unique character for an accurate identification of this species.

Microgaster jft01. The only specimen available, a male, looks related to *M. sticticus* Ruthe, 1858 (from the Palearctic region) but more material is needed before its status can be clearly defined.

Microgaster jft02. The small size (about 2.5 mm), subparallel eyes, rugulose mesoscutum and small length of ovipositor relate this species to the European *M. fischeri* Papp, 1960, but it is most likely a new species.

Microgaster jft06 and jft07. They seem morphologically related, the second species represented by a single male. However, inter-specific barcodes divergences (2.1%) are more than 20 times larger than intra-specific divergence (0.09%), and both have characteristic 28S haplotypes; thus we consider them as two separate species.

Microplitis varicolor Viereck, 1917. First record for Manitoba and northernmost record of the species to date.

Microplitis jft01. We have not seen authoritatively named specimens from Europe to compare, but according to the descriptions provided by Papp ((10)) and Achterberg ((6)) this species is closely related to *M. coactus* (Lundbeck, 1896), which was previously known just from Greenland and Iceland. The specimens from Churchill may represent a different and new species, with larger metafemur.

Microplitis jft03. It is similar to *Microplitis* jft01 but median tergite 1 sculpture and CO1 data are clearly different, thus we consider them as two species.

Microplitis jft05 and jft06. They are morphologically similar to each other and to *M. varicolor* but with clear morphological (length of metatibial spurs and antennal segments, plus scape colour) and genetic discontinuities.

Pholetesor viminetorum and *P.* jft02. First record for Manitoba. Two male specimens of *Pholetesor* jft02 (07PROBE-22417, 07PROBE-23399) differ slightly from *P. viminetorum* regarding veins r and 2RS, length of metatibial spurs and shape of tergite 1 and 2. The barcode variation between these two species was 1.94 %, and there are also two character states differences within the D2 region of 28S. The combination of these three lines of evidence allows us to consider those males as a separate species from *P. viminetorum*.

Pholetesor jft01. This is likely a new species related to *P. powelli*, *P. bedelliae* and *P. thuiellae* but clearly different from them. A study within the context of Holoarctic species is badly needed.

Protapanteles alaskensis (Ashmead, 1902). First record for Manitoba.

REFERENCES

1. Mason WRM (1974) The *Apanteles* species (Hymenoptera: Braconidae attacking Lepidoptera in the micro-habitat of the spruce budworm (Lepidoptera: Tortricidae). *Canadian Entomologist* 106:1087-1102.
2. Muesebeck CFW (1921) A revision of the North American species of ichneumon-flies belonging to the genus *Apanteles*. *Proceedings of the United States National Museum* 58:483-576.
3. Papp J (1986) A survey of the European species of *Apanteles* Först. (Hymenoptera, Braconidae: Microgastrinae). IX. The glomeratus-group. *Annales Historico-Naturales Musei Nationalis Hungarici.* 78:225-247.
4. Papp J (1987) A survey of the European species of *Apanteles* Förster (Hymenoptera, Braconidae: Microgastrinae), X. The glomeratus-group 2 and the cultellatus-group. *Annales Historico-Naturales Musei Nationalis Hungarici* 79:207-258.
5. Papp J (1989) Contribution to the Braconid wasp of Greenland, Denmark (Hymenoptera: Braconidae). *Folia Entomologica Hungarica* 100:95-104.
6. van Achterberg K (2006) The Braconidae (Hymenoptera) of Greenland. *Zool. Med. Leiden* 80:13-62.
7. Muesebeck CFW (1922) A revision of the North American ichneumon-flies, belonging to the subfamilies Neoneurinae and Microgasterinae. *Proceedings of the United States National Museum* 61(2436):1-76.
8. Papp J (1978) A survey of the European species of *Apanteles* Först. (Hymenoptera, Braconidae: Microgasterinae), II. The laevigatus-group. *Annales Historico-Naturales Musei Nationalis Hungarici* 70:265-301.
9. Papp J (1983) A survey of the European species of *Apanteles* Först. (Hymenoptera, Braconidae: Microgastrinae), VII. The carbonarius-, circumscriptus-, fraternus-, pallipes-, parasitellae-, vitripennis-, liparidis-, octonarius- and thompsoni- group *Annales Historico-Naturales Musei Nationalis Hungarici* 75 (247-283).
10. Papp J (1984) Palaearctic species of *Microgaster* Latreille (=*Microplitis* Förster) with description of seven new species (Hymenoptera, Braconidae, Microgastrinae). *Entomologische Abhandlungen* 47:95-140.
