# Supplementary material for: A Poorly Known High-Latitude Parasitoid Wasp Community: Unexpected Diversity and Dramatic Changes through Time
Source: PLoS One. 2011 Aug 29;6(8):e23719. doi: 10.1371/journal.pone.0023719 (PMC3163582; doi:10.1371/journal.pone.0023719)
Supplement: Table S4 — Morphological characters used to study the species of Microgastrine from Churchill, Manitoba, Canada; and its definition. Based on Mason, 1981 [1], Whitfield et al., 2002 [2], Smith et al., 2008 [3], Fernandez-Triana and Goulet, 2009 [4], Valerio and Whitfield, 2009 [5], and unpublished data from JFT. (PDF) [file pone.0023719.s007.pdf]

Table S4: Morphological characters used to study the species of Microgastrine from Churchill, Manitoba, Canada; and its definition. Based on Mason, 1981 [1], Whitfield et al., 2002 [2], Smith et al., 2008 [3], Fernandez-Triana and Goulet, 2009 [4], Valerio and Whitfield, 2009 [5], and unpublished data from JFT.

1- Head Height/Width: Quantitative measurement. Height is measured from ocelli to base of mandibles; width is the widest point between outer edges of eyes.
2- Face Rostriformis: Yes/No. “Rostriformis" refers to face elongation but needs to be defined yet to accurately depict the degree of variation in this character.
3- Head Width below Ocelli/Face Width at Tentorial Pits: Quantitative measurement to show degree of convergence in eyes.
4- Eyes Height/Head Height: Quantitative measurement. Head Height as in 1; eyes maximum height measured in the same position.
5- Ocello-ocular Distance/Posterior Ocelli Diameter: Quantitative measurement from ocellus edge to the nearest edge of eye.
6- Distance betwen Ocelli/Ocelli Diameter: Quantitative measurement. It refers to posterior ocelli.
7- Malar Space: Shorter or about the same of mandible width at base/Larger than mandibular base.
8- Clypeus Width/Height: Quantitative measurement.
9- Palpi Color: Pale/Pale in basal segments-Dark apically/Dark. NOTE: Pale can be Yellow/White; Dark can be Brown/Black.
10- Glossa: Truncate/Bilobed but short/Divaricate. NOTE: Sensu Whitfield et al. (2002)
11- Antenna Length/Body Length: Antenna shorter than body/Antenna about the same length or larger than body. Body length measured from head to metasoma. NOTE: Some species have a very short antenna (shorter than length of head plus mesosoma) that may require further clarification.
12- Flagellar Segment 2 Length/Width: >2.0X/1.5-2.0X/1.0-1.4X/<1.0X.
13- Flagellar Segment 14 Length/Width: >2.0X/1.5-2.0X/1.0-1.4X/<1.0X.
14- Flagellar Segment 14 Length/Segment 2 Length: Quantitative measurement.
15- Lateral Pronotal Grooves: One/Two/Lower margin of pronotum excavated. NOTE: Sensu Whitfield et al. (2002).
16- Tegula Color: Pale/Pale apically-Dark basally/Dark. NOTE: Pale can be Yellow/White/Translucent; Dark can be Orange-Brown/Brown/Black.
17- Mesoscutum Punctures: Smooth/Shallow, sparse punctures/Closer, deeper punctures. NOTE: Sparse punctures are those separate for more than twice its maximum diameter; close punctures are separated by less than 2X its maximum diameter. Some genera with very deep, coarse and close punctures may require further definition, especially related to depth of punctures.
18- Notauli: Complete and well defined/Partially defined, marked by shallow sulcus/Not defined at all.
19- Scutellum Punctures: Mostly smooth/With a few punctures, mostly near the borders/Mostly or completely punctured.
20- Scutellum Length/Width: Length measured from the distal border of scutellar suture (i.e. excluding the suture) to medioposterior band; width measured at base of scutellum.

21- Scutellum Lunules: Transverse (<10% the maximum height of the lateral face of scutellum)/Semicircular (about 50 %)/Triangular (>70%).

22- Number of Costulae in Scutellar Suture: Quantitative number.

23- Scutellar Suture: Wide and deep, well defined/More or less wide but shallow/Narrow, almost obliterated, and usually superficial.

24- Medioposterior Band of Scutellum: Smooth/Sculptured

25- Sublateral Hairs on Metanotum: Sensu Mason (1981), but needs to be better defined.

26- Anterior Margin of Metanotum: Appressed to scutellum/Excavated sublaterally, exposing phragma sublaterally/Sloping away, phragma well exposed.

27- Dorsal, Anterior, Horizontal portion of Propodeum: Angled relative to posterior declivous portion at about mid-length of propodeum/Dorsal part greatly shortened/Dorsal part evenly and gently curved relative to posterior portion of propodeum.

28- Propodeum Background Sculpture: Mostly smooth/Partially sculptured /Mostly sculptured (punctures, striation, etc)/Irregular carinae pattern covering most of the propodeum.

29- Propodeal Areola: Present and complete/Incomplete (broadly open anteriorly)/Absent.

30- Medial Longitudinal Carina of Propodeum: Present and complete/Present only anteriorly/Present only posteriorly/Absent. NOTE: Partial presence may be as divided (parallel) short carinae.

31- Transverse Carinae of Propodeum: Present/Absent.

32- Stigma Color: Dark/Pale at base/Pale with only borders dark/Fully pale, translucent. NOTE: Dark can be Black/Brown/Orange; Pale can be White/Light Yellow.

33- Veins Color: Mostly dark (some basal veins may be unpigmented)/Partially pigmented (only a few veins are dark, mostly pale)/Mostly unpigmented (white color). NOTE: Dark can be Brown/Orange; Pale can be White/Light Yellow.

34- Length of Vein r/Length of Vein 2RS in Forewing: >2.0 X/2.0-1.5X/<1.5-1.0X/<1

35- Veins r and 2RS Shape: Evenly curved/Distinctly but not strongly angulated/Strongly angulated. NOTE: The presence of a stub needs to be defined and incorporated.

36- Second Submarginal Cell (Areolet) Shape: Vein r-m meeting 3Rsa (areolet 4 sided and usually large)/Vein r-m meeting 2RS near its junction with r, or meeting the junction by itself (large 3 sided, triangular)/ Vein r-m meeting 2RS near its junction with M (small triangular, sometimes not well defined areolet)/Vein r-m absent (no areolet).

37- Second Submarginal Cell (Areolet) Height: <0.3/0.3-0.5/>0.5 NOTE: The height of areolet compares to the distance between vein M and the point of stigma where vein r arises. NOTE: Sensu Fernandez-Triana and Goulet (2009).

38- Basal Vein (1M+1Rs) of Forewing: Conspicuously angled (~90°) at junction of M and Rs/Not conspicuously angled.

39- Vein R1 Length/Stigma Length: Quantitative measurement.

40- Stigma Length/Width: Quantitative measurement.

41- Distance between Vein R1 and 3RS/Vein R1 Length: Quantitative measurement.

42- Height/Width of First Discal Cell in Forewing: Quantitative measurement.

43- Vannal Margin of Hindwing: Distinctly convex beyond widest point/Distally flattened to concave beyond widest point.

44- Vannal Fringe of Hindwing: Long, even, and dense beyond broadest point of clavum/Short, much sparser beyond broadest point/Absent beyond broadest point.

45- Tarsal Claws: Simple/Pectinated/Single basal tooth. NOTE: Sensu Whitfield et al. (2002).

46- Female Distal Protarsomere 5: Normal/Excavated ventrally with curved seta.

47- Coxa Color: Dark/Pale; formula 1/2/3 refers to legs. NOTE: Dark can be Black/Dark brown, Pale can be Light Brown/Orange/Yellow.
48- Femur Color: Dark/Pale; formula 1/2/3 refers to legs. NOTE: Dark can be Black/Dark brown, Pale can be Light Brown/Orange/Yellow.
49- Tibia Color: Dark/Pale; formula 1/2/3 refers to legs. NOTE: Dark can be Black/Dark brown, Pale can be Light Brown/Orange/Yellow.
50- Tarsi Color: Dark/Pale; formula 1/2/3 refers to legs. NOTE: Dark can be Black/Dark brown, Pale can be Light Brown/Orange/Yellow.
51- Metacoxa Size: Shorter than T1/About the same size than T1, can be slightly larger/Reaching apex of T2/Surpasing apex of T2.
52- Metafemur Length/Width: Quantitative measurement.
53- Metatibia Inner Spur Length/Outer Spur Length: Quantitative measurement.
54- Metatibial Inner Spur Length/Basitarsus Length: Quantitative measurement.
55- Medio Tergite 1 Shape: Clearly widening at apex/Parallel sided or barrel shaped or slightly wide at apex or base/Clearly narrowing at apex.
56- Medio Tergite 1 Length/Width at Apex: 2/1.5/1/<1. NOTE: The values provide an approximated value to define the classes; they should be rounded to the nearest range when measured.
57- Medio Tergite 1 Basal Excavation: Present/Absent. NOTE: Sensu Mason (1981).
58- Medio Tergite 1 Mediobasal Sharp Longitudinal Groove: Present/Absent.
59- Medio Tergite 1 Apical Half: Flat to gently arched, without excavation/With shallow medial longitudinal excavation.
60- Medio Tergite 2 Shape: Rectangular and covering most of the dorsal surface of tergum/Broadly sub-triangular (rather trapezoidal) or transverse, much broader than medially long/Narrowly sub-triangular (trapezoidal)/Desclerotized anterolaterally and mediapically to form a slender, inverted Y shape. NOTE: Sensu Whitfield et al. (2002).
61- Delineation between Medio Tergites 2 and 3: Fine, distinct suture/Weak or absent/Broad crenulate or transcostate groove.
62- Hairs on Terga 3+: Abundant and rather completely and evenly dispersed/Less numerous and grouped in various patterns or lateral clumps or transverse bands/Pointing in various directions to form patterns. NOTE: Sensu Mason (1981).
63- Laterotergites and Sternites Color: Pale/Dark. NOTE: Color may be different between basal and apical lateral tergites and sternites, and thus must be defined accordingly.
64- Hypopygium: Evenly sclerotized, not medially folded/More or less evenly sclerotized but sharply folded medially/Medially strongly desclerotized and longitudinally folded into pleats.
65- Hypopigium Size: Shorter than last sternites/About the same size/Larger than last sternites.
66- Hypopigial Tip Shape: Angled/Evenly curved to point/Apically truncate.
67- Ovipositor Sheath Length/Metatibia Length: <1/1/1.5/2/>2. NOTE: The values provide an approximated value to define the classes; they should be rounded to the nearest range when measured.
68- Ovipositor Shape and Length: Long, evenly tapered or sinuate at apex/Short, thick basally with abrupt attenuation near or beyond the middle/Rather short, strongly curved downward through 90° or more.
69- Distribution of Hairs in the Ovipositor Sheath: Uniformly distributed over apical half or more of its length/With some areas of sparse or absent hairs/Concentrated at the extreme apex.

70- Apical Hairs in the Ovipositor Sheaths: As large or larger than normal hairs in the rest of metasoma (especially last sternal segments)/Much smaller than hairs in the rest of metasoma/Modified to form dome-shaped sensilla or thick, apically truncated hairs.

71- Body Length: Quantitative measurement (mm). NOTE: Body length measured from head to metasoma, ie. excluding the ovipositor.

REFERENCES:

1. Mason WRM (1981) The polyphyletic nature of *Apanteles* Foerster (Hymenoptera: Braconidae): A phylogeny and reclassification of Microgastrinae. Memoirs of the Entomological Society of Canada 115: 1-147.
2. Whitfield JB, Mardulyn P, Austin AD, Dowton M (2002) Phylogenetic relationships among microgastrine braconid wasp genera based on data from the 16S, COI and 28S genes and morphology. Systematic Entomology 27: 337-359.
3. Smith MA, Rodriguez JJ, Whitfield JB, Deans AR, Janzen DH, et al. (2008) Extreme diversity of tropical parasitoid wasps exposed by iterative integration of natural history, DNA barcoding, morphology, and collections. Proc Natl Acad Sci U S A 105: 12359-12364.
4. Fernández-Triana J, Goulet H, Bostanian N, Boudreault C (2009) Diversity of Microgastrinae (Hymenoptera: Braconidae) in apple orchards of southern Quebec, Canada. Biocontrol Science and Technology 19: 237-248.
5. Valerio AA, Whitfield JB (2009) Review of world *Parapanteles* Ashmead (Hymenoptera: Braconidae: Microgastrinae), with description of fourteen new Neotropical species and the first description of the final instar larvae. Zootaxa 2084: 1-49.
