# Supplementary material for: A Poorly Known High-Latitude Parasitoid Wasp Community: Unexpected Diversity and Dramatic Changes through Time
Source: PLoS One. 2011 Aug 29;6(8):e23719. doi: 10.1371/journal.pone.0023719 (PMC3163582; doi:10.1371/journal.pone.0023719)
Supplement: Table S5 — All collection and accession information associated with specimens analysed here. (PDF) [file pone.0023719.s008.pdf]

Table S5: All collection and accession information associated with specimens analysed here.

ProcessID	Sample ID	Field Num	Catalog Num	COI-5P Seq.	COI GenBank	28S Seq. Length	28S GenBank	Sample ID	Phylum	Class	Order	Family	Subfamily	Genus	Species	Identifier	Identifier email	Sex
ASCNC014-09	CAM0629	CAM0629	CAM0629	657	GU715080			CAM0629	Arthropoda	Insecta	Hymenoptera	Braconidae	Microgastrinae	Protapanteles	Protapanteles jft02	Jose Leonardo Fernandez	jftriana@uoguelph.ca	
ASCNC015-09	CAM0630	CAM0630	CAM0630	657	GU715081			CAM0630	Arthropoda	Insecta	Hymenoptera	Braconidae	Microgastrinae	Protapanteles	Protapanteles jft02	Jose Leonardo Fernandez	jftriana@uoguelph.ca	
ASCNC016-09	CAM0631	CAM0631	CAM0631	657	GU715079			CAM0631	Arthropoda	Insecta	Hymenoptera	Braconidae	Microgastrinae	Protapanteles	Protapanteles jft02	Jose Leonardo Fernandez	jftriana@uoguelph.ca	
ASCNC021-09	CAM0636	CAM0636	CAM0636	657	GU715077			CAM0636	Arthropoda	Insecta	Hymenoptera	Braconidae	Microgastrinae	Dolichogenidea	Dolichogenidea jft05	Jose Leonardo Fernandez	jftriana@uoguelph.ca	
ASWA1026-08	07PROBE-20358	07PROBE-20358	07PROBE-20358	622	GU141342			07PROBE-20358	Arthropoda	Insecta	Hymenoptera	Braconidae	Microgastrinae	Pholetesor	Pholetesor ornigis	Jose Leonardo Fernandez	jftriana@uoguelph.ca	
ASWA1027-08	07PROBE-20359	07PROBE-20359	07PROBE-20359	622	GU141341			07PROBE-20359	Arthropoda	Insecta	Hymenoptera	Braconidae	Microgastrinae	Pholetesor	Pholetesor ornigis	Jose Leonardo Fernandez	jftriana@uoguelph.ca	
ASWA1028-08	07PROBE-20360	07PROBE-20360	07PROBE-20360	655	GU141172			07PROBE-20360	Arthropoda	Insecta	Hymenoptera	Braconidae	Microgastrinae	Glyptapanteles	Glyptapanteles jft01	Jose Leonardo Fernandez	jftriana@uoguelph.ca	
ASWA1029-08	07PROBE-20361	07PROBE-20361	07PROBE-20361	622	GU141102			07PROBE-20361	Arthropoda	Insecta	Hymenoptera	Braconidae	Microgastrinae	Cotesia	Cotesia jft08	Jose Leonardo Fernandez	jftriana@uoguelph.ca	
ASWA1031-08	07PROBE-20363	07PROBE-20363	07PROBE-20363	656	GU141049			07PROBE-20363	Arthropoda	Insecta	Hymenoptera	Braconidae	Microgastrinae	Apanteles	Apanteles jft01	Jose Leonardo Fernandez	jftriana@uoguelph.ca	
ASWA1032-08	07PROBE-20364	07PROBE-20364	07PROBE-20364	653	GU141279			07PROBE-20364	Arthropoda	Insecta	Hymenoptera	Braconidae	Microgastrinae	Microplitis	Microplitis jft08	Jose Leonardo Fernandez	jftriana@uoguelph.ca	
ASWA1033-08	07PROBE-20365	07PROBE-20365	07PROBE-20365	654	GU141140			07PROBE-20365	Arthropoda	Insecta	Hymenoptera	Braconidae	Microgastrinae	Diolcogaster	Diolcogaster jft01	Jose Leonardo Fernandez	jftriana@uoguelph.ca	
ASWA1034-08	07PROBE-20366	07PROBE-20366	07PROBE-20366	645	GU141219			07PROBE-20366	Arthropoda	Insecta	Hymenoptera	Braconidae	Microgastrinae	Glyptapanteles	Glyptapanteles jft07	Jose Leonardo Fernandez	jftriana@uoguelph.ca	
ASWA1035-08	07PROBE-20367	07PROBE-20367	07PROBE-20367	631	GU141285			07PROBE-20367	Arthropoda	Insecta	Hymenoptera	Braconidae	Microgastrinae	Microplitis	Microplitis jft08	Jose Leonardo Fernandez	jftriana@uoguelph.ca	
ASWA1036-08	07PROBE-20368	07PROBE-20368	07PROBE-20368	631	GU141301			07PROBE-20368	Arthropoda	Insecta	Hymenoptera	Braconidae	Microgastrinae	Microplitis	Microplitis varicolor	Jose Leonardo Fernandez	jftriana@uoguelph.ca	
ASWA1070-08	07PROBE-20402	07PROBE-20402	07PROBE-20402	632	GU141300			07PROBE-20402	Arthropoda	Insecta	Hymenoptera	Braconidae	Microgastrinae	Microplitis	Microplitis varicolor	Jose Leonardo Fernandez	jftriana@uoguelph.ca	
ASWA1071-08	07PROBE-20403	07PROBE-20403	07PROBE-20403	657	GU141235			07PROBE-20403	Arthropoda	Insecta	Hymenoptera	Braconidae	Microgastrinae	Microgaster	Microgaster jft03	Jose Leonardo Fernandez	jftriana@uoguelph.ca	
ASWA1078-08	07PROBE-20410	07PROBE-20410	07PROBE-20410	620	GU141299			07PROBE-20410	Arthropoda	Insecta	Hymenoptera	Braconidae	Microgastrinae	Microplitis	Microplitis varicolor	Jose Leonardo Fernandez	jftriana@uoguelph.ca	
ASWA211-08	07PROBE-20013	07PROBE-20013	07PROBE-20013	657	GU141064			07PROBE-20013	Arthropoda	Insecta	Hymenoptera	Braconidae	Microgastrinae	Apanteles	Apanteles jft02	Jose Leonardo Fernandez	jftriana@uoguelph.ca	
ASWA222-08	07PROBE-20024	07PROBE-20024	07PROBE-20024	657	GU141286			07PROBE-20024	Arthropoda	Insecta	Hymenoptera	Braconidae	Microgastrinae	Microplitis	Microplitis jft09	Jose Leonardo Fernandez	jftriana@uoguelph.ca	
ASWA224-08	07PROBE-20026	07PROBE-20026	07PROBE-20026	657	GU141080			07PROBE-20026	Arthropoda	Insecta	Hymenoptera	Braconidae	Microgastrinae	Cotesia	Cotesia jft03	Jose Leonardo Fernandez	jftriana@uoguelph.ca	
ASWA270-08	07PROBE-20072	07PROBE-20072	07PROBE-20072	0		0		07PROBE-20072	Arthropoda	Insecta	Hymenoptera	Braconidae	Microgastrinae	Pholetesor	Pholetesor bedelliae	Jose Leonardo Fernandez	jftriana@uoguelph.ca	
ASWA289-08	HYM00001003	HYM00001003	HYM00001003	657				HYM00001003	Arthropoda	Insecta	Hymenoptera	Braconidae	Microgastrinae	Apanteles	Apanteles ensiger	Jose Leonardo Fernandez	jftriana@uoguelph.ca	
ASWA407-08	HYM00001121	HYM00001121	HYM00001121	619				HYM00001121	Arthropoda	Insecta	Hymenoptera	Braconidae	Microgastrinae	Cotesia	Cotesia jft02	Jose Leonardo Fernandez	jftriana@uoguelph.ca	
ASWA408-08	HYM00001122	HYM00001122	HYM00001122	626				HYM00001122	Arthropoda	Insecta	Hymenoptera	Braconidae	Microgastrinae	Cotesia	Cotesia jft02	Jose Leonardo Fernandez	jftriana@uoguelph.ca	
ASWA409-08	HYM00001123	HYM00001123	HYM00001123	291				HYM00001123	Arthropoda	Insecta	Hymenoptera	Braconidae	Microgastrinae	Cotesia	Cotesia jft02	Jose Leonardo Fernandez	jftriana@uoguelph.ca	
ASWA491-08	HYM00001205	HYM00001205	HYM00001205	606				HYM00001205	Arthropoda	Insecta	Hymenoptera	Braconidae	Microgastrinae	Cotesia	Cotesia jft03	Jose Leonardo Fernandez	jftriana@uoguelph.ca	
ASWA492-08	HYM00001206	HYM00001206	HYM00001206	597				HYM00001206	Arthropoda	Insecta	Hymenoptera	Braconidae	Microgastrinae	Cotesia	Cotesia jft03	Jose Leonardo Fernandez	jftriana@uoguelph.ca	
ASWA518-08	HYM00001232	HYM00001232	HYM00001232	657				HYM00001232	Arthropoda	Insecta	Hymenoptera	Braconidae	Microgastrinae	Microplitis	Microplitis auripes	Jose Leonardo Fernandez	jftriana@uoguelph.ca	
ASWA520-08	HYM00001234	HYM00001234	HYM00001234	642				HYM00001234	Arthropoda	Insecta	Hymenoptera	Braconidae	Microgastrinae	Microplitis	Microplitis jft03	Jose Leonardo Fernandez	jftriana@uoguelph.ca	F
ASWA606-08	HYM00001320	HYM00001320	HYM00001320	657				HYM00001320	Arthropoda	Insecta	Hymenoptera	Braconidae	Microgastrinae	Pholetesor	Pholetesor bedelliae	Jose Leonardo Fernandez	jftriana@uoguelph.ca	
ASWA608-08	HYM00001322	HYM00001322	HYM00001322	657				HYM00001322	Arthropoda	Insecta	Hymenoptera	Braconidae	Microgastrinae	Pholetesor	Pholetesor bedelliae	Jose Leonardo Fernandez	jftriana@uoguelph.ca	
ASWA609-08	HYM00001323	HYM00001323	HYM00001323	628				HYM00001323	Arthropoda	Insecta	Hymenoptera	Braconidae	Microgastrinae	Pholetesor	Pholetesor ornigis	Jose Leonardo Fernandez	jftriana@uoguelph.ca	
ASWA610-08	HYM00001324	HYM00001324	HYM00001324	630				HYM00001324	Arthropoda	Insecta	Hymenoptera	Braconidae	Microgastrinae	Pholetesor	Pholetesor ornigis	Jose Leonardo Fernandez	jftriana@uoguelph.ca	
ASWA611-08	HYM00001325	HYM00001325	HYM00001325	630				HYM00001325	Arthropoda	Insecta	Hymenoptera	Braconidae	Microgastrinae	Pholetesor	Pholetesor ornigis	Jose Leonardo Fernandez	jftriana@uoguelph.ca	
ASWA615-08	HYM00001329	HYM00001329	HYM00001329	657				HYM00001329	Arthropoda	Insecta	Hymenoptera	Braconidae	Microgastrinae	Pholetesor	Pholetesor viminetorum	Jose Leonardo Fernandez	jftriana@uoguelph.ca	
ASWA619-08	HYM00001333	HYM00001333	HYM00001333	657				HYM00001333	Arthropoda	Insecta	Hymenoptera	Braconidae	Microgastrinae	Microgaster	Microgaster jft10	Jose Leonardo Fernandez	jftriana@uoguelph.ca	
ASWA621-08	HYM00001335	HYM00001335	HYM00001335	657				HYM00001335	Arthropoda	Insecta	Hymenoptera	Braconidae	Microgastrinae	Microgaster	Microgaster jft05	Jose Leonardo Fernandez	jftriana@uoguelph.ca	F
ASWA622-08	HYM00001336	HYM00001336	HYM00001336	646				HYM00001336	Arthropoda	Insecta	Hymenoptera	Braconidae	Microgastrinae	Microgaster	Microgaster jft05	Jose Leonardo Fernandez	jftriana@uoguelph.ca	M
ASWA629-08	HYM00001343	HYM00001343	HYM00001343	657				HYM00001343	Arthropoda	Insecta	Hymenoptera	Braconidae	Microgastrinae	Microgaster	Microgaster jft05	Jose Leonardo Fernandez	jftriana@uoguelph.ca	M
ASWA630-08	HYM00001344	HYM00001344	HYM00001344	646				HYM00001344	Arthropoda	Insecta	Hymenoptera	Braconidae	Microgastrinae	Microgaster	Microgaster jft05	Jose Leonardo Fernandez	jftriana@uoguelph.ca	F
ASWA631-08	HYM00001345	HYM00001345	HYM00001345	631				HYM00001345	Arthropoda	Insecta	Hymenoptera	Braconidae	Microgastrinae	Microgaster	Microgaster jft05	Jose Leonardo Fernandez	jftriana@uoguelph.ca	M
ASWA637-08	HYM00001351	HYM00001351	HYM00001351	629				HYM00001351	Arthropoda	Insecta	Hymenoptera	Braconidae	Microgastrinae	Microgaster	Microgaster jft05	Jose Leonardo Fernandez	jftriana@uoguelph.ca	M
ASWA638-08	HYM00001352	HYM00001352	HYM00001352	657				HYM00001352	Arthropoda	Insecta	Hymenoptera	Braconidae	Microgastrinae	Microgaster	Microgaster jft05	Jose Leonardo Fernandez	jftriana@uoguelph.ca	M
ASWA644-08	HYM00001358	HYM00001358	HYM00001358	657				HYM00001358	Arthropoda	Insecta	Hymenoptera	Braconidae	Microgastrinae	Microgaster	Microgaster jft05	Jose Leonardo Fernandez	jftriana@uoguelph.ca	F
ASWA645-08	HYM00001359	HYM00001359	HYM00001359	631				HYM00001359	Arthropoda	Insecta	Hymenoptera	Braconidae	Microgastrinae	Microgaster	Microgaster jft05	Jose Leonardo Fernandez	jftriana@uoguelph.ca	F
ASWA726-08	HYM-00001440		HYM-00001440	631				HYM-00001440	Arthropoda	Insecta	Hymenoptera	Braconidae	Microgastrinae	Microplitis	Microplitis jft06	Jose Leonardo Fernandez	jftriana@uoguelph.ca	
ASWA772-08	07PROBE-20104	07PROBE-20104	07PROBE-20104	0		0		07PROBE-20104	Arthropoda	Insecta	Hymenoptera	Braconidae	Microgastrinae	Dolichogenidea	Dolichogenidea jft04	Jose Leonardo Fernandez	jftriana@uoguelph.ca	
ASWA920-08	07PROBE-20252	07PROBE-20252	07PROBE-20252	0		0		07PROBE-20252	Arthropoda	Insecta	Hymenoptera	Braconidae	Microgastrinae	Apanteles	Apanteles jft01	Jose Leonardo Fernandez	jftriana@uoguelph.ca	
ASWA923-08	07PROBE-20255	07PROBE-20255	07PROBE-20255	0		0		07PROBE-20255	Arthropoda	Insecta	Hymenoptera	Braconidae	Microgastrinae	Microplitis	Microplitis jft04	Jose Leonardo Fernandez	jftriana@uoguelph.ca	
ASWA941-08	07PROBE-20273	07PROBE-20273	07PROBE-20273	631	GU141176			07PROBE-20273	Arthropoda	Insecta	Hymenoptera	Braconidae	Microgastrinae	Glyptapanteles	Glyptapanteles jft01	Jose Leonardo Fernandez	jftriana@uoguelph.ca	
ASWA942-08	07PROBE-20274	07PROBE-20274	07PROBE-20274	657	GU141284			07PROBE-20274	Arthropoda	Insecta	Hymenoptera	Braconidae	Microgastrinae	Microplitis	Microplitis jft08	Jose Leonardo Fernandez	jftriana@uoguelph.ca	
ASWA943-08	07PROBE-20275	07PROBE-20275	07PROBE-20275	644	GU141241			07PROBE-20275	Arthropoda	Insecta	Hymenoptera	Braconidae	Microgastrinae	Microgaster	Microgaster jft06	Jose Leonardo Fernandez	jftriana@uoguelph.ca	
ASWA944-08	07PROBE-20276	07PROBE-20276	07PROBE-20276	655	GU141283			07PROBE-20276	Arthropoda	Insecta	Hymenoptera	Braconidae	Microgastrinae	Microplitis	Microplitis jft08	Jose Leonardo Fernandez	jftriana@uoguelph.ca	
ASWA945-08	07PROBE-20277	07PROBE-20277	07PROBE-20277	657	GU141298			07PROBE-20277	Arthropoda	Insecta	Hymenoptera	Braconidae	Microgastrinae	Microplitis	Microplitis varicolor	Jose Leonardo Fernandez	jftriana@uoguelph.ca	
ASWA946-08	07PROBE-20278	07PROBE-20278	07PROBE-20278	631	GU141240			07PROBE-20278	Arthropoda	Insecta	Hymenoptera	Braconidae	Microgastrinae	Microgaster	Microgaster jft06	Jose Leonardo Fernandez	jftriana@uoguelph.ca	
ASWA947-08	07PROBE-20279	07PROBE-20279	07PROBE-20279	646	GU141082			07PROBE-20279	Arthropoda	Insecta	Hymenoptera	Braconidae	Microgastrinae	Cotesia	Cotesia jft04	Jose Leonardo Fernandez	jftriana@uoguelph.ca	
ASWA948-08	07PROBE-20280	07PROBE-20280	07PROBE-20280	631	GU141221			07PROBE-20280	Arthropoda	Insecta	Hymenoptera	Braconidae	Microgastrinae	Glyptapanteles	Glyptapanteles jft08	Jose Leonardo Fernandez	jftriana@uoguelph.ca	
ASWA949-08	07PROBE-20281	07PROBE-20281	07PROBE-20281	657	GU141109			07PROBE-20281	Arthropoda	Insecta	Hymenoptera	Braconidae	Microgastrinae	Cotesia	Cotesia jft08	Jose Leonardo Fernandez	jftriana@uoguelph.ca	
ASWA950-08	07PROBE-20282	07PROBE-20282	07PROBE-20282	631	GU141108			07PROBE-20282	Arthropoda	Insecta	Hymenoptera	Braconidae	Microgastrinae	Cotesia	Cotesia jft08	Jose Leonardo Fernandez	jftriana@uoguelph.ca	
ASWA970-08	07PROBE-20302	07PROBE-20302	07PROBE-20302	657	GU141282			07PROBE-20302	Arthropoda	Insecta	Hymenoptera	Braconidae	Microgastrinae	Microplitis	Microplitis jft08	Jose Leonardo Fernandez	jftriana@uoguelph.ca	
ASWA983-08	07PROBE-20315	07PROBE-20315	07PROBE-20315	657	GU141281			07PROBE-20315	Arthropoda	Insecta	Hymenoptera	Braconidae	Microgastrinae	Microplitis	Microplitis jft08	Jose Leonardo Fernandez	jftriana@uoguelph.ca	
ASWAS973-07	HYM00000774	HYM00000774	HYM00000774	289	GU141245			HYM00000774	Arthropoda	Insecta	Hymenoptera	Braconidae	Microgastrinae	Microplitis	Microplitis jft01	Jose Leonardo Fernandez	jftriana@uoguelph.ca	
ASWAS993-07	HYM00000794	HYM00000794	HYM00000794	0		0		HYM00000794	Arthropoda	Insecta	Hymenoptera	Braconidae	Microgastrinae	Pholetesor	Pholetesor viminetorum	Jose Leonardo Fernandez	jftriana@uoguelph.ca	
ASWAS994-07	HYM00000795	HYM00000795	HYM00000795	0		0		HYM00000795	Arthropoda	Insecta	Hymenoptera	Braconidae	Microgastrinae	Pholetesor	Pholetesor viminetorum	Jose Leonardo Fernandez	jftriana@uoguelph.ca	
ASWAS995-07	HYM00000796	HYM00000796	HYM00000796	0		0		HYM00000796	Arthropoda	Insecta	Hymenoptera	Braconidae	Microgastrinae	Pholetesor	Pholetesor viminetorum	Jose Leonardo Fernandez	jftriana@uoguelph.ca	
ASWAS996-07	HYM00000797	HYM00000797	HYM00000797	0		0		HYM00000797	Arthropoda	Insecta	Hymenoptera	Braconidae	Microgastrinae	Pholetesor	Pholetesor viminetorum	Jose Leonardo Fernandez	jftriana@uoguelph.ca	
ASWAS997-07	HYM00000798	HYM00000798	HYM00000798	0		0		HYM00000798	Arthropoda	Insecta	Hymenoptera	Braconidae	Microgastrinae	Pholetesor	Pholetesor viminetorum	Jose Leonardo Fernandez	jftriana@uoguelph.ca	
ASWAS998-07	HYM00000799	HYM00000799	HYM00000799	0		0		HYM00000799	Arthropoda	Insecta	Hymenoptera	Braconidae	Microgastrinae	Pholetesor	Pholetesor viminetorum	Jose Leonardo Fernandez	jftriana@uoguelph.ca	
ASWAS999-07	HYM00000800	HYM00000800	HYM00000800	257	GU141375			HYM00000800	Arthropoda	Insecta	Hymenoptera	Braconidae	Microgastrinae	Pholetesor	Pholetesor viminetorum	Jose Leonardo Fernandez	jftriana@uoguelph.ca	
ASWAT338-08	07PROBE-20798	07PROBE-20798	07PROBE-20798	0		0		07PROBE-20798	Arthropoda	Insecta	Hymenoptera	Braconidae	Microgastrinae	Apanteles	Apanteles jft02	Jose Leonardo Fernandez	jftriana@uoguelph.ca	
ASWAT756-08	07PROBE-24224	07PROBE-24224	07PROBE-24224	657	GU141237			07PROBE-24224	Arthropoda	Insecta	Hymenoptera	Braconidae	Microgastrinae	Microgaster	Microgaster jft05	Jose Leonardo Fernandez	jftriana@uoguelph.ca	
ASWAT757-08	07PROBE-24225	07PROBE-24225	07PROBE-24225	657	GU141244			07PROBE-24225	Arthropoda	Insecta	Hymenoptera	Braconidae	Microgastrinae	Microgaster	Microgaster jft08	Jose Leonardo Fernandez	jftriana@uoguelph.ca	
ASWAT758-08	07PROBE-24226	07PROBE-24226	07PROBE-24226	657	GU141236			07PROBE-24226	Arthropoda	Insecta	Hymenoptera	Braconidae	Microgastrinae	Microgaster	Microgaster jft05	Jose Leonardo Fernandez	jftriana@uoguelph.ca	
ASWAT760-08	07PROBE-24228	07PROBE-24228	07PROBE-24228	657	GU141081			07PROBE-24228	Arthropoda	Insecta	Hymenoptera	Braconidae	Microgastrinae	Cotesia	Cotesia jft04	Jose Leonardo Fernandez	jftriana@uoguelph.ca	
ASWAT761-08	07PROBE-24229	07PROBE-24229	07PROBE-24229	657	GU141101			07PROBE-24229	Arthropoda	Insecta	Hymenoptera	Braconidae	Microgastrinae	Cotesia	Cotesia jft07	Jose Leonardo Fernandez	jftriana@uoguelph.ca	
ASWAT762-08	07PROBE-24230	07PROBE-24230	07PROBE-24230	657	GU141107			07PROBE-24230	Arthropoda	Insecta	Hymenoptera	Braconidae	Microgastrinae	Cotesia	Cotesia jft08	Jose Leonardo Fernandez	jftriana@uoguelph.ca	
ASWAT763-08	07PROBE-24231	07PROBE-24231	07PROBE-24231	657	GU141287			07PROBE-24231	Arthropoda	Insecta	Hymenoptera	Braconidae	Microgastrinae	Microplitis	Microplitis jft10	Jose Leonardo Fernandez	jftriana@uoguelph.ca	
ASWAT764-08	07PROBE-24232	07PROBE-24232	07PROBE-24232	657	GU141297			07PROBE-24232	Arthropoda	Insecta	Hymenoptera	Braconidae	Microgastrinae	Microplitis	Microplitis varicolor	Jose Leonardo Fernandez	jftriana@uoguelph.ca	
ASWAT766-08	07PROBE-24234	07PROBE-24234	07PROBE-24234	657	GU141175			07PROBE-24234	Arthropoda	Insecta	Hymenoptera	Braconidae	Microgastrinae	Glyptapanteles	Glyptapanteles jft01	Jose Leonardo Fernandez	jftriana@uoguelph.ca	
ASWAT767-08	07PROBE-24235	07PROBE-24235	07PROBE-24235	631	GU141352			07PROBE-24235	Arthropoda	Insecta	Hymenoptera	Braconidae	Microgastrinae	Pholetesor	Pholetesor ornigis	Jose Leonardo Fernandez	jftriana@uoguelph.ca	
ASWAT768-08	07PROBE-24236	07PROBE-24236	07PROBE-24236	657	GU141365			07PROBE-24236	Arthropoda	Insecta	Hymenoptera	Braconidae	Microgastrinae	Pholetesor	Pholetesor salicifoliellae	Jose Leonardo Fernandez	jftriana@uoguelph.ca	
ASWAT769-08	07PROBE-24237	07PROBE-24237	07PROBE-24237	613	GU141351			07PROBE-24237	Arthropoda	Insecta	Hymenoptera	Braconidae	Microgastrinae	Pholetesor	Pholetesor ornigis	Jose Leonardo Fernandez	jftriana@uoguelph.ca	
ASWAT770-08	07PROBE-24238	07PROBE-24238	07PROBE-24238	631	GU141364			07PROBE-24238	Arthropoda	Insecta	Hymenoptera	Braconidae	Microgastrinae	Pholetesor	Pholetesor salicifoliellae	Jose Leonardo Fernandez	jftriana@uoguelph.ca	
ASWAT772-08	07PROBE-24240	07PROBE-24240	07PROBE-24240	631	GU141350			07PROBE-24240	Arthropoda	Insecta	Hymenoptera	Braconidae	Microgastrinae	Pholetesor	Pholetesor ornigis	Jose Leonardo Fernandez	jftriana@uoguelph.ca	
ASWAT773-08	07PROBE-24241	07PROBE-24241	07PROBE-24241	657	GU141349			07PROBE-24241	Arthropoda	Insecta	Hymenoptera	Braconidae	Microgastrinae	Pholetesor	Pholetesor ornigis	Jose Leonardo Fernandez	jftriana@uoguelph.ca	
ASWAT774-08	07PROBE-24242	07PROBE-24242	07PROBE-24242	657	GU141348			07PROBE-24242	Arthropoda	Insecta	Hymenoptera	Braconidae	Microgastrinae	Pholetesor	Pholetesor ornigis	Jose Leonardo Fernandez	jftriana@uoguelph.ca	
ASWAT775-08	07PROBE-24243	07PROBE-24243	07PROBE-24243	626	GU141347			07PROBE-24243	Arthropoda	Insecta	Hymenoptera	Braconidae	Microgastrinae	Pholetesor	Pholetesor ornigis	Jose Leonardo Fernandez	jftriana@uoguelph.ca	
ASWAT776-08	07PROBE-24244	07PROBE-24244	07PROBE-24244	633	GU141346			07PROBE-24244	Arthropoda	Insecta	Hymenoptera	Braconidae	Microgastrinae	Pholetesor	Pholetesor ornigis	Jose Leonardo Fernandez	jftriana@uoguelph.ca	
ASWAT777-08	07PROBE-24245	07PROBE-24245	07PROBE-24245	623	GU141345			07PROBE-24245	Arthropoda	Insecta	Hymenoptera	Braconidae	Microgastrinae	Pholetesor	Pholetesor ornigis	Jose Leonardo Fernandez	jftriana@uoguelph.ca	
ASWAT779-08	07PROBE-24247	07PROBE-24247	07PROBE-24247	623	GU141344			07PROBE-24247	Arthropoda	Insecta	Hymenoptera	Braconidae	Microgastrinae	Pholetesor	Pholetesor ornigis	Jose Leonardo Fernandez	jftriana@uoguelph.ca	
ASWAT781-08	07PROBE-24249	07PROBE-24249	07PROBE-24249	625	GU141356			07PROBE-24249	Arthropoda	Insecta	Hymenoptera	Braconidae	Microgastrinae	Pholetesor	Pholetesor ornigis	Jose Leonardo Fernandez	jftriana@uoguelph.ca	
ASWAT782-08	07PROBE-24250	07PROBE-24250	07PROBE-24250	596	GU141373			07PROBE-24250	Arthropoda	Insecta	Hymenoptera	Braconidae	Microgastrinae	Pholetesor	Pholetesor salicifoliellae	Jose Leonardo Fernandez	jftriana@uoguelph.ca	
ASWAU194-08	HYM00001566	HYM00001566	HYM00001566	657				HYM00001566	Arthropoda	Insecta	Hymenoptera	Braconidae	Microgastrinae	Protapanteles	Protapanteles jft01	Jose Leonardo Fernandez	jftriana@uoguelph.ca	
ASWAU195-08	HYM00001567	HYM00001567	HYM00001567	619				HYM00001567	Arthropoda	Insecta	Hymenoptera	Braconidae	Microgastrinae	Cotesia	Cotesia jft08	Jose Leonardo Fernandez	jftriana@uoguelph.ca	
ASWAU196-08	HYM00001568	HYM00001568	HYM00001568	616				HYM00001568	Arthropoda	Insecta	Hymenoptera	Braconidae	Microgastrinae	Cotesia	Cotesia jft02	Jose Leonardo Fernandez	jftriana@uoguelph.ca	
ASWAU213-08	HYM00001585	HYM00001585	HYM00001585	657				HYM00001585	Arthropoda	Insecta	Hymenoptera	Braconidae	Microgastrinae	Cotesia	Cotesia jft02	Jose Leonardo Fernandez	jftriana@uoguelph.ca	
ASWAU214-08	HYM00001586	HYM00001586	HYM00001586	623				HYM00001586	Arthropoda	Insecta	Hymenoptera	Braconidae	Microgastrinae	Cotesia	Cotesia jft02	Jose Leonardo Fernandez	jftriana@uoguelph.ca	
ASWAU225-08	HYM00001597	HYM00001597	HYM00001597	657				HYM00001597	Arthropoda	Insecta	Hymenoptera	Braconidae	Microgastrinae	Cotesia	Cotesia jft02	Jose Leonardo Fernandez	jftriana@uoguelph.ca	
ASWAU230-08	HYM00001602	HYM00001602	HYM00001602	657				HYM00001602	Arthropoda	Insecta	Hymenoptera	Braconidae	Microgastrinae	Cotesia	Cotesia jft08	Jose Leonardo Fernandez	jftriana@uoguelph.ca	
ASWAU251-08	HYM00001623	HYM00001623	HYM00001623	657				HYM00001623	Arthropoda	Insecta	Hymenoptera	Braconidae	Microgastrinae	Cotesia	Cotesia jft02	Jose Leonardo Fernandez	jftriana@uoguelph.ca	
ASWAU254-08	HYM00001626	HYM00001626	HYM00001626	574				HYM00001626	Arthropoda	Insecta	Hymenoptera	Braconidae	Microgastrinae	Cotesia	Cotesia jft08	Jose Leonardo Fernandez	jftriana@uoguelph.ca	
ASWAU255-08	HYM00001627	HYM00001627	HYM00001627	596				HYM00001627	Arthropoda	Insecta	Hymenoptera	Braconidae	Microgastrinae	Cotesia	Cotesia jft02	Jose Leonardo Fernandez	jftriana@uoguelph.ca	
ASWAU258-08	HYM00001630	HYM00001630	HYM00001630	657				HYM00001630	Arthropoda	Insecta	Hymenoptera	Braconidae	Microgastrinae	Cotesia	Cotesia jft08	Jose Leonardo Fernandez	jftriana@uoguelph.ca	
ASWAU283-08	HYM00001655	HYM00001655	HYM00001655	566				HYM00001655	Arthropoda	Insecta	Hymenoptera	Braconidae	Microgastrinae	Microgaster	Microgaster jft03	Jose Leonardo Fernandez	jftriana@uoguelph.ca	
ASWAU284-08	HYM00001656	HYM00001656	HYM00001656	269				HYM00001656	Arthropoda	Insecta	Hymenoptera	Braconidae	Microgastrinae	Microgaster	Microgaster jft03	Jose Leonardo Fernandez	jftriana@uoguelph.ca	
ASWAU285-08	HYM00001657	HYM00001657	HYM00001657	576				HYM00001657	Arthropoda	Insecta	Hymenoptera	Braconidae	Microgastrinae	Microgaster	Microgaster jft03	Jose Leonardo Fernandez	jftriana@uoguelph.ca	
ASWAU288-08	HYM00001660	HYM00001660	HYM00001660	257				HYM00001660	Arthropoda	Insecta	Hymenoptera	Braconidae	Microgastrinae	Microgaster	Microgaster jft10	Jose Leonardo Fernandez	jftriana@uoguelph.ca	
ASWAU291-08	HYM00001663	HYM00001663	HYM00001663	283				HYM00001663	Arthropoda	Insecta	Hymenoptera	Braconidae	Microgastrinae	Microgaster	Microgaster jft05	Jose Leonardo Fernandez	jftriana@uoguelph.ca	
ASWAU293-08	HYM00001665	HYM00001665	HYM00001665	547				HYM00001665	Arthropoda	Insecta	Hymenoptera	Braconidae	Microgastrinae	Microgaster	Microgaster jft10	Jose Leonardo Fernandez	jftriana@uoguelph.ca	
ASWAU294-08	HYM00001666	HYM00001666	HYM00001666	595				HYM00001666	Arthropoda	Insecta	Hymenoptera	Braconidae	Microgastrinae	Microgaster	Microgaster jft03	Jose Leonardo Fernandez	jftriana@uoguelph.ca	
ASWAU295-08	HYM00001667	HYM00001667	HYM00001667	549				HYM00001667	Arthropoda	Insecta	Hymenoptera	Braconidae	Microgastrinae	Microgaster	Microgaster jft03	Jose Leonardo Fernandez	jftriana@uoguelph.ca	
ASWAU310-08	HYM00001682	HYM00001682	HYM00001682	287				HYM00001682	Arthropoda	Insecta	Hymenoptera	Braconidae	Microgastrinae	Pholetesor	Pholetesor viminetorum	Jose Leonardo Fernandez	jftriana@uoguelph.ca	
ASWAU312-08	HYM00001684	HYM00001684	HYM00001684	567				HYM00001684	Arthropoda	Insecta	Hymenoptera	Braconidae	Microgastrinae	Dolichogenidea	Dolichogenidea jft03	Jose Leonardo Fernandez	jftriana@uoguelph.ca	
ASWAU314-08	HYM00001686	HYM00001686	HYM00001686	574				HYM00001686	Arthropoda	Insecta	Hymenoptera	Braconidae	Microgastrinae	Dolichogenidea	Dolichogenidea jft03	Jose Leonardo Fernandez	jftriana@uoguelph.ca	
ASWAU315-08	HYM00001687	HYM00001687	HYM00001687	552				HYM00001687	Arthropoda	Insecta	Hymenoptera	Braconidae	Microgastrinae	Dolichogenidea	Dolichogenidea jft02	Jose Leonardo Fernandez	jftriana@uoguelph.ca	
ASWAU323-08	HYM00001695	HYM00001695	HYM00001695	570				HYM00001695	Arthropoda	Insecta	Hymenoptera	Braconidae	Microgastrinae	Dolichogenidea	Dolichogenidea jft03	Jose Leonardo Fernandez	jftriana@uoguelph.ca	
ASWAU328-08	HYM00001700	HYM00001700	HYM00001700	574				HYM00001700	Arthropoda	Insecta	Hymenoptera	Braconidae	Microgastrinae	Dolichogenidea	Dolichogenidea jft03	Jose Leonardo Fernandez	jftriana@uoguelph.ca	
ASWAU332-08	HYM00001704	HYM00001704	HYM00001704	560				HYM00001704	Arthropoda	Insecta	Hymenoptera	Braconidae	Microgastrinae	Dolichogenidea	Dolichogenidea jft03	Jose Leonardo Fernandez	jftriana@uoguelph.ca	
ASWAU333-08	HYM00001705	HYM00001705	HYM00001705	585				HYM00001705	Arthropoda	Insecta	Hymenoptera	Braconidae	Microgastrinae	Dolichogenidea	Dolichogenidea jft03	Jose Leonardo Fernandez	jftriana@uoguelph.ca	
ASWAU334-08	HYM00001706	HYM00001706	HYM00001706	595				HYM00001706	Arthropoda	Insecta	Hymenoptera	Braconidae	Microgastrinae	Dolichogenidea	Dolichogenidea jft02	Jose Leonardo Fernandez	jftriana@uoguelph.ca	
ASWAU337-08	HYM00001709	HYM00001709	HYM00001709	547				HYM00001709	Arthropoda	Insecta	Hymenoptera	Braconidae	Microgastrinae	Dolichogenidea	Dolichogenidea jft03	Jose Leonardo Fernandez	jftriana@uoguelph.ca	
ASWAU338-08	HYM00001710	HYM00001710		271				HYM00001710	Arthropoda	Insecta	Hymenoptera	Braconidae	Microgastrinae	Dolichogenidea	Dolichogenidea jft06	Jose Leonardo Fernandez	jftriana@uoguelph.ca	
ASWAU340-08	HYM00001712	HYM00001712	HYM00001712	534				HYM00001712	Arthropoda	Insecta	Hymenoptera	Braconidae	Microgastrinae	Dolichogenidea	Dolichogenidea jft03	Jose Leonardo Fernandez	jftriana@uoguelph.ca	
ASWAU343-08	HYM00001715	HYM00001715	HYM00001715	556				HYM00001715	Arthropoda	Insecta	Hymenoptera	Braconidae	Microgastrinae	Dolichogenidea	Dolichogenidea jft03	Jose Leonardo Fernandez	jftriana@uoguelph.ca	
ASWAU344-08	HYM00001716	HYM00001716	HYM00001716	575				HYM00001716	Arthropoda	Insecta	Hymenoptera	Braconidae	Microgastrinae	Dolichogenidea	Dolichogenidea jft03	Jose Leonardo Fernandez	jftriana@uoguelph.ca	
ASWAU345-08	HYM00001717	HYM00001717	HYM00001717	547				HYM00001717	Arthropoda	Insecta	Hymenoptera	Braconidae	Microgastrinae	Dolichogenidea	Dolichogenidea jft06	Jose Leonardo Fernandez	jftriana@uoguelph.ca	
ASWAU346-08	HYM00001718	HYM00001718	HYM00001718	556				HYM00001718	Arthropoda	Insecta	Hymenoptera	Braconidae	Microgastrinae	Dolichogenidea	Dolichogenidea jft03	Jose Leonardo Fernandez	jftriana@uoguelph.ca	
ASWAU351-08	HYM00001723	HYM00001723	HYM00001723	559				HYM00001723	Arthropoda	Insecta	Hymenoptera	Braconidae	Microgastrinae	Dolichogenidea	Dolichogenidea jft03	Jose Leonardo Fernandez	jftriana@uoguelph.ca	
ASWAU354-08	HYM00001726	HYM00001726	HYM00001726	546				HYM00001726	Arthropoda	Insecta	Hymenoptera	Braconidae	Microgastrinae	Microgaster	Microgaster jft03	Jose Leonardo Fernandez	jftriana@uoguelph.ca	
ASWAU357-08	HYM00001729	HYM00001729	HYM00001729	562				HYM00001729	Arthropoda	Insecta	Hymenoptera	Braconidae	Microgastrinae	Microgaster	Microgaster jft03	Jose Leonardo Fernandez	jftriana@uoguelph.ca	
ASWAU358-08	HYM00001730	HYM00001730	HYM00001730	612				HYM00001730	Arthropoda	Insecta	Hymenoptera	Braconidae	Microgastrinae	Microgaster	Microgaster jft03	Jose Leonardo Fernandez	jftriana@uoguelph.ca	
ASWAU361-08	HYM00001733	HYM00001733	HYM00001733	603				HYM00001733	Arthropoda	Insecta	Hymenoptera	Braconidae	Microgastrinae	Microgaster	Microgaster jft03	Jose Leonardo Fernandez	jftriana@uoguelph.ca	
ASWAU363-08	HYM00001735	HYM00001735	HYM00001735	556				HYM00001735	Arthropoda	Insecta	Hymenoptera	Braconidae	Microgastrinae	Microgaster	Microgaster jft03	Jose Leonardo Fernandez	jftriana@uoguelph.ca	
ASWAU364-08	HYM00001736	HYM00001736	HYM00001736	571				HYM00001736	Arthropoda	Insecta	Hymenoptera	Braconidae	Microgastrinae	Microgaster	Microgaster jft03	Jose Leonardo Fernandez	jftriana@uoguelph.ca	
ASWAU365-08	HYM00001737	HYM00001737	HYM00001737	257				HYM00001737	Arthropoda	Insecta	Hymenoptera	Braconidae	Microgastrinae	Microgaster	Microgaster jft03	Jose Leonardo Fernandez	jftriana@uoguelph.ca	
ASWAU366-08	HYM00001738	HYM00001738	HYM00001738	604				HYM00001738	Arthropoda	Insecta	Hymenoptera	Braconidae	Microgastrinae	Microgaster	Microgaster jft03	Jose Leonardo Fernandez	jftriana@uoguelph.ca	
ASWAU367-08	HYM00001739	HYM00001739	HYM00001739	594				HYM00001739	Arthropoda	Insecta	Hymenoptera	Braconidae	Microgastrinae	Microgaster	Microgaster jft03	Jose Leonardo Fernandez	jftriana@uoguelph.ca	
ASWAU368-08	HYM00001740	HYM00001740	HYM00001740	603				HYM00001740	Arthropoda	Insecta	Hymenoptera	Braconidae	Microgastrinae	Microgaster	Microgaster jft03	Jose Leonardo Fernandez	jftriana@uoguelph.ca	
ASWAU369-08	HYM00001741	HYM00001741	HYM00001741	579				HYM00001741	Arthropoda	Insecta	Hymenoptera	Braconidae	Microgastrinae	Microgaster	Microgaster jft03	Jose Leonardo Fernandez	jftriana@uoguelph.ca	
ASWAU370-08	HYM00001742	HYM00001742	HYM00001742	574				HYM00001742	Arthropoda	Insecta	Hymenoptera	Braconidae	Microgastrinae	Microgaster	Microgaster jft03	Jose Leonardo Fernandez	jftriana@uoguelph.ca	
ASWAU371-08	HYM00001743	HYM00001743	HYM00001743	577				HYM00001743	Arthropoda	Insecta	Hymenoptera	Braconidae	Microgastrinae	Microgaster	Microgaster jft03	Jose Leonardo Fernandez	jftriana@uoguelph.ca	
ASWAU372-08	HYM00001744	HYM00001744	HYM00001744	565				HYM00001744	Arthropoda	Insecta	Hymenoptera	Braconidae	Microgastrinae	Microgaster	Microgaster jft03	Jose Leonardo Fernandez	jftriana@uoguelph.ca	
ASWAU373-08	HYM00001745	HYM00001745	HYM00001745	570				HYM00001745	Arthropoda	Insecta	Hymenoptera	Braconidae	Microgastrinae	Microgaster	Microgaster jft03	Jose Leonardo Fernandez	jftriana@uoguelph.ca	
ASWAU374-08	HYM00001746	HYM00001746	HYM00001746	256				HYM00001746	Arthropoda	Insecta	Hymenoptera	Braconidae	Microgastrinae	Microgaster	Microgaster jft03	Jose Leonardo Fernandez	jftriana@uoguelph.ca	
ASWAU375-08	HYM00001747	HYM00001747	HYM00001747	570				HYM00001747	Arthropoda	Insecta	Hymenoptera	Braconidae	Microgastrinae	Microgaster	Microgaster jft03	Jose Leonardo Fernandez	jftriana@uoguelph.ca	
ASWAU376-08	HYM00001748	HYM00001748	HYM00001748	543				HYM00001748	Arthropoda	Insecta	Hymenoptera	Braconidae	Microgastrinae	Microgaster	Microgaster jft03	Jose Leonardo Fernandez	jftriana@uoguelph.ca	
ASWAU491-08	HYM00001769	HYM00001769		657				HYM00001769	Arthropoda	Insecta	Hymenoptera	Braconidae	Microgastrinae	Dolichogenidea	Dolichogenidea jft03	Jose Leonardo Fernandez	jftriana@uoguelph.ca	
ASWAU498-08	HYM00001776	HYM00001776		622				HYM00001776	Arthropoda	Insecta	Hymenoptera	Braconidae	Microgastrinae	Dolichogenidea	Dolichogenidea jft02	Jose Leonardo Fernandez	jftriana@uoguelph.ca	
ASWAV855-08	07PROBE-22314	07PROBE-22314	07PROBE-22314	649	GU141383			07PROBE-22314	Arthropoda	Insecta	Hymenoptera	Braconidae	Microgastrinae	Protapanteles	Protapanteles jft02	Jose Leonardo Fernandez	jftriana@uoguelph.ca	

ProcessID	Reproduction	Life Stage	Extra Info	Notes	Voucher Type	Tissue Type	Collectors	Date Collected	Continent	Country/Ocean	Province	Region	Sector	Exact Site	Lat	Lon	Elev	GPS Source
ASCNC014-09							N. Islam	10-Jul-2007		Canada	Ontario	Cochrane		Cochrane & Kapuscani	49.3981018	-80.472198		
ASCNC015-09							N. Islam	10-Jul-2007		Canada	Ontario	Cochrane		Cochrane & Kapuscani	49.3981018	-80.472198		
ASCNC016-09							N. Islam	15-Jun-2007		Canada	Ontario	Cochrane		Cochrane & Kapuscani	49.3981018	-80.472198		
ASCNC021-09							N. Islam	09-Jul-2007		Canada	Ontario	Cochrane		Cochrane & Kapuscani	49.3981018	-80.472198		
ASWA1026-08				<VERBATIM LOCALITY>|A-Frame, 12 Km E of			A.Renaud	17-Jul-2007		Canada	Manitoba	Churchill	10 km ESE Churchill, L		58.7550011	-93.998001		
ASWA1027-08				<VERBATIM LOCALITY>|A-Frame, 12 Km E of			A.Renaud	17-Jul-2007		Canada	Manitoba	Churchill	10 km ESE Churchill, L		58.7550011	-93.998001		
ASWA1028-08				<VERBATIM LOCALITY>|A-Frame, 12 Km E of			A.Renaud	17-Jul-2007		Canada	Manitoba	Churchill	10 km ESE Churchill, L		58.7550011	-93.998001		
ASWA1029-08				<VERBATIM LOCALITY>|A-Frame, 12 Km E of			A.Renaud	17-Jul-2007		Canada	Manitoba	Churchill	10 km ESE Churchill, L		58.7550011	-93.998001		
ASWA1031-08				<VERBATIM LOCALITY>|A-Frame, 12 Km E of			A.Renaud	17-Jul-2007		Canada	Manitoba	Churchill	10 km ESE Churchill, L		58.7550011	-93.998001		
ASWA1032-08				<VERBATIM LOCALITY>|A-Frame, 12 Km E of			A.Renaud	17-Jul-2007		Canada	Manitoba	Churchill	10 km ESE Churchill, L		58.7550011	-93.998001		
ASWA1033-08				<VERBATIM LOCALITY>|A-Frame, 12 Km E of			A.Renaud	17-Jul-2007		Canada	Manitoba	Churchill	10 km ESE Churchill, L		58.7550011	-93.998001		
ASWA1034-08				<VERBATIM LOCALITY>|A-Frame, 12 Km E of			A.Renaud	17-Jul-2007		Canada	Manitoba	Churchill	10 km ESE Churchill, L		58.7550011	-93.998001		
ASWA1035-08				<VERBATIM LOCALITY>|A-Frame, 12 Km E of			A.Renaud	17-Jul-2007		Canada	Manitoba	Churchill	10 km ESE Churchill, L		58.7550011	-93.998001		
ASWA1036-08				<VERBATIM LOCALITY>|A-Frame, 12 Km E of						Canada	Manitoba	Churchill	10 km ESE Churchill, L		58.7550011	-93.998001		
ASWA1070-08				<VERBATIM LOCALITY>|Twin Lakes, 22 km SE			A.Renaud	07-Jul-2007		Canada	Manitoba	Churchill	26 km SE Churchill, Tw		58.632	-93.788002		

Table S5: All collection and accession information associated with specimens analysed here.

ProcessID	Sample ID	Field Num	Catalog Num	COI-5P Seq.	COI GenBank	28S Seq. Length	28S GenBan	Sample ID	Phylum	Class	Order	Family	Subfamily	Genus	Species	Identifier	Identifier email	Sex	Reproduction	Life Stage	Extra Info	Notes	Voucher Type	Tissue Type	Collectors	Date Collected	Continent	Country/Ocean	Province	Region	Sector	Exact Site	Lat	Lon	Elev	GPS Source
ASWAV856-08	07PROBE-22315	07PROBE-22315	07PROBE-22315	656	GU141382			07PROBE-22315	Arthropoda	Insecta	Hymenoptera	Braconidae	Microgastrinae	Protapanteles	Protapanteles jft02	Jose Leonardo Fernandez	jftriana@uoguelph.ca					<VERBATIM LOCALITY>|Pumphouse|58.373|-9			A.Renaud	12-Jul-2007		Canada	Manitoba	Churchill	16 km SW Churchill, Ch		58.6259995	-94.228996		
ASWAV857-08	07PROBE-22316	07PROBE-22316	07PROBE-22316	614	GU141381			07PROBE-22316	Arthropoda	Insecta	Hymenoptera	Braconidae	Microgastrinae	Protapanteles	Protapanteles jft02	Jose Leonardo Fernandez	jftriana@uoguelph.ca					<VERBATIM LOCALITY>|Pumphouse|58.373|-9			A.Renaud	12-Jul-2007		Canada	Manitoba	Churchill	16 km SW Churchill, Ch		58.6259995	-94.228996		
ASWAV858-08	07PROBE-22317	07PROBE-22317	07PROBE-22317	629	GU141288			07PROBE-22317	Arthropoda	Insecta	Hymenoptera	Braconidae	Microgastrinae	Microplitis	Microplitis jft10	Jose Leonardo Fernandez	jftriana@uoguelph.ca					<VERBATIM LOCALITY>|Pumphouse|58.373|-9			A.Renaud	12-Jul-2007		Canada	Manitoba	Churchill	16 km SW Churchill, Ch		58.6259995	-94.228996		
ASWAV860-08	07PROBE-22319	07PROBE-22319	07PROBE-22319	646	GU141057			07PROBE-22319	Arthropoda	Insecta	Hymenoptera	Braconidae	Microgastrinae	Apanteles	Apanteles jft01	Jose Leonardo Fernandez	jftriana@uoguelph.ca					<VERBATIM LOCALITY>|Pumphouse|58.373|-9			A.Renaud	12-Jul-2007		Canada	Manitoba	Churchill	16 km SW Churchill, Ch		58.6259995	-94.228996		
ASWAV861-08	07PROBE-22320	07PROBE-22320	07PROBE-22320	656	GU141114			07PROBE-22320	Arthropoda	Insecta	Hymenoptera	Braconidae	Microgastrinae	Cotesia	Cotesia jft08	Jose Leonardo Fernandez	jftriana@uoguelph.ca					<VERBATIM LOCALITY>|Pumphouse|58.373|-9			A.Renaud	12-Jul-2007		Canada	Manitoba	Churchill	16 km SW Churchill, Ch		58.6259995	-94.228996		
ASWAV862-08	07PROBE-22321	07PROBE-22321	07PROBE-22321	634	GU141075			07PROBE-22321	Arthropoda	Insecta	Hymenoptera	Braconidae	Microgastrinae	Apanteles	Apanteles jft03	Jose Leonardo Fernandez	jftriana@uoguelph.ca					<VERBATIM LOCALITY>|Pumphouse|58.373|-9	NULL		A.Renaud	12-Jul-2007		Canada	Manitoba	Churchill	16 km SW Churchill, Ch		58.6259995	-94.228996		
ASWAV863-08	07PROBE-22322	07PROBE-22322	07PROBE-22322	656	GU141136			07PROBE-22322	Arthropoda	Insecta	Hymenoptera	Braconidae	Microgastrinae	Cotesia	Cotesia xylina	Jose Leonardo Fernandez	jftriana@uoguelph.ca					<VERBATIM LOCALITY>|Pumphouse|58.373|-9			A.Renaud	12-Jul-2007		Canada	Manitoba	Churchill	16 km SW Churchill, Ch		58.6259995	-94.228996		
ASWAV864-08	07PROBE-22323	07PROBE-22323	07PROBE-22323	626	GU141135			07PROBE-22323	Arthropoda	Insecta	Hymenoptera	Braconidae	Microgastrinae	Cotesia	Cotesia xylina	Jose Leonardo Fernandez	jftriana@uoguelph.ca					<VERBATIM LOCALITY>|Pumphouse|58.373|-9			A.Renaud	12-Jul-2007		Canada	Manitoba	Churchill	16 km SW Churchill, Ch		58.6259995	-94.228996		
ASWAV865-08	07PROBE-22324	07PROBE-22324	07PROBE-22324	631	GU141372			07PROBE-22324	Arthropoda	Insecta	Hymenoptera	Braconidae	Microgastrinae	Pholetesor	Pholetesor salicifoliellae	Jose Leonardo Fernandez	jftriana@uoguelph.ca					<VERBATIM LOCALITY>|Pumphouse|58.373|-9			A.Renaud	12-Jul-2007		Canada	Manitoba	Churchill	16 km SW Churchill, Ch		58.6259995	-94.228996		
ASWAV866-08	07PROBE-22325	07PROBE-22325	07PROBE-22325	626	GU141380			07PROBE-22325	Arthropoda	Insecta	Hymenoptera	Braconidae	Microgastrinae	Protapanteles	Protapanteles jft02	Jose Leonardo Fernandez	jftriana@uoguelph.ca					<VERBATIM LOCALITY>|Ramsay Creek|58.75			A.Renaud	02-Aug-2007		Canada	Manitoba	Churchill	23 km E Churchill, Ram		58.730999	-93.779999	10	
ASWAV867-08	07PROBE-22326	07PROBE-22326	07PROBE-22326	0		0		07PROBE-22326	Arthropoda	Insecta	Hymenoptera	Braconidae	Microgastrinae	Apanteles	Apanteles morrisi	Jose Leonardo Fernandez	jftriana@uoguelph.ca					<VERBATIM LOCALITY>|Pumphouse|58.373|-9			A.Renaud	12-Jul-2007		Canada	Manitoba	Churchill	16 km SW Churchill, Ch		58.6259995	-94.228996		
ASWAV868-08	07PROBE-22327	07PROBE-22327	07PROBE-22327	631	GU141355			07PROBE-22327	Arthropoda	Insecta	Hymenoptera	Braconidae	Microgastrinae	Pholetesor	Pholetesor ornigis	Jose Leonardo Fernandez	jftriana@uoguelph.ca					<VERBATIM LOCALITY>|Ramsay Creek|58.75			A.Renaud	02-Aug-2007		Canada	Manitoba	Churchill	23 km E Churchill, Ram		58.730999	-93.779999	10	
ASWAV869-08	07PROBE-22328	07PROBE-22328	07PROBE-22328	654	GU141047			07PROBE-22328	Arthropoda	Insecta	Hymenoptera	Braconidae	Microgastrinae	Apanteles	Apanteles ensiger	Jose Leonardo Fernandez	jftriana@uoguelph.ca					<VERBATIM LOCALITY>|Ramsay Creek|58.75			A.Renaud	02-Aug-2007		Canada	Manitoba	Churchill	23 km E Churchill, Ram		58.730999	-93.779999	10	
ASWAV870-08	07PROBE-22329	07PROBE-22329	07PROBE-22329	652	GU141274			07PROBE-22329	Arthropoda	Insecta	Hymenoptera	Braconidae	Microgastrinae	Microplitis	Microplitis jft04	Jose Leonardo Fernandez	jftriana@uoguelph.ca					<VERBATIM LOCALITY>|Ramsay Creek|58.75			A.Renaud	02-Aug-2007		Canada	Manitoba	Churchill	23 km E Churchill, Ram		58.730999	-93.779999	10	
ASWAV871-08	07PROBE-22330	07PROBE-22330	07PROBE-22330	630	GU141338			07PROBE-22330	Arthropoda	Insecta	Hymenoptera	Braconidae	Microgastrinae	Pholetesor	Pholetesor jft01	Jose Leonardo Fernandez	jftriana@uoguelph.ca					<VERBATIM LOCALITY>|Ramsay Creek|58.75			A.Renaud	02-Aug-2007		Canada	Manitoba	Churchill	23 km E Churchill, Ram		58.730999	-93.779999	10	
ASWAV872-08	07PROBE-22331	07PROBE-22331	07PROBE-22331	617	GU141379			07PROBE-22331	Arthropoda	Insecta	Hymenoptera	Braconidae	Microgastrinae	Protapanteles	Protapanteles jft02	Jose Leonardo Fernandez	jftriana@uoguelph.ca					<VERBATIM LOCALITY>|Ramsay Creek|58.75			A.Renaud	02-Aug-2007		Canada	Manitoba	Churchill	23 km E Churchill, Ram		58.730999	-93.779999	10	
ASWAV873-08	07PROBE-22332	07PROBE-22332	07PROBE-22332	646	GU141134			07PROBE-22332	Arthropoda	Insecta	Hymenoptera	Braconidae	Microgastrinae	Cotesia	Cotesia xylina	Jose Leonardo Fernandez	jftriana@uoguelph.ca					<VERBATIM LOCALITY>|Ramsay Creek|58.75			A.Renaud	02-Aug-2007		Canada	Manitoba	Churchill	23 km E Churchill, Ram		58.730999	-93.779999	10	
ASWAV874-08	07PROBE-22333	07PROBE-22333	07PROBE-22333	656	GU141056			07PROBE-22333	Arthropoda	Insecta	Hymenoptera	Braconidae	Microgastrinae	Apanteles	Apanteles jft01	Jose Leonardo Fernandez	jftriana@uoguelph.ca					<VERBATIM LOCALITY>|Ramsay Creek|58.75			A.Renaud	02-Aug-2007		Canada	Manitoba	Churchill	23 km E Churchill, Ram		58.730999	-93.779999	10	
ASWAV875-08	07PROBE-22334	07PROBE-22334	07PROBE-22334	0		0		07PROBE-22334	Arthropoda	Insecta	Hymenoptera	Braconidae	Microgastrinae	Protapanteles	Protapanteles jft02	Jose Leonardo Fernandez	jftriana@uoguelph.ca					<VERBATIM LOCALITY>|Ramsay Creek|58.75			A.Renaud	02-Aug-2007		Canada	Manitoba	Churchill	23 km E Churchill, Ram		58.730999	-93.779999	10	
ASWAV876-08	07PROBE-22335	07PROBE-22335	07PROBE-22335	623	GU141074			07PROBE-22335	Arthropoda	Insecta	Hymenoptera	Braconidae	Microgastrinae	Apanteles	Apanteles jft02	Jose Leonardo Fernandez	jftriana@uoguelph.ca					<VERBATIM LOCALITY>|Ramsay Creek|58.75			A.Renaud	02-Aug-2007		Canada	Manitoba	Churchill	23 km E Churchill, Ram		58.730999	-93.779999	10	
ASWAV877-08	07PROBE-22336	07PROBE-22336	07PROBE-22336	614	GU141073			07PROBE-22336	Arthropoda	Insecta	Hymenoptera	Braconidae	Microgastrinae	Apanteles	Apanteles jft02	Jose Leonardo Fernandez	jftriana@uoguelph.ca					<VERBATIM LOCALITY>|Ramsay Creek|58.75			A.Renaud	02-Aug-2007		Canada	Manitoba	Churchill	23 km E Churchill, Ram		58.730999	-93.779999	10	
ASWAV878-08	07PROBE-22337	07PROBE-22337	07PROBE-22337	656	GU141072			07PROBE-22337	Arthropoda	Insecta	Hymenoptera	Braconidae	Microgastrinae	Apanteles	Apanteles jft02	Jose Leonardo Fernandez	jftriana@uoguelph.ca					<VERBATIM LOCALITY>|Ramsay Creek|58.75			A.Renaud	02-Aug-2007		Canada	Manitoba	Churchill	23 km E Churchill, Ram		58.730999	-93.779999	10	
ASWAV879-08	07PROBE-22338	07PROBE-22338	07PROBE-22338	621	GU141100			07PROBE-22338	Arthropoda	Insecta	Hymenoptera	Braconidae	Microgastrinae	Cotesia	Cotesia jft06	Jose Leonardo Fernandez	jftriana@uoguelph.ca					<VERBATIM LOCALITY>|Ramsay Creek|58.75			A.Renaud	02-Aug-2007		Canada	Manitoba	Churchill	23 km E Churchill, Ram		58.730999	-93.779999	10	
ASWAV880-08	07PROBE-22339	07PROBE-22339	07PROBE-22339	623	GU141071			07PROBE-22339	Arthropoda	Insecta	Hymenoptera	Braconidae	Microgastrinae	Apanteles	Apanteles jft02	Jose Leonardo Fernandez	jftriana@uoguelph.ca					<VERBATIM LOCALITY>|Ramsay Creek|58.75			A.Renaud	02-Aug-2007		Canada	Manitoba	Churchill	23 km E Churchill, Ram		58.730999	-93.779999	10	
ASWAV881-08	07PROBE-22340	07PROBE-22340	07PROBE-22340	631	GU141196			07PROBE-22340	Arthropoda	Insecta	Hymenoptera	Braconidae	Microgastrinae	Glyptapanteles	Glyptapanteles jft03	Jose Leonardo Fernandez	jftriana@uoguelph.ca					<VERBATIM LOCALITY>|Twin Lakes, 22 km SE			A.Renaud	11-Aug-2007		Canada	Manitoba	Churchill	26 km SE Churchill, Tw		58.632	-93.788002		
ASWAV882-08	07PROBE-22341	07PROBE-22341	07PROBE-22341	616	GU141332	535	GU141541	07PROBE-22341	Arthropoda	Insecta	Hymenoptera	Braconidae	Microgastrinae	Microplitis	Microplitis varicolor	Jose Leonardo Fernandez	jftriana@uoguelph.ca					<VERBATIM LOCALITY>|Twin Lakes, 22 km SE			A.Renaud	11-Aug-2007		Canada	Manitoba	Churchill	26 km SE Churchill, Tw		58.632	-93.788002		
ASWAV883-08	07PROBE-22342	07PROBE-22342	07PROBE-22342	619	GU141276	525	GU141506	07PROBE-22342	Arthropoda	Insecta	Hymenoptera	Braconidae	Microgastrinae	Microplitis	Microplitis jft06	Jose Leonardo Fernandez	jftriana@uoguelph.ca					<VERBATIM LOCALITY>|Twin Lakes, 22 km SE			A.Renaud	11-Aug-2007		Canada	Manitoba	Churchill	26 km SE Churchill, Tw		58.632	-93.788002		
ASWAV884-08	07PROBE-22343	07PROBE-22343	07PROBE-22343	613	GU141162	536	GU141465	07PROBE-22343	Arthropoda	Insecta	Hymenoptera	Braconidae	Microgastrinae	Dolichogenidea	Dolichogenidea jft03	Jose Leonardo Fernandez	jftriana@uoguelph.ca					<VERBATIM LOCALITY>|Twin Lakes, 22 km SE			A.Renaud	11-Aug-2007		Canada	Manitoba	Churchill	26 km SE Churchill, Tw		58.632	-93.788002		
ASWAV885-08	07PROBE-22344	07PROBE-22344	07PROBE-22344	657	GU141337	536	GU141543	07PROBE-22344	Arthropoda	Insecta	Hymenoptera	Braconidae	Microgastrinae	Pholetesor	Pholetesor jft01	Jose Leonardo Fernandez	jftriana@uoguelph.ca					<VERBATIM LOCALITY>|Twin Lakes, 22 km SE			A.Renaud	11-Aug-2007		Canada	Manitoba	Churchill	26 km SE Churchill, Tw		58.632	-93.788002		
ASWAV886-08	07PROBE-22345	07PROBE-22345	07PROBE-22345	657	GU141161	536	GU141464	07PROBE-22345	Arthropoda	Insecta	Hymenoptera	Braconidae	Microgastrinae	Dolichogenidea	Dolichogenidea jft03	Jose Leonardo Fernandez	jftriana@uoguelph.ca					<VERBATIM LOCALITY>|Twin Lakes, 22 km SE			A.Renaud	11-Aug-2007		Canada	Manitoba	Churchill	26 km SE Churchill, Tw		58.632	-93.788002		
ASWAV887-08	07PROBE-22346	07PROBE-22346	07PROBE-22346	619	GU141331	472	GU141540	07PROBE-22346	Arthropoda	Insecta	Hymenoptera	Braconidae	Microgastrinae	Microplitis	Microplitis varicolor	Jose Leonardo Fernandez	jftriana@uoguelph.ca					<VERBATIM LOCALITY>|Twin Lakes, 22 km SE				11-Aug-2007		Canada	Manitoba	Churchill	26 km SE Churchill, Tw		58.632	-93.788002		
ASWAV888-08	07PROBE-22347	07PROBE-22347	07PROBE-22347	589	GU141127	511	GU141441	07PROBE-22347	Arthropoda	Insecta	Hymenoptera	Braconidae	Microgastrinae	Cotesia	Cotesia jft06	Jose Leonardo Fernandez	jftriana@uoguelph.ca					<VERBATIM LOCALITY>|Twin Lakes, 22 km SE			A.Renaud	11-Aug-2007		Canada	Manitoba	Churchill	26 km SE Churchill, Tw		58.632	-93.788002		
ASWAV889-08	07PROBE-22348	07PROBE-22348	07PROBE-22348	656	GU141330	418	GU141539	07PROBE-22348	Arthropoda	Insecta	Hymenoptera	Braconidae	Microgastrinae	Microplitis	Microplitis varicolor	Jose Leonardo Fernandez	jftriana@uoguelph.ca					<VERBATIM LOCALITY>|Twin Lakes, 22 km SE			A.Renaud	11-Aug-2007		Canada	Manitoba	Churchill	26 km SE Churchill, Tw		58.632	-93.788002		
ASWAV890-08	07PROBE-22349	07PROBE-22349	07PROBE-22349	629	GU141273	509	GU141504	07PROBE-22349	Arthropoda	Insecta	Hymenoptera	Braconidae	Microgastrinae	Microplitis	Microplitis jft04	Jose Leonardo Fernandez	jftriana@uoguelph.ca					<VERBATIM LOCALITY>|Twin Lakes, 22 km SE			A.Renaud	11-Aug-2007		Canada	Manitoba	Churchill	26 km SE Churchill, Tw		58.632	-93.788002		
ASWAV891-08	07PROBE-22350	07PROBE-22350	07PROBE-22350	657	GU141160	536	GU141463	07PROBE-22350	Arthropoda	Insecta	Hymenoptera	Braconidae	Microgastrinae	Dolichogenidea	Dolichogenidea jft03	Jose Leonardo Fernandez	jftriana@uoguelph.ca					<VERBATIM LOCALITY>|Twin Lakes, 22 km SE			A.Renaud	11-Aug-2007		Canada	Manitoba	Churchill	26 km SE Churchill, Tw		58.632	-93.788002		
ASWAV892-08	07PROBE-22351	07PROBE-22351	07PROBE-22351	615	GU141371	379	GU141561	07PROBE-22351	Arthropoda	Insecta	Hymenoptera	Braconidae	Microgastrinae	Pholetesor	Pholetesor salicifoliellae	Jose Leonardo Fernandez	jftriana@uoguelph.ca					<VERBATIM LOCALITY>|Twin Lakes, 22 km SE			A.Renaud	11-Aug-2007		Canada	Manitoba	Churchill	26 km SE Churchill, Tw		58.632	-93.788002		
ASWAV893-08	07PROBE-22352	07PROBE-22352	07PROBE-22352	657	GU141329	536	GU141538	07PROBE-22352	Arthropoda	Insecta	Hymenoptera	Braconidae	Microgastrinae	Microplitis	Microplitis varicolor	Jose Leonardo Fernandez	jftriana@uoguelph.ca					<VERBATIM LOCALITY>|Twin Lakes, 22 km SE			A.Renaud	11-Aug-2007		Canada	Manitoba	Churchill	26 km SE Churchill, Tw		58.632	-93.788002		
ASWAV894-08	07PROBE-22353	07PROBE-22353	07PROBE-22353	657	GU141055	308	GU141392	07PROBE-22353	Arthropoda	Insecta	Hymenoptera	Braconidae	Microgastrinae	Apanteles	Apanteles jft01	Jose Leonardo Fernandez	jftriana@uoguelph.ca					<VERBATIM LOCALITY>|Twin Lakes, 22 km SE			A.Renaud	11-Aug-2007		Canada	Manitoba	Churchill	26 km SE Churchill, Tw		58.632	-93.788002		
ASWAV895-08	07PROBE-22354	07PROBE-22354	07PROBE-22354	620	GU141328	536	GU141537	07PROBE-22354	Arthropoda	Insecta	Hymenoptera	Braconidae	Microgastrinae	Microplitis	Microplitis varicolor	Jose Leonardo Fernandez	jftriana@uoguelph.ca					<VERBATIM LOCALITY>|Twin Lakes, 22 km SE			A.Renaud	11-Aug-2007		Canada	Manitoba	Churchill	26 km SE Churchill, Tw		58.632	-93.788002		
ASWAV896-08	07PROBE-22355	07PROBE-22355	07PROBE-22355	643	GU141327	536	GU141536	07PROBE-22355	Arthropoda	Insecta	Hymenoptera	Braconidae	Microgastrinae	Microplitis	Microplitis varicolor	Jose Leonardo Fernandez	jftriana@uoguelph.ca					<VERBATIM LOCALITY>|Twin Lakes, 22 km SE			A.Renaud	11-Aug-2007		Canada	Manitoba	Churchill	26 km SE Churchill, Tw		58.632	-93.788002		
ASWAV897-08	07PROBE-22356	07PROBE-22356	07PROBE-22356	655	GU141326	536	GU141535	07PROBE-22356	Arthropoda	Insecta	Hymenoptera	Braconidae	Microgastrinae	Microplitis	Microplitis varicolor	Jose Leonardo Fernandez	jftriana@uoguelph.ca					<VERBATIM LOCALITY>|Twin Lakes, 22 km SE			A.Renaud	11-Aug-2007		Canada	Manitoba	Churchill	26 km SE Churchill, Tw		58.632	-93.788002		
ASWAV898-08	07PROBE-22357	07PROBE-22357	07PROBE-22357	657	GU141325	422	GU141534	07PROBE-22357	Arthropoda	Insecta	Hymenoptera	Braconidae	Microgastrinae	Microplitis	Microplitis varicolor	Jose Leonardo Fernandez	jftriana@uoguelph.ca					<VERBATIM LOCALITY>|Twin Lakes, 22 km SE			A.Renaud	11-Aug-2007		Canada	Manitoba	Churchill	26 km SE Churchill, Tw		58.632	-93.788002		
ASWAV899-08	07PROBE-22358	07PROBE-22358	07PROBE-22358	614	GU141324	536	GU141533	07PROBE-22358	Arthropoda	Insecta	Hymenoptera	Braconidae	Microgastrinae	Microplitis	Microplitis varicolor	Jose Leonardo Fernandez	jftriana@uoguelph.ca					<VERBATIM LOCALITY>|Twin Lakes, 22 km SE			A.Renaud	11-Aug-2007		Canada	Manitoba	Churchill	26 km SE Churchill, Tw		58.632	-93.788002		
ASWAV900-08	07PROBE-22359	07PROBE-22359	07PROBE-22359	615	GU141323	536	GU141532	07PROBE-22359	Arthropoda	Insecta	Hymenoptera	Braconidae	Microgastrinae	Microplitis	Microplitis varicolor	Jose Leonardo Fernandez	jftriana@uoguelph.ca					<VERBATIM LOCALITY>|Twin Lakes, 22 km SE			A.Renaud	11-Aug-2007		Canada	Manitoba	Churchill	26 km SE Churchill, Tw		58.632	-93.788002		
ASWAV901-08	07PROBE-22360	07PROBE-22360	07PROBE-22360	619	GU141322	536	GU141531	07PROBE-22360	Arthropoda	Insecta	Hymenoptera	Braconidae	Microgastrinae	Microplitis	Microplitis varicolor	Jose Leonardo Fernandez	jftriana@uoguelph.ca					<VERBATIM LOCALITY>|Twin Lakes, 22 km SE			A.Renaud	11-Aug-2007		Canada	Manitoba	Churchill	26 km SE Churchill, Tw		58.632	-93.788002		
ASWAV902-08	07PROBE-22361	07PROBE-22361	07PROBE-22361	620	GU141321	536	GU141530	07PROBE-22361	Arthropoda	Insecta	Hymenoptera	Braconidae	Microgastrinae	Microplitis	Microplitis varicolor	Jose Leonardo Fernandez	jftriana@uoguelph.ca					<VERBATIM LOCALITY>|Twin Lakes, 22 km SE			A.Renaud	11-Aug-2007		Canada	Manitoba	Churchill	26 km SE Churchill, Tw		58.632	-93.788002		
ASWAV903-08	07PROBE-22362	07PROBE-22362	07PROBE-22362	631	GU141370	401	GU141560	07PROBE-22362	Arthropoda	Insecta	Hymenoptera	Braconidae	Microgastrinae	Pholetesor	Pholetesor salicifoliellae	Jose Leonardo Fernandez	jftriana@uoguelph.ca					<VERBATIM LOCALITY>|Twin Lakes, 22 km SE				11-Aug-2007		Canada	Manitoba	Churchill	26 km SE Churchill, Tw		58.632	-93.788002		
ASWAV904-08	07PROBE-22363	07PROBE-22363	07PROBE-22363	625	GU141126	536	GU141440	07PROBE-22363	Arthropoda	Insecta	Hymenoptera	Braconidae	Microgastrinae	Cotesia	Cotesia jft06	Jose Leonardo Fernandez	jftriana@uoguelph.ca					<VERBATIM LOCALITY>|Twin Lakes, 22 km SE	NULL		A.Renaud	11-Aug-2007		Canada	Manitoba	Churchill	26 km SE Churchill, Tw		58.632	-93.788002		
ASWAV905-08	07PROBE-22364	07PROBE-22364	07PROBE-22364	606	GU141157	536	GU141461	07PROBE-22364	Arthropoda	Insecta	Hymenoptera	Braconidae	Microgastrinae	Dolichogenidea	Dolichogenidea jft03	Jose Leonardo Fernandez	jftriana@uoguelph.ca					<VERBATIM LOCALITY>|Twin Lakes, 22 km SE			A.Renaud	11-Aug-2007		Canada	Manitoba	Churchill	26 km SE Churchill, Tw		58.632	-93.788002		
ASWAV906-08	07PROBE-22365	07PROBE-22365	07PROBE-22365	648	GU141156	536	GU141460	07PROBE-22365	Arthropoda	Insecta	Hymenoptera	Braconidae	Microgastrinae	Dolichogenidea	Dolichogenidea jft03	Jose Leonardo Fernandez	jftriana@uoguelph.ca					<VERBATIM LOCALITY>|Twin Lakes, 22 km SE			A.Renaud	11-Aug-2007		Canada	Manitoba	Churchill	26 km SE Churchill, Tw		58.632	-93.788002		
ASWAV907-08	07PROBE-22366	07PROBE-22366	07PROBE-22366	616	GU141053	303	GU141390	07PROBE-22366	Arthropoda	Insecta	Hymenoptera	Braconidae	Microgastrinae	Apanteles	Apanteles jft01	Jose Leonardo Fernandez	jftriana@uoguelph.ca					<VERBATIM LOCALITY>|Twin Lakes, 22 km SE			A.Renaud	11-Aug-2007		Canada	Manitoba	Churchill	26 km SE Churchill, Tw		58.632	-93.788002		
ASWAV908-08	07PROBE-22367	07PROBE-22367	07PROBE-22367	620	GU141296	536	GU141516	07PROBE-22367	Arthropoda	Insecta	Hymenoptera	Braconidae	Microgastrinae	Microplitis	Microplitis varicolor	Jose Leonardo Fernandez	jftriana@uoguelph.ca					<VERBATIM LOCALITY>|Twin Lakes, 22 km SE			A.Renaud	11-Aug-2007		Canada	Manitoba	Churchill	26 km SE Churchill, Tw		58.632	-93.788002		
ASWAV909-08	07PROBE-22368	07PROBE-22368	07PROBE-22368	648	GU141257	469	GU141499	07PROBE-22368	Arthropoda	Insecta	Hymenoptera	Braconidae	Microgastrinae	Microplitis	Microplitis jft04	Jose Leonardo Fernandez	jftriana@uoguelph.ca					<VERBATIM LOCALITY>|Twin Lakes, 22 km SE			A.Renaud	11-Aug-2007		Canada	Manitoba	Churchill	26 km SE Churchill, Tw		58.632	-93.788002		
ASWAV910-08	07PROBE-22369	07PROBE-22369	07PROBE-22369	619	GU141218	536	GU141478	07PROBE-22369	Arthropoda	Insecta	Hymenoptera	Braconidae	Microgastrinae	Glyptapanteles	Glyptapanteles jft06	Jose Leonardo Fernandez	jftriana@uoguelph.ca					<VERBATIM LOCALITY>|Twin Lakes, 22 km SE	NULL		A.Renaud	11-Aug-2007		Canada	Manitoba	Churchill	26 km SE Churchill, Tw		58.632	-93.788002		
ASWAV911-08	07PROBE-22370	07PROBE-22370	07PROBE-22370	648	GU141256	469	GU141498	07PROBE-22370	Arthropoda	Insecta	Hymenoptera	Braconidae	Microgastrinae	Microplitis	Microplitis jft04	Jose Leonardo Fernandez	jftriana@uoguelph.ca					<VERBATIM LOCALITY>|Twin Lakes, 22 km SE			A.Renaud	11-Aug-2007		Canada	Manitoba	Churchill	26 km SE Churchill, Tw		58.632	-93.788002		
ASWAV912-08	07PROBE-22371	07PROBE-22371	07PROBE-22371	656	GU141275	536	GU141505	07PROBE-22371	Arthropoda	Insecta	Hymenoptera	Braconidae	Microgastrinae	Microplitis	Microplitis jft06	Jose Leonardo Fernandez	jftriana@uoguelph.ca					<VERBATIM LOCALITY>|Twin Lakes, 22 km SE			A.Renaud	11-Aug-2007		Canada	Manitoba	Churchill	26 km SE Churchill, Tw		58.632	-93.788002		
ASWAV913-08	07PROBE-22372	07PROBE-22372	07PROBE-22372	657	GU141295	536	GU141515	07PROBE-22372	Arthropoda	Insecta	Hymenoptera	Braconidae	Microgastrinae	Microplitis	Microplitis varicolor	Jose Leonardo Fernandez	jftriana@uoguelph.ca					<VERBATIM LOCALITY>|Twin Lakes, 22 km SE			A.Renaud	11-Aug-2007		Canada	Manitoba	Churchill	26 km SE Churchill, Tw		58.632	-93.788002		
ASWAV914-08	07PROBE-22373	07PROBE-22373	07PROBE-22373	614	GU141052	303	GU141389	07PROBE-22373	Arthropoda	Insecta	Hymenoptera	Braconidae	Microgastrinae	Apanteles	Apanteles jft01	Jose Leonardo Fernandez	jftriana@uoguelph.ca					<VERBATIM LOCALITY>|A-Frame, 12 Km E of			A.Renaud	08-Aug-2007		Canada	Manitoba	Churchill	10 km ESE Churchill, L		58.7550011	-93.998001		
ASWAV915-08	07PROBE-22374	07PROBE-22374	07PROBE-22374	623	GU141086	531	GU141411	07PROBE-22374	Arthropoda	Insecta	Hymenoptera	Braconidae	Microgastrinae	Cotesia	Cotesia jft05	Jose Leonardo Fernandez	jftriana@uoguelph.ca					<VERBATIM LOCALITY>|A-Frame, 12 Km E of			A.Renaud	08-Aug-2007		Canada	Manitoba	Churchill	10 km ESE Churchill, L		58.7550011	-93.998001		
ASWAV916-08	07PROBE-22375	07PROBE-22375	07PROBE-22375	657	GU141171	315	GU141469	07PROBE-22375	Arthropoda	Insecta	Hymenoptera	Braconidae	Microgastrinae	Dolichogenidea	Dolichogenidea jft06	Jose Leonardo Fernandez	jftriana@uoguelph.ca					<VERBATIM LOCALITY>|A-Frame, 12 Km E of			A.Renaud	08-Aug-2007		Canada	Manitoba	Churchill	10 km ESE Churchill, L		58.7550011	-93.998001		
ASWAV917-08	07PROBE-22376	07PROBE-22376	07PROBE-22376	631	GU141153	358	GU141457	07PROBE-22376	Arthropoda	Insecta	Hymenoptera	Braconidae	Microgastrinae	Dolichogenidea	Dolichogenidea jft02	Jose Leonardo Fernandez	jftriana@uoguelph.ca					<VERBATIM LOCALITY>|A-Frame, 12 Km E of			A.Renaud	08-Aug-2007		Canada	Manitoba	Churchill	10 km ESE Churchill, L		58.7550011	-93.998001		
ASWAV918-08	07PROBE-22377	07PROBE-22377	07PROBE-22377	620	GU141077	304	GU141406	07PROBE-22377	Arthropoda	Insecta	Hymenoptera	Braconidae	Microgastrinae	Apanteles	Apanteles polychrosidis	Jose Leonardo Fernandez	jftriana@uoguelph.ca					<VERBATIM LOCALITY>|A-Frame, 12 Km E of			A.Renaud	08-Aug-2007		Canada	Manitoba	Churchill	10 km ESE Churchill, L		58.7550011	-93.998001		
ASWAV919-08	07PROBE-22378	07PROBE-22378	07PROBE-22378	645	GU141063	536	GU141398	07PROBE-22378	Arthropoda	Insecta	Hymenoptera	Braconidae	Microgastrinae	Apanteles	Apanteles jft02	Jose Leonardo Fernandez	jftriana@uoguelph.ca					<VERBATIM LOCALITY>|A-Frame, 12 Km E of				08-Aug-2007		Canada	Manitoba	Churchill	10 km ESE Churchill, L		58.7550011	-93.998001		
ASWAV920-08	07PROBE-22379	07PROBE-22379	07PROBE-22379	627	GU141125	536	GU141439	07PROBE-22379	Arthropoda	Insecta	Hymenoptera	Braconidae	Microgastrinae	Cotesia	Cotesia jft06	Jose Leonardo Fernandez	jftriana@uoguelph.ca					<VERBATIM LOCALITY>|A-Frame, 12 Km E of	NULL			08-Aug-2007		Canada	Manitoba	Churchill	10 km ESE Churchill, L		58.7550011	-93.998001		
ASWAV921-08	07PROBE-22380	07PROBE-22380	07PROBE-22380	618	GU141129	531	GU141443	07PROBE-22380	Arthropoda	Insecta	Hymenoptera	Braconidae	Microgastrinae	Cotesia	Cotesia jft06	Jose Leonardo Fernandez	jftriana@uoguelph.ca					<VERBATIM LOCALITY>|A-Frame, 12 Km E of	NULL		A.Renaud	08-Aug-2007		Canada	Manitoba	Churchill	10 km ESE Churchill, L		58.7550011	-93.998001		
ASWAV922-08	07PROBE-22381	07PROBE-22381	07PROBE-22381	624	GU141143	536	GU141449	07PROBE-22381	Arthropoda	Insecta	Hymenoptera	Braconidae	Microgastrinae	Dolichogenidea	Dolichogenidea jft01	Jose Leonardo Fernandez	jftriana@uoguelph.ca					<VERBATIM LOCALITY>|A-Frame, 12 Km E of			A.Renaud	08-Aug-2007		Canada	Manitoba	Churchill	10 km ESE Churchill, L		58.7550011	-93.998001		
ASWAV923-08	07PROBE-22382	07PROBE-22382	07PROBE-22382	629	GU141062	536	GU141397	07PROBE-22382	Arthropoda	Insecta	Hymenoptera	Braconidae	Microgastrinae	Apanteles	Apanteles jft02	Jose Leonardo Fernandez	jftriana@uoguelph.ca					<VERBATIM LOCALITY>|A-Frame, 12 Km E of				08-Aug-2007		Canada	Manitoba	Churchill	10 km ESE Churchill, L		58.7550011	-93.998001		
ASWAV924-08	07PROBE-22383	07PROBE-22383	07PROBE-22383	289	GU141117	536	GU141431	07PROBE-22383	Arthropoda	Insecta	Hymenoptera	Braconidae	Microgastrinae	Cotesia	Cotesia jft06	Jose Leonardo Fernandez	jftriana@uoguelph.ca					<VERBATIM LOCALITY>|A-Frame, 12 Km E of				08-Aug-2007		Canada	Manitoba	Churchill	10 km ESE Churchill, L		58.7550011	-93.998001		
ASWAV925-08	07PROBE-22384	07PROBE-22384	07PROBE-22384	627	GU141120	533	GU141434	07PROBE-22384	Arthropoda	Insecta	Hymenoptera	Braconidae	Microgastrinae	Cotesia	Cotesia jft06	Jose Leonardo Fernandez	jftriana@uoguelph.ca					<VERBATIM LOCALITY>|A-Frame, 12 Km E of	NULL			08-Aug-2007		Canada	Manitoba	Churchill	10 km ESE Churchill, L		58.7550011	-93.998001		
ASWAV926-08	07PROBE-22385	07PROBE-22385	07PROBE-22385	613	GU141123	536	GU141437	07PROBE-22385	Arthropoda	Insecta	Hymenoptera	Braconidae	Microgastrinae	Cotesia	Cotesia jft06	Jose Leonardo Fernandez	jftriana@uoguelph.ca					<VERBATIM LOCALITY>|A-Frame, 12 Km E of	NULL		A.Renaud	08-Aug-2007		Canada	Manitoba	Churchill	10 km ESE Churchill, L		58.7550011	-93.998001		
ASWAV927-08	07PROBE-22386	07PROBE-22386	07PROBE-22386	657	GU141152	358	GU141456	07PROBE-22386	Arthropoda	Insecta	Hymenoptera	Braconidae	Microgastrinae	Dolichogenidea	Dolichogenidea jft02	Jose Leonardo Fernandez	jftriana@uoguelph.ca					<VERBATIM LOCALITY>|A-Frame, 12 Km E of			A.Renaud	08-Aug-2007		Canada	Manitoba	Churchill	10 km ESE Churchill, L		58.7550011	-93.998001		
ASWAV928-08	07PROBE-22387	07PROBE-22387	07PROBE-22387	617	GU141076	526	GU141405	07PROBE-22387	Arthropoda	Insecta	Hymenoptera	Braconidae	Microgastrinae	Apanteles	Apanteles petrovae	Jose Leonardo Fernandez	jftriana@uoguelph.ca					<VERBATIM LOCALITY>|A-Frame, 12 Km E of	NULL		A.Renaud	08-Aug-2007		Canada	Manitoba	Churchill	10 km ESE Churchill, L		58.7550011	-93.998001		
ASWAV929-08	07PROBE-22388	07PROBE-22388	07PROBE-22388	657	GU141226	536	GU141481	07PROBE-22388	Arthropoda	Insecta	Hymenoptera	Braconidae	Microgastrinae	Microgaster	Microgaster canadensis	Jose Leonardo Fernandez	jftriana@uoguelph.ca					<VERBATIM LOCALITY>|A-Frame, 12 Km E of			A.Renaud	08-Aug-2007		Canada	Manitoba	Churchill	10 km ESE Churchill, L		58.7550011	-93.998001		
ASWAV930-08	07PROBE-22389	07PROBE-22389	07PROBE-22389	620	GU141167	304	GU141467	07PROBE-22389	Arthropoda	Insecta	Hymenoptera	Braconidae	Microgastrinae	Dolichogenidea	Dolichogenidea jft05	Jose Leonardo Fernandez	jftriana@uoguelph.ca					<VERBATIM LOCALITY>|A-Frame, 12 Km E of			A.Renaud	08-Aug-2007		Canada	Manitoba	Churchill	10 km ESE Churchill, L		58.7550011	-93.998001		
ASWAV931-08	07PROBE-22390	07PROBE-22390	07PROBE-22390	657	GU141174	303	GU141471	07PROBE-22390	Arthropoda	Insecta	Hymenoptera	Braconidae	Microgastrinae	Glyptapanteles	Glyptapanteles jft01	Jose Leonardo Fernandez	jftriana@uoguelph.ca					<VERBATIM LOCALITY>|A-Frame, 12 Km E of			A.Renaud	08-Aug-2007		Canada	Manitoba	Churchill	10 km ESE Churchill, L		58.7550011	-93.998001		
ASWAV932-08	07PROBE-22391	07PROBE-22391	07PROBE-22391	657	GU141239	536	GU141488	07PROBE-22391	Arthropoda	Insecta	Hymenoptera	Braconidae	Microgastrinae	Microgaster	Microgaster jft06	Jose Leonardo Fernandez	jftriana@uoguelph.ca					<VERBATIM LOCALITY>|A-Frame, 12 Km E of				08-Aug-2007		Canada	Manitoba	Churchill	10 km ESE Churchill, L		58.7550011	-93.998001		
ASWAV933-08	07PROBE-22392	07PROBE-22392	07PROBE-22392	585	GU141119	536	GU141433	07PROBE-22392	Arthropoda	Insecta	Hymenoptera	Braconidae	Microgastrinae	Cotesia	Cotesia jft06	Jose Leonardo Fernandez	jftriana@uoguelph.ca					<VERBATIM LOCALITY>|A-Frame, 12 Km E of	NULL			08-Aug-2007		Canada	Manitoba	Churchill	10 km ESE Churchill, L		58.7550011	-93.998001		
ASWAV934-08	07PROBE-22393	07PROBE-22393	07PROBE-22393	623	GU141122	536	GU141436	07PROBE-22393	Arthropoda	Insecta	Hymenoptera	Braconidae	Microgastrinae	Cotesia	Cotesia jft06	Jose Leonardo Fernandez	jftriana@uoguelph.ca					<VERBATIM LOCALITY>|A-Frame, 12 Km E of	NULL		A.Renaud	08-Aug-2007		Canada	Manitoba	Churchill	10 km ESE Churchill, L		58.7550011	-93.998001		
ASWAV935-08	07PROBE-22394	07PROBE-22394	07PROBE-22394	620	GU141085	536	GU141410	07PROBE-22394	Arthropoda	Insecta	Hymenoptera	Braconidae	Microgastrinae	Cotesia	Cotesia jft05	Jose Leonardo Fernandez	jftriana@uoguelph.ca					<VERBATIM LOCALITY>|A-Frame, 12 Km E of				08-Aug-2007		Canada	Manitoba	Churchill	10 km ESE Churchill, L		58.7550011	-93.998001		
ASWAV936-08	07PROBE-22395	07PROBE-22395	07PROBE-22395	617	GU141124	536	GU141438	07PROBE-22395	Arthropoda	Insecta	Hymenoptera	Braconidae	Microgastrinae	Cotesia	Cotesia jft06	Jose Leonardo Fernandez	jftriana@uoguelph.ca					<VERBATIM LOCALITY>|A-Frame, 12 Km E of	NULL		A.Renaud	08-Aug-2007		Canada	Manitoba	Churchill	10 km ESE Churchill, L		58.7550011	-93.998001		
ASWAV937-08	07PROBE-22396	07PROBE-22396	07PROBE-22396	649	GU141255	469	GU141497	07PROBE-22396	Arthropoda	Insecta	Hymenoptera	Braconidae	Microgastrinae	Microplitis	Microplitis jft04	Jose Leonardo Fernandez	jftriana@uoguelph.ca					<VERBATIM LOCALITY>|A-Frame, 12 Km E of				08-Aug-2007		Canada	Manitoba	Churchill	10 km ESE Churchill, L		58.7550011	-93.998001		
ASWAV938-08	07PROBE-22397	07PROBE-22397	07PROBE-22397	622	GU141115	533	GU141429	07PROBE-22397	Arthropoda	Insecta	Hymenoptera	Braconidae	Microgastrinae	Cotesia	Cotesia jft06	Jose Leonardo Fernandez	jftriana@uoguelph.ca					<VERBATIM LOCALITY>|A-Frame, 12 Km E of	NULL			08-Aug-2007		Canada	Manitoba	Churchill	10 km ESE Churchill, L		58.7550011	-93.998001		
ASWAV939-08	07PROBE-22398	07PROBE-22398	07PROBE-22398	627	GU141116	536	GU141430	07PROBE-22398	Arthropoda	Insecta	Hymenoptera	Braconidae	Microgastrinae	Cotesia	Cotesia jft06	Jose Leonardo Fernandez	jftriana@uoguelph.ca					<VERBATIM LOCALITY>|A-Frame, 12 Km E of	NULL		A.Renaud	08-Aug-2007		Canada	Manitoba	Churchill	10 km ESE Churchill, L		58.7550011	-93.998001		
ASWAV940-08	07PROBE-22399	07PROBE-22399	07PROBE-22399	630	GU141247			07PROBE-22399	Arthropoda	Insecta	Hymenoptera	Braconidae	Microgastrinae	Microplitis	Microplitis jft02	Jose Leonardo Fernandez	jftriana@uoguelph.ca					<VERBATIM LOCALITY>|A-Frame, 12 Km E of				08-Aug-2007		Canada	Manitoba	Churchill	10 km ESE Churchill, L		58.7550011	-93.998001		
ASWAV941-08	07PROBE-22400	07PROBE-22400	07PROBE-22400	621	GU141118	533	GU141432	07PROBE-22400	Arthropoda	Insecta	Hymenoptera	Braconidae	Microgastrinae	Cotesia	Cotesia jft06	Jose Leonardo Fernandez	jftriana@uoguelph.ca					<VERBATIM LOCALITY>|A-Frame, 12 Km E of	NULL		A.Renaud	08-Aug-2007		Canada	Manitoba	Churchill	10 km ESE Churchill, L		58.7550011	-93.998001		
ASWAV942-08	07PROBE-22401	07PROBE-22401	07PROBE-22401	645	GU141142	536	GU141448	07PROBE-22401	Arthropoda	Insecta	Hymenoptera	Braconidae	Microgastrinae	Dolichogenidea	Dolichogenidea jft01	Jose Leonardo Fernandez	jftriana@uoguelph.ca					<VERBATIM LOCALITY>|A-Frame, 12 Km E of			A.Renaud	08-Aug-2007		Canada	Manitoba	Churchill	10 km ESE Churchill, L		58.7550011	-93.998001		
ASWAV943-08	07PROBE-22402	07PROBE-22402	07PROBE-22402	655	GU141151	358	GU141455	07PROBE-22402	Arthropoda	Insecta	Hymenoptera	Braconidae	Microgastrinae	Dolichogenidea	Dolichogenidea jft02	Jose Leonardo Fernandez	jftriana@uoguelph.ca					<VERBATIM LOCALITY>|A-Frame, 12 Km E of			A.Renaud	08-Aug-2007		Canada	Manitoba	Churchill	10 km ESE Churchill, L		58.7550011	-93.998001		
ASWAV944-08	07PROBE-22403	07PROBE-22403	07PROBE-22403	623	GU141094	535	GU141417	07PROBE-22403	Arthropoda	Insecta	Hymenoptera	Braconidae	Microgastrinae	Cotesia	Cotesia jft06	Jose Leonardo Fernandez	jftriana@uoguelph.ca					<VERBATIM LOCALITY>|A-Frame, 12 Km E of				08-Aug-2007		Canada	Manitoba	Churchill	10 km ESE Churchill, L		58.7550011	-93.998001		
ASWAV945-08	07PROBE-22404	07PROBE-22404	07PROBE-22404	626	GU141128	532	GU141442	07PROBE-22404	Arthropoda	Insecta	Hymenoptera	Braconidae	Microgastrinae	Cotesia	Cotesia jft06	Jose Leonardo Fernandez	jftriana@uoguelph.ca					<VERBATIM LOCALITY>|A-Frame, 12 Km E of	NULL		A.Renaud	08-Aug-2007		Canada	Manitoba	Churchill	10 km ESE Churchill, L		58.7550011	-93.998001		
ASWAV946-08	07PROBE-22405	07PROBE-22405	07PROBE-22405	631	GU141141	536	GU141447	07PROBE-22405	Arthropoda	Insecta	Hymenoptera	Braconidae	Microgastrinae	Dolichogenidea	Dolichogenidea jft01	Jose Leonardo Fernandez	jftriana@uoguelph.ca					<VERBATIM LOCALITY>|A-Frame, 12 Km E of			A.Renaud	08-Aug-2007		Canada	Manitoba	Churchill	10 km ESE Churchill, L		58.7550011	-93.998001		
ASWAV947-08	07PROBE-22406	07PROBE-22406	07PROBE-22406	657	GU141170	315	GU141468	07PROBE-22406	Arthropoda	Insecta	Hymenoptera	Braconidae	Microgastrinae	Dolichogenidea	Dolichogenidea jft06	Jose Leonardo Fernandez	jftriana@uoguelph.ca					<VERBATIM LOCALITY>|A-Frame, 12 Km E of			A.Renaud	08-Aug-2007		Canada	Manitoba	Churchill	10 km ESE Churchill, L		58.7550011	-93.998001		
ASWAV948-08	07PROBE-22407	07PROBE-22407	07PROBE-22407	657	GU141150	358	GU141454	07PROBE-22407	Arthropoda	Insecta	Hymenoptera	Braconidae	Microgastrinae	Dolichogenidea	Dolichogenidea jft02	Jose Leonardo Fernandez	jftriana@uoguelph.ca					<VERBATIM LOCALITY>|A-Frame, 12 Km E of			A.Renaud	08-Aug-2007		Canada	Manitoba	Churchill	10 km ESE Churchill, L		58.7550011	-93.998001		
ASWAV949-08	07PROBE-22408	07PROBE-22408	07PROBE-22408	644	GU141149	358	GU141453	07PROBE-22408	Arthropoda	Insecta	Hymenoptera	Braconidae	Microgastrinae	Dolichogenidea	Dolichogenidea jft02	Jose Leonardo Fernandez	jftriana@uoguelph.ca					<VERBATIM LOCALITY>|A-Frame, 12 Km E of				08-Aug-2007		Canada	Manitoba	Churchill	10 km ESE Churchill, L		58.7550011	-93.998001		
ASWAV950-08	07PROBE-22409	07PROBE-22409	07PROBE-22409	622	GU141121	536	GU141435	07PROBE-22409	Arthropoda	Insecta	Hymenoptera	Braconidae	Microgastrinae	Cotesia	Cotesia jft06	Jose Leonardo Fernandez	jftriana@uoguelph.ca					<VERBATIM LOCALITY>|A-Frame, 12 Km E of	NULL		A.Renaud	08-Aug-2007		Canada	Manitoba	Churchill	10 km ESE Churchill, L		58.7550011	-93.998001		
ASWAV951-08	07PROBE-22410	07PROBE-22410	07PROBE-22410	657	GU141148	358	GU141452	07PROBE-22410	Arthropoda	Insecta	Hymenoptera	Braconidae	Microgastrinae	Dolichogenidea	Dolichogenidea jft02	Jose Leonardo Fernandez	jftriana@uoguelph.ca					<VERBATIM LOCALITY>|A-Frame, 12 Km E of			A.Renaud	08-Aug-2007		Canada	Manitoba	Churchill	10 km ESE Churchill, L		58.7550011	-93.998001		
ASWAV952-08	07PROBE-22411	07PROBE-22411	07PROBE-22411	657	GU141147	358	GU141451	07PROBE-22411	Arthropoda	Insecta	Hymenoptera	Braconidae	Microgastrinae	Dolichogenidea	Dolichogenidea jft02	Jose Leonardo Fernandez	jftriana@uoguelph.ca					<VERBATIM LOCALITY>|Ramsay Creek|58.75			A.Renaud	16-Jul-2007		Canada	Manitoba	Churchill	23 km E Churchill, Ram		58.730999	-93.779999	10	
ASWAV953-08	07PROBE-22412	07PROBE-22412	07PROBE-22412	657	GU141061	536	GU141396	07PROBE-22412	Arthropoda	Insecta	Hymenoptera	Braconidae	Microgastrinae	Apanteles	Apanteles jft02	Jose Leonardo Fernandez	jftriana@uoguelph.ca					<VERBATIM LOCALITY>|Ramsay Creek|58.75			A.Renaud	16-Jul-2007		Canada	Manitoba	Churchill	23 km E Churchill, Ram		58.730999	-93.779999	10	
ASWAV954-08	07PROBE-22413	07PROBE-22413	07PROBE-22413	623	GU141106	536	GU141425	07PROBE-22413	Arthropoda	Insecta	Hymenoptera	Braconidae	Microgastrinae	Cotesia	Cotesia jft08	Jose Leonardo Fernandez	jftriana@uoguelph.ca					<VERBATIM LOCALITY>|Ramsay Creek|58.75			A.Renaud	16-Jul-2007		Canada	Manitoba	Churchill	23 km E Churchill, Ram		58.730999	-93.779999	10	
ASWAV955-08	07PROBE-22414	07PROBE-22414	07PROBE-22414	657	GU141060	536	GU141395	07PROBE-22414	Arthropoda	Insecta	Hymenoptera	Braconidae	Microgastrinae	Apanteles	Apanteles jft02	Jose Leonardo Fernandez	jftriana@uoguelph.ca					<VERBATIM LOCALITY>|Ramsay Creek|58.75			A.Renaud	16-Jul-2007		Canada	Manitoba	Churchill	23 km E Churchill, Ram		58.730999	-93.779999	10	
ASWAV956-08	07PROBE-22415	07PROBE-22415	07PROBE-22415	599	GU141294	533	GU141514	07PROBE-22415	Arthropoda	Insecta	Hymenoptera	Braconidae	Microgastrinae	Microplitis	Microplitis varicolor	Jose Leonardo Fernandez	jftriana@uoguelph.ca					<VERBATIM LOCALITY>|Ramsay Creek|58.75			A.Renaud	16-Jul-2007		Canada	Manitoba	Churchill	23 km E Churchill, Ram		58.730999	-93.779999	10	
ASWAV958-08	07PROBE-22417	07PROBE-22417	07PROBE-22417	632	GU141339	536	GU141544	07PROBE-22417	Arthropoda	Insecta	Hymenoptera	Braconidae	Microgastrinae	Pholetesor	Pholetesor jft02	Jose Leonardo Fernandez	jftriana@uoguelph.ca					<VERBATIM LOCALITY>|Ramsay Creek|58.75			A.Renaud	16-Jul-2007		Canada	Manitoba	Churchill	23 km E Churchill, Ram		58.730999	-93.779999	10	
ASWAV959-08	07PROBE-22418	07PROBE-22418	07PROBE-22418	656	GU141293	536	GU141513	07PROBE-22418	Arthropoda	Insecta	Hymenoptera	Braconidae	Microgastrinae	Microplitis	Microplitis varicolor	Jose Leonardo Fernandez	jftriana@uoguelph.ca					<VERBATIM LOCALITY>|Ramsay Creek|58.75			A.Renaud	16-Jul-2007		Canada	Manitoba	Churchill	23 km E Churchill, Ram		58.730999	-93.779999	10	
ASWAV960-08	07PROBE-22419	07PROBE-22419	07PROBE-22419	630	GU141246	536	GU141491	07PROBE-22419	Arthropoda	Insecta	Hymenoptera	Braconidae	Microgastrinae	Microplitis	Microplitis jft02	Jose Leonardo Fernandez	jftriana@uoguelph.ca					<VERBATIM LOCALITY>|Ramsay Creek|58.75			A.Renaud	16-Jul-2007		Canada	Manitoba	Churchill	23 km E Churchill, Ram		58.730999	-93.779999	10	
ASWAV961-08	07PROBE-22420	07PROBE-22420	07PROBE-22420	657	GU141290	536	GU141511	07PROBE-22420	Arthropoda	Insecta	Hymenoptera	Braconidae	Microgastrinae	Microplitis	Microplitis jft12	Jose Leonardo Fernandez	jftriana@uoguelph.ca					<VERBATIM LOCALITY>|Ramsay Creek|58.75			A.Renaud	16-Jul-2007		Canada	Manitoba	Churchill	23 km E Churchill, Ram		58.730999	-93.779999	10	
ASWAV962-08	07PROBE-22421	07PROBE-22421	07PROBE-22421	620	GU141173	303	GU141470	07PROBE-22421	Arthropoda	Insecta	Hymenoptera	Braconidae	Microgastrinae	Glyptapanteles	Glyptapanteles jft01	Jose Leonardo Fernandez	jftriana@uoguelph.ca					<VERBATIM LOCALITY>|Ramsay Creek|58.75			A.Renaud	16-Jul-2007		Canada	Manitoba	Churchill	23 km E Churchill, Ram		58.730999	-93.779999	10	
ASWAV963-08	07PROBE-22422	07PROBE-22422	07PROBE-22422	631	GU141059	536	GU141394	07PROBE-22422	Arthropoda	Insecta	Hymenoptera	Braconidae	Microgastrinae	Apanteles	Apanteles jft02	Jose Leonardo Fernandez	jftriana@uoguelph.ca					<VERBATIM LOCALITY>|Ramsay Creek|58.75			A.Renaud	16-Jul-2007		Canada	Manitoba	Churchill	23 km E Churchill, Ram		58.730999	-93.779999	10	
ASWAV964-08	07PROBE-22423	07PROBE-22423	07PROBE-22423	613	GU141374	536	GU141562	07PROBE-22423	Arthropoda	Insecta	Hymenoptera	Braconidae	Microgastrinae	Pholetesor	Pholetesor viminetorum	Jose Leonardo Fernandez	jftriana@uoguelph.ca					<VERBATIM LOCALITY>|Ramsay Creek|58.75			A.Renaud	16-Jul-2007		Canada	Manitoba	Churchill	23 km E Churchill, Ram		58.730999	-93.779999	10	
ASWAV965-08	07PROBE-22424	07PROBE-22424	07PROBE-22424	619	GU141289	536	GU141510	07PROBE-22424	Arthropoda	Insecta	Hymenoptera	Braconidae	Microgastrinae	Microplitis	Microplitis jft12	Jose Leonardo Fernandez	jftriana@uoguelph.ca					<VERBATIM LOCALITY>|Ramsay Creek|58.75			A.Renaud	16-Jul-2007		Canada	Manitoba	Churchill	23 km E Churchill, Ram		58.730999	-93.779999	10	
ASWAV966-08	07PROBE-22425	07PROBE-22425	07PROBE-22425	657	GU141084	536	GU141409	07PROBE-22425	Arthropoda	Insecta	Hymenoptera	Braconidae	Microgastrinae	Cotesia	Cotesia jft05	Jose Leonardo Fernandez	jftriana@uoguelph.ca					<VERBATIM LOCALITY>|Ramsay Creek|58.75			A.Renaud	16-Jul-2007		Canada	Manitoba	Churchill	23 km E Churchill, Ram		58.730999	-93.779999	10	
ASWAV967-08	07PROBE-22426	07PROBE-22426	07PROBE-22426	631	GU141343	381	GU141546	07PROBE-22426	Arthropoda	Insecta	Hymenoptera	Braconidae	Microgastrinae	Pholetesor	Pholetesor ornigis	Jose Leonardo Fernandez	jftriana@uoguelph.ca					<VERBATIM LOCALITY>|Ramsay Creek|58.75			A.Renaud	16-Jul-2007		Canada	Manitoba	Churchill	23 km E Churchill, Ram		58.730999	-93.779999	10	
ASWAV968-08	07PROBE-22427	07PROBE-22427	07PROBE-22427	626	GU141105	536	GU141424	07PROBE-22427	Arthropoda	Insecta	Hymenoptera	Braconidae	Microgastrinae	Cotesia	Cotesia jft08	Jose Leonardo Fernandez	jftriana@uoguelph.ca					<VERBATIM LOCALITY>|Ramsay Creek|58.75			A.Renaud	16-Jul-2007		Canada	Manitoba	Churchill	23 km E Churchill, Ram		58.730999	-93.779999	10	
ASWAV969-08	07PROBE-22428	07PROBE-22428	07PROBE-22428	645	GU141292	534	GU141512	07PROBE-22428	Arthropoda	Insecta	Hymenoptera	Braconidae	Microgastrinae	Microplitis	Microplitis varicolor	Jose Leonardo Fernandez	jftriana@uoguelph.ca					<VERBATIM LOCALITY>|Ramsay Creek|58.75			A.Renaud	16-Jul-2007		Canada	Manitoba	Churchill	23 km E Churchill, Ram		58.730999	-93.779999	10	
ASWAV970-08	07PROBE-22429	07PROBE-22429	07PROBE-22429	613	GU141291			07PROBE-22429	Arthropoda	Insecta	Hymenoptera	Braconidae	Microgastrinae	Microplitis	Microplitis varicolor	Jose Leonardo Fernandez	jftriana@uoguelph.ca					<VERBATIM LOCALITY>|Ramsay Creek|58.75			A.Renaud	16-Jul-2007		Canada	Manitoba	Churchill	23 km E Churchill, Ram		58.730999	-93.779999	10	
ASWAV971-08	07PROBE-22430	07PROBE-22430	07PROBE-22430	645	GU141058	536	GU141393	07PROBE-22430	Arthropoda	Insecta	Hymenoptera	Braconidae	Microgastrinae	Apanteles	Apanteles jft02	Jose Leonardo Fernandez	jftriana@uoguelph.ca					<VERBATIM LOCALITY>|Ramsay Creek|58.75			A.Renaud	16-Jul-2007		Canada	Manitoba	Churchill	23 km E Churchill, Ram		58.730999	-93.779999	10	
ASWAV972-08	07PROBE-22431	07PROBE-22431	07PROBE-22431	631	GU141104	536	GU141423	07PROBE-22431	Arthropoda	Insecta	Hymenoptera	Braconidae	Microgastrinae	Cotesia	Cotesia jft08	Jose Leonardo Fernandez	jftriana@uoguelph.ca					<VERBATIM LOCALITY>|Ramsay Creek|58.75			A.Renaud	16-Jul-2007		Canada	Manitoba	Churchill	23 km E Churchill, Ram		58.730999	-93.779999	10	
ASWAV973-08	07PROBE-22432	07PROBE-22432	07PROBE-22432	635	GU141280	534	GU141509	07PROBE-22432	Arthropoda	Insecta	Hymenoptera	Braconidae	Microgastrinae	Microplitis	Microplitis jft08	Jose Leonardo Fernandez	jftriana@uoguelph.ca					<VERBATIM LOCALITY>|Ramsay Creek|58.75			A.Renaud	16-Jul-2007		Canada	Manitoba	Churchill	23 km E Churchill, Ram		58.730999	-93.779999	10	
ASWAV974-08	07PROBE-22433	07PROBE-22433	07PROBE-22433	623	GU141103	536	GU141422	07PROBE-22433	Arthropoda	Insecta	Hymenoptera	Braconidae	Microgastrinae	Cotesia	Cotesia jft08	Jose Leonardo Fernandez	jftriana@uoguelph.ca					<VERBATIM LOCALITY>|Ramsay Creek|58.75			A.Renaud	02-Aug-2007		Canada	Manitoba	Churchill	23 km E Churchill, Ram		58.730999	-93.779999	10	
ASWAV975-08	07PROBE-22434	07PROBE-22434	07PROBE-22434	624	GU141051	303	GU141388	07PROBE-22434	Arthropoda	Insecta	Hymenoptera	Braconidae	Microgastrinae	Apanteles	Apanteles jft01	Jose Leonardo Fernandez	jftriana@uoguelph.ca					<VERBATIM LOCALITY>|Ramsay Creek|58.75				21-Jul-2007		Canada	Manitoba	Churchill	23 km E Churchill, Ram		58.730999	-93.779999	10	
ASWAX941-08	07PROBE-23375	07PROBE-23375	07PROBE-23375	577	GU141363	381	GU141555	07PROBE-23375	Arthropoda	Insecta	Hymenoptera	Braconidae	Microgastrinae	Pholetesor	Pholetesor salicifoliellae	Jose Leonardo Fernandez	jftriana@uoguelph.ca					<VERBATIM LOCALITY>|Pumphouse|58.373|-9			A.Renaud	10-Aug-2007		Canada	Manitoba	Churchill	16 km SW Churchill, Ch		58.6259995	-94.228996		
ASWAX942-08	07PROBE-23376	07PROBE-23376	07PROBE-23376	657	GU141362	381	GU141554	07PROBE-23376	Arthropoda	Insecta	Hymenoptera	Braconidae	Microgastrinae	Pholetesor	Pholetesor salicifoliellae	Jose Leonardo Fernandez	jftriana@uoguelph.ca					<VERBATIM LOCALITY>|Pumphouse|58.373|-9			A.Renaud	10-Aug-2007		Canada	Manitoba	Churchill	16 km SW Churchill, Ch		58.6259995	-94.228996		
ASWAX943-08	07PROBE-23377	07PROBE-23377	07PROBE-23377	657	GU141050	531	GU141387	07PROBE-23377	Arthropoda	Insecta	Hymenoptera	Braconidae	Microgastrinae	Apanteles	Apanteles jft01	Jose Leonardo Fernandez	jftriana@uoguelph.ca					<VERBATIM LOCALITY>|Pumphouse|58.373|-9			A.Renaud	10-Aug-2007		Canada	Manitoba	Churchill	16 km SW Churchill, Ch		58.6259995	-94.228996		
ASWAX944-08	07PROBE-23378	07PROBE-23378	07PROBE-23378	632	GU141361	403	GU141553	07PROBE-23378	Arthropoda	Insecta	Hymenoptera	Braconidae	Microgastrinae	Pholetesor	Pholetesor salicifoliellae	Jose Leonardo Fernandez	jftriana@uoguelph.ca					<VERBATIM LOCALITY>|Pumphouse|58.373|-9			A.Renaud	10-Aug-2007		Canada	Manitoba	Churchill	16 km SW Churchill, Ch		58.6259995	-94.228996		
ASWAX945-08	07PROBE-23379	07PROBE-23379	07PROBE-23379	657	GU141132	535	GU141446	07PROBE-23379	Arthropoda	Insecta	Hymenoptera	Braconidae	Microgastrinae	Cotesia	Cotesia xylina	Jose Leonardo Fernandez	jftriana@uoguelph.ca					<VERBATIM LOCALITY>|Pumphouse|58.373|-9			A.Renaud	10-Aug-2007		Canada	Manitoba	Churchill	16 km SW Churchill, Ch		58.6259995	-94.228996		
ASWAX946-08	07PROBE-23380	07PROBE-23380	07PROBE-23380	657	GU141131	534	GU141445	07PROBE-23380	Arthropoda	Insecta	Hymenoptera	Braconidae	Microgastrinae	Cotesia	Cotesia xylina	Jose Leonardo Fernandez	jftriana@uoguelph.ca					<VERBATIM LOCALITY>|Pumphouse|58.373|-9			A.Renaud	10-Aug-2007		Canada	Manitoba	Churchill	16 km SW Churchill, Ch		58.6259995	-94.228996		
ASWAX947-08	07PROBE-23381	07PROBE-23381	07PROBE-23381	629	GU141360	518	GU141552	07PROBE-23381	Arthropoda	Insecta	Hymenoptera	Braconidae	Microgastrinae	Pholetesor	Pholetesor salicifoliellae	Jose Leonardo Fernandez	jftriana@uoguelph.ca					<VERBATIM LOCALITY>|Pumphouse|58.373|-9			A.Renaud	10-Aug-2007		Canada	Manitoba	Churchill	16 km SW Churchill, Ch		58.6259995	-94.228996		
ASWAX948-08	07PROBE-23382	07PROBE-23382	07PROBE-23382	656	GU141359	380	GU141551	07PROBE-23382	Arthropoda	Insecta	Hymenoptera	Braconidae	Microgastrinae	Pholetesor	Pholetesor salicifoliellae	Jose Leonardo Fernandez	jftriana@uoguelph.ca					<VERBATIM LOCALITY>|Pumphouse|58.373|-9			A.Renaud	10-Aug-2007		Canada	Manitoba	Churchill	16 km SW Churchill, Ch		58.6259995	-94.228996		
ASWAX949-08	07PROBE-23383	07PROBE-23383	07PROBE-23383	623	GU141358	518	GU141550	07PROBE-23383	Arthropoda	Insecta	Hymenoptera	Braconidae	Microgastrinae	Pholetesor	Pholetesor salicifoliellae	Jose Leonardo Fernandez	jftriana@uoguelph.ca					<VERBATIM LOCALITY>|Pumphouse|58.373|-9			A.Renaud	10-Aug-2007		Canada	Manitoba	Churchill	16 km SW Churchill, Ch		58.6259995	-94.228996		
ASWAX950-08	07PROBE-23384	07PROBE-23384	07PROBE-23384	657	GU141130	536	GU141444	07PROBE-23384	Arthropoda	Insecta	Hymenoptera	Braconidae	Microgastrinae	Cotesia	Cotesia xylina	Jose Leonardo Fernandez	jftriana@uoguelph.ca					<VERBATIM LOCALITY>|Pumphouse|58.373|-9			A.Renaud	10-Aug-2007		Canada	Manitoba	Churchill	16 km SW Churchill, Ch		58.6259995	-94.228996		
ASWAX952-08	07PROBE-23386	07PROBE-23386	07PROBE-23386	657	GU141378	511	GU141564	07PROBE-23386	Arthropoda	Insecta	Hymenoptera	Braconidae	Microgastrinae	Protapanteles	Protapanteles jft02	Jose Leonardo Fernandez	jftriana@uoguelph.ca					<VERBATIM LOCALITY>|Twin Lakes, 22 km SE			A.Renaud	17-Jul-2007		Canada	Manitoba	Churchill	26 km SE Churchill, Tw		58.632	-93.788002		
ASWAX953-08	07PROBE-23387	07PROBE-23387	07PROBE-23387	657	GU141238	536	GU141487	07PROBE-23387	Arthropoda	Insecta	Hymenoptera	Braconidae	Microgastrinae	Microgaster	Microgaster jft06	Jose Leonardo Fernandez	jftriana@uoguelph.ca					<VERBATIM LOCALITY>|Twin Lakes, 22 km SE			A.Renaud	17-Jul-2007		Canada	Manitoba	Churchill	26 km SE Churchill, Tw		58.632	-93.788002		
ASWAX954-08	07PROBE-23388	07PROBE-23388	07PROBE-23388	657	GU141357	381	GU141549	07PROBE-23388	Arthropoda	Insecta	Hymenoptera	Braconidae	Microgastrinae	Pholetesor	Pholetesor salicifoliellae	Jose Leonardo Fernandez	jftriana@uoguelph.ca					<VERBATIM LOCALITY>|Twin Lakes, 22 km SE			A.Renaud	17-Jul-2007		Canada	Manitoba	Churchill	26 km SE Churchill, Tw		58.632	-93.788002		
ASWAX955-08	07PROBE-23389	07PROBE-23389	07PROBE-23389	615	GU141272	469	GU141503	07PROBE-23389	Arthropoda	Insecta	Hymenoptera	Braconidae	Microgastrinae	Microplitis	Microplitis jft04	Jose Leonardo Fernandez	jftriana@uoguelph.ca					<VERBATIM LOCALITY>|Twin Lakes, 22 km SE			A.Renaud	17-Jul-2007		Canada	Manitoba	Churchill	26 km SE Churchill, Tw		58.632	-93.788002		
ASWAX956-08	07PROBE-23390	07PROBE-23390	07PROBE-23390	631	GU141369	381	GU141559	07PROBE-23390	Arthropoda	Insecta	Hymenoptera	Braconidae	Microgastrinae	Pholetesor	Pholetesor salicifoliellae	Jose Leonardo Fernandez	jftriana@uoguelph.ca					<VERBATIM LOCALITY>|Twin Lakes, 22 km SE			A.Renaud	17-Jul-2007		Canada	Manitoba	Churchill	26 km SE Churchill, Tw		58.632	-93.788002		
ASWAX957-08	07PROBE-23391	07PROBE-23391	07PROBE-23391	657	GU141234	536	GU141486	07PROBE-23391	Arthropoda	Insecta	Hymenoptera	Braconidae	Microgastrinae	Microgaster	Microgaster jft10	Jose Leonardo Fernandez	jftriana@uoguelph.ca					<VERBATIM LOCALITY>|Twin Lakes, 22 km SE			A.Renaud	17-Jul-2007		Canada	Manitoba	Churchill	26 km SE Churchill, Tw		58.632	-93.788002		
ASWAX958-08	07PROBE-23392	07PROBE-23392	07PROBE-23392	657	GU141271	469	GU141502	07PROBE-23392	Arthropoda	Insecta	Hymenoptera	Braconidae	Microgastrinae	Microplitis	Microplitis jft04	Jose Leonardo Fernandez	jftriana@uoguelph.ca					<VERBATIM LOCALITY>|Twin Lakes, 22 km SE			A.Renaud	17-Jul-2007		Canada	Manitoba	Churchill	26 km SE Churchill, Tw		58.632	-93.788002		
ASWAX959-08	07PROBE-23393	07PROBE-23393	07PROBE-23393	657	GU141046	473	GU141386	07PROBE-23393	Arthropoda	Insecta	Hymenoptera	Braconidae	Microgastrinae	Apanteles	Apanteles ensiger	Jose Leonardo Fernandez	jftriana@uoguelph.ca					<VERBATIM LOCALITY>|Ramsay Creek|58.75			A.Renaud	23-Jul-2007		Canada	Manitoba	Churchill	23 km E Churchill, Ram		58.730999	-93.779999	10	
ASWAX960-08	07PROBE-23394	07PROBE-23394	07PROBE-23394	657	GU141253	536	GU141496	07PROBE-23394	Arthropoda	Insecta	Hymenoptera	Braconidae	Microgastrinae	Microplitis	Microplitis jft02	Jose Leonardo Fernandez	jftriana@uoguelph.ca					<VERBATIM LOCALITY>|Ramsay Creek|58.75			A.Renaud	23-Jul-2007		Canada	Manitoba	Churchill	23 km E Churchill, Ram		58.730999	-93.779999	10	
ASWAX961-08	07PROBE-23395	07PROBE-23395	07PROBE-23395	657	GU141070	536	GU141404	07PROBE-23395	Arthropoda	Insecta	Hymenoptera	Braconidae	Microgastrinae	Apanteles	Apanteles jft02	Jose Leonardo Fernandez	jftriana@uoguelph.ca					<VERBATIM LOCALITY>|Ramsay Creek|58.75			A.Renaud	23-Jul-2007		Canada	Manitoba	Churchill	23 km E Churchill, Ram		58.730999	-93.779999	10	
ASWAX962-08	07PROBE-23396	07PROBE-23396	07PROBE-23396	281	GU141252	536	GU141495	07PROBE-23396	Arthropoda	Insecta	Hymenoptera	Braconidae	Microgastrinae	Microplitis	Microplitis jft02	Jose Leonardo Fernandez	jftriana@uoguelph.ca					<VERBATIM LOCALITY>|Ramsay Creek|58.75			A.Renaud	23-Jul-2007		Canada	Manitoba	Churchill	23 km E Churchill, Ram		58.730999	-93.779999	10	
ASWAX963-08	07PROBE-23397	07PROBE-23397	07PROBE-23397	657	GU141093	536	GU141416	07PROBE-23397	Arthropoda	Insecta	Hymenoptera	Braconidae	Microgastrinae	Cotesia	Cotesia jft05	Jose Leonardo Fernandez	jftriana@uoguelph.ca					<VERBATIM LOCALITY>|Ramsay Creek|58.75			A.Renaud	23-Jul-2007		Canada	Manitoba	Churchill	23 km E Churchill, Ram		58.730999	-93.779999	10	
ASWAX964-08	07PROBE-23398	07PROBE-23398	07PROBE-23398	393	GU141384	381	GU141565	07PROBE-23398	Arthropoda	Insecta	Hymenoptera	Braconidae	Microgastrinae	Protapanteles	Protapanteles jft03	Jose Leonardo Fernandez	jftriana@uoguelph.ca					<VERBATIM LOCALITY>|Ramsay Creek|58.75			A.Renaud	23-Jul-2007		Canada	Manitoba	Churchill	23 km E Churchill, Ram		58.730999	-93.779999	10	
ASWAX965-08	07PROBE-23399	07PROBE-23399	07PROBE-23399	657	GU141340	536	GU141545	07PROBE-23399	Arthropoda	Insecta	Hymenoptera	Braconidae	Microgastrinae	Pholetesor	Pholetesor jft02	Jose Leonardo Fernandez	jftriana@uoguelph.ca					<VERBATIM LOCALITY>|Ramsay Creek|58.75			A.Renaud	23-Jul-2007		Canada	Manitoba	Churchill	23 km E Churchill, Ram		58.730999	-93.779999	10	
ASWAX966-08	07PROBE-23400	07PROBE-23400	07PROBE-23400	657	GU141354	530	GU141548	07PROBE-23400	Arthropoda	Insecta	Hymenoptera	Braconidae	Microgastrinae	Pholetesor	Pholetesor ornigis	Jose Leonardo Fernandez	jftriana@uoguelph.ca					<VERBATIM LOCALITY>|Ramsay Creek|58.75			A.Renaud	23-Jul-2007		Canada	Manitoba	Churchill	23 km E Churchill, Ram		58.730999	-93.779999	10	
ASWAX967-08	07PROBE-23401	07PROBE-23401	07PROBE-23401	657	GU141069	536	GU141403	07PROBE-23401	Arthropoda	Insecta	Hymenoptera	Braconidae	Microgastrinae	Apanteles	Apanteles jft02	Jose Leonardo Fernandez	jftriana@uoguelph.ca					<VERBATIM LOCALITY>|Ramsay Creek|58.75			A.Renaud	23-Jul-2007		Canada	Manitoba	Churchill	23 km E Churchill, Ram		58.730999	-93.779999	10	
ASWAX968-08	07PROBE-23402	07PROBE-23402	07PROBE-23402	657	GU141320	534	GU141529	07PROBE-23402	Arthropoda	Insecta	Hymenoptera	Braconidae	Microgastrinae	Microplitis	Microplitis varicolor	Jose Leonardo Fernandez	jftriana@uoguelph.ca					<VERBATIM LOCALITY>|Ramsay Creek|58.75			A.Renaud	23-Jul-2007		Canada	Manitoba	Churchill	23 km E Churchill, Ram		58.730999	-93.779999	10	
ASWAX969-08	07PROBE-23403	07PROBE-23403	07PROBE-23403	657	GU141270	469	GU141501	07PROBE-23403	Arthropoda	Insecta	Hymenoptera	Braconidae	Microgastrinae	Microplitis	Microplitis jft04	Jose Leonardo Fernandez	jftriana@uoguelph.ca					<VERBATIM LOCALITY>|Ramsay Creek|58.75			A.Renaud	23-Jul-2007		Canada	Manitoba	Churchill	23 km E Churchill, Ram		58.730999	-93.779999	10	
ASWAX970-08	07PROBE-23404	07PROBE-23404	07PROBE-23404	657	GU141319	536	GU141528	07PROBE-23404	Arthropoda	Insecta	Hymenoptera	Braconidae	Microgastrinae	Microplitis	Microplitis varicolor	Jose Leonardo Fernandez	jftriana@uoguelph.ca					<VERBATIM LOCALITY>|Ramsay Creek|58.75			A.Renaud	23-Jul-2007		Canada	Manitoba	Churchill	23 km E Churchill, Ram		58.730999	-93.779999	10	
ASWAX971-08	07PROBE-23405	07PROBE-23405	07PROBE-23405	657	GU141318	536	GU141527	07PROBE-23405	Arthropoda	Insecta	Hymenoptera	Braconidae	Microgastrinae	Microplitis	Microplitis varicolor	Jose Leonardo Fernandez	jftriana@uoguelph.ca					<VERBATIM LOCALITY>|Ramsay Creek|58.75			A.Renaud	23-Jul-2007		Canada	Manitoba	Churchill	23 km E Churchill, Ram		58.730999	-93.779999	10	
ASWAX972-08	07PROBE-23406	07PROBE-23406	07PROBE-23406	657	GU141251	536	GU141494	07PROBE-23406	Arthropoda	Insecta	Hymenoptera	Braconidae	Microgastrinae	Microplitis	Microplitis jft02	Jose Leonardo Fernandez	jftriana@uoguelph.ca					<VERBATIM LOCALITY>|Ramsay Creek|58.75			A.Renaud	23-Jul-2007		Canada	Manitoba	Churchill	23 km E Churchill, Ram		58.730999	-93.779999	10	

Table S5: All collection and accession information associated with specimens analysed here.

ProcessID	Sample ID	Field Num	Catalog Num	COI-5P Seq.	COI GenBank	28S Seq. Length	28S GenBan	Sample ID	Phylum	Class	Order	Family	Subfamily	Genus	Species	Identifier	Identifier email	Sex	Reproduction	Life Stage	Extra Info	Notes	Voucher Type	Tissue Type	Collectors	Date Collected	Continent	Country/Ocean	Province	Region	Sector	Exact Site	Lat	Lon	Elev	GPS Source
ASWAX973-08	07PROBE-23407	07PROBE-23407	07PROBE-23407	657	GU141278	536	GU141508	07PROBE-23407	Arthropoda	Insecta	Hymenoptera	Braconidae	Microgastrinae	Microplitis	Microplitis jft07	Jose Leonardo Fernandez	jftriana@uoguelph.ca					<VERBATIM LOCALITY>|Ramsay Creek|58.75			A.Renaud	23-Jul-2007		Canada	Manitoba	Churchill	23 km E Churchill, Ram		58.730999	-93.779999	10	
ASWAX974-08	07PROBE-23408	07PROBE-23408	07PROBE-23408	657	GU141250	536	GU141493	07PROBE-23408	Arthropoda	Insecta	Hymenoptera	Braconidae	Microgastrinae	Microplitis	Microplitis jft02	Jose Leonardo Fernandez	jftriana@uoguelph.ca					<VERBATIM LOCALITY>|Ramsay Creek|58.75			A.Renaud	23-Jul-2007		Canada	Manitoba	Churchill	23 km E Churchill, Ram		58.730999	-93.779999	10	
ASWAX975-08	07PROBE-23409	07PROBE-23409	07PROBE-23409	657	GU141054	303	GU141391	07PROBE-23409	Arthropoda	Insecta	Hymenoptera	Braconidae	Microgastrinae	Apanteles	Apanteles jft01	Jose Leonardo Fernandez	jftriana@uoguelph.ca					<VERBATIM LOCALITY>|Ramsay Creek|58.75			A.Renaud	23-Jul-2007		Canada	Manitoba	Churchill	23 km E Churchill, Ram		58.730999	-93.779999	10	
ASWAX976-08	07PROBE-23410	07PROBE-23410	07PROBE-23410	657	GU141277	536	GU141507	07PROBE-23410	Arthropoda	Insecta	Hymenoptera	Braconidae	Microgastrinae	Microplitis	Microplitis jft07	Jose Leonardo Fernandez	jftriana@uoguelph.ca					<VERBATIM LOCALITY>|Ramsay Creek|58.75			A.Renaud	23-Jul-2007		Canada	Manitoba	Churchill	23 km E Churchill, Ram		58.730999	-93.779999	10	
ASWAX977-08	07PROBE-23411	07PROBE-23411	07PROBE-23411	657	GU141068	536	GU141402	07PROBE-23411	Arthropoda	Insecta	Hymenoptera	Braconidae	Microgastrinae	Apanteles	Apanteles jft02	Jose Leonardo Fernandez	jftriana@uoguelph.ca					<VERBATIM LOCALITY>|Ramsay Creek|58.75			A.Renaud	23-Jul-2007		Canada	Manitoba	Churchill	23 km E Churchill, Ram		58.730999	-93.779999	10	
ASWAX978-08	07PROBE-23412	07PROBE-23412	07PROBE-23412	657	GU141317	536	GU141526	07PROBE-23412	Arthropoda	Insecta	Hymenoptera	Braconidae	Microgastrinae	Microplitis	Microplitis varicolor	Jose Leonardo Fernandez	jftriana@uoguelph.ca					<VERBATIM LOCALITY>|Ramsay Creek|58.75			A.Renaud	23-Jul-2007		Canada	Manitoba	Churchill	23 km E Churchill, Ram		58.730999	-93.779999	10	
ASWAX979-08	07PROBE-23413	07PROBE-23413	07PROBE-23413	657	GU141377	536	GU141563	07PROBE-23413	Arthropoda	Insecta	Hymenoptera	Braconidae	Microgastrinae	Pholetesor	Pholetesor viminetorum	Jose Leonardo Fernandez	jftriana@uoguelph.ca					<VERBATIM LOCALITY>|Ramsay Creek|58.75			A.Renaud	23-Jul-2007		Canada	Manitoba	Churchill	23 km E Churchill, Ram		58.730999	-93.779999	10	
ASWAX980-08	07PROBE-23414	07PROBE-23414	07PROBE-23414	657	GU141316	534	GU141525	07PROBE-23414	Arthropoda	Insecta	Hymenoptera	Braconidae	Microgastrinae	Microplitis	Microplitis varicolor	Jose Leonardo Fernandez	jftriana@uoguelph.ca					<VERBATIM LOCALITY>|Ramsay Creek|58.75			A.Renaud	23-Jul-2007		Canada	Manitoba	Churchill	23 km E Churchill, Ram		58.730999	-93.779999	10	
ASWAX981-08	07PROBE-23415	07PROBE-23415	07PROBE-23415	657	GU141249	536	GU141492	07PROBE-23415	Arthropoda	Insecta	Hymenoptera	Braconidae	Microgastrinae	Microplitis	Microplitis jft02	Jose Leonardo Fernandez	jftriana@uoguelph.ca		S	A		<VERBATIM LOCALITY>|Ramsay Creek|58.75			A.Renaud	23-Jul-2007		Canada	Manitoba	Churchill	23 km E Churchill, Ram		58.730999	-93.779999	10	
ASWAX982-08	07PROBE-23416	07PROBE-23416	07PROBE-23416	657	GU141368	380	GU141558	07PROBE-23416	Arthropoda	Insecta	Hymenoptera	Braconidae	Microgastrinae	Pholetesor	Pholetesor salicifoliellae	Jose Leonardo Fernandez	jftriana@uoguelph.ca					<VERBATIM LOCALITY>|Ramsay Creek|58.75			A.Renaud	23-Jul-2007		Canada	Manitoba	Churchill	23 km E Churchill, Ram		58.730999	-93.779999	10	
ASWAX983-08	07PROBE-23417	07PROBE-23417	07PROBE-23417	657	GU141067	536	GU141401	07PROBE-23417	Arthropoda	Insecta	Hymenoptera	Braconidae	Microgastrinae	Apanteles	Apanteles jft02	Jose Leonardo Fernandez	jftriana@uoguelph.ca					<VERBATIM LOCALITY>|Ramsay Creek|58.75			A.Renaud	23-Jul-2007		Canada	Manitoba	Churchill	23 km E Churchill, Ram		58.730999	-93.779999	10	
ASWAX984-08	07PROBE-23418	07PROBE-23418	07PROBE-23418	657	GU141315	536	GU141524	07PROBE-23418	Arthropoda	Insecta	Hymenoptera	Braconidae	Microgastrinae	Microplitis	Microplitis varicolor	Jose Leonardo Fernandez	jftriana@uoguelph.ca					<VERBATIM LOCALITY>|Twin Lakes, 22 km SE			A.Renaud	07-Aug-2007		Canada	Manitoba	Churchill	26 km SE Churchill, Tw		58.632	-93.788002		
ASWAX985-08	07PROBE-23419	07PROBE-23419	07PROBE-23419	657	GU141314	536	GU141523	07PROBE-23419	Arthropoda	Insecta	Hymenoptera	Braconidae	Microgastrinae	Microplitis	Microplitis varicolor	Jose Leonardo Fernandez	jftriana@uoguelph.ca					<VERBATIM LOCALITY>|Twin Lakes, 22 km SE			A.Renaud	07-Aug-2007		Canada	Manitoba	Churchill	26 km SE Churchill, Tw		58.632	-93.788002		
ASWAX986-08	07PROBE-23420	07PROBE-23420	07PROBE-23420	657	GU141099	534	GU141421	07PROBE-23420	Arthropoda	Insecta	Hymenoptera	Braconidae	Microgastrinae	Cotesia	Cotesia jft06	Jose Leonardo Fernandez	jftriana@uoguelph.ca					<VERBATIM LOCALITY>|Twin Lakes, 22 km SE			A.Renaud	07-Aug-2007		Canada	Manitoba	Churchill	26 km SE Churchill, Tw		58.632	-93.788002		
ASWAX987-08	07PROBE-23421	07PROBE-23421	07PROBE-23421	657	GU141313	496	GU141522	07PROBE-23421	Arthropoda	Insecta	Hymenoptera	Braconidae	Microgastrinae	Microplitis	Microplitis varicolor	Jose Leonardo Fernandez	jftriana@uoguelph.ca					<VERBATIM LOCALITY>|Twin Lakes, 22 km SE			A.Renaud	07-Aug-2007		Canada	Manitoba	Churchill	26 km SE Churchill, Tw		58.632	-93.788002		
ASWAX988-08	07PROBE-23422	07PROBE-23422	07PROBE-23422	657	GU141233	536	GU141485	07PROBE-23422	Arthropoda	Insecta	Hymenoptera	Braconidae	Microgastrinae	Microgaster	Microgaster jft10	Jose Leonardo Fernandez	jftriana@uoguelph.ca					<VERBATIM LOCALITY>|Twin Lakes, 22 km SE			A.Renaud	07-Aug-2007		Canada	Manitoba	Churchill	26 km SE Churchill, Tw		58.632	-93.788002		
ASWAX989-08	07PROBE-23423	07PROBE-23423	07PROBE-23423	657	GU141159	536	GU141462	07PROBE-23423	Arthropoda	Insecta	Hymenoptera	Braconidae	Microgastrinae	Dolichogenidea	Dolichogenidea jft03	Jose Leonardo Fernandez	jftriana@uoguelph.ca					<VERBATIM LOCALITY>|Twin Lakes, 22 km SE			A.Renaud	07-Aug-2007		Canada	Manitoba	Churchill	26 km SE Churchill, Tw		58.632	-93.788002		
ASWAX990-08	07PROBE-23424	07PROBE-23424	07PROBE-23424	657	GU141098	535	GU141420	07PROBE-23424	Arthropoda	Insecta	Hymenoptera	Braconidae	Microgastrinae	Cotesia	Cotesia jft06	Jose Leonardo Fernandez	jftriana@uoguelph.ca					<VERBATIM LOCALITY>|Twin Lakes, 22 km SE			A.Renaud	07-Aug-2007		Canada	Manitoba	Churchill	26 km SE Churchill, Tw		58.632	-93.788002		
ASWAX991-08	07PROBE-23425	07PROBE-23425	07PROBE-23425	657	GU141097	536	GU141419	07PROBE-23425	Arthropoda	Insecta	Hymenoptera	Braconidae	Microgastrinae	Cotesia	Cotesia jft06	Jose Leonardo Fernandez	jftriana@uoguelph.ca					<VERBATIM LOCALITY>|Twin Lakes, 22 km SE			A.Renaud	07-Aug-2007		Canada	Manitoba	Churchill	26 km SE Churchill, Tw		58.632	-93.788002		
ASWAX992-08	07PROBE-23426	07PROBE-23426	07PROBE-23426	657	GU141232	536	GU141484	07PROBE-23426	Arthropoda	Insecta	Hymenoptera	Braconidae	Microgastrinae	Microgaster	Microgaster jft10	Jose Leonardo Fernandez	jftriana@uoguelph.ca					<VERBATIM LOCALITY>|Twin Lakes, 22 km SE			A.Renaud	07-Aug-2007		Canada	Manitoba	Churchill	26 km SE Churchill, Tw		58.632	-93.788002		
ASWAX993-08	07PROBE-23427	07PROBE-23427	07PROBE-23427	657	GU141312	519	GU141521	07PROBE-23427	Arthropoda	Insecta	Hymenoptera	Braconidae	Microgastrinae	Microplitis	Microplitis varicolor	Jose Leonardo Fernandez	jftriana@uoguelph.ca					<VERBATIM LOCALITY>|Twin Lakes, 22 km SE			A.Renaud	07-Aug-2007		Canada	Manitoba	Churchill	26 km SE Churchill, Tw		58.632	-93.788002		
ASWAX994-08	07PROBE-23428	07PROBE-23428	07PROBE-23428	657	GU141096	535	GU141418	07PROBE-23428	Arthropoda	Insecta	Hymenoptera	Braconidae	Microgastrinae	Cotesia	Cotesia jft06	Jose Leonardo Fernandez	jftriana@uoguelph.ca					<VERBATIM LOCALITY>|Twin Lakes, 22 km SE			A.Renaud	07-Aug-2007		Canada	Manitoba	Churchill	26 km SE Churchill, Tw		58.632	-93.788002		
ASWAX995-08	07PROBE-23429	07PROBE-23429	07PROBE-23429	657	GU141066	536	GU141400	07PROBE-23429	Arthropoda	Insecta	Hymenoptera	Braconidae	Microgastrinae	Apanteles	Apanteles jft02	Jose Leonardo Fernandez	jftriana@uoguelph.ca					<VERBATIM LOCALITY>|Twin Lakes, 22 km SE			A.Renaud	07-Aug-2007		Canada	Manitoba	Churchill	26 km SE Churchill, Tw		58.632	-93.788002		
ASWAX996-08	07PROBE-23430	07PROBE-23430	07PROBE-23430	657	GU141269	469	GU141500	07PROBE-23430	Arthropoda	Insecta	Hymenoptera	Braconidae	Microgastrinae	Microplitis	Microplitis jft04	Jose Leonardo Fernandez	jftriana@uoguelph.ca					<VERBATIM LOCALITY>|Twin Lakes, 22 km SE			A.Renaud	07-Aug-2007		Canada	Manitoba	Churchill	26 km SE Churchill, Tw		58.632	-93.788002		
ASWAX997-08	07PROBE-23431	07PROBE-23431	07PROBE-23431	657	GU141367	381	GU141557	07PROBE-23431	Arthropoda	Insecta	Hymenoptera	Braconidae	Microgastrinae	Pholetesor	Pholetesor salicifoliellae	Jose Leonardo Fernandez	jftriana@uoguelph.ca					<VERBATIM LOCALITY>|Twin Lakes, 22 km SE			A.Renaud	07-Aug-2007		Canada	Manitoba	Churchill	26 km SE Churchill, Tw		58.632	-93.788002		
ASWAX998-08	07PROBE-23432	07PROBE-23432	07PROBE-23432	657	GU141166	536	GU141466	07PROBE-23432	Arthropoda	Insecta	Hymenoptera	Braconidae	Microgastrinae	Dolichogenidea	Dolichogenidea jft04	Jose Leonardo Fernandez	jftriana@uoguelph.ca					<VERBATIM LOCALITY>|Twin Lakes, 22 km SE			A.Renaud	07-Aug-2007		Canada	Manitoba	Churchill	26 km SE Churchill, Tw		58.632	-93.788002		
ASWAY001-08	07PROBE-23433	07PROBE-23433	07PROBE-23433	657	GU141078	303	GU141407	07PROBE-23433	Arthropoda	Insecta	Hymenoptera	Braconidae	Microgastrinae	Apanteles	Apanteles polychrosidis	Jose Leonardo Fernandez	jftriana@uoguelph.ca					<VERBATIM LOCALITY>|Twin Lakes, 22 km SE			A.Renaud	07-Aug-2007		Canada	Manitoba	Churchill	26 km SE Churchill, Tw		58.632	-93.788002		
ASWAY002-08	07PROBE-23434	07PROBE-23434	07PROBE-23434	657	GU141311	491	GU141520	07PROBE-23434	Arthropoda	Insecta	Hymenoptera	Braconidae	Microgastrinae	Microplitis	Microplitis varicolor	Jose Leonardo Fernandez	jftriana@uoguelph.ca					<VERBATIM LOCALITY>|Twin Lakes, 22 km SE			A.Renaud	07-Aug-2007		Canada	Manitoba	Churchill	26 km SE Churchill, Tw		58.632	-93.788002		
ASWAY003-08	07PROBE-23435	07PROBE-23435	07PROBE-23435	657	GU141231	536	GU141483	07PROBE-23435	Arthropoda	Insecta	Hymenoptera	Braconidae	Microgastrinae	Microgaster	Microgaster jft10	Jose Leonardo Fernandez	jftriana@uoguelph.ca					<VERBATIM LOCALITY>|Twin Lakes, 22 km SE			A.Renaud	07-Aug-2007		Canada	Manitoba	Churchill	26 km SE Churchill, Tw		58.632	-93.788002		
ASWAY004-08	07PROBE-23436	07PROBE-23436	07PROBE-23436	657	GU141310	534	GU141519	07PROBE-23436	Arthropoda	Insecta	Hymenoptera	Braconidae	Microgastrinae	Microplitis	Microplitis varicolor	Jose Leonardo Fernandez	jftriana@uoguelph.ca					<VERBATIM LOCALITY>|Twin Lakes, 22 km SE			A.Renaud	07-Aug-2007		Canada	Manitoba	Churchill	26 km SE Churchill, Tw		58.632	-93.788002		
ASWAY005-08	07PROBE-23437	07PROBE-23437	07PROBE-23437	657	GU141223	375	GU141480	07PROBE-23437	Arthropoda	Insecta	Hymenoptera	Braconidae	Microgastrinae	Illidops	Illidops jft02	Jose Leonardo Fernandez	jftriana@uoguelph.ca					<VERBATIM LOCALITY>|1.8 km S of Twin Golf	NULL		A.Renaud	25-Jun-2007		Canada	Manitoba	Churchill	14 km E Churchill, Golf	1.8 km S of Twin Golf E	58.7519989	-93.910004		
ASWAY006-08	07PROBE-23438	07PROBE-23438	07PROBE-23438	657	GU141193	303	GU141477	07PROBE-23438	Arthropoda	Insecta	Hymenoptera	Braconidae	Microgastrinae	Glyptapanteles	Glyptapanteles jft01	Jose Leonardo Fernandez	jftriana@uoguelph.ca					<VERBATIM LOCALITY>|A-Frame, 12 Km E of			A.Renaud	28-Jun-2007		Canada	Manitoba	Churchill	10 km ESE Churchill, L		58.7550011	-93.998001		
ASWAY007-08	07PROBE-23439	07PROBE-23439	07PROBE-23439	657	GU141113	536	GU141428	07PROBE-23439	Arthropoda	Insecta	Hymenoptera	Braconidae	Microgastrinae	Cotesia	Cotesia jft08	Jose Leonardo Fernandez	jftriana@uoguelph.ca					<VERBATIM LOCALITY>|A-Frame, 12 Km E of			A.Renaud	28-Jun-2007		Canada	Manitoba	Churchill	10 km ESE Churchill, L		58.7550011	-93.998001		
ASWAY008-08	07PROBE-23440	07PROBE-23440	07PROBE-23440	657	GU141309	535	GU141518	07PROBE-23440	Arthropoda	Insecta	Hymenoptera	Braconidae	Microgastrinae	Microplitis	Microplitis varicolor	Jose Leonardo Fernandez	jftriana@uoguelph.ca					<VERBATIM LOCALITY>|A-Frame, 12 Km E of			A.Renaud	28-Jun-2007		Canada	Manitoba	Churchill	10 km ESE Churchill, L		58.7550011	-93.998001		
ASWAY009-08	07PROBE-23441	07PROBE-23441	07PROBE-23441	657	GU141308	536	GU141517	07PROBE-23441	Arthropoda	Insecta	Hymenoptera	Braconidae	Microgastrinae	Microplitis	Microplitis varicolor	Jose Leonardo Fernandez	jftriana@uoguelph.ca					<VERBATIM LOCALITY>|A-Frame, 12 Km E of			A.Renaud	28-Jun-2007		Canada	Manitoba	Churchill	10 km ESE Churchill, L		58.7550011	-93.998001		
ASWAY010-08	07PROBE-23442	07PROBE-23442	07PROBE-23442	585	GU141192	303	GU141476	07PROBE-23442	Arthropoda	Insecta	Hymenoptera	Braconidae	Microgastrinae	Glyptapanteles	Glyptapanteles jft01	Jose Leonardo Fernandez	jftriana@uoguelph.ca					<VERBATIM LOCALITY>|1.8 km S of Twin Golf			A.Renaud	25-Jul-2007		Canada	Manitoba	Churchill	14 km E Churchill, Golf	1.8 km S of Twin Golf E	58.7519989	-93.910004		
ASWAY011-08	07PROBE-23443	07PROBE-23443	07PROBE-23443	657	GU141092	530	GU141415	07PROBE-23443	Arthropoda	Insecta	Hymenoptera	Braconidae	Microgastrinae	Cotesia	Cotesia jft05	Jose Leonardo Fernandez	jftriana@uoguelph.ca					<VERBATIM LOCALITY>|1.8 km S of Twin Golf			A.Renaud	25-Jul-2007		Canada	Manitoba	Churchill	14 km E Churchill, Golf	1.8 km S of Twin Golf E	58.7519989	-93.910004		
ASWAY013-08	07PROBE-23445	07PROBE-23445	07PROBE-23445	657	GU141065	536	GU141399	07PROBE-23445	Arthropoda	Insecta	Hymenoptera	Braconidae	Microgastrinae	Apanteles	Apanteles jft02	Jose Leonardo Fernandez	jftriana@uoguelph.ca					<VERBATIM LOCALITY>|1.8 km S of Twin Golf			A.Renaud	25-Jul-2007		Canada	Manitoba	Churchill	14 km E Churchill, Golf	1.8 km S of Twin Golf E	58.7519989	-93.910004		
ASWAY014-08	07PROBE-23446	07PROBE-23446	07PROBE-23446	657	GU141091	536	GU141414	07PROBE-23446	Arthropoda	Insecta	Hymenoptera	Braconidae	Microgastrinae	Cotesia	Cotesia jft05	Jose Leonardo Fernandez	jftriana@uoguelph.ca					<VERBATIM LOCALITY>|1.8 km S of Twin Golf			A.Renaud	25-Jul-2007		Canada	Manitoba	Churchill	14 km E Churchill, Golf	1.8 km S of Twin Golf E	58.7519989	-93.910004		
ASWAY015-08	07PROBE-23447	07PROBE-23447	07PROBE-23447	657	GU141090	536	GU141413	07PROBE-23447	Arthropoda	Insecta	Hymenoptera	Braconidae	Microgastrinae	Cotesia	Cotesia jft05	Jose Leonardo Fernandez	jftriana@uoguelph.ca					<VERBATIM LOCALITY>|1.8 km S of Twin Golf			A.Renaud	25-Jul-2007		Canada	Manitoba	Churchill	14 km E Churchill, Golf	1.8 km S of Twin Golf E	58.7519989	-93.910004		
ASWAY016-08	07PROBE-23448	07PROBE-23448	07PROBE-23448	657	GU141155	358	GU141459	07PROBE-23448	Arthropoda	Insecta	Hymenoptera	Braconidae	Microgastrinae	Dolichogenidea	Dolichogenidea jft02	Jose Leonardo Fernandez	jftriana@uoguelph.ca					<VERBATIM LOCALITY>|1.8 km S of Twin Golf			A.Renaud	25-Jul-2007		Canada	Manitoba	Churchill	14 km E Churchill, Golf	1.8 km S of Twin Golf E	58.7519989	-93.910004		
ASWAY017-08	07PROBE-23449	07PROBE-23449	07PROBE-23449	657	GU141146	536	GU141450	07PROBE-23449	Arthropoda	Insecta	Hymenoptera	Braconidae	Microgastrinae	Dolichogenidea	Dolichogenidea jft01	Jose Leonardo Fernandez	jftriana@uoguelph.ca					<VERBATIM LOCALITY>|1.8 km S of Twin Golf			A.Renaud	25-Jul-2007		Canada	Manitoba	Churchill	14 km E Churchill, Golf	1.8 km S of Twin Golf E	58.7519989	-93.910004		
ASWAY018-08	07PROBE-23450	07PROBE-23450	07PROBE-23450	657	GU141089	511	GU141412	07PROBE-23450	Arthropoda	Insecta	Hymenoptera	Braconidae	Microgastrinae	Cotesia	Cotesia jft05	Jose Leonardo Fernandez	jftriana@uoguelph.ca					<VERBATIM LOCALITY>|1.8 km S of Twin Golf			A.Renaud	25-Jul-2007		Canada	Manitoba	Churchill	14 km E Churchill, Golf	1.8 km S of Twin Golf E	58.7519989	-93.910004		
ASWAY019-08	07PROBE-23451	07PROBE-23451	07PROBE-23451	657	GU141336	536	GU141542	07PROBE-23451	Arthropoda	Insecta	Hymenoptera	Braconidae	Microgastrinae	Pholetesor	Pholetesor jft01	Jose Leonardo Fernandez	jftriana@uoguelph.ca					<VERBATIM LOCALITY>|1.8 km S of Twin Golf			A.Renaud	25-Jul-2007		Canada	Manitoba	Churchill	14 km E Churchill, Golf	1.8 km S of Twin Golf E	58.7519989	-93.910004		
ASWAY020-08	07PROBE-23452	07PROBE-23452	07PROBE-23452	657	GU141083	536	GU141408	07PROBE-23452	Arthropoda	Insecta	Hymenoptera	Braconidae	Microgastrinae	Cotesia	Cotesia jft04	Jose Leonardo Fernandez	jftriana@uoguelph.ca					<VERBATIM LOCALITY>|A-Frame, 12 Km E of			A.Renaud	05-Jul-2007		Canada	Manitoba	Churchill	10 km ESE Churchill, L		58.7550011	-93.998001		
ASWAY021-08	07PROBE-23453	07PROBE-23453	07PROBE-23453	657	GU141242	536	GU141489	07PROBE-23453	Arthropoda	Insecta	Hymenoptera	Braconidae	Microgastrinae	Microgaster	Microgaster jft06	Jose Leonardo Fernandez	jftriana@uoguelph.ca					<VERBATIM LOCALITY>|A-Frame, 12 Km E of			A.Renaud	05-Jul-2007		Canada	Manitoba	Churchill	10 km ESE Churchill, L		58.7550011	-93.998001		
ASWAY022-08	07PROBE-23454	07PROBE-23454	07PROBE-23454	657	GU141191	303	GU141475	07PROBE-23454	Arthropoda	Insecta	Hymenoptera	Braconidae	Microgastrinae	Glyptapanteles	Glyptapanteles jft01	Jose Leonardo Fernandez	jftriana@uoguelph.ca					<VERBATIM LOCALITY>|A-Frame, 12 Km E of			A.Renaud	05-Jul-2007		Canada	Manitoba	Churchill	10 km ESE Churchill, L		58.7550011	-93.998001		
ASWAY023-08	07PROBE-23455	07PROBE-23455	07PROBE-23455	657	GU141112	536	GU141427	07PROBE-23455	Arthropoda	Insecta	Hymenoptera	Braconidae	Microgastrinae	Cotesia	Cotesia jft08	Jose Leonardo Fernandez	jftriana@uoguelph.ca					<VERBATIM LOCALITY>|A-Frame, 12 Km E of			A.Renaud	05-Jul-2007		Canada	Manitoba	Churchill	10 km ESE Churchill, L		58.7550011	-93.998001		
ASWAY024-08	07PROBE-23456	07PROBE-23456	07PROBE-23456	657	GU141111	536	GU141426	07PROBE-23456	Arthropoda	Insecta	Hymenoptera	Braconidae	Microgastrinae	Cotesia	Cotesia jft08	Jose Leonardo Fernandez	jftriana@uoguelph.ca					<VERBATIM LOCALITY>|A-Frame, 12 Km E of			A.Renaud	05-Jul-2007		Canada	Manitoba	Churchill	10 km ESE Churchill, L		58.7550011	-93.998001		
ASWAY025-08	07PROBE-23457	07PROBE-23457	07PROBE-23457	657	GU141190	303	GU141474	07PROBE-23457	Arthropoda	Insecta	Hymenoptera	Braconidae	Microgastrinae	Glyptapanteles	Glyptapanteles jft01	Jose Leonardo Fernandez	jftriana@uoguelph.ca					<VERBATIM LOCALITY>|A-Frame, 12 Km E of			A.Renaud	05-Jul-2007		Canada	Manitoba	Churchill	10 km ESE Churchill, L		58.7550011	-93.998001		
ASWAY026-08	07PROBE-23458	07PROBE-23458	07PROBE-23458	657	GU141189	303	GU141473	07PROBE-23458	Arthropoda	Insecta	Hymenoptera	Braconidae	Microgastrinae	Glyptapanteles	Glyptapanteles jft01	Jose Leonardo Fernandez	jftriana@uoguelph.ca					<VERBATIM LOCALITY>|A-Frame, 12 Km E of			A.Renaud	05-Jul-2007		Canada	Manitoba	Churchill	10 km ESE Churchill, L		58.7550011	-93.998001		
ASWAY027-08	07PROBE-23459	07PROBE-23459	07PROBE-23459	657	GU141154	358	GU141458	07PROBE-23459	Arthropoda	Insecta	Hymenoptera	Braconidae	Microgastrinae	Dolichogenidea	Dolichogenidea jft02	Jose Leonardo Fernandez	jftriana@uoguelph.ca					<VERBATIM LOCALITY>|A-Frame, 12 Km E of			A.Renaud	05-Jul-2007		Canada	Manitoba	Churchill	10 km ESE Churchill, L		58.7550011	-93.998001		
ASWAY028-08	07PROBE-23460	07PROBE-23460	07PROBE-23460	657	GU141366	381	GU141556	07PROBE-23460	Arthropoda	Insecta	Hymenoptera	Braconidae	Microgastrinae	Pholetesor	Pholetesor salicifoliellae	Jose Leonardo Fernandez	jftriana@uoguelph.ca					<VERBATIM LOCALITY>|A-Frame, 12 Km E of			A.Renaud	05-Jul-2007		Canada	Manitoba	Churchill	10 km ESE Churchill, L		58.7550011	-93.998001		
ASWAY029-08	07PROBE-23461	07PROBE-23461	07PROBE-23461	657	GU141353	381	GU141547	07PROBE-23461	Arthropoda	Insecta	Hymenoptera	Braconidae	Microgastrinae	Pholetesor	Pholetesor ornigis	Jose Leonardo Fernandez	jftriana@uoguelph.ca					<VERBATIM LOCALITY>|A-Frame, 12 Km E of			A.Renaud	05-Jul-2007		Canada	Manitoba	Churchill	10 km ESE Churchill, L		58.7550011	-93.998001		
ASWAY030-08	07PROBE-23462	07PROBE-23462	07PROBE-23462	657	GU141230	536	GU141482	07PROBE-23462	Arthropoda	Insecta	Hymenoptera	Braconidae	Microgastrinae	Microgaster	Microgaster jft10	Jose Leonardo Fernandez	jftriana@uoguelph.ca					<VERBATIM LOCALITY>|A-Frame, 12 Km E of			A.Renaud	05-Jul-2007		Canada	Manitoba	Churchill	10 km ESE Churchill, L		58.7550011	-93.998001		
ASWAY031-08	07PROBE-23463	07PROBE-23463	07PROBE-23463	608	GU141110			07PROBE-23463	Arthropoda	Insecta	Hymenoptera	Braconidae	Microgastrinae	Cotesia	Cotesia jft08	Jose Leonardo Fernandez	jftriana@uoguelph.ca					<VERBATIM LOCALITY>|A-Frame, 12 Km E of			A.Renaud	05-Jul-2007		Canada	Manitoba	Churchill	10 km ESE Churchill, L		58.7550011	-93.998001		
ASWAY032-08	07PROBE-23464	07PROBE-23464	07PROBE-23464	655	GU141243	536	GU141490	07PROBE-23464	Arthropoda	Insecta	Hymenoptera	Braconidae	Microgastrinae	Microgaster	Microgaster jft07	Jose Leonardo Fernandez	jftriana@uoguelph.ca					<VERBATIM LOCALITY>|A-Frame, 12 Km E of			A.Renaud	05-Jul-2007		Canada	Manitoba	Churchill	10 km ESE Churchill, L		58.7550011	-93.998001		
ASWAY033-08	07PROBE-23465	07PROBE-23465	07PROBE-23465	657	GU141220	511	GU141479	07PROBE-23465	Arthropoda	Insecta	Hymenoptera	Braconidae	Microgastrinae	Glyptapanteles	Glyptapanteles jft07	Jose Leonardo Fernandez	jftriana@uoguelph.ca					<VERBATIM LOCALITY>|A-Frame, 12 Km E of			A.Renaud	05-Jul-2007		Canada	Manitoba	Churchill	10 km ESE Churchill, L		58.7550011	-93.998001		
ASWAY034-08	07PROBE-23466	07PROBE-23466	07PROBE-23466	657	GU141188	303	GU141472	07PROBE-23466	Arthropoda	Insecta	Hymenoptera	Braconidae	Microgastrinae	Glyptapanteles	Glyptapanteles jft01	Jose Leonardo Fernandez	jftriana@uoguelph.ca					<VERBATIM LOCALITY>|A-Frame, 12 Km E of			A.Renaud	05-Jul-2007		Canada	Manitoba	Churchill	10 km ESE Churchill, L		58.7550011	-93.998001		
ASWAY507-08	HYM00000751.2	HYM00000751.2	HYM00000751.2	144	GU141720			HYM00000751.2	Arthropoda	Insecta	Hymenoptera	Braconidae	Microgastrinae	Illidops	Illidops jft03	Jose Leonardo Fernandez	jftriana@uoguelph.ca		S	A			NULL		J. G. Chillcot	05-Aug-1952		Canada	Manitoba	Churchill		Fort Churchill	58.7340012	-93.82		
ASWAY508-08	HYM00000894.2	HYM00000894.2	HYM00000894.2	144	GU141715			HYM00000894.2	Arthropoda	Insecta	Hymenoptera	Braconidae	Microgastrinae	Illidops	Illidops jft02	Jose Leonardo Fernandez	jftriana@uoguelph.ca		S	A			NULL		J. G. Chillcot	25-Jun-1952		Canada	Manitoba	Churchill		Fort Churchill	58.7340012	-93.82		
ASWAY509-08	HYM00000895.2	HYM00000895.2	HYM00000895.2	144	GU141719			HYM00000895.2	Arthropoda	Insecta	Hymenoptera	Braconidae	Microgastrinae	Illidops	Illidops jft03	Jose Leonardo Fernandez	jftriana@uoguelph.ca		S	A			NULL		J. G. Chillcot	05-Aug-1952		Canada	Manitoba	Churchill		Fort Churchill	58.7340012	-93.82		
ASWAY510-08	HYM00000903.2	HYM00000903.2	HYM00000903.2	144	GU141714			HYM00000903.2	Arthropoda	Insecta	Hymenoptera	Braconidae	Microgastrinae	Illidops	Illidops jft01	Jose Leonardo Fernandez	jftriana@uoguelph.ca		S	A			NULL		J. G. Chillcot	12-Jul-1952		Canada	Manitoba	Churchill		Fort Churchill	58.7340012	-93.82		
ASWAY511-08	HYM00000901.2	HYM00000901.2	HYM00000901.2	0		0		HYM00000901.2	Arthropoda	Insecta	Hymenoptera	Braconidae	Microgastrinae	Illidops	Illidops jft03	Jose Leonardo Fernandez	jftriana@uoguelph.ca					<VERBATIM LOCALITY>|Fort Churchill|||</VER			J.G.Chillcot	04-Jun-1952		Canada	Manitoba	Churchill	23 km E Churchill, Fort		58.7340012	-93.789001	27	
ASWAY512-08	HYM00000761.2	HYM00000761.2	HYM00000761.2	144	GU141643			HYM00000761.2	Arthropoda	Insecta	Hymenoptera	Braconidae	Microgastrinae	Dolichogenidea	Dolichogenidea jft01	Jose Leonardo Fernandez	jftriana@uoguelph.ca		S	A			NULL		J. G. Chillcot	23-Jul-1952		Canada	Manitoba	Churchill		Fort Churchill	58.7340012	-93.82		
ASWAY513-08	HYM00000763.2	HYM00000763.2	HYM00000763.2	144	GU141642			HYM00000763.2	Arthropoda	Insecta	Hymenoptera	Braconidae	Microgastrinae	Dolichogenidea	Dolichogenidea jft01	Jose Leonardo Fernandez	jftriana@uoguelph.ca		S	A			NULL		J. G. Chillcot	05-Aug-1952		Canada	Manitoba	Churchill		Fort Churchill	58.7340012	-93.82		
ASWAY514-08	HYM00000764.2	HYM00000764.2	HYM00000764.2	129	GU141718			HYM00000764.2	Arthropoda	Insecta	Hymenoptera	Braconidae	Microgastrinae	Illidops	Illidops jft03	Jose Leonardo Fernandez	jftriana@uoguelph.ca		S	A			NULL		P. R. Ehrlich	03-Jun-1952		Canada	Manitoba	Churchill		Fort Churchill	58.7340012	-93.82		
ASWAY515-08	HYM00000762.2	HYM00000762.2	HYM00000762.2	114	GU141717			HYM00000762.2	Arthropoda	Insecta	Hymenoptera	Braconidae	Microgastrinae	Illidops	Illidops jft03	Jose Leonardo Fernandez	jftriana@uoguelph.ca		S	A			NULL		J. G. Chillcot	05-Aug-1952		Canada	Manitoba	Churchill		Fort Churchill	58.7340012	-93.82		
ASWAY516-08	HYM00000750.2	HYM00000750.2	HYM00000750.2	0		0		HYM00000750.2	Arthropoda	Insecta	Hymenoptera	Braconidae	Microgastrinae	Illidops	Illidops jft03	Jose Leonardo Fernandez	jftriana@uoguelph.ca					<VERBATIM LOCALITY>|Churchill|||</VERBAT			S.M.Clark	19-Aug-1959		Canada	Manitoba	Churchill	unspecified locality		58.7400017	-93.82		
ASWAY517-08	HYM00000752.2	HYM00000752.2	HYM00000752.2	144	GU141716			HYM00000752.2	Arthropoda	Insecta	Hymenoptera	Braconidae	Microgastrinae	Illidops	Illidops jft03	Jose Leonardo Fernandez	jftriana@uoguelph.ca		S	A			NULL		J. G. Chillcot	04-Jun-1952		Canada	Manitoba	Churchill		Fort Churchill	58.7340012	-93.82		
ASWAY518-08	HYM00000768.2	HYM00000768.2	HYM00000768.2	0		0		HYM00000768.2	Arthropoda	Insecta	Hymenoptera	Braconidae	Microgastrinae	Illidops	Illidops jft03	Jose Leonardo Fernandez	jftriana@uoguelph.ca					<VERBATIM LOCALITY>|Fort Churchill|||</VER			J.G.Chillcot	09-Jul-1952		Canada	Manitoba	Churchill	23 km E Churchill, Fort		58.7340012	-93.789001	27	
ASWAY519-08	HYM00000665.2	HYM00000665.2	HYM00000665.2	90	GU141045			HYM00000665.2	Arthropoda	Insecta	Hymenoptera	Braconidae	Microgastrinae	Apanteles	Apanteles ensiger	Jose Leonardo Fernandez	jftriana@uoguelph.ca					<VERBATIM LOCALITY>|Churchill (general coo				17-Aug-2006		Canada	Manitoba	Churchill	unspecified locality		58.7400017	-93.82		
ASWAY520-08	HYM00000663.2	HYM00000663.2	HYM00000663.2	90	GU141048			HYM00000663.2	Arthropoda	Insecta	Hymenoptera	Braconidae	Microgastrinae	Apanteles	Apanteles fumiferanae	Jose Leonardo Fernandez	jftriana@uoguelph.ca					<VERBATIM LOCALITY>|Churchill (general coo				17-Aug-2006		Canada	Manitoba	Churchill	unspecified locality		58.7400017	-93.82		
ASWAY521-08	HYM00000770.2	HYM00000770.2	HYM00000770.2	0		0		HYM00000770.2	Arthropoda	Insecta	Hymenoptera	Braconidae	Microgastrinae	Dolichogenidea	Dolichogenidea jft10	Jose Leonardo Fernandez	jftriana@uoguelph.ca					<VERBATIM LOCALITY>|Fort Churchill|||</VER			J.G.Chillcot	18-Jul-1952		Canada	Manitoba	Churchill	23 km E Churchill, Fort		58.7340012	-93.789001	27	
ASWAY522-08	HYM00000754.2	HYM00000754.2	HYM00000754.2	0		0		HYM00000754.2	Arthropoda	Insecta	Hymenoptera	Braconidae	Microgastrinae	Dolichogenidea	Dolichogenidea jft10	Jose Leonardo Fernandez	jftriana@uoguelph.ca					<VERBATIM LOCALITY>|Fort Churchill|||</VER			J.G.Chillcot	18-Jul-1952		Canada	Manitoba	Churchill	23 km E Churchill, Fort		58.7340012	-93.789001	27	
ASWAY523-08	HYM00000771.2	HYM00000771.2	HYM00000771.2	129	GU141667			HYM00000771.2	Arthropoda	Insecta	Hymenoptera	Braconidae	Microgastrinae	Dolichogenidea	Dolichogenidea jft10	Jose Leonardo Fernandez	jftriana@uoguelph.ca		S	A			NULL		J. G. Chillcot	29-Jul-1952		Canada	Manitoba	Churchill		Fort Churchill	58.7340012	-93.82		
ASWAY524-08	HYM00000909.2	HYM00000909.2	HYM00000909.2	144	GU141666			HYM00000909.2	Arthropoda	Insecta	Hymenoptera	Braconidae	Microgastrinae	Dolichogenidea	Dolichogenidea jft10	Jose Leonardo Fernandez	jftriana@uoguelph.ca		S	A			NULL		J. G. Chillcot	28-Jul-1952		Canada	Manitoba	Churchill		Fort Churchill	58.7340012	-93.82		
ASWAY525-08	HYM00000902.2	HYM00000902.2	HYM00000902.2	119	GU141665			HYM00000902.2	Arthropoda	Insecta	Hymenoptera	Braconidae	Microgastrinae	Dolichogenidea	Dolichogenidea jft10	Jose Leonardo Fernandez	jftriana@uoguelph.ca		S	A			NULL		J. G. Chillcot	12-Jul-1952		Canada	Manitoba	Churchill		Fort Churchill	58.7340012	-93.82		
ASWAY526-08	HYM00000904.2	HYM00000904.2	HYM00000904.2	144	GU141664			HYM00000904.2	Arthropoda	Insecta	Hymenoptera	Braconidae	Microgastrinae	Dolichogenidea	Dolichogenidea jft10	Jose Leonardo Fernandez	jftriana@uoguelph.ca		S	A			NULL		J. G. Chillcot	18-Jul-1952		Canada	Manitoba	Churchill		Fort Churchill	58.7340012	-93.82		
ASWAY527-08	HYM00000760.2	HYM00000760.2	HYM00000760.2	144	GU141660			HYM00000760.2	Arthropoda	Insecta	Hymenoptera	Braconidae	Microgastrinae	Dolichogenidea	Dolichogenidea jft08	Jose Leonardo Fernandez	jftriana@uoguelph.ca		S	A			NULL		J. G. Chillcot	05-Aug-1952		Canada	Manitoba	Churchill		Fort Churchill	58.7340012	-93.82		
ASWAY528-08	HYM00000910.2	HYM00000910.2	HYM00000910.2	144	GU141647			HYM00000910.2	Arthropoda	Insecta	Hymenoptera	Braconidae	Microgastrinae	Dolichogenidea	Dolichogenidea jft02	Jose Leonardo Fernandez	jftriana@uoguelph.ca		S	A			NULL		W. J. Brown	29-Jun-1937		Canada	Manitoba	Churchill		Churchill (general coor	58.7799988	-94.185997		
ASWAY529-08	HYM00000907.2	HYM00000907.2	HYM00000907.2	144	GU141646			HYM00000907.2	Arthropoda	Insecta	Hymenoptera	Braconidae	Microgastrinae	Dolichogenidea	Dolichogenidea jft02	Jose Leonardo Fernandez	jftriana@uoguelph.ca		S	A			NULL		J. G. Chillcot	16-Aug-1952		Canada	Manitoba	Churchill		Fort Churchill	58.7340012	-93.82		
ASWAY530-08	HYM00000759.2	HYM00000759.2	HYM00000759.2	144	GU141572			HYM00000759.2	Arthropoda	Insecta	Hymenoptera	Braconidae	Microgastrinae	Apanteles	Apanteles fumiferanae	Jose Leonardo Fernandez	jftriana@uoguelph.ca		S	A					J. G. Chillcot	23-Jun-1952		Canada	Manitoba	Churchill		Fort Churchill	58.7340012	-93.82		
ASWAY531-08	HYM00000856.2	HYM00000856.2	HYM00000856.2	144	GU141645			HYM00000856.2	Arthropoda	Insecta	Hymenoptera	Braconidae	Microgastrinae	Dolichogenidea	Dolichogenidea jft02	Jose Leonardo Fernandez	jftriana@uoguelph.ca		S	A			NULL		W. J. Brown	29-Jun-1937		Canada	Manitoba	Churchill		Churchill (general coor	58.7799988	-94.185997		
ASWAY533-08	HYM00000765.2	HYM00000765.2	HYM00000765.2	0		0		HYM00000765.2	Arthropoda	Insecta	Hymenoptera	Braconidae	Microgastrinae	Dolichogenidea	Dolichogenidea jft07	Jose Leonardo Fernandez	jftriana@uoguelph.ca					<VERBATIM LOCALITY>|Fort Churchill|||</VER			J.G.Chillcot	29-Jul-1952		Canada	Manitoba	Churchill	23 km E Churchill, Fort		58.7340012	-93.789001	27	
ASWAY534-08	HYM00000846.2	HYM00000846.2	HYM00000846.2	90	GU141655			HYM00000846.2	Arthropoda	Insecta	Hymenoptera	Braconidae	Microgastrinae	Dolichogenidea	Dolichogenidea jft07	Jose Leonardo Fernandez	jftriana@uoguelph.ca		S	A			NULL		J. G. Chillcot	08-Jun-1952		Canada	Manitoba	Churchill		Fort Churchill	58.7340012	-93.82		
ASWAY535-08	HYM00000842.2	HYM00000842.2	HYM00000842.2	144	GU141657			HYM00000842.2	Arthropoda	Insecta	Hymenoptera	Braconidae	Microgastrinae	Dolichogenidea	Dolichogenidea jft07	Jose Leonardo Fernandez	jftriana@uoguelph.ca		S	A			NULL		J. G. Chillcot	21-Jul-1952		Canada	Manitoba	Churchill		Fort Churchill	58.7340012	-93.82		
ASWAY536-08	HYM00000845.2	HYM00000845.2	HYM00000845.2	95	GU141654			HYM00000845.2	Arthropoda	Insecta	Hymenoptera	Braconidae	Microgastrinae	Dolichogenidea	Dolichogenidea jft07	Jose Leonardo Fernandez	jftriana@uoguelph.ca		S	A			NULL		J. G. Chillcot	21-Jul-1952		Canada	Manitoba	Churchill		Fort Churchill	58.7340012	-93.82		
ASWAY537-08	HYM00000844.2	HYM00000844.2	HYM00000844.2	144	GU141656			HYM00000844.2	Arthropoda	Insecta	Hymenoptera	Braconidae	Microgastrinae	Dolichogenidea	Dolichogenidea jft07	Jose Leonardo Fernandez	jftriana@uoguelph.ca		S	A			NULL		J. G. Chillcot	08-Jun-1952		Canada	Manitoba	Churchill		Fort Churchill	58.7340012	-93.82		
ASWAY538-08	HYM00000848.2	HYM00000848.2	HYM00000848.2	144	GU141662			HYM00000848.2	Arthropoda	Insecta	Hymenoptera	Braconidae	Microgastrinae	Dolichogenidea	Dolichogenidea jft09	Jose Leonardo Fernandez	jftriana@uoguelph.ca		S	A			NULL		J. G. Chillcot	27-Aug-1950		Canada	Manitoba	Churchill		Fort Churchill	58.7340012	-93.82		
ASWAY539-08	HYM00000843.2	HYM00000843.2	HYM00000843.2	90	GU141653			HYM00000843.2	Arthropoda	Insecta	Hymenoptera	Braconidae	Microgastrinae	Dolichogenidea	Dolichogenidea jft07	Jose Leonardo Fernandez	jftriana@uoguelph.ca		S	A			NULL		J. G. Chillcot	21-Jul-1952		Canada	Manitoba	Churchill		Fort Churchill	58.7340012	-93.82		
ASWAY540-08	HYM00000847.2	HYM00000847.2	HYM00000847.2	84	GU141652			HYM00000847.2	Arthropoda	Insecta	Hymenoptera	Braconidae	Microgastrinae	Dolichogenidea	Dolichogenidea jft07	Jose Leonardo Fernandez	jftriana@uoguelph.ca		S	A			NULL		J. G. Chillcot	08-Jun-1952		Canada	Manitoba	Churchill		Fort Churchill	58.7340012	-93.82		
ASWAY541-08	HYM00000854.2	HYM00000854.2	HYM00000854.2	144	GU141659			HYM00000854.2	Arthropoda	Insecta	Hymenoptera	Braconidae	Microgastrinae	Dolichogenidea	Dolichogenidea jft08	Jose Leonardo Fernandez	jftriana@uoguelph.ca		S	A					J. G. Chillcot	08-Jun-1952		Canada	Manitoba	Churchill		Fort Churchill	58.7340012	-93.82		
ASWAY544-08	HYM00000758.2	HYM00000758.2	HYM00000758.2	84	GU141658			HYM00000758.2	Arthropoda	Insecta	Hymenoptera	Braconidae	Microgastrinae	Dolichogenidea	Dolichogenidea jft08	Jose Leonardo Fernandez	jftriana@uoguelph.ca		S	A			NULL		J. G. Chillcot	21-Jul-1952		Canada	Manitoba	Churchill		Fort Churchill	58.7340012	-93.82		
ASWAY546-08	HYM00000683.2	HYM00000683.2	HYM00000683.2	631	GU141169			HYM00000683.2	Arthropoda	Insecta	Hymenoptera	Braconidae	Microgastrinae	Dolichogenidea	Dolichogenidea jft05	Jose Leonardo Fernandez	jftriana@uoguelph.ca					<VERBATIM LOCALITY>|Burn Site|58.371|-93.4				15-Aug-2006		Canada	Manitoba	Churchill	26 km SE Churchill, Tw		58.6170006	-93.811996	38	
ASWAY547-08	HYM00000686.2	HYM00000686.2	HYM00000686.2	631	GU141168			HYM00000686.2	Arthropoda	Insecta	Hymenoptera	Braconidae	Microgastrinae	Dolichogenidea	Dolichogenidea jft05	Jose Leonardo Fernandez	jftriana@uoguelph.ca					<VERBATIM LOCALITY>|Burn Site|58.371|-93.4				15-Aug-2006		Canada	Manitoba	Churchill	26 km SE Churchill, Tw		58.6170006	-93.811996	38	
ASWAY548-08	HYM00000852.2	HYM00000852.2	HYM00000852.2	129	GU141651			HYM00000852.2	Arthropoda	Insecta	Hymenoptera	Braconidae	Microgastrinae	Dolichogenidea	Dolichogenidea jft03	Jose Leonardo Fernandez	jftriana@uoguelph.ca		S	A			NULL		L. A. Miller	05-Sep-1948		Canada	Manitoba	Churchill		Churchill (general coor	58.7799988	-94.185997		
ASWAY549-08	HYM00000900.2	HYM00000900.2	HYM00000900.2	144	GU141650			HYM00000900.2	Arthropoda	Insecta	Hymenoptera	Braconidae	Microgastrinae	Dolichogenidea	Dolichogenidea jft03	Jose Leonardo Fernandez	jftriana@uoguelph.ca		S	A			NULL		J. G. Chillcot	03-Aug-1952		Canada	Manitoba	Churchill		Fort Churchill	58.7340012	-93.82		
ASWAY550-08	HYM00000853.2	HYM00000853.2	HYM00000853.2	0		0		HYM00000853.2	Arthropoda	Insecta	Hymenoptera	Braconidae	Microgastrinae	Dolichogenidea	Dolichogenidea jft01	Jose Leonardo Fernandez	jftriana@uoguelph.ca					<VERBATIM LOCALITY>|Churchill|||</VERBAT			W.J.Brown	08-Jul-1937		Canada	Manitoba	Churchill	unspecified locality		58.7400017	-93.82		
ASWAY551-08	HYM00000855.2	HYM00000855.2	HYM00000855.2	129	GU141649			HYM00000855.2	Arthropoda	Insecta	Hymenoptera	Braconidae	Microgastrinae	Dolichogenidea	Dolichogenidea jft03	Jose Leonardo Fernandez	jftriana@uoguelph.ca		S	A			NULL		W. J. Brown	08-Jul-1937		Canada	Manitoba	Churchill		Churchill (general coor	58.7799988	-94.185997		
ASWAY552-08	HYM00000859.2	HYM00000859.2	HYM00000859.2	144	GU141641			HYM00000859.2	Arthropoda	Insecta	Hymenoptera	Braconidae	Microgastrinae	Dolichogenidea	Dolichogenidea jft01	Jose Leonardo Fernandez	jftriana@uoguelph.ca		S	A					J. G. Chillcot	11-Jun-1952		Canada	Manitoba	Churchill		Fort Churchill	58.7340012	-93.82		
ASWAY553-08	HYM00000857.2	HYM00000857.2	HYM00000857.2	617	GU141640			HYM00000857.2	Arthropoda	Insecta	Hymenoptera	Braconidae	Microgastrinae	Dolichogenidea	Dolichogenidea jft01	Jose Leonardo Fernandez	jftriana@uoguelph.ca		S	A					J. G. Chillcot	11-Jun-1952		Canada	Manitoba	Churchill		Fort Churchill	58.7340012	-93.82		
ASWAY554-08	HYM00000769.2	HYM00000769.2	HYM00000769.2	144	GU141639			HYM00000769.2	Arthropoda	Insecta	Hymenoptera	Braconidae	Microgastrinae	Dolichogenidea	Dolichogenidea jft01	Jose Leonardo Fernandez	jftriana@uoguelph.ca		S	A			NULL		J. G. Chillcot	23-Jul-1952		Canada	Manitoba	Churchill		Fort Churchill	58.7340012	-93.82		
ASWAY556-08	HYM00000899.2	HYM00000899.2	HYM00000899.2	144	GU141638			HYM00000899.2	Arthropoda	Insecta	Hymenoptera	Braconidae	Microgastrinae	Dolichogenidea	Dolichogenidea jft01	Jose Leonardo Fernandez	jftriana@uoguelph.ca		S	A					J. G. Chillcot	03-Aug-1952		Canada	Manitoba	Churchill		Fort Churchill	58.7340012	-93.82		
ASWAY557-08	HYM00000858.2	HYM00000858.2	HYM00000858.2	144	GU141637			HYM00000858.2	Arthropoda	Insecta	Hymenoptera	Braconidae	Microgastrinae	Dolichogenidea	Dolichogenidea jft01	Jose Leonardo Fernandez	jftriana@uoguelph.ca		S	A					J. G. Chillcot	18-Jun-1952		Canada	Manitoba	Churchill		Fort Churchill	58.7340012	-93.82		
ASWAY558-08	HYM00000851.2	HYM00000851.2	HYM00000851.2	293	GU141609			HYM00000851.2	Arthropoda	Insecta	Hymenoptera	Braconidae	Microgastrinae	Cotesia	Cotesia yakutatensis	Jose Leonardo Fernandez	jftriana@uoguelph.ca		S	A					J. G. Chillcot	12-Jul-1952		Canada	Manitoba	Churchill		Fort Churchill	58.7340012	-93.82		
ASWAY559-08	HYM00000850.2	HYM00000850.2	HYM00000850.2	144	GU141648			HYM00000850.2	Arthropoda	Insecta	Hymenoptera	Braconidae	Microgastrinae	Dolichogenidea	Dolichogenidea jft03	Jose Leonardo Fernandez	jftriana@uoguelph.ca		S	A			NULL		J. G. Chillcot	29-Jun-1952		Canada	Manitoba	Churchill		Warkworth Creek nr. Cl	58.375	-93.875		
ASWAY560-08	HYM00000849.2	HYM00000849.2	HYM00000849.2	129	GU141663			HYM00000849.2	Arthropoda	Insecta	Hymenoptera	Braconidae	Microgastrinae	Dolichogenidea	Dolichogenidea jft10	Jose Leonardo Fernandez	jftriana@uoguelph.ca		S	A					J. G. Chillcot	12-Jul-1952		Canada	Manitoba	Churchill		Fort Churchill	58.7340012	-93.82		
ASWAY563-08	HYM00000820.2	HYM00000820.2	HYM00000820.2	0		0		HYM00000820.2	Arthropoda	Insecta	Hymenoptera	Braconidae	Microgastrinae	Pholetesor	Pholetesor bedelliae	Jose Leonardo Fernandez	jftriana@uoguelph.ca					<VERBATIM LOCALITY>|Mile 505, Hudson Bay			J.G.Chillcot	16-Jul-1952		Canada	Manitoba	Churchill	Mile 505, Hudson Bay F					
ASWAY564-08	HYM00000821.2	HYM00000821.2	HYM00000821.2	0		0		HYM00000821.2	Arthropoda	Insecta	Hymenoptera	Braconidae	Microgastrinae	Pholetesor	Pholetesor bedelliae	Jose Leonardo Fernandez	jftriana@uoguelph.ca					<VERBATIM LOCALITY>|Churchill|||</VERBAT			R.Gwatkin	17-Jul-1949		Canada	Manitoba	Churchill	unspecified locality		58.7400017	-93.82		
ASWAY565-08	HYM00000912.2	HYM00000912.2	HYM00000912.2	144	GU141767			HYM00000912.2	Arthropoda	Insecta	Hymenoptera	Braconidae	Microgastrinae	Microgaster	Microgaster jft09	Jose Leonardo Fernandez	jftriana@uoguelph.ca		S	A					J. G. Chillcot	21-Jun-1952		Canada	Manitoba	Churchill		Fort Churchill	58.7340012	-93.82		
ASWAY566-08	HYM00000913.2	HYM00000913.2	HYM00000913.2	558	GU141706			HYM00000913.2	Arthropoda	Insecta	Hymenoptera	Braconidae				Jose Leonardo Fernandez	jftriana@uoguelph.ca		S	A					W. J. Brown	21-Jul-1937		Canada	Manitoba	Churchill		Churchill (general coor	58.7799988	-94.185997		
ASWAY567-08	HYM00000917.2	HYM00000917.2	HYM00000917.2	144	GU141766			HYM00000917.2	Arthropoda	Insecta	Hymenoptera	Braconidae	Microgastrinae	Microgaster	Microgaster jft08	Jose Leonardo Fernandez	jftriana@uoguelph.ca		S	A			NULL		J. G. Chillcot	11-Jul-1952		Canada	Manitoba	Churchill		Fort Churchill	58.7340012	-93.82		
ASWAY568-08	HYM00000667.2	HYM00000667.2	HYM00000667.2	616	GU141088			HYM00000667.2	Arthropoda	Insecta	Hymenoptera	Braconidae	Microgastrinae	Cotesia	Cotesia jft05	Jose Leonardo Fernandez	jftriana@uoguelph.ca					<VERBATIM LOCALITY>|Churchill (general coo				17-Aug-2006		Canada	Manitoba	Churchill	unspecified locality		58.7400017	-93.82		
ASWAY569-08	CHU05-WSP-016.2	CHU05-WSP-016.2	CHU05-WSP-016.2	631	GU141087			CHU05-WSP-016.2	Arthropoda	Insecta	Hymenoptera	Braconidae	Microgastrinae	Cotesia	Cotesia jft05	Jose Leonardo Fernandez	jftriana@uoguelph.ca					<VERBATIM LOCALITY>|23 km E of Churchill,			C.Sheffield	01-Aug-2005		Canada	Manitoba	Churchill	22 km E Churchill, CNS	23 km E of Churchill,	58.737999	-93.819		
ASWAY570-08	HYM00000930.2	HYM00000930.2	HYM00000930.2	320	GU141608			HYM00000930.2	Arthropoda	Insecta	Hymenoptera	Braconidae	Microgastrinae	Cotesia	Cotesia jft10	Jose Leonardo Fernandez	jftriana@uoguelph.ca		S	A			NULL		J. G. Chillcot	28-Jun-1952		Canada	Manitoba	Churchill		Fort Churchill	58.7340012	-93.82		
ASWAY571-08	HYM00000731.2	HYM00000731.2	HYM00000731.2	144	GU141605			HYM00000731.2	Arthropoda	Insecta	Hymenoptera	Braconidae	Microgastrinae	Cotesia	Cotesia jft09	Jose Leonardo Fernandez	jftriana@uoguelph.ca		S	A					W. J. Brown	01-Aug-1937		Canada	Manitoba	Churchill		Churchill (general coor	58.7799988	-94.185997		
ASWAY572-08	HYM00000732.2	HYM00000732.2	HYM00000732.2	144	GU141604			HYM00000732.2	Arthropoda	Insecta	Hymenoptera	Braconidae	Microgastrinae	Cotesia	Cotesia jft09	Jose Leonardo Fernandez	jftriana@uoguelph.ca		S	A					W. J. Brown	01-Aug-1937		Canada	Manitoba	Churchill		Churchill (general coor	58.7799988	-94.185997		
ASWAY573-08	HYM00000733.2	HYM00000733.2	HYM00000733.2	129	GU141603			HYM00000733.2	Arthropoda	Insecta	Hymenoptera	Braconidae	Microgastrinae	Cotesia	Cotesia jft09	Jose Leonardo Fernandez	jftriana@uoguelph.ca		S	A					W. J. Brown	01-Aug-1937		Canada	Manitoba	Churchill		Churchill (general coor	58.7799988	-94.185997		
ASWAY574-08	HYM00000734.2	HYM00000734.2	HYM00000734.2	0		0		HYM00000734.2	Arthropoda	Insecta	Hymenoptera	Braconidae	Microgastrinae	Cotesia	Cotesia jft09	Jose Leonardo Fernandez	jftriana@uoguelph.ca					<VERBATIM LOCALITY>|Churchill|||</VERBAT			W.J.Brown	01-Aug-1937		Canada	Manitoba	Churchill	unspecified locality		58.7400017	-93.82		
ASWAY575-08	HYM00000735.2	HYM00000735.2	HYM00000735.2	129	GU141602			HYM00000735.2	Arthropoda	Insecta	Hymenoptera	Braconidae	Microgastrinae	Cotesia	Cotesia jft09	Jose Leonardo Fernandez	jftriana@uoguelph.ca		S	A					W. J. Brown	01-Aug-1937		Canada	Manitoba	Churchill		Churchill (general coor	58.7799988	-94.185997		
ASWAY576-08	HYM00000736.2	HYM00000736.2	HYM00000736.2	144	GU141601			HYM00000736.2	Arthropoda	Insecta	Hymenoptera	Braconidae	Microgastrinae	Cotesia	Cotesia jft09	Jose Leonardo Fernandez	jftriana@uoguelph.ca		S	A					W. J. Brown	01-Aug-1937		Canada	Manitoba	Churchill		Churchill (general coor	58.7799988	-94.185997		
ASWAY577-08	HYM00000737.2	HYM00000737.2	HYM00000737.2	0		0		HYM00000737.2	Arthropoda	Insecta	Hymenoptera	Braconidae	Microgastrinae	Cotesia	Cotesia jft09	Jose Leonardo Fernandez	jftriana@uoguelph.ca					<VERBATIM LOCALITY>|Churchill|||</VERBAT			W.J.Brown	01-Aug-1937		Canada	Manitoba	Churchill	unspecified locality		58.7400017	-93.82		
ASWAY578-08	HYM00000738.2	HYM00000738.2	HYM00000738.2	0		0		HYM00000738.2	Arthropoda	Insecta	Hymenoptera	Braconidae	Microgastrinae	Cotesia	Cotesia jft09	Jose Leonardo Fernandez	jftriana@uoguelph.ca					<VERBATIM LOCALITY>|Churchill|||</VERBAT			W.J.Brown	01-Aug-1937		Canada	Manitoba	Churchill	unspecified locality		58.7400017	-93.82		
ASWAY579-08	HYM00000739.2	HYM00000739.2	HYM00000739.2	0		0		HYM00000739.2	Arthropoda	Insecta	Hymenoptera	Braconidae	Microgastrinae	Cotesia	Cotesia jft09	Jose Leonardo Fernandez	jftriana@uoguelph.ca					<VERBATIM LOCALITY>|Churchill|||</VERBAT			W.J.Brown	01-Aug-1937		Canada	Manitoba	Churchill	unspecified locality		58.7400017	-93.82		
ASWAY580-08	HYM00000929.2	HYM00000929.2	HYM00000929.2	144	GU141600			HYM00000929.2	Arthropoda	Insecta	Hymenoptera	Braconidae	Microgastrinae	Cotesia	Cotesia jft09	Jose Leonardo Fernandez	jftriana@uoguelph.ca		S	A					W. J. Brown	01-Aug-1937		Canada	Manitoba	Churchill		Churchill (general coor	58.7799988	-94.185997		
ASWAY581-08	HYM00000748.2	HYM00000748.2	HYM00000748.2	631	GU141133			HYM00000748.2	Arthropoda	Insecta	Hymenoptera	Braconidae	Microgastrinae	Cotesia	Cotesia xylina	Jose Leonardo Fernandez	jftriana@uoguelph.ca					<VERBATIM LOCALITY>|Burn Site|58.371|-93.4				17-Aug-2006		Canada	Manitoba	Churchill	26 km SE Churchill, Tw		58.6170006	-93.811996	38	
ASWAY582-08	HYM00000749.2	HYM00000749.2	HYM00000749.2	616	GU141095			HYM00000749.2	Arthropoda	Insecta	Hymenoptera	Braconidae	Microgastrinae	Cotesia	Cotesia jft06	Jose Leonardo Fernandez	jftriana@uoguelph.ca					<VERBATIM LOCALITY>|Burn Site|58.371|-93.4				17-Aug-2006		Canada	Manitoba	Churchill	26 km SE Churchill, Tw		58.6170006	-93.811996	38	
ASWAY583-08	HYM00000658.2	HYM00000658.2	HYM00000658.2	631	GU141139			HYM00000658.2	Arthropoda	Insecta	Hymenoptera	Braconidae	Microgastrinae	Cotesia	Cotesia yakutatensis	Jose Leonardo Fernandez	jftriana@uoguelph.ca					<VERBATIM LOCALITY>|Burn Site|58.371|-93.4				20-Aug-2006		Canada	Manitoba	Churchill	26 km SE Churchill, Tw		58.6170006	-93.811996	38	
ASWAY584-08	HYM00000747.2	HYM00000747.2	HYM00000747.2	320	GU141138			HYM00000747.2	Arthropoda	Insecta	Hymenoptera	Braconidae	Microgastrinae	Cotesia	Cotesia yakutatensis	Jose Leonardo Fernandez	jftriana@uoguelph.ca					<VERBATIM LOCALITY>|Burn Site|58.371|-93.4				17-Aug-2006		Canada	Manitoba	Churchill	26 km SE Churchill, Tw		58.6170006	-93.811996	38	
ASWAY585-08	HYM00000652.2	HYM00000652.2	HYM00000652.2	631	GU141079			HYM00000652.2	Arthropoda	Insecta	Hymenoptera	Braconidae	Microgastrinae	Cotesia	Cotesia jft02	Jose Leonardo Fernandez	jftriana@uoguelph.ca					<VERBATIM LOCALITY>|Burn Site|58.371|-93.4				20-Aug-2006		Canada	Manitoba	Churchill	26 km SE Churchill, Tw		58.6170006	-93.811996	38	
ASWAY586-08	HYM00000653.2	HYM00000653.2	HYM00000653.2	631	GU141137			HYM00000653.2	Arthropoda	Insecta	Hymenoptera	Braconidae	Microgastrinae	Cotesia	Cotesia yakutatensis	Jose Leonardo Fernandez	jftriana@uoguelph.ca					<VERBATIM LOCALITY>|Burn Site|58.371|-93.4				20-Aug-2006		Canada	Manitoba	Churchill	26 km SE Churchill, Tw		58.6170006	-93.811996	38	
ASWAY587-08	HYM00000745.2	HYM00000745.2	HYM00000745.2	144	GU141607			HYM00000745.2	Arthropoda	Insecta	Hymenoptera	Braconidae	Microgastrinae	Cotesia	Cotesia jft10	Jose Leonardo Fernandez	jftriana@uoguelph.ca		S	A			NULL		J. G. Chillcot	29-Jun-1952		Canada	Manitoba	Churchill		Warkworth Creek nr. Cl	58.375	-93.875		
ASWAY588-08	HYM00000744.2	HYM00000744.2	HYM00000744.2	129	GU141594			HYM00000744.2	Arthropoda	Insecta	Hymenoptera	Braconidae	Microgastrinae	Cotesia	Cotesia jft08	Jose Leonardo Fernandez	jftriana@uoguelph.ca		S	A			NULL		J. G. Chillcot	29-Jun-1952		Canada	Manitoba	Churchill		Warkworth Creek nr. Cl	58.375	-93.875		
ASWAY589-08	HYM00000705.2	HYM00000705.2	HYM00000705.2	144	GU141764			HYM00000705.2	Arthropoda	Insecta	Hymenoptera	Braconidae	Microgastrinae	Microgaster	Microgaster deductor	Jose Leonardo Fernandez	jftriana@uoguelph.ca		S	A					J. G. Chillcot	18-Jul-1952		Canada	Manitoba	Churchill		Fort Churchill	58.7340012	-93.82		
ASWAY590-08	HYM00000792.2	HYM00000792.2	HYM00000792.2	305	GU141819			HYM00000792.2	Arthropoda	Insecta	Hymenoptera	Braconidae	Microgastrinae	Pholetesor	Pholetesor viminetorum	Jose Leonardo Fernandez	jftriana@uoguelph.ca		S	A					J. G. Chillcot	21-Jul-1952		Canada	Manitoba	Churchill		Fort Churchill	58.7340012	-93.82		
ASWAY591-08	HYM00000793.2	HYM00000793.2	HYM00000793.2	0		0		HYM00000793.2	Arthropoda	Insecta	Hymenoptera	Braconidae	Microgastrinae	Pholetesor	Pholetesor viminetorum	Jose Leonardo Fernandez	jftriana@uoguelph.ca					<VERBATIM LOCALITY>|Fort Churchill|||</VER			J.G.Chillcot	21-Jul-1952		Canada	Manitoba	Churchill	23 km E Churchill, Fort		58.7340012	-93.789001	27	
ASWAY592-08	HYM00000921.2	HYM00000921.2	HYM00000921.2	144	GU141818			HYM00000921.2	Arthropoda	Insecta	Hymenoptera	Braconidae	Microgastrinae	Pholetesor	Pholetesor viminetorum	Jose Leonardo Fernandez	jftriana@uoguelph.ca		S	A					J. G. Chillcot	28-Jul-1952		Canada	Manitoba	Churchill		Fort Churchill	58.7340012	-93.82		
ASWAY593-08	HYM00000816.2	HYM00000816.2	HYM00000816.2	144	GU141644			HYM00000816.2	Arthropoda	Insecta	Hymenoptera	Braconidae	Microgastrinae	Dolichogenidea	Dolichogenidea jft02	Jose Leonardo Fernandez	jftriana@uoguelph.ca		S	A			NULL		J. G. Chillcot	05-Aug-1952		Canada	Manitoba	Churchill		Warkworth Creek nr. Cl	58.375	-93.875		
ASWAY594-08	HYM00000815.2	HYM00000815.2	HYM00000815.2	0		0		HYM00000815.2	Arthropoda	Insecta	Hymenoptera	Braconidae	Microgastrinae	Pholetesor	Pholetesor viminetorum	Jose Leonardo Fernandez	jftriana@uoguelph.ca					<VERBATIM LOCALITY>|Fort Churchill|||</VER	NULL		J.G.Chillcot	01-Jul-1952		Canada	Manitoba	Churchill	23 km E Churchill, Fort		58.7340012	-93.789001	27	
ASWAY595-08	HYM00000814.2	HYM00000814.2	HYM00000814.2	0		0		HYM00000814.2	Arthropoda	Insecta	Hymenoptera	Braconidae	Microgastrinae	Pholetesor	Pholetesor viminetorum	Jose Leonardo Fernandez	jftriana@uoguelph.ca					<VERBATIM LOCALITY>|Fort Churchill|||</VER			J.G.Chillcot	21-Jul-1952		Canada	Manitoba	Churchill	23 km E Churchill, Fort		58.7340012	-93.789001	27	
ASWAY596-08	HYM00000813.2	HYM00000813.2	HYM00000813.2	0		0		HYM00000813.2	Arthropoda	Insecta	Hymenoptera	Braconidae	Microgastrinae	Pholetesor	Pholetesor viminetorum	Jose Leonardo Fernandez	jftriana@uoguelph.ca					<VERBATIM LOCALITY>|Fort Churchill|||</VER			J.G.Chillcot	11-Jul-1952		Canada	Manitoba	Churchill	23 km E Churchill, Fort		58.7340012	-93.789001	27	
ASWAY597-08	HYM00000812.2	HYM00000812.2	HYM00000812.2	144	GU141606			HYM00000812.2	Arthropoda	Insecta	Hymenoptera	Braconidae	Microgastrinae	Cotesia	Cotesia jft10	Jose Leonardo Fernandez	jftriana@uoguelph.ca		S	A			NULL		J. G. Chillcot	11-Jul-1952		Canada	Manitoba	Churchill		Fort Churchill	58.7340012	-93.82		
ASWAY598-08	HYM00000811.2	HYM00000811.2	HYM00000811.2	0		0		HYM00000811.2	Arthropoda	Insecta	Hymenoptera	Braconidae	Microgastrinae	Pholetesor	Pholetesor viminetorum	Jose Leonardo Fernandez	jftriana@uoguelph.ca					<VERBATIM LOCALITY>|Fort Churchill|||</VER			J.G.Chillcot	11-Jul-1952		Canada	Manitoba	Churchill	23 km E Churchill, Fort		58.7340012	-93.789001	27	
ASWAY599-08	HYM00000810.2	HYM00000810.2	HYM00000810.2	129	GU141661			HYM00000810.2	Arthropoda	Insecta	Hymenoptera	Braconidae	Microgastrinae	Dolichogenidea	Dolichogenidea jft09	Jose Leonardo Fernandez	jftriana@uoguelph.ca		S	A			NULL		J. G. Chillcot	24-Jul-1952		Canada	Manitoba	Churchill		Fort Churchill	58.7340012	-93.82		
ASWAY600-08	HYM00000809.2	HYM00000809.2	HYM00000809.2	100	GU141817			HYM00000809.2	Arthropoda	Insecta	Hymenoptera	Braconidae	Microgastrinae	Pholetesor	Pholetesor viminetorum	Jose Leonardo Fernandez	jftriana@uoguelph.ca		S	A					J. G. Chillcot	11-Jul-1952		Canada	Manitoba	Churchill		Fort Churchill	58.7340012	-93.82		
ASWAY601-08	HYM00000684.2	HYM00000684.2	HYM00000684.2	631	GU141158			HYM00000684.2	Arthropoda	Insecta	Hymenoptera	Braconidae	Microgastrinae	Dolichogenidea	Dolichogenidea jft03	Jose Leonardo Fernandez	jftriana@uoguelph.ca					<VERBATIM LOCALITY>|Burn Site|58.371|-93.4				15-Aug-2006		Canada	Manitoba	Churchill	26 km SE Churchill, Tw		58.6170006	-93.811996	38	
ASWAY602-08	HYM00000740.2	HYM00000740.2	HYM00000740.2	0				HYM00000740.2	Arthropoda	Insecta	Hymenoptera	Braconidae	Microgastrinae	Cotesia	Cotesia xylina	Jose Leonardo Fernandez	jftriana@uoguelph.ca		S	A					J. G. Chillcot	13-Jun-1952		Canada	Manitoba	Churchill		Fort Churchill				
ASWAY603-08	HYM00000741.2	HYM00000741.2	HYM00000741.2	0		0		HYM00000741.2	Arthropoda	Insecta	Hymenoptera	Braconidae	Microgastrinae	Cotesia	Cotesia xylina	Jose Leonardo Fernandez	jftriana@uoguelph.ca		S	A					J. G. Chillcot	05-Aug-1952		Canada	Manitoba	Churchill		Fort Churchill				

Table S5: All collection and accession information associated with specimens analysed here.

ProcessID	Sample ID	Field Num	Catalog Num	COI-5P Seq.	COI GenBank	28S Seq. Length	28S GenBan	Sample ID	Phylum	Class	Order	Family	Subfamily	Genus	Species	Identifier	Identifier email	Sex	Reproduction	Life Stage	Extra Info	Notes	Voucher Type	Tissue Type	Collectors	Date Collected	Continent	Country/Ocean	Province	Region	Sector	Exact Site	Lat	Lon	Elev	GPS Source
ASWAY604-08	HYM00000766.2	HYM00000766.2	HYM00000766.2	0		0		HYM00000766.2	Arthropoda	Insecta	Hymenoptera	Braconidae	Microgastrinae	Cotesia	Cotesia xylina	Jose Leonardo Fernandez	jftriana@uoguelph.ca		S	A					J. G. Chillcot	29-Jun-1952		Canada	Manitoba	Churchill		Warkworth Creek nr. Cl				
ASWAY605-08	HYM00000746.2	HYM00000746.2	HYM00000746.2	0		0		HYM00000746.2	Arthropoda	Insecta	Hymenoptera	Braconidae	Microgastrinae	Cotesia	Cotesia yakutatensis	Jose Leonardo Fernandez	jftriana@uoguelph.ca		S	A					J. G. Chillcot	05-Aug-1952		Canada	Manitoba	Churchill		Fort Churchill				
ASWAY606-08	HYM00000742.2	HYM00000742.2	HYM00000742.2	0		0		HYM00000742.2	Arthropoda	Insecta	Hymenoptera	Braconidae	Microgastrinae	Cotesia	Cotesia xylina	Jose Leonardo Fernandez	jftriana@uoguelph.ca		S	A					J. G. Chillcot	03-Aug-1952		Canada	Manitoba	Churchill		Fort Churchill				
ASWAY607-08	HYM00000831.2	HYM00000831.2	HYM00000831.2	144	GU141811			HYM00000831.2	Arthropoda	Insecta	Hymenoptera	Braconidae	Microgastrinae	Microplitis	Microplitis jft11	Jose Leonardo Fernandez	jftriana@uoguelph.ca		S	A					J. G. Chillcot	05-Aug-1952		Canada	Manitoba	Churchill		Fort Churchill	58.7340012	-93.82		
ASWAY608-08	HYM00000832.2	HYM00000832.2	HYM00000832.2	144	GU141813			HYM00000832.2	Arthropoda	Insecta	Hymenoptera	Braconidae	Microgastrinae	Microplitis	Microplitis jft12	Jose Leonardo Fernandez	jftriana@uoguelph.ca		S	A			NULL		J. G. Chillcot	26-Jun-1952		Canada	Manitoba	Churchill		Farnworth Lake	58.7000008	-94.050003		
ASWAY609-08	HYM00000833.2	HYM00000833.2	HYM00000833.2	144	GU141812			HYM00000833.2	Arthropoda	Insecta	Hymenoptera	Braconidae	Microgastrinae	Microplitis	Microplitis jft12	Jose Leonardo Fernandez	jftriana@uoguelph.ca		S	A			NULL		J. G. Chillcot	07-Aug-1952		Canada	Manitoba	Churchill		Fort Churchill	58.7340012	-93.82		
ASWAY610-08	HYM00000834.2	HYM00000834.2	HYM00000834.2	144	GU141814			HYM00000834.2	Arthropoda	Insecta	Hymenoptera	Braconidae	Microgastrinae	Microplitis	Microplitis varicolor	Jose Leonardo Fernandez	jftriana@uoguelph.ca		S	A			NULL		J. G. Chillcot	05-Aug-1952		Canada	Manitoba	Churchill		Fort Churchill	58.7340012	-93.82		
ASWAY611-08	HYM00000837.2	HYM00000837.2	HYM00000837.2	100	GU141304			HYM00000837.2	Arthropoda	Insecta	Hymenoptera	Braconidae	Microgastrinae	Microplitis	Microplitis varicolor	Jose Leonardo Fernandez	jftriana@uoguelph.ca					<VERBATIM LOCALITY>|Burn Site|58.371|-93.4				17-Aug-2006		Canada	Manitoba	Churchill	26 km SE Churchill, Tw		58.6170006	-93.811996	38	
ASWAY612-08	HYM00000651.2	HYM00000651.2	HYM00000651.2	631	GU141303			HYM00000651.2	Arthropoda	Insecta	Hymenoptera	Braconidae	Microgastrinae	Microplitis	Microplitis varicolor	Jose Leonardo Fernandez	jftriana@uoguelph.ca					<VERBATIM LOCALITY>|Burn Site|58.371|-93.4				20-Aug-2006		Canada	Manitoba	Churchill	26 km SE Churchill, Tw		58.6170006	-93.811996	38	
ASWAY613-08	HYM00000680.2	HYM00000680.2	HYM00000680.2	631	GU141302			HYM00000680.2	Arthropoda	Insecta	Hymenoptera	Braconidae	Microgastrinae	Microplitis	Microplitis varicolor	Jose Leonardo Fernandez	jftriana@uoguelph.ca					<VERBATIM LOCALITY>|Ramsay Creek|58.75				19-Aug-2006		Canada	Manitoba	Churchill	23 km E Churchill, Ram		58.730999	-93.779999	10	
ASWAY614-08	HYM00000888.2	HYM00000888.2	HYM00000888.2	129	GU141705			HYM00000888.2	Arthropoda	Insecta	Hymenoptera	Braconidae	Microgastrinae	Glyptapanteles	Glyptapanteles compressiventris	Jose Leonardo Fernandez	jftriana@uoguelph.ca		S	A					J. G. Chillcot	11-Jun-1952		Canada	Manitoba	Churchill		Fort Churchill	58.7340012	-93.82		
ASWAY615-08	HYM00000889.2	HYM00000889.2	HYM00000889.2	0		0		HYM00000889.2	Arthropoda	Insecta	Hymenoptera	Braconidae	Microgastrinae	Glyptapanteles	Glyptapanteles compressiventris	Jose Leonardo Fernandez	jftriana@uoguelph.ca		S	A					J. G. Chillcot	07-Aug-1952		Canada	Manitoba	Churchill		Fort Churchill				
ASWAY616-08	HYM00000890.2	HYM00000890.2	HYM00000890.2	129	GU141704			HYM00000890.2	Arthropoda	Insecta	Hymenoptera	Braconidae	Microgastrinae	Glyptapanteles	Glyptapanteles compressiventris	Jose Leonardo Fernandez	jftriana@uoguelph.ca		S	A					J. G. Chillcot	07-Aug-1952		Canada	Manitoba	Churchill		Fort Churchill	58.7340012	-93.82		
ASWAY617-08	HYM00000891.2	HYM00000891.2	HYM00000891.2	129	GU141703			HYM00000891.2	Arthropoda	Insecta	Hymenoptera	Braconidae	Microgastrinae	Glyptapanteles	Glyptapanteles compressiventris	Jose Leonardo Fernandez	jftriana@uoguelph.ca		S	A					J. G. Chillcot	17-Jun-1952		Canada	Manitoba	Churchill		Fort Churchill	58.7340012	-93.82		
ASWAY618-08	HYM00000892.2	HYM00000892.2	HYM00000892.2	129	GU141702			HYM00000892.2	Arthropoda	Insecta	Hymenoptera	Braconidae	Microgastrinae	Glyptapanteles	Glyptapanteles compressiventris	Jose Leonardo Fernandez	jftriana@uoguelph.ca		S	A					J. G. Chillcot	11-Jun-1952		Canada	Manitoba	Churchill		Fort Churchill	58.7340012	-93.82		
ASWAY619-08	HYM00000893.2	HYM00000893.2	HYM00000893.2	129	GU141701			HYM00000893.2	Arthropoda	Insecta	Hymenoptera	Braconidae	Microgastrinae	Glyptapanteles	Glyptapanteles compressiventris	Jose Leonardo Fernandez	jftriana@uoguelph.ca		S	A					J. G. Chillcot	11-Jun-1952		Canada	Manitoba	Churchill		Fort Churchill	58.7340012	-93.82		
ASWAY620-08	HYM00000743.2	HYM00000743.2	HYM00000743.2	144	GU141822			HYM00000743.2	Arthropoda	Insecta	Hymenoptera	Braconidae	Microgastrinae	Protapanteles	Protapanteles jft01	Jose Leonardo Fernandez	jftriana@uoguelph.ca		S	A					J. G. Chillcot	29-Jun-1952		Canada	Manitoba	Churchill		Warkworth Creek nr. Cl	58.375	-93.875		
ASWAY621-08	HYM00000896.2	HYM00000896		0		0		HYM00000896.2	Arthropoda	Insecta	Hymenoptera	Braconidae	Microgastrinae	Apanteles	Apanteles sp.	Jose Leonardo Fernandez	jftriana@uoguelph.ca								J. G. Chillcot	29-Jul-1952		Canada	Manitoba	Churchill		Fort Churchill				
ASWAY622-08	HYM00000772.2	HYM00000772.2	HYM00000772.2	320	GU141808			HYM00000772.2	Arthropoda	Insecta	Hymenoptera	Braconidae	Microgastrinae	Microplitis	Microplitis jft01	Jose Leonardo Fernandez	jftriana@uoguelph.ca		S	A					J. G. Chillcot	23-Jun-1952		Canada	Manitoba	Churchill		Fort Churchill	58.7340012	-93.82		
ASWAY623-08	HYM00000773.2	HYM00000773.2	HYM00000773.2	129	GU141807			HYM00000773.2	Arthropoda	Insecta	Hymenoptera	Braconidae	Microgastrinae	Microplitis	Microplitis jft01	Jose Leonardo Fernandez	jftriana@uoguelph.ca		S	A					J. G. Chillcot	23-Jun-1952		Canada	Manitoba	Churchill		Fort Churchill	58.7340012	-93.82		
ASWAY624-08	HYM00000775.2	HYM00000775.2	HYM00000775.2	0		0		HYM00000775.2	Arthropoda	Insecta	Hymenoptera	Braconidae	Microgastrinae	Microplitis	Microplitis jft01	Jose Leonardo Fernandez	jftriana@uoguelph.ca		S	A					J. G. Chillcot	09-Jul-1952		Canada	Manitoba	Churchill		Fort Churchill				
ASWAY625-08	HYM00000776.2	HYM00000776.2	HYM00000776.2	144	GU141806			HYM00000776.2	Arthropoda	Insecta	Hymenoptera	Braconidae	Microgastrinae	Microplitis	Microplitis jft01	Jose Leonardo Fernandez	jftriana@uoguelph.ca		S	A					J. G. Chillcot	23-Jun-1952		Canada	Manitoba	Churchill		Fort Churchill	58.7340012	-93.82		
ASWAY626-08	HYM00000777.2	HYM00000777.2	HYM00000777.2	0		0		HYM00000777.2	Arthropoda	Insecta	Hymenoptera	Braconidae	Microgastrinae	Microplitis	Microplitis jft01	Jose Leonardo Fernandez	jftriana@uoguelph.ca		S	A					J. G. Chillcot	23-Jun-1952		Canada	Manitoba	Churchill		Fort Churchill				
ASWAY627-08	HYM00000778.2	HYM00000778.2	HYM00000778.2	129	GU141805			HYM00000778.2	Arthropoda	Insecta	Hymenoptera	Braconidae	Microgastrinae	Microplitis	Microplitis jft01	Jose Leonardo Fernandez	jftriana@uoguelph.ca		S	A					J. G. Chillcot	23-Jun-1952		Canada	Manitoba	Churchill		Fort Churchill	58.7340012	-93.82		
ASWAY628-08	HYM00000779.2	HYM00000779.2	HYM00000779.2	129	GU141804			HYM00000779.2	Arthropoda	Insecta	Hymenoptera	Braconidae	Microgastrinae	Microplitis	Microplitis jft01	Jose Leonardo Fernandez	jftriana@uoguelph.ca		S	A					J. G. Chillcot	23-Jun-1952		Canada	Manitoba	Churchill		Fort Churchill	58.7340012	-93.82		
ASWAY629-08	HYM00000780.2	HYM00000780.2	HYM00000780.2	0		0		HYM00000780.2	Arthropoda	Insecta	Hymenoptera	Braconidae	Microgastrinae	Microplitis	Microplitis jft01	Jose Leonardo Fernandez	jftriana@uoguelph.ca		S	A					J. G. Chillcot	23-Jun-1952		Canada	Manitoba	Churchill		Fort Churchill				
ASWAY630-08	HYM00000781.2	HYM00000781.2	HYM00000781.2	129	GU141803			HYM00000781.2	Arthropoda	Insecta	Hymenoptera	Braconidae	Microgastrinae	Microplitis	Microplitis jft01	Jose Leonardo Fernandez	jftriana@uoguelph.ca		S	A					J. G. Chillcot	23-Jun-1952		Canada	Manitoba	Churchill		Fort Churchill	58.7340012	-93.82		
ASWAY631-08	HYM00000782.2	HYM00000782.2	HYM00000782.2	129	GU141802			HYM00000782.2	Arthropoda	Insecta	Hymenoptera	Braconidae	Microgastrinae	Microplitis	Microplitis jft01	Jose Leonardo Fernandez	jftriana@uoguelph.ca		S	A					J. G. Chillcot	23-Jun-1952		Canada	Manitoba	Churchill		Fort Churchill	58.7340012	-93.82		
ASWAY632-08	HYM00000783.2	HYM00000783.2	HYM00000783.2	144	GU141801			HYM00000783.2	Arthropoda	Insecta	Hymenoptera	Braconidae	Microgastrinae	Microplitis	Microplitis jft01	Jose Leonardo Fernandez	jftriana@uoguelph.ca		S	A					J. G. Chillcot	23-Jun-1952		Canada	Manitoba	Churchill		Fort Churchill	58.7340012	-93.82		
ASWAY633-08	HYM00000784.2	HYM00000784.2	HYM00000784.2	144	GU141800			HYM00000784.2	Arthropoda	Insecta	Hymenoptera	Braconidae	Microgastrinae	Microplitis	Microplitis jft01	Jose Leonardo Fernandez	jftriana@uoguelph.ca		S	A					J. G. Chillcot	18-Jun-1952		Canada	Manitoba	Churchill		Fort Churchill	58.7340012	-93.82		
ASWAY634-08	HYM00000785.2	HYM00000785.2	HYM00000785.2	0		0		HYM00000785.2	Arthropoda	Insecta	Hymenoptera	Braconidae	Microgastrinae	Microplitis	Microplitis jft01	Jose Leonardo Fernandez	jftriana@uoguelph.ca		S	A					J. G. Chillcot	10-Jun-1952		Canada	Manitoba	Churchill		Fort Churchill				
ASWAY635-08	HYM00000786.2	HYM00000786.2	HYM00000786.2	144	GU141799			HYM00000786.2	Arthropoda	Insecta	Hymenoptera	Braconidae	Microgastrinae	Microplitis	Microplitis jft01	Jose Leonardo Fernandez	jftriana@uoguelph.ca		S	A					J. G. Chillcot	16-Jun-1952		Canada	Manitoba	Churchill		Fort Churchill	58.7340012	-93.82		
ASWAY636-08	HYM00000787.2	HYM00000787.2	HYM00000787.2	144	GU141798			HYM00000787.2	Arthropoda	Insecta	Hymenoptera	Braconidae	Microgastrinae	Microplitis	Microplitis jft01	Jose Leonardo Fernandez	jftriana@uoguelph.ca		S	A					J. G. Chillcot	23-Jun-1952		Canada	Manitoba	Churchill		Fort Churchill	58.7340012	-93.82		
ASWAY637-08	HYM00000788.2	HYM00000788.2	HYM00000788.2	129	GU141797			HYM00000788.2	Arthropoda	Insecta	Hymenoptera	Braconidae	Microgastrinae	Microplitis	Microplitis jft01	Jose Leonardo Fernandez	jftriana@uoguelph.ca		S	A					J. G. Chillcot	23-Jun-1952		Canada	Manitoba	Churchill		Fort Churchill	58.7340012	-93.82		
ASWAY638-08	HYM00000789.2	HYM00000789.2	HYM00000789.2	320	GU141796			HYM00000789.2	Arthropoda	Insecta	Hymenoptera	Braconidae	Microgastrinae	Microplitis	Microplitis jft01	Jose Leonardo Fernandez	jftriana@uoguelph.ca		S	A					J. G. Chillcot	23-Jun-1952		Canada	Manitoba	Churchill		Fort Churchill	58.7340012	-93.82		
ASWAY639-08	HYM00000790.2	HYM00000790.2	HYM00000790.2	129	GU141795			HYM00000790.2	Arthropoda	Insecta	Hymenoptera	Braconidae	Microgastrinae	Microplitis	Microplitis jft01	Jose Leonardo Fernandez	jftriana@uoguelph.ca		S	A					J. G. Chillcot	29-Jun-1952		Canada	Manitoba	Churchill		Fort Churchill	58.7340012	-93.82		
ASWAY640-08	HYM00000791.2	HYM00000791.2	HYM00000791.2	129	GU141794			HYM00000791.2	Arthropoda	Insecta	Hymenoptera	Braconidae	Microgastrinae	Microplitis	Microplitis jft01	Jose Leonardo Fernandez	jftriana@uoguelph.ca		S	A					J. G. Chillcot	23-Jun-1952		Canada	Manitoba	Churchill		Fort Churchill	58.7340012	-93.82		
ASWAY641-08	HYM00000650.2	HYM00000650.2	HYM00000650.2	631	GU141208			HYM00000650.2	Arthropoda	Insecta	Hymenoptera	Braconidae	Microgastrinae	Glyptapanteles	Glyptapanteles jft05	Jose Leonardo Fernandez	jftriana@uoguelph.ca					<VERBATIM LOCALITY>|Burn Site|58.371|-93.4				20-Aug-2006		Canada	Manitoba	Churchill	26 km SE Churchill, Tw		58.6170006	-93.811996	38	
ASWAY642-08	HYM00000655.2	HYM00000655.2	HYM00000655.2	631	GU141207			HYM00000655.2	Arthropoda	Insecta	Hymenoptera	Braconidae	Microgastrinae	Glyptapanteles	Glyptapanteles jft05	Jose Leonardo Fernandez	jftriana@uoguelph.ca					<VERBATIM LOCALITY>|Burn Site|58.371|-93.4				20-Aug-2006		Canada	Manitoba	Churchill	26 km SE Churchill, Tw		58.6170006	-93.811996	38	
ASWAY643-08	HYM00000656.2	HYM00000656.2	HYM00000656.2	631	GU141217			HYM00000656.2	Arthropoda	Insecta	Hymenoptera	Braconidae	Microgastrinae	Glyptapanteles	Glyptapanteles jft05	Jose Leonardo Fernandez	jftriana@uoguelph.ca					<VERBATIM LOCALITY>|Burn Site|58.371|-93.4				20-Aug-2006		Canada	Manitoba	Churchill	26 km SE Churchill, Tw		58.6170006	-93.811996	38	
ASWAY644-08	HYM00000657.2	HYM00000657.2	HYM00000657.2	631	GU141194			HYM00000657.2	Arthropoda	Insecta	Hymenoptera	Braconidae	Microgastrinae	Glyptapanteles	Glyptapanteles jft01	Jose Leonardo Fernandez	jftriana@uoguelph.ca					<VERBATIM LOCALITY>|Burn Site|58.371|-93.4				20-Aug-2006		Canada	Manitoba	Churchill	26 km SE Churchill, Tw		58.6170006	-93.811996	38	
ASWAY645-08	HYM00000659.2	HYM00000659.2	HYM00000659.2	631	GU141216			HYM00000659.2	Arthropoda	Insecta	Hymenoptera	Braconidae	Microgastrinae	Glyptapanteles	Glyptapanteles jft05	Jose Leonardo Fernandez	jftriana@uoguelph.ca					<VERBATIM LOCALITY>|Burn Site, Polar trees				14-Aug-2006		Canada	Manitoba	Churchill	26 km SE Churchill, Tw	Poplar trees	58.618	-93.829002		
ASWAY646-08	HYM00000660.2	HYM00000660.2	HYM00000660.2	631	GU141215			HYM00000660.2	Arthropoda	Insecta	Hymenoptera	Braconidae	Microgastrinae	Glyptapanteles	Glyptapanteles jft05	Jose Leonardo Fernandez	jftriana@uoguelph.ca					<VERBATIM LOCALITY>|Burn Site|58.371|-93.4				20-Aug-2006		Canada	Manitoba	Churchill	26 km SE Churchill, Tw		58.6170006	-93.811996	38	
ASWAY647-08	HYM00000661.2	HYM00000661.2	HYM00000661.2	631	GU141214			HYM00000661.2	Arthropoda	Insecta	Hymenoptera	Braconidae	Microgastrinae	Glyptapanteles	Glyptapanteles jft05	Jose Leonardo Fernandez	jftriana@uoguelph.ca					<VERBATIM LOCALITY>|Burn Site|58.371|-93.4				20-Aug-2006		Canada	Manitoba	Churchill	26 km SE Churchill, Tw		58.6170006	-93.811996	38	
ASWAY648-08	HYM00000662.2	HYM00000662.2	HYM00000662.2	631	GU141213			HYM00000662.2	Arthropoda	Insecta	Hymenoptera	Braconidae	Microgastrinae	Glyptapanteles	Glyptapanteles jft05	Jose Leonardo Fernandez	jftriana@uoguelph.ca					<VERBATIM LOCALITY>|Burn Site, Polar trees				14-Aug-2006		Canada	Manitoba	Churchill	26 km SE Churchill, Tw	Poplar trees	58.618	-93.829002		
ASWAY649-08	HYM00000670.2	HYM00000670.2	HYM00000670.2	631	GU141222			HYM00000670.2	Arthropoda	Insecta	Hymenoptera	Braconidae	Microgastrinae	Glyptapanteles	Glyptapanteles jft09	Jose Leonardo Fernandez	jftriana@uoguelph.ca					<VERBATIM LOCALITY>|Pump house, 15 Km S				08-Sep-2006		Canada	Manitoba	Churchill	16 km SW Churchill, Ch		58.6259995	-94.228996		
ASWAY650-08	HYM00000671.2	HYM00000671.2	HYM00000671.2	631	GU141212			HYM00000671.2	Arthropoda	Insecta	Hymenoptera	Braconidae	Microgastrinae	Glyptapanteles	Glyptapanteles jft05	Jose Leonardo Fernandez	jftriana@uoguelph.ca					<VERBATIM LOCALITY>|Burn Site|58.371|-93.4				20-Aug-2006		Canada	Manitoba	Churchill	26 km SE Churchill, Tw		58.6170006	-93.811996	38	
ASWAY651-08	HYM00000673.2	HYM00000673.2	HYM00000673.2	616	GU141211			HYM00000673.2	Arthropoda	Insecta	Hymenoptera	Braconidae	Microgastrinae	Glyptapanteles	Glyptapanteles jft05	Jose Leonardo Fernandez	jftriana@uoguelph.ca					<VERBATIM LOCALITY>|Burn Site|58.371|-93.4				20-Aug-2006		Canada	Manitoba	Churchill	26 km SE Churchill, Tw		58.6170006	-93.811996	38	
ASWAY652-08	HYM00000676.2	HYM00000676.2	HYM00000676.2	631	GU141210			HYM00000676.2	Arthropoda	Insecta	Hymenoptera	Braconidae	Microgastrinae	Glyptapanteles	Glyptapanteles jft05	Jose Leonardo Fernandez	jftriana@uoguelph.ca					<VERBATIM LOCALITY>|Ramsay Creek|58.75				20-Aug-2006		Canada	Manitoba	Churchill	23 km E Churchill, Ram		58.730999	-93.779999	10	
ASWAY653-08	HYM00000677.2	HYM00000677.2	HYM00000677.2	631	GU141209			HYM00000677.2	Arthropoda	Insecta	Hymenoptera	Braconidae	Microgastrinae	Glyptapanteles	Glyptapanteles jft05	Jose Leonardo Fernandez	jftriana@uoguelph.ca					<VERBATIM LOCALITY>|Ramsay Creek|58.75				19-Aug-2006		Canada	Manitoba	Churchill	23 km E Churchill, Ram		58.730999	-93.779999	10	
ASWAY654-08	HYM00000822.2	HYM00000822.2	HYM00000822.2	129	GU141810			HYM00000822.2	Arthropoda	Insecta	Hymenoptera	Braconidae	Microgastrinae	Microplitis	Microplitis jft10	Jose Leonardo Fernandez	jftriana@uoguelph.ca		S	A			NULL		J. G. Chillcot	21-Jun-1952		Canada	Manitoba	Churchill		Fort Churchill	58.7340012	-93.82		
ASWAY655-08	HYM00000695.2	HYM00000695.2	HYM00000695.2	144	GU141744			HYM00000695.2	Arthropoda	Insecta	Hymenoptera	Braconidae	Microgastrinae	Microgaster	Microgaster deductor	Jose Leonardo Fernandez	jftriana@uoguelph.ca		S	A					J. G. Chillcot	12-Jul-1952		Canada	Manitoba	Churchill		Fort Churchill	58.7340012	-93.82		
ASWAY656-08	HYM00000708.2	HYM00000708.2	HYM00000708.2	144	GU141743			HYM00000708.2	Arthropoda	Insecta	Hymenoptera	Braconidae	Microgastrinae	Microgaster	Microgaster deductor	Jose Leonardo Fernandez	jftriana@uoguelph.ca		S	A					J. G. Chillcot	29-Jun-1952		Canada	Manitoba	Churchill		Warkworth Creek nr. Cl	58.375	-93.875		
ASWAY657-08	HYM00000696.2	HYM00000696.2	HYM00000696.2	144	GU141742			HYM00000696.2	Arthropoda	Insecta	Hymenoptera	Braconidae	Microgastrinae	Microgaster	Microgaster deductor	Jose Leonardo Fernandez	jftriana@uoguelph.ca		S	A					J. G. Chillcot	29-Jun-1952		Canada	Manitoba	Churchill		Warkworth Creek nr. Cl	58.375	-93.875		
ASWAY658-08	HYM00000702.2	HYM00000702.2	HYM00000702.2	144	GU141741			HYM00000702.2	Arthropoda	Insecta	Hymenoptera	Braconidae	Microgastrinae	Microgaster	Microgaster deductor	Jose Leonardo Fernandez	jftriana@uoguelph.ca		S	A					J. G. Chillcot	12-Jul-1952		Canada	Manitoba	Churchill		Fort Churchill	58.7340012	-93.82		
ASWAY659-08	HYM00000700.2	HYM00000700.2	HYM00000700.2	0		0		HYM00000700.2	Arthropoda	Insecta	Hymenoptera	Braconidae	Microgastrinae	Microgaster	Microgaster deductor	Jose Leonardo Fernandez	jftriana@uoguelph.ca		S	A					J. G. Chillcot	12-Jul-1952		Canada	Manitoba	Churchill		Fort Churchill				
ASWAY660-08	HYM00000704.2	HYM00000704.2	HYM00000704.2	144	GU141740			HYM00000704.2	Arthropoda	Insecta	Hymenoptera	Braconidae	Microgastrinae	Microgaster	Microgaster deductor	Jose Leonardo Fernandez	jftriana@uoguelph.ca		S	A					J. G. Chillcot	12-Jul-1952		Canada	Manitoba	Churchill		Fort Churchill	58.7340012	-93.82		
ASWAY661-08	HYM00000703.2	HYM00000703.2	HYM00000703.2	144	GU141739			HYM00000703.2	Arthropoda	Insecta	Hymenoptera	Braconidae	Microgastrinae	Microgaster	Microgaster deductor	Jose Leonardo Fernandez	jftriana@uoguelph.ca		S	A					J. G. Chillcot	12-Jul-1952		Canada	Manitoba	Churchill		Fort Churchill	58.7340012	-93.82		
ASWAY662-08	HYM00000712.2	HYM00000712.2	HYM00000712.2	144	GU141738			HYM00000712.2	Arthropoda	Insecta	Hymenoptera	Braconidae	Microgastrinae	Microgaster	Microgaster deductor	Jose Leonardo Fernandez	jftriana@uoguelph.ca		S	A					J. G. Chillcot	12-Jul-1952		Canada	Manitoba	Churchill		Fort Churchill	58.7340012	-93.82		
ASWAY663-08	HYM00000707.2	HYM00000707.2	HYM00000707.2	144	GU141737			HYM00000707.2	Arthropoda	Insecta	Hymenoptera	Braconidae	Microgastrinae	Microgaster	Microgaster deductor	Jose Leonardo Fernandez	jftriana@uoguelph.ca		S	A					J. G. Chillcot	07-Jul-1952		Canada	Manitoba	Churchill		Warkworth Creek nr. Cl	58.375	-93.875		
ASWAY664-08	HYM00000699.2	HYM00000699.2	HYM00000699.2	320	GU141736			HYM00000699.2	Arthropoda	Insecta	Hymenoptera	Braconidae	Microgastrinae	Microgaster	Microgaster deductor	Jose Leonardo Fernandez	jftriana@uoguelph.ca		S	A					J. G. Chillcot	23-Jul-1952		Canada	Manitoba	Churchill		Fort Churchill	58.7340012	-93.82		
ASWAY665-08	HYM00000711.2	HYM00000711.2	HYM00000711.2	0		0		HYM00000711.2	Arthropoda	Insecta	Hymenoptera	Braconidae	Microgastrinae	Microgaster	Microgaster deductor	Jose Leonardo Fernandez	jftriana@uoguelph.ca		S	A					J. G. Chillcot	05-Aug-1952		Canada	Manitoba	Churchill		Fort Churchill				
ASWAY666-08	HYM00000706.2	HYM00000706.2	HYM00000706.2	143	GU141735			HYM00000706.2	Arthropoda	Insecta	Hymenoptera	Braconidae	Microgastrinae	Microgaster	Microgaster deductor	Jose Leonardo Fernandez	jftriana@uoguelph.ca		S	A					J. G. Chillcot	12-Jul-1952		Canada	Manitoba	Churchill		Fort Churchill	58.7340012	-93.82		
ASWAY667-08	HYM00000697.2	HYM00000697.2	HYM00000697.2	144	GU141734			HYM00000697.2	Arthropoda	Insecta	Hymenoptera	Braconidae	Microgastrinae	Microgaster	Microgaster deductor	Jose Leonardo Fernandez	jftriana@uoguelph.ca		S	A					J. G. Chillcot	23-Jul-1952		Canada	Manitoba	Churchill		Fort Churchill	58.7340012	-93.82		
ASWAY668-08	HYM00000710.2	HYM00000710.2	HYM00000710.2	144	GU141733			HYM00000710.2	Arthropoda	Insecta	Hymenoptera	Braconidae	Microgastrinae	Microgaster	Microgaster deductor	Jose Leonardo Fernandez	jftriana@uoguelph.ca		S	A					J. G. Chillcot	12-Jul-1952		Canada	Manitoba	Churchill		Fort Churchill	58.7340012	-93.82		
ASWAY669-08	HYM00000709.2	HYM00000709.2	HYM00000709.2	144	GU141732			HYM00000709.2	Arthropoda	Insecta	Hymenoptera	Braconidae	Microgastrinae	Microgaster	Microgaster deductor	Jose Leonardo Fernandez	jftriana@uoguelph.ca		S	A					J. G. Chillcot	12-Jul-1952		Canada	Manitoba	Churchill		Fort Churchill	58.7340012	-93.82		
ASWAY670-08	HYM00000701.2	HYM00000701.2	HYM00000701.2	129	GU141731			HYM00000701.2	Arthropoda	Insecta	Hymenoptera	Braconidae	Microgastrinae	Microgaster	Microgaster deductor	Jose Leonardo Fernandez	jftriana@uoguelph.ca		S	A					J. G. Chillcot	12-Jul-1952		Canada	Manitoba	Churchill		Fort Churchill	58.7340012	-93.82		
ASWAY671-08	HYM00000714.2	HYM00000714.2	HYM00000714.2	144	GU141763			HYM00000714.2	Arthropoda	Insecta	Hymenoptera	Braconidae	Microgastrinae	Microgaster	Microgaster deductor	Jose Leonardo Fernandez	jftriana@uoguelph.ca		S	A					J. G. Chillcot	29-Jul-1952		Canada	Manitoba	Churchill		Fort Churchill	58.7340012	-93.82		
ASWAY672-08	HYM00000713.2	HYM00000713.2	HYM00000713.2	129	GU141762			HYM00000713.2	Arthropoda	Insecta	Hymenoptera	Braconidae	Microgastrinae	Microgaster	Microgaster deductor	Jose Leonardo Fernandez	jftriana@uoguelph.ca		S	A					J. G. Chillcot	23-Jul-1952		Canada	Manitoba	Churchill		Fort Churchill	58.7340012	-93.82		
ASWAY673-08	HYM00000730.2	HYM00000730.2	HYM00000730.2	144	GU141761			HYM00000730.2	Arthropoda	Insecta	Hymenoptera	Braconidae	Microgastrinae	Microgaster	Microgaster deductor	Jose Leonardo Fernandez	jftriana@uoguelph.ca		S	A					J. G. Chillcot	03-Aug-1952		Canada	Manitoba	Churchill		Fort Churchill	58.7340012	-93.82		
ASWAY674-08	HYM00000919.2	HYM00000919.2	HYM00000919.2	144	GU141760			HYM00000919.2	Arthropoda	Insecta	Hymenoptera	Braconidae	Microgastrinae	Microgaster	Microgaster deductor	Jose Leonardo Fernandez	jftriana@uoguelph.ca		S	A					J. G. Chillcot	28-Jul-1952		Canada	Manitoba	Churchill		Fort Churchill	58.7340012	-93.82		
ASWAY675-08	HYM00000717.2	HYM00000717.2	HYM00000717.2	0		0		HYM00000717.2	Arthropoda	Insecta	Hymenoptera	Braconidae	Microgastrinae	Microgaster	Microgaster deductor	Jose Leonardo Fernandez	jftriana@uoguelph.ca		S	A					J. G. Chillcot	03-Aug-1952		Canada	Manitoba	Churchill		Warkworth Creek nr. Cl				
ASWAY676-08	HYM00000716.2	HYM00000716.2	HYM00000716.2	144	GU141759			HYM00000716.2	Arthropoda	Insecta	Hymenoptera	Braconidae	Microgastrinae	Microgaster	Microgaster deductor	Jose Leonardo Fernandez	jftriana@uoguelph.ca		S	A					J. G. Chillcot	03-Aug-1952		Canada	Manitoba	Churchill		Fort Churchill	58.7340012	-93.82		
ASWAY677-08	HYM00000724.2	HYM00000724.2	HYM00000724.2	129	GU141758			HYM00000724.2	Arthropoda	Insecta	Hymenoptera	Braconidae	Microgastrinae	Microgaster	Microgaster deductor	Jose Leonardo Fernandez	jftriana@uoguelph.ca		S	A					J. G. Chillcot	12-Jul-1952		Canada	Manitoba	Churchill		Fort Churchill	58.7340012	-93.82		
ASWAY678-08	HYM00000723.2	HYM00000723.2	HYM00000723.2	144	GU141757			HYM00000723.2	Arthropoda	Insecta	Hymenoptera	Braconidae	Microgastrinae	Microgaster	Microgaster deductor	Jose Leonardo Fernandez	jftriana@uoguelph.ca		S	A					J. G. Chillcot	12-Jul-1952		Canada	Manitoba	Churchill		Fort Churchill	58.7340012	-93.82		
ASWAY679-08	HYM00000722.2	HYM00000722.2	HYM00000722.2	144	GU141756			HYM00000722.2	Arthropoda	Insecta	Hymenoptera	Braconidae	Microgastrinae	Microgaster	Microgaster deductor	Jose Leonardo Fernandez	jftriana@uoguelph.ca		S	A					J. G. Chillcot	28-Jul-1952		Canada	Manitoba	Churchill		Fort Churchill	58.7340012	-93.82		
ASWAY680-08	HYM00000721.2	HYM00000721.2	HYM00000721.2	144	GU141755			HYM00000721.2	Arthropoda	Insecta	Hymenoptera	Braconidae	Microgastrinae	Microgaster	Microgaster deductor	Jose Leonardo Fernandez	jftriana@uoguelph.ca		S	A					J. G. Chillcot	03-Aug-1952		Canada	Manitoba	Churchill		Fort Churchill	58.7340012	-93.82		
ASWAY681-08	HYM00000729.2	HYM00000729.2	HYM00000729.2	144	GU141754			HYM00000729.2	Arthropoda	Insecta	Hymenoptera	Braconidae	Microgastrinae	Microgaster	Microgaster deductor	Jose Leonardo Fernandez	jftriana@uoguelph.ca		S	A					J. G. Chillcot	23-Jul-1952		Canada	Manitoba	Churchill		Fort Churchill	58.7340012	-93.82		
ASWAY682-08	HYM00000715.2	HYM00000715.2	HYM00000715.2	144	GU141753			HYM00000715.2	Arthropoda	Insecta	Hymenoptera	Braconidae	Microgastrinae	Microgaster	Microgaster deductor	Jose Leonardo Fernandez	jftriana@uoguelph.ca		S	A					J. G. Chillcot	12-Jul-1952		Canada	Manitoba	Churchill		Fort Churchill	58.7340012	-93.82		
ASWAY683-08	HYM00000698.2	HYM00000698.2	HYM00000698.2	144	GU141752			HYM00000698.2	Arthropoda	Insecta	Hymenoptera	Braconidae	Microgastrinae	Microgaster	Microgaster deductor	Jose Leonardo Fernandez	jftriana@uoguelph.ca		S	A					J. G. Chillcot	18-Jul-1952		Canada	Manitoba	Churchill		Fort Churchill	58.7340012	-93.82		
ASWAY684-08	HYM00000725.2	HYM00000725.2	HYM00000725.2	144	GU141751			HYM00000725.2	Arthropoda	Insecta	Hymenoptera	Braconidae	Microgastrinae	Microgaster	Microgaster deductor	Jose Leonardo Fernandez	jftriana@uoguelph.ca		S	A					J. G. Chillcot	03-Aug-1952		Canada	Manitoba	Churchill		Warkworth Creek nr. Cl	58.375	-93.875		
ASWAY685-08	HYM00000718.2	HYM00000718.2	HYM00000718.2	129	GU141750			HYM00000718.2	Arthropoda	Insecta	Hymenoptera	Braconidae	Microgastrinae	Microgaster	Microgaster deductor	Jose Leonardo Fernandez	jftriana@uoguelph.ca		S	A					J. G. Chillcot	29-Jul-1952		Canada	Manitoba	Churchill		Fort Churchill	58.7340012	-93.82		
ASWAY686-08	HYM00000726.2	HYM00000726.2	HYM00000726.2	129	GU141749			HYM00000726.2	Arthropoda	Insecta	Hymenoptera	Braconidae	Microgastrinae	Microgaster	Microgaster deductor	Jose Leonardo Fernandez	jftriana@uoguelph.ca		S	A					J. G. Chillcot	28-Jul-1952		Canada	Manitoba	Churchill		Fort Churchill	58.7340012	-93.82		
ASWAY687-08	HYM00000719.2	HYM00000719.2	HYM00000719.2	144	GU141748			HYM00000719.2	Arthropoda	Insecta	Hymenoptera	Braconidae	Microgastrinae	Microgaster	Microgaster deductor	Jose Leonardo Fernandez	jftriana@uoguelph.ca		S	A					J. G. Chillcot	12-Jul-1952		Canada	Manitoba	Churchill		Fort Churchill	58.7340012	-93.82		
ASWAY688-08	HYM00000727.2	HYM00000727.2	HYM00000727.2	0		0		HYM00000727.2	Arthropoda	Insecta	Hymenoptera	Braconidae	Microgastrinae	Microgaster	Microgaster deductor	Jose Leonardo Fernandez	jftriana@uoguelph.ca		S	A					J. G. Chillcot	12-Jul-1952		Canada	Manitoba	Churchill		Fort Churchill				
ASWAY689-08	HYM00000720.2	HYM00000720.2	HYM00000720.2	144	GU141747			HYM00000720.2	Arthropoda	Insecta	Hymenoptera	Braconidae	Microgastrinae	Microgaster	Microgaster deductor	Jose Leonardo Fernandez	jftriana@uoguelph.ca		S	A					J. G. Chillcot	03-Aug-1952		Canada	Manitoba	Churchill		Fort Churchill	58.7340012	-93.82		
ASWAY690-08	HYM00000728.2	HYM00000728.2	HYM00000728.2	144	GU141746			HYM00000728.2	Arthropoda	Insecta	Hymenoptera	Braconidae	Microgastrinae	Microgaster	Microgaster deductor	Jose Leonardo Fernandez	jftriana@uoguelph.ca		S	A					J. G. Chillcot	23-Jul-1952		Canada	Manitoba	Churchill		Fort Churchill	58.7340012	-93.82		
ASWAY691-08	HYM00000918.2	HYM00000918.2	HYM00000918.2	144	GU141745			HYM00000918.2	Arthropoda	Insecta	Hymenoptera	Braconidae	Microgastrinae	Microgaster	Microgaster deductor	Jose Leonardo Fernandez	jftriana@uoguelph.ca		S	A					J. G. Chillcot	28-Jul-1952		Canada	Manitoba	Churchill		Fort Churchill	58.7340012	-93.82		
ASWAY692-08	HYM00000689.2	HYM00000689.2	HYM00000689.2	631	GU141165			HYM00000689.2	Arthropoda	Insecta	Hymenoptera	Braconidae	Microgastrinae	Dolichogenidea	Dolichogenidea jft03	Jose Leonardo Fernandez	jftriana@uoguelph.ca					<VERBATIM LOCALITY>|Burn Site|58.371|-93.4				15-Aug-2006		Canada	Manitoba	Churchill	26 km SE Churchill, Tw		58.6170006	-93.811996	38	
ASWAY693-08	HYM00000690.2	HYM00000690.2	HYM00000690.2	631	GU141164			HYM00000690.2	Arthropoda	Insecta	Hymenoptera	Braconidae	Microgastrinae	Dolichogenidea	Dolichogenidea jft03	Jose Leonardo Fernandez	jftriana@uoguelph.ca					<VERBATIM LOCALITY>|Burn Site|58.371|-93.4				15-Aug-2006		Canada	Manitoba	Churchill	26 km SE Churchill, Tw		58.6170006	-93.811996	38	
ASWAY694-08	HYM00000692.2	HYM00000692.2	HYM00000692.2	631	GU141163			HYM00000692.2	Arthropoda	Insecta	Hymenoptera	Braconidae	Microgastrinae	Dolichogenidea	Dolichogenidea jft03	Jose Leonardo Fernandez	jftriana@uoguelph.ca					<VERBATIM LOCALITY>|Burn Site|58.371|-93.4				17-Aug-2006		Canada	Manitoba	Churchill	26 km SE Churchill, Tw		58.6170006	-93.811996	38	
ASWAY695-08	HYM00001926.2	HYM00001926.2	HYM00001926.2	144	GU141816			HYM00001926.2	Arthropoda	Insecta	Hymenoptera	Braconidae	Microgastrinae	Pholetesor	Pholetesor ornigis	Jose Leonardo Fernandez	jftriana@uoguelph.ca		S	A					A. Renaud	07-Jul-2007		Canada	Manitoba	Churchill		Beach Bay, 4 km S Chu	58.7459984	-94.135002		
ASWAY696-08	HYM00000908.2	HYM00000908.2	HYM00000908.2	631	GU141385			HYM00000908.2	Arthropoda	Insecta	Hymenoptera	Braconidae	Microgastrinae	Sathon	Sathon jft01	Jose Leonardo Fernandez	jftriana@uoguelph.ca					<VERBATIM LOCALITY>|Burn Site|58.371|-93.4				17-Aug-2006		Canada	Manitoba	Churchill	26 km SE Churchill, Tw		58.6170006	-93.811996	38	
CNCHX052-09	CAM0922	CAM0922	CAM0922	657				CAM0922	Arthropoda	Insecta	Hymenoptera	Braconidae	Microgastrinae	Microgaster	Microgaster jft10	Jose Leonardo Fernandez	jftriana@uoguelph.ca		S	A			NULL		H. Goulet			Canada	Ontario	Ottawa		Ottwa city garden	45.355999	-75.707001		
CNCHX053-09	CAM0923	CAM0923	CAM0923	560				CAM0923	Arthropoda	Insecta	Hymenoptera	Braconidae	Microgastrinae	Microgaster	Microgaster jft10	Jose Leonardo Fernandez	jftriana@uoguelph.ca		S	A			NULL		H. Goulet			Canada	Ontario	Ottawa		Ottwa city garden	45.355999	-75.707001		
CNCHX055-09	CAM0925	CAM0925	CAM0925	538				CAM0925	Arthropoda	Insecta	Hymenoptera	Braconidae	Microgastrinae	Microgaster	Microgaster jft10	Jose Leonardo Fernandez	jftriana@uoguelph.ca		S	A			NULL		H. Goulet			Canada	Ontario	Ottawa		Ottwa city garden	45.355999	-75.707001		
CNCHX056-09	CAM0926	CAM0926	CAM0926	657				CAM0926	Arthropoda	Insecta	Hymenoptera	Braconidae	Microgastrinae	Microgaster	Microgaster jft02	Jose Leonardo Fernandez	jftriana@uoguelph.ca		S	A			NULL		H. Goulet			Canada	Ontario	Ottawa		Ottwa city garden	45.355999	-75.707001		
CNCHX057-09	CAM0927	CAM0927	CAM0927	657				CAM0927	Arthropoda	Insecta	Hymenoptera	Braconidae	Microgastrinae	Microgaster	Microgaster jft10	Jose Leonardo Fernandez	jftriana@uoguelph.ca		S	A			NULL		H. Goulet			Canada	Ontario	Ottawa		Ottwa city garden	45.355999	-75.707001		
CNCHX058-09	CAM0928	CAM0928	CAM0928	657				CAM0928	Arthropoda	Insecta	Hymenoptera	Braconidae	Microgastrinae	Microgaster	Microgaster jft10	Jose Leonardo Fernandez	jftriana@uoguelph.ca		S	A			NULL		H. Goulet			Canada	Ontario	Ottawa		Ottwa city garden	45.355999	-75.707001		
CNCHX126-09	CAM0996	CAM0996	CAM0996	657				CAM0996	Arthropoda	Insecta	Hymenoptera	Braconidae	Microgastrinae	Cotesia	Cotesia xylina	Jose Leonardo Fernandez	jftriana@uoguelph.ca		S	A					Goulet, Boudreault	03-Jun-2008		Canada	Ontario	Evansville		7 km W of Evansville, a	45.8089981	-82.646004		
CNCHX128-09	CAM0998	CAM0998	CAM0998	657				CAM0998	Arthropoda	Insecta	Hymenoptera	Braconidae	Microgastrinae	Cotesia	Cotesia xylina	Jose Leonardo Fernandez	jftriana@uoguelph.ca		S	A					Goulet, Boudreault	03-Jun-2008		Canada	Ontario	Evansville		7 km W of Evansville, a	45.8089981	-82.646004		
CNCHX130-09	CAM1000	CAM1000	CAM1000	657				CAM1000	Arthropoda	Insecta	Hymenoptera	Braconidae	Microgastrinae	Apanteles	Apanteles petrovae	Jose Leonardo Fernandez	jftriana@uoguelph.ca								H. Goulet			Canada	Ontario	Ottawa		Ottwa city garden	45.3560982	-75.706902		
CNCHX132-09	CAM1002	CAM1002	CAM1002	657				CAM1002	Arthropoda	Insecta	Hymenoptera	Braconidae	Microgastrinae	Apanteles	Apanteles petrovae	Jose Leonardo Fernandez	jftriana@uoguelph.ca								H. Goulet			Canada	Ontario	Ottawa		Ottwa city garden	45.3560982	-75.706902		
CNCHX136-09	CAM1006	CAM1006	CAM1006	657				CAM1006	Arthropoda	Insecta	Hymenoptera	Braconidae	Microgastrinae	Apanteles	Apanteles petrovae	Jose Leonardo Fernandez	jftriana@uoguelph.ca								H. Goulet			Canada	Ontario	Ottawa		Ottwa city garden	45.3560982	-75.706902		
CNCHX138-09	CAM1008	CAM1008	CAM1008	657				CAM1008	Arthropoda	Insecta	Hymenoptera	Braconidae	Microgastrinae	Apanteles	Apanteles petrovae	Jose Leonardo Fernandez	jftriana@uoguelph.ca								H. Goulet			Canada	Ontario	Ottawa		Ottwa city garden	45.3560982	-75.706902		
CNCHX139-09	CAM1009	CAM1009	CAM1009	657				CAM1009	Arthropoda	Insecta	Hymenoptera	Braconidae	Microgastrinae	Apanteles	Apanteles petrovae	Jose Leonardo Fernandez	jftriana@uoguelph.ca								H. Goulet			Canada	Ontario	Ottawa		Ottwa city garden	45.3560982	-75.706902		
CNCHX163-09	CAM1033	CAM1033	CAM1033	657				CAM1033	Arthropoda	Insecta	Hymenoptera	Braconidae	Microgastrinae	Cotesia	Cotesia jft02	Jose Leonardo Fernandez	jftriana@uoguelph.ca		S	A					H. Goulet			Canada	Ontario	Ottawa		Ottwa city garden	45.355999	-75.707001		
CNCHX166-09	CAM1036	CAM1036	CAM1036	657				CAM1036	Arthropoda	Insecta	Hymenoptera	Braconidae	Microgastrinae	Cotesia	Cotesia jft02	Jose Leonardo Fernandez	jftriana@uoguelph.ca		S	A					H. Goulet			Canada	Ontario	Ottawa		Ottwa city garden	45.355999	-75.707001		
CNCHX168-09	CAM1038	CAM1038	CAM1038	657				CAM1038	Arthropoda	Insecta	Hymenoptera	Braconidae	Microgastrinae	Cotesia	Cotesia jft04	Jose Leonardo Fernandez	jftriana@uoguelph.ca		S	A					H. Goulet			Canada	Ontario	Ottawa		Ottwa city garden	45.355999	-75.707001		
CNCHX171-09	HYM 00000997	HYM 00000997	HYM 00000997	657				HYM 00000997	Arthropoda	Insecta	Hymenoptera	Braconidae	Microgastrinae	Cotesia	Cotesia jft07	Jose Leonardo Fernandez	jftriana@uoguelph.ca		S	A					H. Goulet			Canada	Ontario	Ottawa		Ottwa city garden	45.355999	-75.707001		
CNCHX172-09	HYM 00000998	HYM 00000998	HYM 00000998	657				HYM 00000998	Arthropoda	Insecta	Hymenoptera	Braconidae	Microgastrinae	Protapanteles	Protapanteles jft02	Jose Leonardo Fernandez	jftriana@uoguelph.ca								H. Goulet			Canada	Ontario	Ottawa		Ottwa city garden	45.3560982	-75.706902		
CNCHX173-09	HYM 00000999	HYM 00000999	HYM 00000999	657				HYM 00000999	Arthropoda	Insecta	Hymenoptera	Braconidae	Microgastrinae	Cotesia	Cotesia jft02	Jose Leonardo Fernandez	jftriana@uoguelph.ca		S	A					H. Goulet			Canada	Ontario	Ottawa		Ottwa city garden	45.355999	-75.707001		
CNCHX193-09	WMIC 0193	WMIC 0193	WMIC 0193	657				WMIC 0193	Arthropoda	Insecta	Hymenoptera	Braconidae	Microgastrinae	Microplitis	Microplitis varicolor	Jose Leonardo Fernandez	jftriana@uoguelph.ca						NULL		H. Goulet			Canada	Ontario	Ottawa		Ottwa city garden	45.3560982	-75.706902		
CNCHX194-09	WMIC 0194	WMIC 0194	WMIC 0194	657				WMIC 0194	Arthropoda	Insecta	Hymenoptera	Braconidae	Microgastrinae	Microplitis	Microplitis varicolor	Jose Leonardo Fernandez	jftriana@uoguelph.ca						NULL		H. Goulet			Canada	Ontario	Ottawa		Ottwa city garden	45.3560982	-75.706902		
CNCHX195-09	WMIC 0195	WMIC 0195	WMIC 0195	657				WMIC 0195	Arthropoda	Insecta	Hymenoptera	Braconidae	Microgastrinae	Microplitis	Microplitis varicolor	Jose Leonardo Fernandez	jftriana@uoguelph.ca						NULL		H. Goulet			Canada	Ontario	Ottawa		Ottwa city garden	45.3560982	-75.706902		
CNCHX196-09	WMIC 0196	WMIC 0196	WMIC 0196	650				WMIC 0196	Arthropoda	Insecta	Hymenoptera	Braconidae	Microgastrinae	Microplitis	Microplitis jft09	Jose Leonardo Fernandez	jftriana@uoguelph.ca						NULL		H. Goulet			Canada	Ontario	Ottawa		Ottwa city garden	45.3560982	-75.706902		
CNCHX197-09	WMIC 0197	WMIC 0197	WMIC 0197	657				WMIC 0197	Arthropoda	Insecta	Hymenoptera	Braconidae	Microgastrinae	Microplitis	Microplitis varicolor	Jose Leonardo Fernandez	jftriana@uoguelph.ca						NULL		H. Goulet			Canada	Ontario	Ottawa		Ottwa city garden	45.3560982	-75.706902		
CNCHX201-09	WMIC 0201	WMIC 0201	WMIC 0201	657				WMIC 0201	Arthropoda	Insecta	Hymenoptera	Braconidae	Microgastrinae	Microplitis	Microplitis varicolor	Jose Leonardo Fernandez	jftriana@uoguelph.ca						NULL		H. Goulet			Canada	Ontario	Ottawa		Ottwa city garden	45.3560982	-75.706902		
CNCHX203-09	WMIC 0203	WMIC 0203	WMIC 0203	657				WMIC 0203	Arthropoda	Insecta	Hymenoptera	Braconidae	Microgastrinae	Microplitis	Microplitis varicolor	Jose Leonardo Fernandez	jftriana@uoguelph.ca						NULL		H. Goulet			Canada	Ontario	Ottawa		Ottwa city garden	45.3560982	-75.706902		
CNCHX204-09	WMIC 0204	WMIC 0204	WMIC 0204	657				WMIC 0204	Arthropoda	Insecta	Hymenoptera	Braconidae	Microgastrinae	Microplitis	Microplitis jft09	Jose Leonardo Fernandez	jftriana@uoguelph.ca						NULL		H. Goulet			Canada	Ontario	Ottawa		Ottwa city garden	45.3560982	-75.706902		
CNCHX205-09	WMIC 0205	WMIC 0205	WMIC 0205	657				WMIC 0205	Arthropoda	Insecta	Hymenoptera	Braconidae	Microgastrinae	Microplitis	Microplitis jft09	Jose Leonardo Fernandez	jftriana@uoguelph.ca						NULL		H. Goulet			Canada	Ontario	Ottawa		Ottwa city garden	45.3560982	-75.706902		
CNCHX206-09	WMIC 0206	WMIC 0206	WMIC 0206	620				WMIC 0206	Arthropoda	Insecta	Hymenoptera	Braconidae	Microgastrinae	Microplitis	Microplitis jft09	Jose Leonardo Fernandez	jftriana@uoguelph.ca						NULL		H. Goulet			Canada	Ontario	Ottawa		Ottwa city garden	45.3560982	-75.706902		
CNCHX208-09	WMIC 0208	WMIC 0208	WMIC 0208	657				WMIC 0208	Arthropoda	Insecta	Hymenoptera	Braconidae	Microgastrinae	Microplitis	Microplitis jft09	Jose Leonardo Fernandez	jftriana@uoguelph.ca						NULL		H. Goulet			Canada	Ontario	Ottawa		Ottwa city garden	45.3560982	-75.706902		
CNCHX209-09	WMIC 0209	WMIC 0209	WMIC 0209	606				WMIC 0209	Arthropoda	Insecta	Hymenoptera	Braconidae	Microgastrinae	Microplitis	Microplitis varicolor	Jose Leonardo Fernandez	jftriana@uoguelph.ca						NULL		H. Goulet			Canada	Ontario	Ottawa		Ottwa city garden	45.3560982	-75.706902		
CNCHX210-09	WMIC 0210	WMIC 0210	WMIC 0210	657				WMIC 0210	Arthropoda	Insecta	Hymenoptera	Braconidae	Microgastrinae	Microplitis	Microplitis jft09	Jose Leonardo Fernandez	jftriana@uoguelph.ca						NULL		H. Goulet			Canada	Ontario	Ottawa		Ottwa city garden	45.3560982	-75.706902		
CNCHX211-09	WMIC 0211	WMIC 0211	WMIC 0211	657				WMIC 0211	Arthropoda	Insecta	Hymenoptera	Braconidae	Microgastrinae	Microplitis	Microplitis jft09	Jose Leonardo Fernandez	jftriana@uoguelph.ca						NULL		H. Goulet			Canada	Ontario	Ottawa		Ottwa city garden	45.3560982	-75.706902		
CNCHX215-09	WMIC 0215	WMIC 0215	WMIC 0215	657				WMIC 0215	Arthropoda	Insecta	Hymenoptera	Braconidae	Microgastrinae	Microplitis	Microplitis jft09	Jose Leonardo Fernandez	jftriana@uoguelph.ca						NULL		H. Goulet			Canada	Ontario	Ottawa		Ottwa city garden	45.3560982	-75.706902		
CNCHX217-09	WMIC 0217	WMIC 0217	WMIC 0217	657				WMIC 0217	Arthropoda	Insecta	Hymenoptera	Braconidae	Microgastrinae	Microplitis	Microplitis jft09	Jose Leonardo Fernandez	jftriana@uoguelph.ca						NULL		H. Goulet			Canada	Ontario	Ottawa		Ottwa city garden	45.3560982	-75.706902		
CNCHX219-09	WMIC 0219	WMIC 0219	WMIC 0219	657				WMIC 0219	Arthropoda	Insecta	Hymenoptera	Braconidae	Microgastrinae	Microplitis	Microplitis varicolor	Jose Leonardo Fernandez	jftriana@uoguelph.ca						NULL		H. Goulet			Canada	Ontario	Ottawa		Ottwa city garden	45.3560982	-75.706902		
CNCHX220-09	WMIC 0220	WMIC 0220	WMIC 0220	657				WMIC 0220	Arthropoda	Insecta	Hymenoptera	Braconidae	Microgastrinae	Microplitis	Microplitis jft09	Jose Leonardo Fernandez	jftriana@uoguelph.ca						NULL		H. Goulet			Canada	Ontario	Ottawa		Ottwa city garden	45.3560982	-75.706902		
CNCHX222-09	WMIC 0222	WMIC 0222	WMIC 0222	657				WMIC 0222	Arthropoda	Insecta	Hymenoptera	Braconidae	Microgastrinae	Microplitis	Microplitis varicolor	Jose Leonardo Fernandez	jftriana@uoguelph.ca						NULL		H. Goulet			Canada	Ontario	Ottawa		Ottwa city garden	45.3560982	-75.706902		
CNCHX223-09	WMIC 0223	WMIC 0223	WMIC 0223	657				WMIC 0223	Arthropoda	Insecta	Hymenoptera	Braconidae	Microgastrinae	Microplitis	Microplitis varicolor	Jose Leonardo Fernandez	jftriana@uoguelph.ca						NULL		H. Goulet			Canada	Ontario	Ottawa		Ottwa city garden	45.3560982	-75.706902		
CNCHX224-09	WMIC 0224	WMIC 0224	WMIC 0224	657				WMIC 0224	Arthropoda	Insecta	Hymenoptera	Braconidae	Microgastrinae	Microplitis	Microplitis varicolor	Jose Leonardo Fernandez	jftriana@uoguelph.ca						NULL		H. Goulet			Canada	Ontario	Ottawa		Ottwa city garden	45.3560982	-75.706902		
CNCHX225-09	WMIC 0225	WMIC 0225	WMIC 0225	657				WMIC 0225	Arthropoda	Insecta	Hymenoptera	Braconidae	Microgastrinae	Microplitis	Microplitis varicolor	Jose Leonardo Fernandez	jftriana@uoguelph.ca						NULL		H. Goulet			Canada	Ontario	Ottawa		Ottwa city garden	45.3560982	-75.706902		
CNCHX226-09	WMIC 0226	WMIC 0226	WMIC 0226	657				WMIC 0226	Arthropoda	Insecta	Hymenoptera	Braconidae	Microgastrinae	Microplitis	Microplitis varicolor	Jose Leonardo Fernandez	jftriana@uoguelph.ca						NULL		H. Goulet			Canada	Ontario	Ottawa		Ottwa city garden	45.3560982	-75.706902		
CNCHX797-09	WMIC 0512	WMIC 0512	WMIC 0512	657				WMIC 0512	Arthropoda	Insecta	Hymenoptera	Braconidae	Microgastrinae	Microplitis	Microplitis jft09	Jose Leonardo Fernandez	jftriana@uoguelph.ca						NULL		L. Gualtieri	07-Jul-2003		Canada	Ontario			London	43.0294991	-81.205498		
CNCHY034-07	HYM00000306	HYM00000306	HYM00000306	290				HYM00000306	Arthropoda	Insecta	Hymenoptera	Braconidae	Microgastrinae	Cotesia	Cotesia xylina	Jose Leonardo Fernandez	jftriana@uoguelph.ca		S	A					Boudreault & Goule	07-Jul-2006		Canada	Yukon Territory			E. of Haines Junction, f	60.8520012	-137.172	664	
CNCHY035-07	HYM00000307	HYM00000307	HYM00000307	288				HYM00000307	Arthropoda	Insecta	Hymenoptera	Braconidae	Microgastrinae	Cotesia	Cotesia xylina	Jose Leonardo Fernandez	jftriana@uoguelph.ca		S	A					Boudreault & Goule	10-Jul-2006		Canada	Yukon Territory			Whitehorse, fallow field	60.7099991	-135.05499	689	
CNCHY036-07	HYM00000308	HYM00000308	HYM00000308	290				HYM00000308	Arthropoda	Insecta	Hymenoptera	Braconidae	Microgastrinae	Cotesia	Cotesia xylina	Jose Leonardo Fernandez	jftriana@uoguelph.ca		S	A					Boudreault & Goule	10-Jul-2006		Canada	Yukon Territory			Whitehorse, fallow field	60.7099991	-135.05499	689	
CNCHY037-07	HYM00000309	HYM00000309	HYM00000309	290				HYM00000309	Arthropoda	Insecta	Hymenoptera	Braconidae	Microgastrinae	Cotesia	Cotesia xylina	Jose Leonardo Fernandez	jftriana@uoguelph.ca		S	A					Boudreault & Goule	11-Jul-2006		Canada	Yukon Territory			Whitehorse, hay field	60.7410011	-135.073	640	
CNCHY038-07	HYM00000310	HYM00000310	HYM00000310	290				HYM00000310	Arthropoda	Insecta	Hymenoptera	Braconidae	Microgastrinae	Cotesia	Cotesia xylina	Jose Leonardo Fernandez	jftriana@uoguelph.ca		S	A					Boudreault & Goule	11-Jul-2006		Canada	Yukon Territory			Whitehorse, hay field	60.7410011	-135.073	640	
CNCHY039-07	HYM00000311	HYM00000311	HYM00000311	657				HYM00000311	Arthropoda	Insecta	Hymenoptera	Braconidae	Microgastrinae	Cotesia	Cotesia xylina	Jose Leonardo Fernandez	jftriana@uoguelph.ca		S	A					Boudreault & Goule	11-Jul-2006		Canada	Yukon Territory			Whitehorse, hay field	60.7410011	-135.073	640	
CNCHY041-07	HYM00000313	HYM00000313	HYM00000313	290				HYM00000313	Arthropoda	Insecta	Hymenoptera	Braconidae	Microgastrinae	Cotesia	Cotesia xylina	Jose Leonardo Fernandez	jftriana@uoguelph.ca		S	A					Boudreault & Goule	11-Jul-2006		Canada	Yukon Territory			Whitehorse, hay field	60.7410011	-135.073	640	
CNCHY042-07	HYM00000314	HYM00000314	HYM00000314	628				HYM00000314	Arthropoda	Insecta	Hymenoptera	Braconidae	Microgastrinae	Cotesia	Cotesia xylina	Jose Leonardo Fernandez	jftriana@uoguelph.ca		S	A					Boudreault & Goule	11-Jul-2006		Canada	Yukon Territory			Whitehorse, hay field	60.7410011	-135.073	640	
CNCHY043-07	HYM00000315	HYM00000315	HYM00000315	290				HYM00000315	Arthropoda	Insecta	Hymenoptera	Braconidae	Microgastrinae	Cotesia	Cotesia xylina	Jose Leonardo Fernandez	jftriana@uoguelph.ca		S	A					Boudreault & Goule	11-Jul-2006		Canada	Yukon Territory			Whitehorse, hay field	60.7410011	-135.073	640	
CNCHY044-07	HYM00000316	HYM00000316	HYM00000316	288				HYM00000316	Arthropoda	Insecta	Hymenoptera	Braconidae	Microgastrinae	Cotesia	Cotesia xylina	Jose Leonardo Fernandez	jftriana@uoguelph.ca		S	A					Boudreault & Goule	11-Jul-2006		Canada	Yukon Territory			Whitehorse, hay field	60.7410011	-135.073	640	
CNCHY045-07	HYM00000317	HYM00000317	HYM00000317	290				HYM00000317	Arthropoda	Insecta	Hymenoptera	Braconidae	Microgastrinae	Cotesia	Cotesia xylina	Jose Leonardo Fernandez	jftriana@uoguelph.ca		S	A					Boudreault & Goule	11-Jul-2006		Canada	Yukon Territory			Whitehorse, hay field	60.7410011	-135.073	640	
CNCHY046-07	HYM00000318	HYM00000318	HYM00000318	625				HYM00000318	Arthropoda	Insecta	Hymenoptera	Braconidae	Microgastrinae	Cotesia	Cotesia xylina	Jose Leonardo Fernandez	jftriana@uoguelph.ca		S	A					Boudreault & Goule	11-Jul-2006		Canada	Yukon Territory			Whitehorse, hay field	60.7410011	-135.073	640	
CNCHY047-07	HYM00000319	HYM00000319	HYM00000319	289				HYM00000319	Arthropoda	Insecta	Hymenoptera	Braconidae	Microgastrinae	Cotesia	Cotesia xylina	Jose Leonardo Fernandez	jftriana@uoguelph.ca		S	A					Boudreault & Goule	11-Jul-2006		Canada	Yukon Territory			Whitehorse, hay field	60.7410011	-135.073	640	

Table S5: All collection and accession information associated with specimens analysed here.

ProcessID	Sample ID	Field Num	Catalog Num	COI-5P Seq.	COI GenBank	28S Seq. Length	28S GenBan	Sample ID	Phylum	Class	Order	Family	Subfamily	Genus	Species	Identifier	Identifier email	Sex	Reproduction	Life Stage	Extra Info	Notes	Voucher Type	Tissue Type	Collectors	Date Collected	Continent	Country/Ocean	Province	Region	Sector	Exact Site	Lat	Lon	Elev	GPS Source
CNCHY059-07	HYM00000331	HYM00000331	HYM00000331	657				HYM00000331	Arthropoda	Insecta	Hymenoptera	Braconidae	Microgastrinae	Pholetesor	Pholetesor bedelliae	Jose Leonardo Fernandez	jftriana@uoguelph.ca								Boudreault & Goule	10-Jul-2006		Canada	Yukon Territory			Whitehorse, fallow field	60.7099991	-135.05499	689	
CNCHY061-07	HYM00000333	HYM00000333	HYM00000333	290				HYM00000333	Arthropoda	Insecta	Hymenoptera	Braconidae	Microgastrinae	Dolichogenidea	Dolichogenidea jft03	Jose Leonardo Fernandez	jftriana@uoguelph.ca				Undetermined sp. 1				Boudreault & Goule	10-Jul-2006		Canada	Yukon Territory			Whitehorse, fallow field	60.7099991	-135.05499	689	
CNCHY063-07	HYM00000335	HYM00000335	HYM00000335	626				HYM00000335	Arthropoda	Insecta	Hymenoptera	Braconidae	Microgastrinae	Microgaster	Microgaster jft10	Jose Leonardo Fernandez	jftriana@uoguelph.ca		S	A					Boudreault & Goule	10-Jul-2006		Canada	Yukon Territory			Whitehorse, fallow field	60.7099991	-135.05499	689	
CNCHY064-07	HYM00000336	HYM00000336	HYM00000336	290				HYM00000336	Arthropoda	Insecta	Hymenoptera	Braconidae	Microgastrinae	Microgaster	Microgaster jft10	Jose Leonardo Fernandez	jftriana@uoguelph.ca		S	A					Boudreault & Goule	10-Jul-2006		Canada	Yukon Territory			Whitehorse, fallow field	60.7099991	-135.05499	689	
CNCHY070-07	HYM00000342	HYM00000342	HYM00000342	657				HYM00000342	Arthropoda	Insecta	Hymenoptera	Braconidae	Microgastrinae	Microplitis	Microplitis auripes	Jose Leonardo Fernandez	jftriana@uoguelph.ca				Microplitis spp				Boudreault & Goule	10-Jul-2006		Canada	Yukon Territory			Whitehorse, fallow field	60.7099991	-135.05499	689	
CNCHY076-07	HYM00000348	HYM00000348	HYM00000348	657				HYM00000348	Arthropoda	Insecta	Hymenoptera	Braconidae	Microgastrinae	Microplitis	Microplitis jft08	Jose Leonardo Fernandez	jftriana@uoguelph.ca				Microplitis spp				Boudreault & Goule	11-Jul-2006		Canada	Yukon Territory			Whitehorse, hay field	60.7410011	-135.073	640	
CNCHY078-07	HYM00000350	HYM00000350	HYM00000350	657				HYM00000350	Arthropoda	Insecta	Hymenoptera	Braconidae	Microgastrinae	Microplitis	Microplitis jft08	Jose Leonardo Fernandez	jftriana@uoguelph.ca				Microplitis spp				Boudreault & Goule	11-Jul-2006		Canada	Yukon Territory			Whitehorse, hay field	60.7410011	-135.073	640	
CNCHY189-07	HYM00000461	HYM00000461	HYM00000461	600				HYM00000461	Arthropoda	Insecta	Hymenoptera	Braconidae	Microgastrinae	Microgaster	Microgaster jft03	Jose Leonardo Fernandez	jftriana@uoguelph.ca		S	A					Goulet & Boudrealt	08-Jul-2006		Canada	Yukon Territory			Champagne	60.7900009	-136.437	733	
CNCHY190-07	HYM00000462	HYM00000462	HYM00000462	604				HYM00000462	Arthropoda	Insecta	Hymenoptera	Braconidae	Microgastrinae	Microgaster	Microgaster jft03	Jose Leonardo Fernandez	jftriana@uoguelph.ca		S	A					Goulet & Boudrealt	08-Jul-2006		Canada	Yukon Territory			Champagne	60.7900009	-136.437	733	
CNCHY191-07	HYM00000463	HYM00000463	HYM00000463	604				HYM00000463	Arthropoda	Insecta	Hymenoptera	Braconidae	Microgastrinae	Microgaster	Microgaster jft03	Jose Leonardo Fernandez	jftriana@uoguelph.ca		S	A					Goulet & Boudrealt	06-Jul-2006		Canada	Yukon Territory			Tahkini River road	60.875	-135.386	693	
CNCHY192-07	HYM00000464	HYM00000464	HYM00000464	596				HYM00000464	Arthropoda	Insecta	Hymenoptera	Braconidae	Microgastrinae	Microgaster	Microgaster jft03	Jose Leonardo Fernandez	jftriana@uoguelph.ca		S	A					Goulet & Boudrealt	06-Jul-2006		Canada	Yukon Territory			Kusawa Lake road	60.7060013	-136.075	699	
CNCHY193-07	HYM00000465	HYM00000465	HYM00000465	594				HYM00000465	Arthropoda	Insecta	Hymenoptera	Braconidae	Microgastrinae	Microgaster	Microgaster jft03	Jose Leonardo Fernandez	jftriana@uoguelph.ca		S	A					Goulet & Boudrealt	06-Jul-2006		Canada	Yukon Territory			Kusawa Lake road	60.7060013	-136.075	699	
CNCHY194-07	HYM00000466	HYM00000466	HYM00000466	603				HYM00000466	Arthropoda	Insecta	Hymenoptera	Braconidae	Microgastrinae	Microgaster	Microgaster jft03	Jose Leonardo Fernandez	jftriana@uoguelph.ca		S	A					Goulet & Boudrealt	06-Jul-2006		Canada	Yukon Territory			Kusawa Lake road	60.7060013	-136.075	699	
CNCHY195-07	HYM00000467	HYM00000467	HYM00000467	594				HYM00000467	Arthropoda	Insecta	Hymenoptera	Braconidae	Microgastrinae	Microgaster	Microgaster jft03	Jose Leonardo Fernandez	jftriana@uoguelph.ca		S	A					Goulet & Boudrealt	06-Jul-2006		Canada	Yukon Territory			Kusawa Lake road	60.7060013	-136.075	699	
CNCHY202-07	HYM00000474	HYM00000474	HYM00000474	657				HYM00000474	Arthropoda	Insecta	Hymenoptera	Braconidae	Microgastrinae	Microgaster	Microgaster jft03	Jose Leonardo Fernandez	jftriana@uoguelph.ca		S	A					Goulet & Boudrealt	08-Jul-2006		Canada	Yukon Territory			Champagne	60.7900009	-136.437	733	
CNCHY203-07	HYM00000475	HYM00000475	HYM00000475	599				HYM00000475	Arthropoda	Insecta	Hymenoptera	Braconidae	Microgastrinae	Microgaster	Microgaster jft03	Jose Leonardo Fernandez	jftriana@uoguelph.ca		S	A					Goulet & Boudrealt	08-Jul-2006		Canada	Yukon Territory			Champagne	60.7900009	-136.437	733	
CNCHY204-07	HYM00000476	HYM00000476	HYM00000476	657				HYM00000476	Arthropoda	Insecta	Hymenoptera	Braconidae	Microgastrinae	Microgaster	Microgaster jft03	Jose Leonardo Fernandez	jftriana@uoguelph.ca		S	A					Goulet & Boudrealt	08-Jul-2006		Canada	Yukon Territory			Champagne	60.7900009	-136.437	733	
CNCHY205-07	HYM00000477	HYM00000477	HYM00000477	624				HYM00000477	Arthropoda	Insecta	Hymenoptera	Braconidae	Microgastrinae	Microgaster	Microgaster jft03	Jose Leonardo Fernandez	jftriana@uoguelph.ca		S	A					Goulet & Boudrealt	06-Jul-2006		Canada	Yukon Territory			Tahkini River road	60.875	-135.386	693	
CNCHY206-07	HYM00000478	HYM00000478	HYM00000478	599				HYM00000478	Arthropoda	Insecta	Hymenoptera	Braconidae	Microgastrinae	Microgaster	Microgaster jft03	Jose Leonardo Fernandez	jftriana@uoguelph.ca		S	A					Goulet & Boudrealt	06-Jul-2006		Canada	Yukon Territory			Tahkini River road	60.875	-135.386	693	
CNCHY207-07	HYM00000479	HYM00000479	HYM00000479	657				HYM00000479	Arthropoda	Insecta	Hymenoptera	Braconidae	Microgastrinae	Microgaster	Microgaster jft03	Jose Leonardo Fernandez	jftriana@uoguelph.ca		S	A					Goulet & Boudrealt	06-Jul-2006		Canada	Yukon Territory			Tahkini River road	60.875	-135.386	693	
CNCHY208-07	HYM00000480	HYM00000480	HYM00000480	611				HYM00000480	Arthropoda	Insecta	Hymenoptera	Braconidae	Microgastrinae	Microgaster	Microgaster jft03	Jose Leonardo Fernandez	jftriana@uoguelph.ca		S	A					Goulet & Boudrealt	08-Jul-2006		Canada	Yukon Territory			Champagne	60.7900009	-136.437	733	
CNCHY209-07	HYM00000481	HYM00000481	HYM00000481	602				HYM00000481	Arthropoda	Insecta	Hymenoptera	Braconidae	Microgastrinae	Microgaster	Microgaster jft03	Jose Leonardo Fernandez	jftriana@uoguelph.ca		S	A					Goulet & Boudrealt	06-Jul-2006		Canada	Yukon Territory			Tahkini River road	60.875	-135.386	693	
CNCHY210-07	HYM00000482	HYM00000482	HYM00000482	657				HYM00000482	Arthropoda	Insecta	Hymenoptera	Braconidae	Microgastrinae	Microgaster	Microgaster jft10	Jose Leonardo Fernandez	jftriana@uoguelph.ca		S	A					Goulet & Boudrealt	06-Jul-2006		Canada	Yukon Territory			Kusawa Lake road	60.7060013	-136.075	699	
CNCHY212-07	HYM00000484	HYM00000484	HYM00000484	563				HYM00000484	Arthropoda	Insecta	Hymenoptera	Braconidae	Microgastrinae	Microgaster	Microgaster jft03	Jose Leonardo Fernandez	jftriana@uoguelph.ca		S	A					Goulet & Boudrealt	08-Jul-2006		Canada	Yukon Territory			Champagne	60.7900009	-136.437	733	
CNCHY214-07	HYM00000486	HYM00000486	HYM00000486	657				HYM00000486	Arthropoda	Insecta	Hymenoptera	Braconidae	Microgastrinae	Microgaster	Microgaster jft09	Jose Leonardo Fernandez	jftriana@uoguelph.ca		S	A					Goulet & Boudrealt	19-Jul-2006		Canada	Yukon Territory			Top of the world, Hwy,	64.0950012	-140.951		
CNCHY215-07	HYM00000487	HYM00000487	HYM00000487	657				HYM00000487	Arthropoda	Insecta	Hymenoptera	Braconidae	Microgastrinae	Microgaster	Microgaster jft10	Jose Leonardo Fernandez	jftriana@uoguelph.ca		S	A					Goulet & Boudrealt	06-Jul-2006		Canada	Yukon Territory			Tahkini River road	60.875	-135.386	693	
CNCHY216-07	HYM00000488	HYM00000488	HYM00000488	599				HYM00000488	Arthropoda	Insecta	Hymenoptera	Braconidae	Microgastrinae	Microgaster	Microgaster jft03	Jose Leonardo Fernandez	jftriana@uoguelph.ca		S	A					Goulet & Boudrealt	14-Jul-2006		Canada	Yukon Territory			Marsh Lake, Alaska Hw	60.5089989	-134.29201		
CNCHY217-07	HYM00000489	HYM00000489	HYM00000489	595				HYM00000489	Arthropoda	Insecta	Hymenoptera	Braconidae	Microgastrinae	Microplitis	Microplitis varicolor	Jose Leonardo Fernandez	jftriana@uoguelph.ca								Goulet & Boudrealt	15-Jul-2006		Canada	Yukon Territory			Pelly Crossing	62.8310013	-136.58501	495	
CNCHY218-07	HYM00000490	HYM00000490	HYM00000490	582				HYM00000490	Arthropoda	Insecta	Hymenoptera	Braconidae	Microgastrinae	Microplitis	Microplitis varicolor	Jose Leonardo Fernandez	jftriana@uoguelph.ca								Goulet & Boudrealt	19-Jul-2006		Canada	Yukon Territory			Top of the world, Hwy,	64.0950012	-140.951		
CNCHY221-07	HYM00000493	HYM00000493	HYM00000493	657				HYM00000493	Arthropoda	Insecta	Hymenoptera	Braconidae	Microgastrinae	Microplitis	Microplitis varicolor	Jose Leonardo Fernandez	jftriana@uoguelph.ca								Goulet & Boudrealt	19-Jul-2006		Canada	Yukon Territory			Top of the world, Hwy,	64.0950012	-140.951		
CNCHY224-07	HYM00000496	HYM00000496	HYM00000496	593				HYM00000496	Arthropoda	Insecta	Hymenoptera	Braconidae	Microgastrinae	Microplitis	Microplitis varicolor	Jose Leonardo Fernandez	jftriana@uoguelph.ca								Goulet & Boudrealt	19-Jul-2006		Canada	Yukon Territory			Top of the world, Hwy,	64.0950012	-140.951		
CNCHY227-07	HYM00000499	HYM00000499	HYM00000499	576				HYM00000499	Arthropoda	Insecta	Hymenoptera	Braconidae	Microgastrinae	Microplitis	Microplitis jft06	Jose Leonardo Fernandez	jftriana@uoguelph.ca								Goulet & Boudrealt	13-Jul-2006		Canada	Yukon Territory			Kluane Nat. Park	60.6829987	-138.005	625	
CNCHY236-07	HYM00000508	HYM00000508	HYM00000508	657				HYM00000508	Arthropoda	Insecta	Hymenoptera	Braconidae	Microgastrinae	Microplitis	Microplitis jft08	Jose Leonardo Fernandez	jftriana@uoguelph.ca								Goulet & Boudrealt	05-Jul-2006		Canada	Yukon Territory			Fish Lake road	60.6879997	-135.23801	1107	
CNCHY242-07	HYM00000514	HYM00000514	HYM00000514	657				HYM00000514	Arthropoda	Insecta	Hymenoptera	Braconidae	Microgastrinae	Microplitis	Microplitis auripes	Jose Leonardo Fernandez	jftriana@uoguelph.ca								Goulet & Boudrealt	10-Jul-2006		Canada	Yukon Territory			Whitehorse	60.9539986	-135.255	689	
CNCHY243-07	HYM00000515	HYM00000515	HYM00000515	657				HYM00000515	Arthropoda	Insecta	Hymenoptera	Braconidae	Microgastrinae	Microplitis	Microplitis auripes	Jose Leonardo Fernandez	jftriana@uoguelph.ca								Goulet & Boudrealt	16-Jul-2006		Canada	Yukon Territory			Pelly Crossing	62.8310013	-136.58501	495	
CNCHY244-07	HYM00000516	HYM00000516	HYM00000516	598				HYM00000516	Arthropoda	Insecta	Hymenoptera	Braconidae	Microgastrinae	Microplitis	Microplitis auripes	Jose Leonardo Fernandez	jftriana@uoguelph.ca								Goulet & Boudrealt	16-Jul-2006		Canada	Yukon Territory			Pelly Crossing	62.8310013	-136.58501	495	
CNCHY248-07	HYM00000520	HYM00000520	HYM00000520	629				HYM00000520	Arthropoda	Insecta	Hymenoptera	Braconidae	Microgastrinae	Microplitis	Microplitis jft08	Jose Leonardo Fernandez	jftriana@uoguelph.ca								Goulet & Boudrealt	05-Jul-2006		Canada	Yukon Territory			Fish Lake road	60.6879997	-135.23801	1107	
CNCHY255-07	HYM00000527	HYM00000527	HYM00000527	595				HYM00000527	Arthropoda	Insecta	Hymenoptera	Braconidae	Microgastrinae	Microplitis	Microplitis varicolor	Jose Leonardo Fernandez	jftriana@uoguelph.ca								Goulet & Boudrealt	19-Jul-2006		Canada	Yukon Territory			Top of the world, Hwy,	64.0950012	-140.951		
CNCHY259-07	HYM00000531	HYM00000531	HYM00000531	576				HYM00000531	Arthropoda	Insecta	Hymenoptera	Braconidae	Microgastrinae	Microplitis	Microplitis varicolor	Jose Leonardo Fernandez	jftriana@uoguelph.ca								Goulet & Boudrealt	15-Jul-2006		Canada	Yukon Territory			Pelly Crossing	62.8310013	-136.58501	495	
CNCHY262-07	HYM00000534	HYM00000534	HYM00000534	574				HYM00000534	Arthropoda	Insecta	Hymenoptera	Braconidae	Microgastrinae	Microplitis	Microplitis varicolor	Jose Leonardo Fernandez	jftriana@uoguelph.ca								Goulet & Boudrealt	15-Jul-2006		Canada	Yukon Territory			Pelly Crossing	62.8310013	-136.58501	495	
CNCHY264-07	HYM00000536	HYM00000536	HYM00000536	574				HYM00000536	Arthropoda	Insecta	Hymenoptera	Braconidae	Microgastrinae	Microplitis	Microplitis varicolor	Jose Leonardo Fernandez	jftriana@uoguelph.ca								Goulet & Boudrealt	13-Jul-2006		Canada	Yukon Territory			Kluane Nat. Park	60.6829987	-138.005	625	
CNCHY270-07	HYM00000542	HYM00000542	HYM00000542	657				HYM00000542	Arthropoda	Insecta	Hymenoptera	Braconidae	Microgastrinae	Protapanteles	Protapanteles jft01	Jose Leonardo Fernandez	jftriana@uoguelph.ca								Goulet & Boudrealt	15-Jul-2006		Canada	Yukon Territory			Pelly Crossing	62.8310013	-136.58501	495	
CNCHY282-07	HYM00000554	HYM00000554	HYM00000554	589				HYM00000554	Arthropoda	Insecta	Hymenoptera	Braconidae	Microgastrinae	Microplitis	Microplitis jft06	Jose Leonardo Fernandez	jftriana@uoguelph.ca								Goulet & Boudrealt	18-Jul-2006		Canada	Yukon Territory			Dempster Hwy. Km 108	64.7890015	-138.35899	1051	
CNCHY284-07	HYM00000556	HYM00000556	HYM00000556	657				HYM00000556	Arthropoda	Insecta	Hymenoptera	Braconidae	Microgastrinae	Dolichogenidea	Dolichogenidea jft03	Jose Leonardo Fernandez	jftriana@uoguelph.ca								Goulet & Boudrealt	15-Jul-2006		Canada	Yukon Territory			Pelly Crossing	62.8310013	-136.58501	495	
CNCHY286-07	HYM00000558	HYM00000558	HYM00000558	657				HYM00000558	Arthropoda	Insecta	Hymenoptera	Braconidae	Microgastrinae	Dolichogenidea	Dolichogenidea jft03	Jose Leonardo Fernandez	jftriana@uoguelph.ca								Goulet & Boudrealt	15-Jul-2006		Canada	Yukon Territory			Pelly Crossing	62.8310013	-136.58501	495	
CNCHY291-07	HYM00000563	HYM00000563	HYM00000563	657				HYM00000563	Arthropoda	Insecta	Hymenoptera	Braconidae	Microgastrinae	Dolichogenidea	Dolichogenidea jft03	Jose Leonardo Fernandez	jftriana@uoguelph.ca								Goulet & Boudrealt	15-Jul-2006		Canada	Yukon Territory			Pelly Crossing	62.8310013	-136.58501	495	
CNCHY292-07	HYM00000564	HYM00000564	HYM00000564	657				HYM00000564	Arthropoda	Insecta	Hymenoptera	Braconidae	Microgastrinae	Dolichogenidea	Dolichogenidea jft03	Jose Leonardo Fernandez	jftriana@uoguelph.ca								Goulet & Boudrealt	15-Jul-2006		Canada	Yukon Territory			Pelly Crossing	62.8310013	-136.58501	495	
CNCHY294-07	HYM00000566	HYM00000566	HYM00000566	657				HYM00000566	Arthropoda	Insecta	Hymenoptera	Braconidae	Microgastrinae	Dolichogenidea	Dolichogenidea jft03	Jose Leonardo Fernandez	jftriana@uoguelph.ca								Goulet & Boudrealt	15-Jul-2006		Canada	Yukon Territory			Pelly Crossing	62.8310013	-136.58501	495	
CNCHY295-07	HYM00000567	HYM00000567	HYM00000567	616				HYM00000567	Arthropoda	Insecta	Hymenoptera	Braconidae	Microgastrinae	Dolichogenidea	Dolichogenidea jft03	Jose Leonardo Fernandez	jftriana@uoguelph.ca								Goulet & Boudrealt	15-Jul-2006		Canada	Yukon Territory			Pelly Crossing	62.8310013	-136.58501	495	
CNCHY297-07	HYM00000569	HYM00000569	HYM00000569	606				HYM00000569	Arthropoda	Insecta	Hymenoptera	Braconidae	Microgastrinae	Dolichogenidea	Dolichogenidea jft03	Jose Leonardo Fernandez	jftriana@uoguelph.ca								Goulet & Boudrealt	15-Jul-2006		Canada	Yukon Territory			Pelly Crossing	62.8310013	-136.58501	495	
CNCHY300-07	HYM00000572	HYM00000572	HYM00000572	657				HYM00000572	Arthropoda	Insecta	Hymenoptera	Braconidae	Microgastrinae	Dolichogenidea	Dolichogenidea jft03	Jose Leonardo Fernandez	jftriana@uoguelph.ca								Goulet & Boudrealt	15-Jul-2006		Canada	Yukon Territory			Pelly Crossing	62.8310013	-136.58501	495	
CNCHY301-07	HYM00000573	HYM00000573	HYM00000573	657				HYM00000573	Arthropoda	Insecta	Hymenoptera	Braconidae	Microgastrinae	Apanteles	Apanteles jft01	Jose Leonardo Fernandez	jftriana@uoguelph.ca								Goulet & Boudrealt	15-Jul-2006		Canada	Yukon Territory			Pelly Crossing	62.8310013	-136.58501	495	
CNCHY302-07	HYM00000574	HYM00000574	HYM00000574	657				HYM00000574	Arthropoda	Insecta	Hymenoptera	Braconidae	Microgastrinae	Dolichogenidea	Dolichogenidea jft03	Jose Leonardo Fernandez	jftriana@uoguelph.ca								Goulet & Boudrealt	15-Jul-2006		Canada	Yukon Territory			Pelly Crossing	62.8310013	-136.58501	495	
CNCHY303-07	HYM00000575	HYM00000575	HYM00000575	657				HYM00000575	Arthropoda	Insecta	Hymenoptera	Braconidae	Microgastrinae	Dolichogenidea	Dolichogenidea jft03	Jose Leonardo Fernandez	jftriana@uoguelph.ca								Goulet & Boudrealt	15-Jul-2006		Canada	Yukon Territory			Pelly Crossing	62.8310013	-136.58501	495	
CNCHY304-07	HYM00000576	HYM00000576	HYM00000576	623				HYM00000576	Arthropoda	Insecta	Hymenoptera	Braconidae	Microgastrinae	Dolichogenidea	Dolichogenidea jft03	Jose Leonardo Fernandez	jftriana@uoguelph.ca								Goulet & Boudrealt	15-Jul-2006		Canada	Yukon Territory			Pelly Crossing	62.8310013	-136.58501	495	
CNCHY305-07	HYM00000577	HYM00000577	HYM00000577	657				HYM00000577	Arthropoda	Insecta	Hymenoptera	Braconidae	Microgastrinae	Dolichogenidea	Dolichogenidea jft03	Jose Leonardo Fernandez	jftriana@uoguelph.ca								Goulet & Boudrealt	15-Jul-2006		Canada	Yukon Territory			Pelly Crossing	62.8310013	-136.58501	495	
CNCHY306-07	HYM00000578	HYM00000578	HYM00000578	657				HYM00000578	Arthropoda	Insecta	Hymenoptera	Braconidae	Microgastrinae	Dolichogenidea	Dolichogenidea jft03	Jose Leonardo Fernandez	jftriana@uoguelph.ca								Goulet & Boudrealt	06-Jul-2006		Canada	Yukon Territory			Tahkini River road	60.875	-135.386	693	
CNCHY314-07	HYM00000586	HYM00000586	HYM00000586	657				HYM00000586	Arthropoda	Insecta	Hymenoptera	Braconidae	Microgastrinae	Dolichogenidea	Dolichogenidea jft02	Jose Leonardo Fernandez	jftriana@uoguelph.ca								Goulet & Boudrealt	16-Jul-2006		Canada	Yukon Territory			Pelly Crossing	62.8310013	-136.58501	495	
CNCHY317-07	HYM00000589	HYM00000589	HYM00000589	582				HYM00000589	Arthropoda	Insecta	Hymenoptera	Braconidae	Microgastrinae	Dolichogenidea	Dolichogenidea jft03	Jose Leonardo Fernandez	jftriana@uoguelph.ca								Goulet & Boudrealt	15-Jul-2006		Canada	Yukon Territory			Pelly Crossing	62.8310013	-136.58501	495	
CNCHY318-07	HYM00000590	HYM00000590	HYM00000590	657				HYM00000590	Arthropoda	Insecta	Hymenoptera	Braconidae	Microgastrinae	Dolichogenidea	Dolichogenidea jft06	Jose Leonardo Fernandez	jftriana@uoguelph.ca								Goulet & Boudrealt	13-Jul-2006		Canada	Yukon Territory			Kluane Nat. Park	60.6829987	-138.005	625	
CNCHY319-07	HYM00000591	HYM00000591	HYM00000591	657				HYM00000591	Arthropoda	Insecta	Hymenoptera	Braconidae	Microgastrinae	Cotesia	Cotesia jft09	Jose Leonardo Fernandez	jftriana@uoguelph.ca		S	A					Goulet & Boudrealt	08-Jul-2006		Canada	Yukon Territory			Champagne	60.7900009	-136.437	733	
CNCHY324-07	HYM00000596	HYM00000596	HYM00000596	657				HYM00000596	Arthropoda	Insecta	Hymenoptera	Braconidae	Microgastrinae	Dolichogenidea	Dolichogenidea jft01	Jose Leonardo Fernandez	jftriana@uoguelph.ca								Goulet & Boudrealt	06-Jul-2006		Canada	Yukon Territory			Tahkini River road	60.875	-135.386	693	
CNCHY328-07	HYM00000600	HYM00000600	HYM00000600	657				HYM00000600	Arthropoda	Insecta	Hymenoptera	Braconidae	Microgastrinae	Apanteles	Apanteles jft01	Jose Leonardo Fernandez	jftriana@uoguelph.ca								Goulet & Boudrealt	05-Jul-2006		Canada	Yukon Territory			Fish Lake road	60.6879997	-135.23801	1107	
CNCHY329-07	HYM00000601	HYM00000601	HYM00000601	657				HYM00000601	Arthropoda	Insecta	Hymenoptera	Braconidae	Microgastrinae	Apanteles	Apanteles jft01	Jose Leonardo Fernandez	jftriana@uoguelph.ca								Goulet & Boudrealt	05-Jul-2006		Canada	Yukon Territory			Fish Lake road	60.6879997	-135.23801	1107	
CNCHY330-07	HYM00000602	HYM00000602	HYM00000602	621				HYM00000602	Arthropoda	Insecta	Hymenoptera	Braconidae	Microgastrinae	Apanteles	Apanteles jft01	Jose Leonardo Fernandez	jftriana@uoguelph.ca								Goulet & Boudrealt	05-Jul-2006		Canada	Yukon Territory			Fish Lake road	60.6879997	-135.23801	1107	
CNCHY331-07	HYM00000603	HYM00000603	HYM00000603	657				HYM00000603	Arthropoda	Insecta	Hymenoptera	Braconidae	Microgastrinae	Pholetesor	Pholetesor viminetorum	Jose Leonardo Fernandez	jftriana@uoguelph.ca								Goulet & Boudrealt	05-Jul-2006		Canada	Yukon Territory			Fish Lake road	60.6879997	-135.23801	1107	
CNCHY332-07	HYM00000604	HYM00000604	HYM00000604	657				HYM00000604	Arthropoda	Insecta	Hymenoptera	Braconidae	Microgastrinae	Pholetesor	Pholetesor viminetorum	Jose Leonardo Fernandez	jftriana@uoguelph.ca								Goulet & Boudrealt	05-Jul-2006		Canada	Yukon Territory			Fish Lake road	60.6879997	-135.23801	1107	
CNCHY333-07	HYM00000605	HYM00000605	HYM00000605	657				HYM00000605	Arthropoda	Insecta	Hymenoptera	Braconidae	Microgastrinae	Apanteles	Apanteles jft01	Jose Leonardo Fernandez	jftriana@uoguelph.ca								Goulet & Boudrealt	05-Jul-2006		Canada	Yukon Territory			Fish Lake road	60.6879997	-135.23801	1107	
CNCHY334-07	HYM00000606	HYM00000606	HYM00000606	657				HYM00000606	Arthropoda	Insecta	Hymenoptera	Braconidae	Microgastrinae	Dolichogenidea	Dolichogenidea jft03	Jose Leonardo Fernandez	jftriana@uoguelph.ca								Goulet & Boudrealt	05-Jul-2006		Canada	Yukon Territory			Fish Lake road	60.6879997	-135.23801	1107	
CNCHY335-07	HYM00000607	HYM00000607	HYM00000607	657				HYM00000607	Arthropoda	Insecta	Hymenoptera	Braconidae	Microgastrinae	Dolichogenidea	Dolichogenidea jft03	Jose Leonardo Fernandez	jftriana@uoguelph.ca								Goulet & Boudrealt	18-Jul-2006		Canada	Yukon Territory			Dempster Hwy. Km 108	64.7890015	-138.35899	1051	
CNCHY338-07	HYM00000610	HYM00000610	HYM00000610	607				HYM00000610	Arthropoda	Insecta	Hymenoptera	Braconidae	Microgastrinae	Pholetesor	Pholetesor ornigis	Jose Leonardo Fernandez	jftriana@uoguelph.ca								Goulet & Boudrealt	05-Jul-2006		Canada	Yukon Territory			Fish Lake road	60.6879997	-135.23801	1107	
CNCHY340-07	HYM00000612	HYM00000612	HYM00000612	657				HYM00000612	Arthropoda	Insecta	Hymenoptera	Braconidae	Microgastrinae	Dolichogenidea	Dolichogenidea jft02	Jose Leonardo Fernandez	jftriana@uoguelph.ca								Goulet & Boudrealt	19-Jul-2006		Canada	Yukon Territory			Top of the world, Hwy,	64.0950012	-140.951		
CNCHY341-07	HYM00000613	HYM00000613	HYM00000613	657				HYM00000613	Arthropoda	Insecta	Hymenoptera	Braconidae	Microgastrinae	Dolichogenidea	Dolichogenidea jft03	Jose Leonardo Fernandez	jftriana@uoguelph.ca								Goulet & Boudrealt	19-Jul-2006		Canada	Yukon Territory			Top of the world, Hwy,	64.0950012	-140.951		
CNCHY344-07	HYM00000616	HYM00000616	HYM00000616	610				HYM00000616	Arthropoda	Insecta	Hymenoptera	Braconidae	Microgastrinae	Dolichogenidea	Dolichogenidea jft02	Jose Leonardo Fernandez	jftriana@uoguelph.ca								Goulet & Boudrealt	19-Jul-2006		Canada	Yukon Territory			Top of the world, Hwy,	64.0950012	-140.951		
CNCHY345-07	HYM00000617	HYM00000617	HYM00000617	657				HYM00000617	Arthropoda	Insecta	Hymenoptera	Braconidae	Microgastrinae	Dolichogenidea	Dolichogenidea jft02	Jose Leonardo Fernandez	jftriana@uoguelph.ca								Goulet & Boudrealt	14-Jul-2006		Canada	Yukon Territory			Marsh Lake, Alaska Hw	60.5089989	-134.29201		
CNCHY346-07	HYM00000618	HYM00000618	HYM00000618	600				HYM00000618	Arthropoda	Insecta	Hymenoptera	Braconidae	Microgastrinae	Dolichogenidea	Dolichogenidea jft02	Jose Leonardo Fernandez	jftriana@uoguelph.ca								Goulet & Boudrealt	08-Jul-2006		Canada	Yukon Territory			Champagne	60.7900009	-136.437	733	
CNCHY347-07	HYM00000619	HYM00000619	HYM00000619	657				HYM00000619	Arthropoda	Insecta	Hymenoptera	Braconidae	Microgastrinae	Dolichogenidea	Dolichogenidea jft03	Jose Leonardo Fernandez	jftriana@uoguelph.ca								Goulet & Boudrealt	05-Jul-2006		Canada	Yukon Territory			Fish Lake road	60.6879997	-135.23801	1107	
CNCHY348-07	HYM00000620	HYM00000620	HYM00000620	657				HYM00000620	Arthropoda	Insecta	Hymenoptera	Braconidae	Microgastrinae	Dolichogenidea	Dolichogenidea jft03	Jose Leonardo Fernandez	jftriana@uoguelph.ca								Goulet & Boudrealt	15-Jul-2006		Canada	Yukon Territory			Pelly Crossing	62.8310013	-136.58501	495	
CNCHY349-07	HYM00000621	HYM00000621	HYM00000621	657				HYM00000621	Arthropoda	Insecta	Hymenoptera	Braconidae	Microgastrinae	Dolichogenidea	Dolichogenidea jft03	Jose Leonardo Fernandez	jftriana@uoguelph.ca								Goulet & Boudrealt	15-Jul-2006		Canada	Yukon Territory			Pelly Crossing	62.8310013	-136.58501	495	
CNCHY350-07	HYM00000622	HYM00000622	HYM00000622	656				HYM00000622	Arthropoda	Insecta	Hymenoptera	Braconidae	Microgastrinae	Dolichogenidea	Dolichogenidea jft03	Jose Leonardo Fernandez	jftriana@uoguelph.ca								Goulet & Boudrealt	15-Jul-2006		Canada	Yukon Territory			Pelly Crossing	62.8310013	-136.58501	495	
CNCHY351-07	HYM00000623	HYM00000623	HYM00000623	657				HYM00000623	Arthropoda	Insecta	Hymenoptera	Braconidae	Microgastrinae	Dolichogenidea	Dolichogenidea jft03	Jose Leonardo Fernandez	jftriana@uoguelph.ca								Goulet & Boudrealt	15-Jul-2006		Canada	Yukon Territory			Pelly Crossing	62.8310013	-136.58501	495	
CNCHY352-07	HYM00000624	HYM00000624	HYM00000624	657				HYM00000624	Arthropoda	Insecta	Hymenoptera	Braconidae	Microgastrinae	Dolichogenidea	Dolichogenidea jft03	Jose Leonardo Fernandez	jftriana@uoguelph.ca								Goulet & Boudrealt	15-Jul-2006		Canada	Yukon Territory			Pelly Crossing	62.8310013	-136.58501	495	
CNCHY353-07	HYM00000625	HYM00000625	HYM00000625	657				HYM00000625	Arthropoda	Insecta	Hymenoptera	Braconidae	Microgastrinae	Dolichogenidea	Dolichogenidea jft03	Jose Leonardo Fernandez	jftriana@uoguelph.ca								Goulet & Boudrealt	16-Jul-2006		Canada	Yukon Territory			Pelly Crossing	62.8310013	-136.58501	495	
CNCHY358-07	HYM00000630	HYM00000630	HYM00000630	629				HYM00000630	Arthropoda	Insecta	Hymenoptera	Braconidae	Microgastrinae	Dolichogenidea	Dolichogenidea jft02	Jose Leonardo Fernandez	jftriana@uoguelph.ca								Goulet & Boudrealt	08-Jul-2006		Canada	Yukon Territory			Champagne	60.7900009	-136.437	733	
CNCHY368-07	HYM00000640	HYM00000640	HYM00000640	657				HYM00000640	Arthropoda	Insecta	Hymenoptera	Braconidae	Microgastrinae	Dolichogenidea	Dolichogenidea jft06	Jose Leonardo Fernandez	jftriana@uoguelph.ca								Goulet & Boudrealt	16-Jul-2006		Canada	Yukon Territory			Pelly Crossing	62.8310013	-136.58501	495	
CNCHY374-07	HYM00000646	HYM00000646	HYM00000646	618				HYM00000646	Arthropoda	Insecta	Hymenoptera	Braconidae	Microgastrinae	Dolichogenidea	Dolichogenidea jft06	Jose Leonardo Fernandez	jftriana@uoguelph.ca								Goulet & Boudrealt	16-Jul-2006		Canada	Yukon Territory			Pelly Crossing	62.8310013	-136.58501	495	
CNCHZ001-09	MIC 000284		MIC 000284	130	GU141636			MIC 000284	Arthropoda	Insecta	Hymenoptera	Braconidae	Microgastrinae	Dolichogenidea	Dolichogenidea jft03	Jose Leonardo Fernandez	jftriana@uoguelph.ca								H. K. Rutz	21-Jul-1963		Canada	Nunavut	Axel Heiberg Island		Axel Heiberg Island	79.25	-90.449997		
CNCHZ002-09	MIC 000285		MIC 000285	144	GU141713			MIC 000285	Arthropoda	Insecta	Hymenoptera	Braconidae	Microgastrinae	Illidops	Illidops jft02	Jose Leonardo Fernandez	jftriana@uoguelph.ca								J. E. Martin	25-Jul-1965		Canada	Northwest Territories	Melville Island		Bailey Point	75	-115		
CNCHZ003-09	MIC 000286		MIC 000286	144	GU141635			MIC 000286	Arthropoda	Insecta	Hymenoptera	Braconidae	Microgastrinae	Pholetesor	Pholetesor jft01	Jose Leonardo Fernandez	jftriana@uoguelph.ca								F. DiLabio	31-Jul-1948		Canada	Nunavut	Baffin Island		Frobisher Bay	63.4500008	-68.309998		
CNCHZ004-09	MIC 000287		MIC 000287	144	GU141712			MIC 000287	Arthropoda	Insecta	Hymenoptera	Braconidae	Microgastrinae	Illidops		Jose Leonardo Fernandez	jftriana@uoguelph.ca								Canadian Peary La	05-Aug-1966		Greenland		Greenland		Nedre Midsommer So,	82.1500015	-34.150002		
CNCHZ005-09	MIC 000288		MIC 000288	0		0		MIC 000288	Arthropoda	Insecta	Hymenoptera	Braconidae	Microgastrinae	Apanteles		Jose Leonardo Fernandez	jftriana@uoguelph.ca								J. Ryan	15-Aug-1970		Canada	Nunavut	Devon Island			75	-85		
CNCHZ006-09	MIC 000289		MIC 000289	144	GU141634			MIC 000289	Arthropoda	Insecta	Hymenoptera	Braconidae	Microgastrinae	Dolichogenidea	Dolichogenidea jft03	Jose Leonardo Fernandez	jftriana@uoguelph.ca								R. Madge	25-Jul-1962		Canada	Nunavut	Ellesmere Island		Hazen Camp	81.4899979	-71.18		
CNCHZ007-09	MIC 000290		MIC 000290	144	GU141633			MIC 000290	Arthropoda	Insecta	Hymenoptera	Braconidae	Microgastrinae	Dolichogenidea	Dolichogenidea jft11	Jose Leonardo Fernandez	jftriana@uoguelph.ca								W. Mason	29-Jul-1968		Canada	Northwest Territories	Banks Islands		Masik River	71.3700027	-123.07		
CNCHZ008-09	MIC 000291		MIC 000291	126	GU141711			MIC 000291	Arthropoda	Insecta	Hymenoptera	Braconidae	Microgastrinae	Illidops	Illidops jft02	Jose Leonardo Fernandez	jftriana@uoguelph.ca								G & M. Wood	13-Jul-1975		Canada	Northwest Territories	Victoria Island			71.1699982	-114		
CNCHZ009-09	MIC 000292		MIC 000292	144	GU141632			MIC 000292	Arthropoda	Insecta	Hymenoptera	Braconidae	Microgastrinae	Dolichogenidea	Dolichogenidea jft03	Jose Leonardo Fernandez	jftriana@uoguelph.ca								J. E. Martin	07-Aug-1958		Canada	Nunavut	Baffin Island		Head of Clyde Inlet	70.2900009	-68.309998		
CNCHZ010-09	MIC 000293		MIC 000293	0		0		MIC 000293	Arthropoda	Insecta	Hymenoptera	Braconidae	Microgastrinae	Glyptapanteles		Jose Leonardo Fernandez	jftriana@uoguelph.ca								G & M. Wood	03-Jul-1975		Canada	Northwest Territories	Victoria Island			71.1699982	-114		
CNCHZ011-09	MIC 000294		MIC 000294	144	GU141700			MIC 000294	Arthropoda	Insecta	Hymenoptera	Braconidae	Microgastrinae	Glyptapanteles	Glyptapanteles jft07	Jose Leonardo Fernandez	jftriana@uoguelph.ca								G. Shewell	03-Jul-1958		Canada	Nunavut	Baffin Island		Clyde Inlet	70.2900009	-68.309998		
CNCHZ012-09	MIC 000295		MIC 000295	134	GU141699			MIC 000295	Arthropoda	Insecta	Hymenoptera	Braconidae	Microgastrinae	Glyptapanteles		Jose Leonardo Fernandez	jftriana@uoguelph.ca								P. F. Bruggemann	19-Jun-1951		Canada	Nunavut	Ellesmere Island		Alert, R. E. # 3	82.3000031	-62.18		
CNCHZ013-09	MIC 000296		MIC 000296	129	GU141698			MIC 000296	Arthropoda	Insecta	Hymenoptera	Braconidae	Microgastrinae	Glyptapanteles	Glyptapanteles jft05	Jose Leonardo Fernandez	jftriana@uoguelph.ca								O. Kukal	27-Jul-1982		Canada	Nunavut	Ellesmere Island		Alexandra Fiord	78.5299988	-75.550003		
CNCHZ014-09	MIC 000297		MIC 000297	144	GU141697			MIC 000297	Arthropoda	Insecta	Hymenoptera	Braconidae	Microgastrinae	Glyptapanteles	Glyptapanteles jft07	Jose Leonardo Fernandez	jftriana@uoguelph.ca								G & M. Wood	13-Jul-1975		Canada	Northwest Territories	Victoria Island			71.1699982	-114		
CNCHZ015-09	MIC 000298		MIC 000298	144	GU141696			MIC 000298	Arthropoda	Insecta	Hymenoptera	Braconidae	Microgastrinae	Glyptapanteles	Glyptapanteles jft09	Jose Leonardo Fernandez	jftriana@uoguelph.ca								W. Mason	16-Jul-1968		Canada	Northwest Territories	Banks Islands		Masik River	71.3700027	-123.07		
CNCHZ016-09	MIC 000299		MIC 000299	0		0		MIC 000299	Arthropoda	Insecta	Hymenoptera	Braconidae	Microgastrinae	Glyptapanteles		Jose Leonardo Fernandez	jftriana@uoguelph.ca								H. Huckel	26-Aug-1954		Canada	Nunavut	Dorset Island		Cape Dorset	64.1399994	-76.32		
CNCHZ017-09	MIC 000300		MIC 000300	144	GU141695			MIC 000300	Arthropoda	Insecta	Hymenoptera	Braconidae	Microgastrinae	Glyptapanteles	Glyptapanteles jft05	Jose Leonardo Fernandez	jftriana@uoguelph.ca								W. Brown	07-Aug-1935		Canada	Nunavut	Baffin Island		Lake Harbour	62.5099983	-69.519997		
CNCHZ018-09	MIC 000301		MIC 000301	144	GU141694			MIC 000301	Arthropoda	Insecta	Hymenoptera	Braconidae	Microgastrinae	Glyptapanteles		Jose Leonardo Fernandez	jftriana@uoguelph.ca								H. K. Rutz	20-Jul-1963		Canada	Nunavut	Axel Heiberg Island		Axel Heiberg Island	79.25	-90.449997		
CNCHZ019-09	MIC 000302		MIC 000302	104	GU141693			MIC 000302	Arthropoda	Insecta	Hymenoptera	Braconidae	Microgastrinae	Glyptapanteles	Glyptapanteles jft07	Jose Leonardo Fernandez	jftriana@uoguelph.ca								G & M. Wood	03-Jul-1975		Canada	Northwest Territories	Victoria Island			71.1699982	-114		
CNCHZ020-09	MIC 000303		MIC 000303	144	GU141692			MIC 000303	Arthropoda	Insecta	Hymenoptera	Braconidae	Microgastrinae	Glyptapanteles	Glyptapanteles jft05	Jose Leonardo Fernandez	jftriana@uoguelph.ca								F. DiLabio	31-Jul-1948		Canada	Nunavut	Baffin Island		Frobisher Bay	63.4500008	-68.309998		
CNCHZ021-09	MIC 000304		MIC 000304	144	GU141691			MIC 000304	Arthropoda	Insecta	Hymenoptera	Braconidae	Microgastrinae	Glyptapanteles	Glyptapanteles jft09	Jose Leonardo Fernandez	jftriana@uoguelph.ca								G. Shewell	07-Aug-1958		Canada	Nunavut	Baffin Island		Head of Clyde Inlet	70.2900009	-68.309998		
CNCHZ022-09	MIC 000305		MIC 000305	144	GU141690			MIC 000305	Arthropoda	Insecta	Hymenoptera	Braconidae	Microgastrinae	Glyptapanteles		Jose Leonardo Fernandez	jftriana@uoguelph.ca								P. F. Bruggemann	19-Jun-1951		Canada	Nunavut	Ellesmere Island		Alert, R. E. # 3	82.3000031	-62.18		
CNCHZ023-09	MIC 000306		MIC 000306	144	GU141689			MIC 000306	Arthropoda	Insecta	Hymenoptera	Braconidae	Microgastrinae	Glyptapanteles	Glyptapanteles jft05	Jose Leonardo Fernandez	jftriana@uoguelph.ca								W. Mason	30-Jul-1968		Canada	Northwest Territories	Banks Islands		Masik River	71.3700027	-123.07		
CNCHZ024-09	MIC 000307	MIC 000307	MIC 000307	144	GU141688			MIC 000307	Arthropoda	Insecta	Hymenoptera	Braconidae	Microgastrinae	Apanteles	Apanteles jft39	Jose Leonardo Fernandez	jftriana@uoguelph.ca						NULL		G. Shewell	06-Aug-1966		Canada	Nunavut	Bathurst Inlet		Baychimo Harb.	67.4199982	-107.57		
CNCHZ025-09	MIC 000308		MIC 000308	0		0		MIC 000308	Arthropoda	Insecta	Hymenoptera	Braconidae	Microgastrinae	Glyptapanteles		Jose Leonardo Fernandez	jftriana@uoguelph.ca								J. Chillcot	13-Aug-1951		Canada	Northwest Territories			Spence Bay	69.3199997	-93.32		
CNCHZ026-09	MIC 000309		MIC 000309	124	GU141687			MIC 000309	Arthropoda	Insecta	Hymenoptera	Braconidae	Microgastrinae	Glyptapanteles	Glyptapanteles jft05	Jose Leonardo Fernandez	jftriana@uoguelph.ca								Canadian Peary La	03-Aug-1966		Greenland		Greenland		Nedre Midsommer So,	82.1500015	-34.150002		
CNCHZ027-09	MIC 000310		MIC 000310	144	GU141686			MIC 000310	Arthropoda	Insecta	Hymenoptera	Braconidae	Microgastrinae	Glyptapanteles	Glyptapanteles jft05	Jose Leonardo Fernandez	jftriana@uoguelph.ca								H. K. Rutz	14-Aug-1963		Canada	Nunavut	Axel Heiberg Island		Axel Heiberg Island	79.25	-90.449997		
CNCHZ028-09	MIC 000311	MIC 000311	MIC 000311	144	GU141730			MIC 000311	Arthropoda	Insecta	Hymenoptera	Braconidae	Microgastrinae	Microgaster	Microgaster jft08	Jose Leonardo Fernandez	jftriana@uoguelph.ca		S	A			NULL		W. Mason	08-Jul-1968		Canada	Northwest Territories	Banks Islands		Masik River	71.3700027	-123.07		
CNCHZ029-09	MIC 000312	MIC 000312	MIC 000312	144	GU141793			MIC 000312	Arthropoda	Insecta	Hymenoptera	Braconidae	Microgastrinae	Microplitis	Microplitis jft01	Jose Leonardo Fernandez	jftriana@uoguelph.ca		S	A			NULL		J. Smith	11-Jul-1980		Canada	Nunavut	Ellesmere Island			79.2900009	-75.5		
CNCHZ030-09	MIC 000313		MIC 000313	144	GU141792			MIC 000313	Arthropoda	Insecta	Hymenoptera	Braconidae	Microgastrinae	Microplitis	Microplitis jft32	Jose Leonardo Fernandez	jftriana@uoguelph.ca		S	A					O. Kukal	27-Jul-1982		Canada	Nunavut	Ellesmere Island		Alexandra Fiord	78.5299988	-75.550003		
CNCHZ031-09	MIC 000314	MIC 000314	MIC 000314	129	GU141791			MIC 000314	Arthropoda	Insecta	Hymenoptera	Braconidae	Microgastrinae	Microplitis	Microplitis jft01	Jose Leonardo Fernandez	jftriana@uoguelph.ca		S	A			NULL		Canadian Peary La	19-Jul-1966		Greenland		Greenland		Nedre Midsommer So	82.1500015	-34.150002		
CNCHZ032-09	MIC 000315		MIC 000315	114	GU141790			MIC 000315	Arthropoda	Insecta	Hymenoptera	Braconidae	Microgastrinae	Microplitis	Microplitis jft41	Jose Leonardo Fernandez	jftriana@uoguelph.ca		S	A					J. Ryan & C. Herge	15-Jul-1971		Canada	Nunavut	Devon Island			75	-85		
CNCHZ033-09	MIC 000316		MIC 000316	0		0		MIC 000316	Arthropoda	Insecta	Hymenoptera	Braconidae	Microgastrinae	Microplitis		Jose Leonardo Fernandez	jftriana@uoguelph.ca								R. Leech	08-Aug-1963		Canada	Nunavut	Ellesmere Island		Hazen Camp	81.4899979	-71.18		
CNCHZ034-09	MIC 000317	MIC 000317	MIC 000317	144	GU141599			MIC 000317	Arthropoda	Insecta	Hymenoptera	Braconidae	Microgastrinae	Cotesia	Cotesia jft08	Jose Leonardo Fernandez	jftriana@uoguelph.ca		S	A			NULL		P. F. Bruggemann	06-Jul-1953		Canada	Nunavut	Ellesmere Island		Eureka	79.5899963	-85.790001		
CNCHZ035-09	MIC 000318		MIC 000318	144	GU141593			MIC 000318	Arthropoda	Insecta	Hymenoptera	Braconidae	Microgastrinae	Cotesia	Cotesia jft22	Jose Leonardo Fernandez	jftriana@uoguelph.ca		S	A					W. Mason	28-Jul-1968		Canada	Northwest Territories	Banks Islands		Masik River	71.3700027	-123.07		
CNCHZ036-09	MIC 000319	MIC 000319	MIC 000319	144	GU141598			MIC 000319	Arthropoda	Insecta	Hymenoptera	Braconidae	Microgastrinae	Cotesia	Cotesia jft08	Jose Leonardo Fernandez	jftriana@uoguelph.ca		S	A			NULL		O. Kukal	15-Jul-1982		Canada	Nunavut	Ellesmere Island		Alexandra Fiord	78.5299988	-75.550003		
CNCHZ037-09	MIC 000320	MIC 000320	MIC 000320	144	GU141597			MIC 000320	Arthropoda	Insecta	Hymenoptera	Braconidae	Microgastrinae	Cotesia	Cotesia jft08	Jose Leonardo Fernandez	jftriana@uoguelph.ca		S	A			NULL		P. F. Bruggemann	06-Jul-1953		Canada	Nunavut	Ellesmere Island		Eureka	79.5899963	-85.790001		
CNCHZ038-09	MIC 000321	MIC 000321	MIC 000321	144	GU141596			MIC 000321	Arthropoda	Insecta	Hymenoptera	Braconidae	Microgastrinae	Cotesia	Cotesia jft08	Jose Leonardo Fernandez	jftriana@uoguelph.ca		S	A			NULL		O. Kukal	06-Jul-1982		Canada	Nunavut	Ellesmere Island		Alexandra Fiord	78.5299988	-75.550003		
CNCHZ039-09	MIC 000322		MIC 000322	144	GU141592			MIC 000322	Arthropoda	Insecta	Hymenoptera	Braconidae	Microgastrinae	Cotesia	Cotesia jft09	Jose Leonardo Fernandez	jftriana@uoguelph.ca		S	A					J. Smith	11-Jul-1980		Canada	Nunavut	Ellesmere Island			79.2900009	-75.5		
CNCHZ040-09	MIC 000323		MIC 000323	0		0		MIC 000323	Arthropoda	Insecta	Hymenoptera	Braconidae	Microgastrinae	Glyptapanteles		Jose Leonardo Fernandez	jftriana@uoguelph.ca								F. Brodo	07-Aug-1991		Canada	Nunavut	Ellesmere Island		I. Fosheim Pen., Hot W	79.5800018	-84.279999		
CNCHZ041-09	MIC 000324		MIC 000324	144	GU141591			MIC 000324	Arthropoda	Insecta	Hymenoptera	Braconidae	Microgastrinae	Cotesia	Cotesia jft23	Jose Leonardo Fernandez	jftriana@uoguelph.ca		S	A					J. E. Martin	28-Jun-1950		Canada	Nunavut			Repulse Bay	66.3099976	-86.129997		
CNCHZ042-09	MIC 000325		MIC 000325	144	GU141590			MIC 000325	Arthropoda	Insecta	Hymenoptera	Braconidae	Microgastrinae	Cotesia	Cotesia jft09	Jose Leonardo Fernandez	jftriana@uoguelph.ca		S	A					F. DiLabio	31-Jul-1948		Canada	Nunavut	Baffin Island		Frobisher Bay	63.4500008	-68.309998		
CNCHZ043-09	MIC 000326		MIC 000326	144	GU141589			MIC 000326	Arthropoda	Insecta	Hymenoptera	Braconidae	Microgastrinae	Cotesia		Jose Leonardo Fernandez	jftriana@uoguelph.ca								Canadian Peary La	01-Jul-1966		Greenland		Greenland		Nedre Midsommer So	82.1500015	-34.150002		
CNCHZ044-09	MIC 000327		MIC 000327	113	GU141588			MIC 000327	Arthropoda	Insecta	Hymenoptera	Braconidae	Microgastrinae	Cotesia	Cotesia jft08	Jose Leonardo Fernandez	jftriana@uoguelph.ca		S	A					D. R. Oliver	12-Jul-1963		Canada	Nunavut	Ellesmere Island		Hazen Camp	81.4899979	-71.18		
CNCHZ045-09	MIC 000328		MIC 000328	144	GU141587			MIC 000328	Arthropoda	Insecta	Hymenoptera	Braconidae	Microgastrinae	Cotesia	Cotesia jft08	Jose Leonardo Fernandez	jftriana@uoguelph.ca		S	A					R. Leech	15-Jul-1963		Canada	Nunavut	Ellesmere Island		Hazen Camp	81.4899979	-71.18		
CNCHZ046-09	MIC 000329	MIC 000329	MIC 000329	144	GU141595			MIC 000329	Arthropoda	Insecta	Hymenoptera	Braconidae	Microgastrinae	Cotesia	Cotesia jft08	Jose Leonardo Fernandez	jftriana@uoguelph.ca		S	A			NULL			07-Jul-1970		Canada	Nunavut	Devon Island			75	-85		
CNCHZ047-09	MIC 000330		MIC 000330	144	GU141586			MIC 000330	Arthropoda	Insecta	Hymenoptera	Braconidae	Microgastrinae	Cotesia	Cotesia jft52	Jose Leonardo Fernandez	jftriana@uoguelph.ca		S	A					W. Mason	29-Jul-1968		Canada	Northwest Territories	Banks Islands		Masik River	71.3700027	-123.07		
CNCHZ048-09	MIC 000331		MIC 000331	144	GU141585			MIC 000331	Arthropoda	Insecta	Hymenoptera	Braconidae	Microgastrinae	Cotesia	Cotesia halli	Jose Leonardo Fernandez	jftriana@uoguelph.ca		S	A					H. K. Rutz	19-Jul-1963		Canada	Nunavut	Axel Heiberg Island		Axel Heiberg Island	79.25	-90.449997		
CNCHZ049-09	MIC 000332		MIC 000332	0		0		MIC 000332	Arthropoda	Insecta	Hymenoptera	Braconidae	Microgastrinae	Glyptapanteles		Jose Leonardo Fernandez	jftriana@uoguelph.ca								J. Chillcot	10-Jul-1953		Canada	Nunavut			Muskox Lake	64.4499969	-108.1		
CNCHZ050-09	MIC 000333		MIC 000333	144	GU141685			MIC 000333	Arthropoda	Insecta	Hymenoptera	Braconidae	Microgastrinae	Glyptapanteles	Glyptapanteles jft07	Jose Leonardo Fernandez	jftriana@uoguelph.ca								E. Smith	03-Aug-1950		Canada	Nunavut			Cambridge Bay	69.0699997	-105.03		
CNCHZ051-09	MIC 000334		MIC 000334	144	GU141684			MIC 000334	Arthropoda	Insecta	Hymenoptera	Braconidae	Microgastrinae	Glyptapanteles	Glyptapanteles jft01	Jose Leonardo Fernandez	jftriana@uoguelph.ca								W. Mason	09-Jul-1971		Canada	Northwest Territories			32 Km E of Tuktoyaktu	69.3000031	-132.14999		
CNCHZ052-09	MIC 000335		MIC 000335	0		0		MIC 000335	Arthropoda	Insecta	Hymenoptera	Braconidae	Microgastrinae	Glyptapanteles		Jose Leonardo Fernandez	jftriana@uoguelph.ca								W. Mason	25-Jul-1971		Canada	Northwest Territories			32 Km E of Tuktoyaktu	69.3000031	-132.14999		
CNCHZ053-09	MIC 000336		MIC 000336	144	GU141683			MIC 000336	Arthropoda	Insecta	Hymenoptera	Braconidae	Microgastrinae	Glyptapanteles	Glyptapanteles jft01	Jose Leonardo Fernandez	jftriana@uoguelph.ca								L. Humble	30-Jul-1979		Canada	Northwest Territories			32 Km NE of Tuktoyakt	69.3000031	-132.14999		
CNCHZ054-09	MIC 000337		MIC 000337	144	GU141682			MIC 000337	Arthropoda	Insecta	Hymenoptera	Braconidae	Microgastrinae	Glyptapanteles	Glyptapanteles jft01	Jose Leonardo Fernandez	jftriana@uoguelph.ca								W. Mason	22-Jun-1971		Canada	Northwest Territories			Kovaluk River	69.1100006	-131		
CNCHZ055-09	MIC 000338		MIC 000338	144	GU141681			MIC 000338	Arthropoda	Insecta	Hymenoptera	Braconidae	Microgastrinae	Glyptapanteles	Glyptapanteles jft01	Jose Leonardo Fernandez	jftriana@uoguelph.ca								J. Chillcot	04-Jul-1953		Canada	Northwest Territories			Salmita Mines	64.0500031	-111.15		
CNCHZ056-09	MIC 000339	MIC 000339	MIC 000339	144	GU141631			MIC 000339	Arthropoda	Insecta	Hymenoptera	Braconidae	Microgastrinae	Pholetesor	Pholetesor jft11	Jose Leonardo Fernandez	jftriana@uoguelph.ca						NULL		G. Shewell	17-Jul-1966		Canada	Nunavut			Nr. Beechey Lake	65.1399994	-106.5		
CNCHZ057-09	MIC 000340		MIC 000340	144	GU141680			MIC 000340	Arthropoda	Insecta	Hymenoptera	Braconidae	Microgastrinae	Glyptapanteles		Jose Leonardo Fernandez	jftriana@uoguelph.ca								W. Mason	09-Jul-1949		Canada	Northwest Territories			Norman Wells	65.1699982	-126.51		
CNCHZ058-09	MIC 000341		MIC 000341	144	GU141679			MIC 000341	Arthropoda	Insecta	Hymenoptera	Braconidae	Microgastrinae	Glyptapanteles	Glyptapanteles jft07	Jose Leonardo Fernandez	jftriana@uoguelph.ca								G. Shewell	02-Jul-1966		Canada	Northwest Territories			Ford Lake	63.1100006	-107.19		
CNCHZ059-09	MIC 000342		MIC 000342	144	GU141584			MIC 000342	Arthropoda	Insecta	Hymenoptera	Braconidae	Microgastrinae	Cotesia	Cotesia xylina	Jose Leonardo Fernandez	jftriana@uoguelph.ca		S	A					W. Mason	10-Jul-1949		Canada	Northwest Territories			Norman Wells	65.1699982	-126.51		
CNCHZ060-09	MIC 000343		MIC 000343	0		0		MIC 000343	Arthropoda	Insecta	Hymenoptera	Braconidae	Microgastrinae	Apanteles		Jose Leonardo Fernandez	jftriana@uoguelph.ca								G. Walley	14-Jul-1934		Canada	Ontario			Moosonee	51.1599998	-80.389999		
CNCHZ061-09	MIC 000344		MIC 000344	0		0		MIC 000344	Arthropoda	Insecta	Hymenoptera	Braconidae	Microgastrinae	Illidops		Jose Leonardo Fernandez	jftriana@uoguelph.ca								W. Mason	19-Jul-1958		Canada	Quebec			Payne Bay	59.5499992	-69.339996		
CNCHZ062-09	MIC 000345		MIC 000345	144	GU141710			MIC 000345	Arthropoda	Insecta	Hymenoptera	Braconidae	Microgastrinae	Illidops	Illidops jft02	Jose Leonardo Fernandez	jftriana@uoguelph.ca								J. Chillcot	08-Aug-1950		Canada	Northwest Territories			Chesterfield	63.2000008	-90.419998		
CNCHZ063-09	MIC 000346		MIC 000346	144	GU141709			MIC 000346	Arthropoda	Insecta	Hymenoptera	Braconidae	Microgastrinae	Illidops		Jose Leonardo Fernandez	jftriana@uoguelph.ca								W. Mason	18-Jul-1971		Canada	Northwest Territories			32 Km E of Tuktoyaktu	69.3000031	-132.14999		

Table S5: All collection and accession information associated with specimens analysed here.

ProcessID	Sample ID	Field Num	Catalog Num	COI-5P Seq.	COI GenBank	28S Seq. Length	28S GenBank	Sample ID	Phylum	Class	Order	Family	Subfamily	Genus	Species	Identifier	Identifier email
CNCHZ064-09	MIC 000347		MIC 000347	120	GU141708			MIC 000347	Arthropoda	Insecta	Hymenoptera	Braconidae	Microgastrinae	Illidops	Illidops jft02	Jose Leonardo Fernandez	jftriana@uoguelph.ca
CNCHZ065-09	MIC 000348		MIC 000348	144	GU141613			MIC 000348	Arthropoda	Insecta	Hymenoptera	Braconidae	Microgastrinae	Diolcogaster		Jose Leonardo Fernandez	jftriana@uoguelph.ca
CNCHZ066-09	MIC 000349		MIC 000349	144	GU141612			MIC 000349	Arthropoda	Insecta	Hymenoptera	Braconidae	Microgastrinae	Diolcogaster		Jose Leonardo Fernandez	jftriana@uoguelph.ca
CNCHZ067-09	MIC 000350		MIC 000350	144	GU141630			MIC 000350	Arthropoda	Insecta	Hymenoptera	Braconidae	Microgastrinae	Pholetesor	Pholetesor viminetorum	Jose Leonardo Fernandez	jftriana@uoguelph.ca
CNCHZ068-09	MIC 000351		MIC 000351	144	GU141707			MIC 000351	Arthropoda	Insecta	Hymenoptera	Braconidae	Microgastrinae	Illidops	Illidops jft02	Jose Leonardo Fernandez	jftriana@uoguelph.ca
CNCHZ069-09	MIC 000352		MIC 000352	0		0		MIC 000352	Arthropoda	Insecta	Hymenoptera	Braconidae	Microgastrinae	Cotesia		Jose Leonardo Fernandez	jftriana@uoguelph.ca
CNCHZ070-09	MIC 000353		MIC 000353	144	GU141583			MIC 000353	Arthropoda	Insecta	Hymenoptera	Braconidae	Microgastrinae	Cotesia	Cotesia jft23	Jose Leonardo Fernandez	jftriana@uoguelph.ca
CNCHZ071-09	MIC 000354		MIC 000354	144	GU141582			MIC 000354	Arthropoda	Insecta	Hymenoptera	Braconidae	Microgastrinae	Cotesia	Cotesia jft23	Jose Leonardo Fernandez	jftriana@uoguelph.ca
CNCHZ072-09	MIC 000355		MIC 000355	144	GU141571			MIC 000355	Arthropoda	Insecta	Hymenoptera	Braconidae	Microgastrinae	Dolichogenidea	Dolichogenidea jft16	Jose Leonardo Fernandez	jftriana@uoguelph.ca
CNCHZ073-09	MIC 000356		MIC 000356	0		0		MIC 000356	Arthropoda	Insecta	Hymenoptera	Braconidae	Microgastrinae	Apanteles		Jose Leonardo Fernandez	jftriana@uoguelph.ca
CNCHZ074-09	MIC 000357		MIC 000357	144	GU141629			MIC 000357	Arthropoda	Insecta	Hymenoptera	Braconidae	Microgastrinae	Dolichogenidea	Dolichogenidea jft03	Jose Leonardo Fernandez	jftriana@uoguelph.ca
CNCHZ075-09	MIC 000358		MIC 000358	144	GU141628			MIC 000358	Arthropoda	Insecta	Hymenoptera	Braconidae	Microgastrinae	Dolichogenidea	Dolichogenidea jft04	Jose Leonardo Fernandez	jftriana@uoguelph.ca
CNCHZ076-09	MIC 000359		MIC 000359	144	GU141627			MIC 000359	Arthropoda	Insecta	Hymenoptera	Braconidae	Microgastrinae	Dolichogenidea	Dolichogenidea jft02	Jose Leonardo Fernandez	jftriana@uoguelph.ca
CNCHZ077-09	MIC 000360		MIC 000360	144	GU141570			MIC 000360	Arthropoda	Insecta	Hymenoptera	Braconidae	Microgastrinae	Apanteles	Apanteles jft11	Jose Leonardo Fernandez	jftriana@uoguelph.ca
CNCHZ078-09	MIC 000361		MIC 000361	144	GU141569			MIC 000361	Arthropoda	Insecta	Hymenoptera	Braconidae	Microgastrinae	Apanteles	Apanteles jft11	Jose Leonardo Fernandez	jftriana@uoguelph.ca
CNCHZ079-09	MIC 000362		MIC 000362	144	GU141789			MIC 000362	Arthropoda	Insecta	Hymenoptera	Braconidae	Microgastrinae	Microplitis	Microplitis jft08	Jose Leonardo Fernandez	jftriana@uoguelph.ca
CNCHZ080-09	MIC 000363		MIC 000363	127	GU141788			MIC 000363	Arthropoda	Insecta	Hymenoptera	Braconidae	Microgastrinae	Microplitis	Microplitis jft45	Jose Leonardo Fernandez	jftriana@uoguelph.ca
CNCHZ081-09	MIC 000364	MIC 000364	MIC 000364	126	GU141787			MIC 000364	Arthropoda	Insecta	Hymenoptera	Braconidae	Microgastrinae	Microplitis	Microplitis jft01	Jose Leonardo Fernandez	jftriana@uoguelph.ca
CNCHZ082-09	MIC 000365		MIC 000365	0		0		MIC 000365	Arthropoda	Insecta	Hymenoptera	Braconidae	Microgastrinae	Microplitis		Jose Leonardo Fernandez	jftriana@uoguelph.ca
CNCHZ083-09	MIC 000366	MIC 000366	MIC 000366	144	GU141786			MIC 000366	Arthropoda	Insecta	Hymenoptera	Braconidae	Microgastrinae	Microplitis	Microplitis jft12	Jose Leonardo Fernandez	jftriana@uoguelph.ca
CNCHZ084-09	MIC 000367		MIC 000367	144	GU141785			MIC 000367	Arthropoda	Insecta	Hymenoptera	Braconidae	Microgastrinae	Microplitis	Microplitis jft30	Jose Leonardo Fernandez	jftriana@uoguelph.ca
CNCHZ085-09	MIC 000368		MIC 000368	0		0		MIC 000368	Arthropoda	Insecta	Hymenoptera	Braconidae	Microgastrinae	Apanteles		Jose Leonardo Fernandez	jftriana@uoguelph.ca
CNCHZ086-09	MIC 000369		MIC 000369	144	GU141626			MIC 000369	Arthropoda	Insecta	Hymenoptera	Braconidae	Microgastrinae	Dolichogenidea	Dolichogenidea jft03	Jose Leonardo Fernandez	jftriana@uoguelph.ca
CNCHZ087-09	MIC 000370		MIC 000370	118	GU141568			MIC 000370	Arthropoda	Insecta	Hymenoptera	Braconidae	Microgastrinae	Apanteles	Apanteles fumiferanae	Jose Leonardo Fernandez	jftriana@uoguelph.ca
CNCHZ088-09	MIC 000371		MIC 000371	0		0		MIC 000371	Arthropoda	Insecta	Hymenoptera	Braconidae	Microgastrinae	Apanteles		Jose Leonardo Fernandez	jftriana@uoguelph.ca
CNCHZ089-09	MIC 000372		MIC 000372	144	GU141784			MIC 000372	Arthropoda	Insecta	Hymenoptera	Braconidae	Microgastrinae	Microplitis	Microplitis jft41	Jose Leonardo Fernandez	jftriana@uoguelph.ca
CNCHZ090-09	MIC 000373	MIC 000373	MIC 000373	144	GU141783			MIC 000373	Arthropoda	Insecta	Hymenoptera	Braconidae	Microgastrinae	Microplitis	Microplitis jft01	Jose Leonardo Fernandez	jftriana@uoguelph.ca
CNCHZ091-09	MIC 000374		MIC 000374	144	GU141678			MIC 000374	Arthropoda	Insecta	Hymenoptera	Braconidae	Microgastrinae	Glyptapanteles	Glyptapanteles jft07	Jose Leonardo Fernandez	jftriana@uoguelph.ca
CNCHZ092-09	MIC 000375		MIC 000375	0		0		MIC 000375	Arthropoda	Insecta	Hymenoptera	Braconidae	Microgastrinae	Apanteles		Jose Leonardo Fernandez	jftriana@uoguelph.ca
CNCHZ093-09	MIC 000376		MIC 000376	144	GU141815			MIC 000376	Arthropoda	Insecta	Hymenoptera	Braconidae	Microgastrinae	Pholetesor	Pholetesor ornigis	Jose Leonardo Fernandez	jftriana@uoguelph.ca
CNCHZ094-09	MIC 000377		MIC 000377	144	GU141677			MIC 000377	Arthropoda	Insecta	Hymenoptera	Braconidae	Microgastrinae	Glyptapanteles	Glyptapanteles jft05	Jose Leonardo Fernandez	jftriana@uoguelph.ca
CNCHZ095-09	MIC 000378		MIC 000378	144	GU141829			MIC 000378	Arthropoda	Insecta	Hymenoptera	Braconidae	Microgastrinae	Sathon		Jose Leonardo Fernandez	jftriana@uoguelph.ca
CNCHZ096-09	MIC 000379		MIC 000379	144	GU141782			MIC 000379	Arthropoda	Insecta	Hymenoptera	Braconidae	Microgastrinae	Microplitis	Microplitis jft40	Jose Leonardo Fernandez	jftriana@uoguelph.ca
CNCHZ097-09	MIC 000380		MIC 000380	144	GU141781			MIC 000380	Arthropoda	Insecta	Hymenoptera	Braconidae	Microgastrinae	Microplitis	Microplitis jft11	Jose Leonardo Fernandez	jftriana@uoguelph.ca
CNCHZ098-09	MIC 000381	MIC 000381	MIC 000381	144	GU141780			MIC 000381	Arthropoda	Insecta	Hymenoptera	Braconidae	Microgastrinae	Microplitis	Microplitis jft04	Jose Leonardo Fernandez	jftriana@uoguelph.ca
CNCHZ099-09	MIC 000382	MIC 000382	MIC 000382	129	GU141779			MIC 000382	Arthropoda	Insecta	Hymenoptera	Braconidae	Microgastrinae	Microplitis	Microplitis jft04	Jose Leonardo Fernandez	jftriana@uoguelph.ca
CNCHZ100-09	MIC 000383		MIC 000383	0		0		MIC 000383	Arthropoda	Insecta	Hymenoptera	Braconidae	Microgastrinae	Microplitis		Jose Leonardo Fernandez	jftriana@uoguelph.ca
CNCHZ101-09	MIC 000384		MIC 000384	0		0		MIC 000384	Arthropoda	Insecta	Hymenoptera	Braconidae	Microgastrinae	Microplitis		Jose Leonardo Fernandez	jftriana@uoguelph.ca
CNCHZ102-09	MIC 000385	MIC 000385	MIC 000385	126	GU141778			MIC 000385	Arthropoda	Insecta	Hymenoptera	Braconidae	Microgastrinae	Microplitis	Microplitis varicolor	Jose Leonardo Fernandez	jftriana@uoguelph.ca
CNCHZ103-09	MIC 000386		MIC 000386	144	GU141777			MIC 000386	Arthropoda	Insecta	Hymenoptera	Braconidae	Microgastrinae	Microplitis	Microplitis jft08	Jose Leonardo Fernandez	jftriana@uoguelph.ca
CNCHZ104-09	MIC 000387		MIC 000387	0		0		MIC 000387	Arthropoda	Insecta	Hymenoptera	Braconidae	Microgastrinae	Glyptapanteles		Jose Leonardo Fernandez	jftriana@uoguelph.ca
CNCHZ105-09	MIC 000388		MIC 000388	0		0		MIC 000388	Arthropoda	Insecta	Hymenoptera	Braconidae	Microgastrinae	Microplitis		Jose Leonardo Fernandez	jftriana@uoguelph.ca
CNCHZ106-09	MIC 000389	MIC 000389	MIC 000389	144	GU141729			MIC 000389	Arthropoda	Insecta	Hymenoptera	Braconidae	Microgastrinae	Microgaster	Microgaster jft13	Jose Leonardo Fernandez	jftriana@uoguelph.ca
CNCHZ107-09	MIC 000390		MIC 000390	129	GU141828			MIC 000390	Arthropoda	Insecta	Hymenoptera	Braconidae	Microgastrinae	Sathon		Jose Leonardo Fernandez	jftriana@uoguelph.ca
CNCHZ108-09	MIC 000391		MIC 000391	144	GU141611			MIC 000391	Arthropoda	Insecta	Hymenoptera	Braconidae	Microgastrinae	Diolcogaster		Jose Leonardo Fernandez	jftriana@uoguelph.ca
CNCHZ109-09	MIC 000392		MIC 000392	0		0		MIC 000392	Arthropoda	Insecta	Hymenoptera	Braconidae	Microgastrinae			Jose Leonardo Fernandez	jftriana@uoguelph.ca
CNCHZ110-09	MIC 000393		MIC 000393	144	GU141581			MIC 000393	Arthropoda	Insecta	Hymenoptera	Braconidae	Microgastrinae	Cotesia	Cotesia jft29	Jose Leonardo Fernandez	jftriana@uoguelph.ca
CNCHZ111-09	MIC 000394		MIC 000394	120	GU141580			MIC 000394	Arthropoda	Insecta	Hymenoptera	Braconidae	Microgastrinae	Cotesia	Cotesia jft24	Jose Leonardo Fernandez	jftriana@uoguelph.ca
CNCHZ112-09	MIC 000395		MIC 000395	134	GU141579			MIC 000395	Arthropoda	Insecta	Hymenoptera	Braconidae	Microgastrinae	Cotesia	Cotesia jft08	Jose Leonardo Fernandez	jftriana@uoguelph.ca
CNCHZ113-09	MIC 000396	MIC 000396	MIC 000396	125	GU141776			MIC 000396	Arthropoda	Insecta	Hymenoptera	Braconidae	Microgastrinae	Microplitis	Microplitis varicolor	Jose Leonardo Fernandez	jftriana@uoguelph.ca
CNCHZ114-09	MIC 000397		MIC 000397	0		0		MIC 000397	Arthropoda	Insecta	Hymenoptera	Braconidae	Microgastrinae	Dolichogenidea		Jose Leonardo Fernandez	jftriana@uoguelph.ca
CNCHZ1141-09	CAM0521	CAM0521	CAM0521	657				CAM0521	Arthropoda	Insecta	Hymenoptera	Braconidae	Microgastrinae	Apanteles	Apanteles ensiger	Jose Leonardo Fernandez	jftriana@uoguelph.ca
CNCHZ1142-09	CAM0522	CAM0522	CAM0522	657				CAM0522	Arthropoda	Insecta	Hymenoptera	Braconidae	Microgastrinae	Apanteles	Apanteles ensiger	Jose Leonardo Fernandez	jftriana@uoguelph.ca
CNCHZ1143-09	CAM0523	CAM0523	CAM0523	657				CAM0523	Arthropoda	Insecta	Hymenoptera	Braconidae	Microgastrinae	Apanteles	Apanteles ensiger	Jose Leonardo Fernandez	jftriana@uoguelph.ca
CNCHZ1144-09	CAM0524	CAM0524	CAM0524	632				CAM0524	Arthropoda	Insecta	Hymenoptera	Braconidae	Microgastrinae	Apanteles	Apanteles ensiger	Jose Leonardo Fernandez	jftriana@uoguelph.ca
CNCHZ1145-09	CAM0525	CAM0525	CAM0525	657				CAM0525	Arthropoda	Insecta	Hymenoptera	Braconidae	Microgastrinae	Apanteles	Apanteles ensiger	Jose Leonardo Fernandez	jftriana@uoguelph.ca
CNCHZ1146-09	CAM0526	CAM0526	CAM0526	657				CAM0526	Arthropoda	Insecta	Hymenoptera	Braconidae	Microgastrinae	Apanteles	Apanteles ensiger	Jose Leonardo Fernandez	jftriana@uoguelph.ca
CNCHZ1147-09	CAM0527	CAM0527	CAM0527	621				CAM0527	Arthropoda	Insecta	Hymenoptera	Braconidae	Microgastrinae	Apanteles	Apanteles ensiger	Jose Leonardo Fernandez	jftriana@uoguelph.ca
CNCHZ1148-09	CAM0528	CAM0528	CAM0528	657				CAM0528	Arthropoda	Insecta	Hymenoptera	Braconidae	Microgastrinae	Apanteles	Apanteles ensiger	Jose Leonardo Fernandez	jftriana@uoguelph.ca
CNCHZ1149-09	CAM0529	CAM0529	CAM0529	657				CAM0529	Arthropoda	Insecta	Hymenoptera	Braconidae	Microgastrinae	Apanteles	Apanteles ensiger	Jose Leonardo Fernandez	jftriana@uoguelph.ca
CNCHZ1150-09	CAM0530	CAM0530	CAM0530	657				CAM0530	Arthropoda	Insecta	Hymenoptera	Braconidae	Microgastrinae	Apanteles	Apanteles ensiger	Jose Leonardo Fernandez	jftriana@uoguelph.ca
CNCHZ115-09	MIC 000398		MIC 000398	144	GU141625			MIC 000398	Arthropoda	Insecta	Hymenoptera	Braconidae	Microgastrinae	Dolichogenidea	Dolichogenidea jft02	Jose Leonardo Fernandez	jftriana@uoguelph.ca
CNCHZ1151-09	CAM0531	CAM0531	CAM0531	657				CAM0531	Arthropoda	Insecta	Hymenoptera	Braconidae	Microgastrinae	Apanteles	Apanteles ensiger	Jose Leonardo Fernandez	jftriana@uoguelph.ca
CNCHZ1154-09	CAM0534	CAM0534	CAM0534	657				CAM0534	Arthropoda	Insecta	Hymenoptera	Braconidae	Microgastrinae	Dolichogenidea	Dolichogenidea jft02	Jose Leonardo Fernandez	jftriana@uoguelph.ca
CNCHZ1158-09	CAM0538	CAM0538	CAM0538	657				CAM0538	Arthropoda	Insecta	Hymenoptera	Braconidae	Microgastrinae	Cotesia	Cotesia jft02	Jose Leonardo Fernandez	jftriana@uoguelph.ca
CNCHZ116-09	MIC 000399		MIC 000399	120	GU141624			MIC 000399	Arthropoda	Insecta	Hymenoptera	Braconidae	Microgastrinae	Dolichogenidea	Dolichogenidea jft02	Jose Leonardo Fernandez	jftriana@uoguelph.ca
CNCHZ117-09	MIC 000400	MIC 000400	MIC 000400	130	GU141775			MIC 000400	Arthropoda	Insecta	Hymenoptera	Braconidae	Microgastrinae	Microplitis	Microplitis varicolor	Jose Leonardo Fernandez	jftriana@uoguelph.ca
CNCHZ1171-09	CAM0551	CAM0551	CAM0551	657				CAM0551	Arthropoda	Insecta	Hymenoptera	Braconidae	Microgastrinae	Microplitis	Microplitis jft07	Jose Leonardo Fernandez	jftriana@uoguelph.ca
CNCHZ1172-09	CAM0552	CAM0552	CAM0552	657				CAM0552	Arthropoda	Insecta	Hymenoptera	Braconidae	Microgastrinae	Microplitis	Microplitis varicolor	Jose Leonardo Fernandez	jftriana@uoguelph.ca
CNCHZ118-09	MIC 000401		MIC 000401	128	GU141623			MIC 000401	Arthropoda	Insecta	Hymenoptera	Braconidae	Microgastrinae	Apanteles	Apanteles petrovae	Jose Leonardo Fernandez	jftriana@uoguelph.ca
CNCHZ1189-09	CAM0569	CAM0569	CAM0569	657				CAM0569	Arthropoda	Insecta	Hymenoptera	Braconidae	Microgastrinae	Cotesia	Cotesia jft07	Jose Leonardo Fernandez	jftriana@uoguelph.ca
CNCHZ1190-09	CAM0570	CAM0570	CAM0570	657				CAM0570	Arthropoda	Insecta	Hymenoptera	Braconidae	Microgastrinae	Cotesia	Cotesia jft07	Jose Leonardo Fernandez	jftriana@uoguelph.ca
CNCHZ119-09	MIC 000402	MIC 000402	MIC 000402	144	GU141774			MIC 000402	Arthropoda	Insecta	Hymenoptera	Braconidae	Microgastrinae	Microplitis	Microplitis jft01	Jose Leonardo Fernandez	jftriana@uoguelph.ca
CNCHZ120-09	MIC 000403		MIC 000403	144	GU141567			MIC 000403	Arthropoda	Insecta	Hymenoptera	Braconidae	Microgastrinae	Apanteles	Apanteles jft01	Jose Leonardo Fernandez	jftriana@uoguelph.ca
CNCHZ121-09	MIC 000404		MIC 000404	122	GU141573			MIC 000404	Arthropoda	Insecta	Hymenoptera	Braconidae	Microgastrinae	Illidops	Illidops jft03	Jose Leonardo Fernandez	jftriana@uoguelph.ca
CNCHZ122-09	MIC 000405		MIC 000405	120	GU141821			MIC 000405	Arthropoda	Insecta	Hymenoptera	Braconidae	Microgastrinae	Protapanteles	Protapanteles jft02	Jose Leonardo Fernandez	jftriana@uoguelph.ca
CNCHZ123-09	MIC 000406		MIC 000406	144	GU141578			MIC 000406	Arthropoda	Insecta	Hymenoptera	Braconidae	Microgastrinae	Cotesia	Cotesia jft09	Jose Leonardo Fernandez	jftriana@uoguelph.ca
CNCHZ124-09	MIC 000407		MIC 000407	118	GU141676			MIC 000407	Arthropoda	Insecta	Hymenoptera	Braconidae	Microgastrinae	Glyptapanteles		Jose Leonardo Fernandez	jftriana@uoguelph.ca
CNCHZ125-09	MIC 000408		MIC 000408	126	GU141809			MIC 000408	Arthropoda	Insecta	Hymenoptera	Braconidae	Microgastrinae	Microplitis	Microplitis jft02	Jose Leonardo Fernandez	jftriana@uoguelph.ca
CNCHZ126-09	MIC 000409		MIC 000409	119	GU141820			MIC 000409	Arthropoda	Insecta	Hymenoptera	Braconidae	Microgastrinae	Protapanteles		Jose Leonardo Fernandez	jftriana@uoguelph.ca
CNCHZ127-09	MIC 000410		MIC 000410	0		0		MIC 000410	Arthropoda	Insecta	Hymenoptera	Braconidae	Microgastrinae	Apanteles		Jose Leonardo Fernandez	jftriana@uoguelph.ca
CNCHZ128-09	MIC 000411	MIC 000411	MIC 000411	113	GU141675			MIC 000411	Arthropoda	Insecta	Hymenoptera	Braconidae	Microgastrinae	Microgaster	Microgaster jft16	Jose Leonardo Fernandez	jftriana@uoguelph.ca
CNCHZ129-09	MIC 000412	MIC 000412	MIC 000412	127	GU141728			MIC 000412	Arthropoda	Insecta	Hymenoptera	Braconidae	Microgastrinae	Microgaster	Microgaster jft13	Jose Leonardo Fernandez	jftriana@uoguelph.ca
CNCHZ130-09	MIC 000413		MIC 000413	144	GU141610			MIC 000413	Arthropoda	Insecta	Hymenoptera	Braconidae	Microgastrinae	Diolcogaster		Jose Leonardo Fernandez	jftriana@uoguelph.ca
CNCHZ131-09	MIC 000414	MIC 000414	MIC 000414	122	GU141727			MIC 000414	Arthropoda	Insecta	Hymenoptera	Braconidae	Microgastrinae	Microgaster	Microgaster jft21	Jose Leonardo Fernandez	jftriana@uoguelph.ca
CNCHZ132-09	MIC 000415	MIC 000415	MIC 000415	144	GU141726			MIC 000415	Arthropoda	Insecta	Hymenoptera	Braconidae	Microgastrinae	Microgaster	Microgaster jft08	Jose Leonardo Fernandez	jftriana@uoguelph.ca
CNCHZ133-09	MIC 000416	MIC 000416	MIC 000416	128	GU141725			MIC 000416	Arthropoda	Insecta	Hymenoptera	Braconidae	Microgastrinae	Microgaster	Microgaster jft09	Jose Leonardo Fernandez	jftriana@uoguelph.ca
CNCHZ134-09	MIC 000417		MIC 000417	123	GU141622			MIC 000417	Arthropoda	Insecta	Hymenoptera	Braconidae	Microgastrinae	Dolichogenidea	Dolichogenidea jft06	Jose Leonardo Fernandez	jftriana@uoguelph.ca
CNCHZ135-09	MIC 000418		MIC 000418	127	GU141674			MIC 000418	Arthropoda	Insecta	Hymenoptera	Braconidae	Microgastrinae	Glyptapanteles		Jose Leonardo Fernandez	jftriana@uoguelph.ca
CNCHZ136-09	MIC 000419		MIC 000419	0		0		MIC 000419	Arthropoda	Insecta	Hymenoptera	Braconidae	Microgastrinae	Apanteles		Jose Leonardo Fernandez	jftriana@uoguelph.ca
CNCHZ137-09	MIC 000420		MIC 000420	0		0		MIC 000420	Arthropoda	Insecta	Hymenoptera	Braconidae	Microgastrinae	Cotesia		Jose Leonardo Fernandez	jftriana@uoguelph.ca
CNCHZ138-09	MIC 000421	MIC 000421	MIC 000421	129	GU141765			MIC 000421	Arthropoda	Insecta	Hymenoptera	Braconidae	Microgastrinae	Microgaster	Microgaster jft05	Jose Leonardo Fernandez	jftriana@uoguelph.ca
CNCHZ139-09	MIC 000422	MIC 000422	MIC 000422	118	GU141724			MIC 000422	Arthropoda	Insecta	Hymenoptera	Braconidae	Microgastrinae	Microgaster	Microgaster jft10	Jose Leonardo Fernandez	jftriana@uoguelph.ca
CNCHZ140-09	MIC 000423	MIC 000423	MIC 000423	126	GU141723			MIC 000423	Arthropoda	Insecta	Hymenoptera	Braconidae	Microgastrinae	Microgaster	Microgaster jft21	Jose Leonardo Fernandez	jftriana@uoguelph.ca
CNCHZ141-09	MIC 000424		MIC 000424	0		0		MIC 000424	Arthropoda	Insecta	Hymenoptera	Braconidae	Microgastrinae	Microgaster		Jose Leonardo Fernandez	jftriana@uoguelph.ca
CNCHZ142-09	MIC 000425		MIC 000425	0		0		MIC 000425	Arthropoda	Insecta	Hymenoptera	Braconidae	Microgastrinae	Sathon		Jose Leonardo Fernandez	jftriana@uoguelph.ca
CNCHZ143-09	MIC 000426		MIC 000426	129	GU141673			MIC 000426	Arthropoda	Insecta	Hymenoptera	Braconidae	Microgastrinae	Glyptapanteles		Jose Leonardo Fernandez	jftriana@uoguelph.ca
CNCHZ144-09	MIC 000427		MIC 000427	144	GU141672			MIC 000427	Arthropoda	Insecta	Hymenoptera	Braconidae	Microgastrinae	Glyptapanteles		Jose Leonardo Fernandez	jftriana@uoguelph.ca
CNCHZ145-09	MIC 000428		MIC 000428	144	GU141621			MIC 000428	Arthropoda	Insecta	Hymenoptera	Braconidae	Microgastrinae	Dolichogenidea	Dolichogenidea jft03	Jose Leonardo Fernandez	jftriana@uoguelph.ca
CNCHZ146-09	MIC 000429	MIC 000429	MIC 000429	130	GU141721			MIC 000429	Arthropoda	Insecta	Hymenoptera	Braconidae	Microgastrinae	Illidops	Illidops jft03	Jose Leonardo Fernandez	jftriana@uoguelph.ca
CNCHZ147-09	MIC 000430		MIC 000430	129	GU141671			MIC 000430	Arthropoda	Insecta	Hymenoptera	Braconidae	Microgastrinae	Glyptapanteles		Jose Leonardo Fernandez	jftriana@uoguelph.ca
CNCHZ148-09	MIC 000431		MIC 000431	129	GU141670			MIC 000431	Arthropoda	Insecta	Hymenoptera	Braconidae	Microgastrinae	Glyptapanteles	Glyptapanteles jft05	Jose Leonardo Fernandez	jftriana@uoguelph.ca
CNCHZ149-09	MIC 000432		MIC 000432	121	GU141577			MIC 000432	Arthropoda	Insecta	Hymenoptera	Braconidae	Microgastrinae	Cotesia	Cotesia jft25	Jose Leonardo Fernandez	jftriana@uoguelph.ca
CNCHZ150-09	MIC 000433		MIC 000433	128	GU141576			MIC 000433	Arthropoda	Insecta	Hymenoptera	Braconidae	Microgastrinae	Cotesia	Cotesia jft09	Jose Leonardo Fernandez	jftriana@uoguelph.ca
CNCHZ151-09	MIC 000434		MIC 000434	134	GU141575			MIC 000434	Arthropoda	Insecta	Hymenoptera	Braconidae	Microgastrinae	Cotesia	Cotesia jft09	Jose Leonardo Fernandez	jftriana@uoguelph.ca
CNCHZ152-09	MIC 000435		MIC 000435	129	GU141574			MIC 000435	Arthropoda	Insecta	Hymenoptera	Braconidae	Microgastrinae	Cotesia	Cotesia jft09	Jose Leonardo Fernandez	jftriana@uoguelph.ca
CNCHZ153-09	MIC 000436		MIC 000436	0		0		MIC 000436	Arthropoda	Insecta	Hymenoptera	Braconidae	Microgastrinae			Jose Leonardo Fernandez	jftriana@uoguelph.ca
CNCHZ154-09	MIC 000437		MIC 000437	0		0		MIC 000437	Arthropoda	Insecta	Hymenoptera	Braconidae	Microgastrinae	Apanteles		Jose Leonardo Fernandez	jftriana@uoguelph.ca
CNCHZ155-09	MIC 000438		MIC 000438	0		0		MIC 000438	Arthropoda	Insecta	Hymenoptera	Braconidae	Microgastrinae	Sathon		Jose Leonardo Fernandez	jftriana@uoguelph.ca
CNCHZ156-09	MIC 000439		MIC 000439	144	GU141827			MIC 000439	Arthropoda	Insecta	Hymenoptera	Braconidae	Microgastrinae	Sathon		Jose Leonardo Fernandez	jftriana@uoguelph.ca
CNCHZ157-09	MIC 000440		MIC 000440	144	GU141773			MIC 000440	Arthropoda	Insecta	Hymenoptera	Braconidae	Microgastrinae	Microplitis	Microplitis jft17	Jose Leonardo Fernandez	jftriana@uoguelph.ca
CNCHZ158-09	MIC 000441	MIC 000441	MIC 000441	144	GU141772			MIC 000441	Arthropoda	Insecta	Hymenoptera	Braconidae	Microgastrinae	Microplitis	Microplitis varicolor	Jose Leonardo Fernandez	jftriana@uoguelph.ca
CNCHZ159-09	MIC 000442		MIC 000442	129	GU141620			MIC 000442	Arthropoda	Insecta	Hymenoptera	Braconidae	Microgastrinae	Dolichogenidea	Dolichogenidea jft02	Jose Leonardo Fernandez	jftriana@uoguelph.ca
CNCHZ160-09	MIC 000443	MIC 000443	MIC 000443	116	GU141722			MIC 000443	Arthropoda	Insecta	Hymenoptera	Braconidae	Microgastrinae	Microgaster	Microgaster jft10	Jose Leonardo Fernandez	jftriana@uoguelph.ca
CNCHZ161-09	MIC 000444		MIC 000444	116	GU141826			MIC 000444	Arthropoda	Insecta	Hymenoptera	Braconidae	Microgastrinae	Sathon		Jose Leonardo Fernandez	jftriana@uoguelph.ca
CNCHZ162-09	MIC 000445		MIC 000445	121	GU141669			MIC 000445	Arthropoda	Insecta	Hymenoptera	Braconidae	Microgastrinae	Glyptapanteles		Jose Leonardo Fernandez	jftriana@uoguelph.ca
CNCHZ163-09	MIC 000446		MIC 000446	129	GU141825			MIC 000446	Arthropoda	Insecta	Hymenoptera	Braconidae	Microgastrinae	Sathon		Jose Leonardo Fernandez	jftriana@uoguelph.ca
CNCHZ164-09	MIC 000447		MIC 000447	0		0		MIC 000447	Arthropoda	Insecta	Hymenoptera	Braconidae	Microgastrinae	Glyptapanteles		Jose Leonardo Fernandez	jftriana@uoguelph.ca
CNCHZ165-09	MIC 000448		MIC 000448	126	GU141824			MIC 000448	Arthropoda	Insecta	Hymenoptera	Braconidae	Microgastrinae	Sathon		Jose Leonardo Fernandez	jftriana@uoguelph.ca
CNCHZ166-09	MIC 000449		MIC 000449	144	GU141823			MIC 000449	Arthropoda	Insecta	Hymenoptera	Braconidae	Microgastrinae	Sathon		Jose Leonardo Fernandez	jftriana@uoguelph.ca
CNCHZ167-09	MIC 000450		MIC 000450	144	GU141668			MIC 000450	Arthropoda	Insecta	Hymenoptera	Braconidae	Microgastrinae	Glyptapanteles		Jose Leonardo Fernandez	jftriana@uoguelph.ca
CNCHZ168-09	MIC 000451		MIC 000451	144	GU141619			MIC 000451	Arthropoda	Insecta	Hymenoptera	Braconidae	Microgastrinae	Dolichogenidea	Dolichogenidea jft02	Jose Leonardo Fernandez	jftriana@uoguelph.ca
CNCHZ169-09	MIC 000452		MIC 000452	144	GU141618			MIC 000452	Arthropoda	Insecta	Hymenoptera	Braconidae	Microgastrinae	Dolichogenidea	Dolichogenidea jft03	Jose Leonardo Fernandez	jftriana@uoguelph.ca
CNCHZ170-09	MIC 000453		MIC 000453	144	GU141617			MIC 000453	Arthropoda	Insecta	Hymenoptera	Braconidae	Microgastrinae	Dolichogenidea	Dolichogenidea jft03	Jose Leonardo Fernandez	jftriana@uoguelph.ca
CNCHZ171-09	MIC 000454		MIC 000454	0		0		MIC 000454	Arthropoda	Insecta	Hymenoptera	Braconidae	Microgastrinae	Dolichogenidea		Jose Leonardo Fernandez	jftriana@uoguelph.ca
CNCHZ172-09	MIC 000455		MIC 000455	134	GU141616			MIC 000455	Arthropoda	Insecta	Hymenoptera	Braconidae	Microgastrinae	Dolichogenidea	Dolichogenidea jft01	Jose Leonardo Fernandez	jftriana@uoguelph.ca
CNCHZ173-09	MIC 000456		MIC 000456	0		0		MIC 000456	Arthropoda	Insecta	Hymenoptera	Braconidae	Microgastrinae	Apanteles		Jose Leonardo Fernandez	jftriana@uoguelph.ca
CNCHZ174-09	MIC 000457		MIC 000457	0		0		MIC 000457	Arthropoda	Insecta	Hymenoptera	Braconidae	Microgastrinae	Dolichogenidea		Jose Leonardo Fernandez	jftriana@uoguelph.ca
CNCHZ175-09	MIC 000458		MIC 000458	134	GU141615			MIC 000458	Arthropoda	Insecta	Hymenoptera	Braconidae	Microgastrinae	Dolichogenidea	Dolichogenidea jft02	Jose Leonardo Fernandez	jftriana@uoguelph.ca
CNCHZ176-09	MIC 000459		MIC 000459	0		0		MIC 000459	Arthropoda	Insecta	Hymenoptera	Braconidae	Microgastrinae	Dolichogenidea		Jose Leonardo Fernandez	jftriana@uoguelph.ca
CNCHZ177-09	MIC 000460		MIC 000460	129	GU141614			MIC 000460	Arthropoda	Insecta	Hymenoptera	Braconidae	Microgastrinae	Dolichogenidea	Dolichogenidea jft03	Jose Leonardo Fernandez	jftriana@uoguelph.ca
CNCHZ178-09	MIC 000461		MIC 000461	0		0		MIC 000461	Arthropoda	Insecta	Hymenoptera	Braconidae	Microgastrinae	Apanteles		Jose Leonardo Fernandez	jftriana@uoguelph.ca
CNCHZ179-09	MIC 000462		MIC 000462	0		0		MIC 000462	Arthropoda	Insecta	Hymenoptera	Braconidae	Microgastrinae	Apanteles		Jose Leonardo Fernandez	jftriana@uoguelph.ca
CNCHZ180-09	MIC 000463		MIC 000463	0		0		MIC 000463	Arthropoda	Insecta	Hymenoptera	Braconidae	Microgastrinae	Apanteles		Jose Leonardo Fernandez	jftriana@uoguelph.ca
CNCHZ181-09	MIC 000464		MIC 000464	144	GU141566			MIC 000464	Arthropoda	Insecta	Hymenoptera	Braconidae	Microgastrinae	Apanteles	Apanteles jft01	Jose Leonardo Fernandez	jftriana@uoguelph.ca
CNCHZ182-09	MIC 000465		MIC 000465	0		0		MIC 000465	Arthropoda	Insecta	Hymenoptera	Braconidae	Microgastrinae	Dolichogenidea		Jose Leonardo Fernandez	jftriana@uoguelph.ca
CNCHZ183-09	MIC 000466		MIC 000466	0		0		MIC 000466	Arthropoda	Insecta	Hymenoptera	Braconidae	Microgastrinae	Dolichogenidea		Jose Leonardo Fernandez	jftriana@uoguelph.ca
CNCHZ184-09	MIC 000467		MIC 000467	0		0		MIC 000467	Arthropoda	Insecta	Hymenoptera	Braconidae	Microgastrinae	Diolcogaster		Jose Leonardo Fernandez	jftriana@uoguelph.ca
CNCHZ185-09	MIC 000468		MIC 000468	144	GU141771			MIC 000468	Arthropoda	Insecta	Hymenoptera	Braconidae	Microgastrinae	Microplitis	Microplitis jft38	Jose Leonardo Fernandez	jftriana@uoguelph.ca
CNCHZ186-09	MIC 000469		MIC 000469	144	GU141770			MIC 000469	Arthropoda	Insecta	Hymenoptera	Braconidae	Microgastrinae	Microplitis	Microplitis jft38	Jose Leonardo Fernandez	jftriana@uoguelph.ca
CNCHZ187-09	MIC 000470		MIC 000470	130	GU141769			MIC 000470	Arthropoda	Insecta	Hymenoptera	Braconidae	Microgastrinae	Microplitis	Microplitis jft12	Jose Leonardo Fernandez	jftriana@uoguelph.ca
CNCHZ188-09	MIC 000471		MIC 000471	131	GU141768			MIC 000471	Arthropoda	Insecta	Hymenoptera	Braconidae	Microgastrinae	Microplitis	Microplitis varicolor	Jose Leonardo Fernandez	jftriana@uoguelph.ca
CNCHZ189-09	MIC 000472		MIC 000472	0		0		MIC 000472	Arthropoda	Insecta	Hymenoptera	Braconidae	Microgastrinae	Microplitis		Jose Leonardo Fernandez	jftriana@uoguelph.ca
CNCHZ190-09	MIC 000473		MIC 000473	0		0		MIC 000473	Arthropoda	Insecta	Hymenoptera	Braconidae	Microgastrinae	Glyptapanteles		Jose Leonardo Fernandez	jftriana@uoguelph.ca
CNCHZ199-09	CAM0009	CAM0009	CAM0009	657				CAM0009	Arthropoda	Insecta	Hymenoptera	Braconidae	Microgastrinae	Apanteles	Apanteles ensiger	Jose Leonardo Fernandez	jftriana@uoguelph.ca
CNCHZ209-09	CAM0019	CAM0019	CAM0019	657				CAM0019	Arthropoda	Insecta	Hymenoptera	Braconidae	Microgastrinae	Apanteles	Apanteles petrovae	Jose Leonardo Fernandez	jftriana@uoguelph.ca
CNCHZ212-09	CAM0022	CAM0022	CAM0022	657				CAM0022	Arthropoda	Insecta	Hymenoptera	Braconidae	Microgastrinae	Apanteles	Apanteles ensiger	Jose Leonardo Fernandez	jftriana@uoguelph.ca
CNCHZ214-09	CAM0024	CAM0024	CAM0024	657				CAM0024	Arthropoda	Insecta	Hymenoptera	Braconidae	Microgastrinae	Apanteles	Apanteles ensiger	Jose Leonardo Fernandez	jftriana@uoguelph.ca
CNCHZ215-09	CAM0025	CAM0025	CAM0025	657				CAM0025	Arthropoda	Insecta	Hymenoptera	Braconidae	Microgastrinae	Apanteles	Apanteles ensiger	Jose Leonardo Fernandez	jftriana@uoguelph.ca

ProcessID	Sex	Reproduction	Life Stage	Extra Info	Notes	Voucher Type	Tissue Type	Collectors	Date Collected	Continent	Country/Ocean	Province	Region	Sector	Exact Site	Lat	Lon	Elev	GPS Source
CNCHZ064-09								W. Mason	07-Jul-1958		Canada	Quebec			Payne Bay	59.5499992	-69.339996		
CNCHZ065-09								W. Mason	13-Jul-1971		Canada	Northwest Territories			Kovaluk River	69.1100006	-131		
CNCHZ066-09								E. Sterns	17-Jul-1955		Canada	Newfoundland and Labrador			Cartwright	53.4199982	-57.009998		
CNCHZ067-09								G. Shewell	04-Jul-1966		Canada	Northwest Territories			Ford Lake	63.1100006	-107.19		
CNCHZ068-09								G. Shewell	18-Jul-1966		Canada	Nunavut			Nr. Beechey Lake	65.1399994	-106.5		
CNCHZ069-09								W. Mason	18-Jul-1971		Canada	Northwest Territories			32 Km E of Tuktoyaktuk	69.3000031	-132.14999		
CNCHZ070-09		S	A					A. Macpherson	05-Jul-1956		Canada	Nunavut			Pelly Bay	68.3199997	-89.5		
CNCHZ071-09		S	A					A. Macpherson	05-Jul-1956		Canada	Nunavut			Pelly Bay	68.3199997	-89.5		
CNCHZ072-09								G. Shewell	18-Jun-1966		Canada	Northwest Territories			Yellowknife, road nr. St	62.2700005	-114.24		
CNCHZ073-09								Canadian Arctic Ex	12-Jul-1915		Canada	Northwest Territories			Bernard Harbour	68.4599991	-114.42		
CNCHZ074-09								S. D. Hicks	02-Jul-1957		Canada	Northwest Territories			Fort McPherson	67.2600021	-134.53		
CNCHZ075-09								J. Chillcot	21-Jul-1951		Canada	Northwest Territories			Spence Bay	69.3199997	-93.32		
CNCHZ076-09								G. Shewell	28-Jun-1966		Canada	Northwest Territories			Ford Lake	63.1100006	-107.19		
CNCHZ077-09									13-Jul-1960		Canada	Northwest Territories			Mouth of Liard River	61.5200005	-121.21		
CNCHZ078-09									13-Jul-1955		Canada	Northwest Territories			Mouth of Liard River	61.5200005	-121.21		
CNCHZ079-09		S	A					G. Shewell	18-Jun-1966		Canada	Northwest Territories			Yellowknife, road nr. St	62.2700005	-114.24		
CNCHZ080-09		S	A					J. Chillcot	30-Jun-1953		Canada	Northwest Territories			Salmita Mines	64.0500031	-111.15		
CNCHZ081-09		S	A			NULL		W. Mason	20-Jun-1971		Canada	Northwest Territories			32 Km E of Tuktoyaktuk	69.3000031	-132.14999		
CNCHZ082-09								G. Walley	19-Jul-1934		Canada	Ontario			Moosonee	51.1599998	-80.389999		
CNCHZ083-09		S	A			NULL		W. Mason	25-Jun-1971		Canada	Northwest Territories			Kovaluk River	69.1100006	-131		
CNCHZ084-09		S	A					G. Shewell	23-Jun-1966		Canada	Northwest Territories			Ford Lake	63.1100006	-107.19		
CNCHZ085-09								J. Chillcot	04-Jul-1953		Canada	Northwest Territories			Salmita Mines	64.0500031	-111.15		
CNCHZ086-09								R. Hall	16-Aug-1949		Canada	Northwest Territories			Yellowknife	62.2700005	-114.24		
CNCHZ087-09								J. Chillcot	02-Jul-1953		Canada	Northwest Territories			Salmita Mines	64.0500031	-111.15		
CNCHZ088-09								W. Mason	21-Jul-1949		Canada	Northwest Territories			Norman Wells	65.1699982	-126.51		
CNCHZ089-09		S	A					J. Chillcot	14-Aug-1950		Canada	Northwest Territories			Chesterfield	63.2000008	-90.419998		
CNCHZ090-09		S	A			NULL		J. Chillcot	22-Jul-1951		Canada	Northwest Territories			Spence Bay	69.3199997	-93.32		
CNCHZ091-09								G. Shewell	02-Jul-1966		Canada	Northwest Territories			Ford Lake	63.1100006	-107.19		
CNCHZ092-09								H. Huckel	22-Jul-1954		Canada	Quebec			Sugluk	62.1199989	-75.379997		
CNCHZ093-09								E. F. Cashman	25-Jun-1955		Canada	Quebec			Cartwright	53.4199982	-57.009998		
CNCHZ094-09								R. McCondochie	16-Aug-1954		Canada	Quebec			Payne Bay	59.5499992	-69.339996		
CNCHZ095-09								R. Coyles	23-Jul-1954		Canada	Quebec			Indian House Lake	56.1500015	-64.419998		
CNCHZ096-09		S	A					R. E. Leech	12-Jul-1959		Canada	British Columbia			Summit Lake, Mile 392	58.3800011	-124.41	1295	
CNCHZ097-09		S	A					E. E. Sterns	20-Jun-1955		Canada	Quebec			Cartwright	53.4199982	-57.009998		
CNCHZ098-09		S	A			NULL		J. Chillcot	12-Jul-1953		Canada	Nunavut			Muskox Lake	64.4499969	-108.1		
CNCHZ099-09		S	A			NULL		J. R. Lonsway	14-Jun-1956		Canada	Quebec			Mistassini Post	50.25	-73.519997		
CNCHZ100-09								W. Mason	10-Jul-1952		United States	Alaska			King Salmon, Naknek F	58.4099998	-156.39		
CNCHZ101-09								J. R. McGillis	13-Jul-1951		United States	Alaska			Big Delta	64.9000015	-145.48		
CNCHZ102-09		S	A			NULL		W. R. Richards	12-Jul-1954		Canada	Quebec			Indian House Lake	56.1500015	-64.419998		
CNCHZ103-09		S	A					R. E. Leech	08-Jul-1959		Canada	British Columbia			Summit Lake, Mile 392	58.3800011	-124.41	1295	
CNCHZ104-09								B. S. Heming	20-Jun-1961		United States	Alaska			Unalakleet	63.5299988	-160.47		
CNCHZ105-09								E. F. Cashman	02-Jul-1956		Canada	Northwest Territories			Aklavik	68.1299973	-135.00999		
CNCHZ106-09		S	A					L. Leblanc	08-Jun-1987		Canada	Quebec			Highway to James Bay,	50.0299988	-77.07		
CNCHZ107-09								W. R. Richards	11-Aug-1954		Canada	Quebec			Indian House Lake	56.1500015	-64.419998		
CNCHZ108-09								W. Mason	13-Jun-1948		Canada	British Columbia			Fort Nelson	58.4799995	-122.42		
CNCHZ109-09								S & J. Peck	12-Aug-1984		United States	Alaska			Chena River, 48 Km E	64.5	-146.38		
CNCHZ110-09		S	A					G. Ball	22-Jun-1953		Canada	Alberta			Fort McMurray	56.4399986	-111.23		
CNCHZ111-09		S	A					E. E. McDougall	12-Jul-1959		Canada	British Columbia			Summit Lake, Mile 392	58.3800011	-124.41	1295	
CNCHZ112-09		S	A					W. W. Moss	10-Aug-1960		Canada	British Columbia			Lisadele Lake	58.4099998	-133.03999	1210	
CNCHZ113-09		S	A			NULL		R. E. Leech	13-Jul-1962		United States	Alaska			Isabel Pass, Richardso	63.1899986	-145.56	880	
CNCHZ114-09								Mason & McGillis	11-Jul-1951		United States	Alaska			Shaw Creek, Richardso	64.1600037	-146.06		
CNCHZ1141-09								A. Bennett			Canada	Ontario			Mixed forest	45.2346992	-75.624001		
CNCHZ1142-09								A. Bennett			Canada	Ontario			Mixed forest	45.2346992	-75.624001		
CNCHZ1143-09								A. Bennett			Canada	Ontario			Mixed forest	45.2346992	-75.624001		
CNCHZ1144-09								A. Bennett			Canada	Ontario			Mixed forest	45.2346992	-75.624001		
CNCHZ1145-09								A. Bennett			Canada	Ontario			Mixed forest	45.2346992	-75.624001		
CNCHZ1146-09								A. Bennett			Canada	Ontario			Mixed forest	45.2346992	-75.624001		
CNCHZ1147-09								A. Bennett			Canada	Ontario			Mixed forest	45.2346992	-75.624001		
CNCHZ1148-09								A. Bennett			Canada	Ontario			Mixed forest	45.2346992	-75.624001		
CNCHZ1149-09								A. Bennett			Canada	Ontario			Mixed forest	45.2346992	-75.624001		
CNCHZ1150-09								A. Bennett			Canada	Ontario			Mixed forest	45.2346992	-75.624001		
CNCHZ115-09								W. Mason	30-Jun-1951		United States	Alaska			Big Delta	64.9000015	-145.48		
CNCHZ1151-09								A. Bennett			Canada	Ontario			Mixed forest	45.2346992	-75.624001		
CNCHZ1154-09								A. Bennett			Canada	Ontario			Mixed forest	45.2346992	-75.624001		
CNCHZ1158-09		S	A			NULL		A. Bennett			Canada	Ontario			Mixed forest	45.2350006	-75.624001		
CNCHZ116-09								J. B. Hartley	03-Aug-1952		United States	Alaska			King Salmon, Naknek F	58.4099998	-156.39		
CNCHZ117-09		S	A			NULL		W. Mason	20-Jul-1952		United States	Alaska			Naknek, on tundra	58.4399986	-156.58		
CNCHZ1171-09								A. Bennett			Canada	Ontario			Mixed forest	45.2346992	-75.624001		
CNCHZ1172-09								A. Bennett			Canada	Ontario			Mixed forest	45.2346992	-75.624001		
CNCHZ118-09								Mason & McGillis	11-Jul-1951		United States	Alaska			Shaw Creek, Richardso	64.1600037	-146.06		
CNCHZ1189-09		S	A					J. Fernandez	11-Nov-2008		Canada	Quebec			Chelsea, near old visito				
CNCHZ1190-09		S	A					J. Fernandez	11-Nov-2008		Canada	Quebec			Chelsea, near old visito				
CNCHZ119-09		S	A			NULL		P. J. Skitsko	18-Jul-1962		United States	Alaska			Isabel Pass, Richardso	63.1899986	-145.56	880	
CNCHZ120-09								W. Mason	17-Aug-1959		Canada	Quebec			Chimo	58.0600014	-68.230003		
CNCHZ121-09						NULL		J. R. Vockeroth	13-Aug-1949		Canada	Quebec			Great Whale River	55.1699982	-77.449997		
CNCHZ122-09								W. R. Richards	09-Jul-1954		Canada	Quebec			Indian House Lake	56.1500015	-64.419998		
CNCHZ123-09		S	A					H. Huckel	13-Aug-1954		Canada	Quebec			Sugluk	62.1199989	-75.379997		
CNCHZ124-09								R. Madge	25-Jul-1961		United States	Alaska			Cape Thompson	68.0899963	-165.59		
CNCHZ125-09		S	A					J. Matthews	31-Aug-1968		United States	Alaska			Deering	66.0500031	-162.44		
CNCHZ126-09								W. Mason	29-Jun-1952		United States	Alaska			Curry	62.3699989	-150.00999		
CNCHZ127-09								J. R. McGillis	13-Jul-1951		United States	Alaska			Big Delta	64.9000015	-145.48		
CNCHZ128-09		S	A					W. Mason	20-Jul-1952		United States	Alaska			Naknek, on tundra	58.4399986	-156.58		
CNCHZ129-09		S	A					W. Mason	26-Jul-1951		United States	Alaska			Alaska Highway, Km 4	64.0199966	-145.42999		
CNCHZ130-09								R. Madge	20-Jul-1959		United States	Alaska			Umiat	69.2200012	-152.09		
CNCHZ131-09		S	A					W. Mason	16-Jul-1952		United States	Alaska			Fairbanks	64.5	-147.42999		
CNCHZ132-09		S	A			NULL		Mason & McGillis	27-Jul-1951		United States	Alaska			Alaska Highway, Km 4	64	-145.45		
CNCHZ133-09		S	A					Mason & McGillis	28-Jul-1951		United States	Alaska			Shaw Creek, Richardso	64.1600037	-146.06		
CNCHZ134-09								W. Mason	17-Aug-1959		Canada	Quebec			Chimo	58.0600014	-68.230003		
CNCHZ135-09								E. E. McDougall	29-Jul-1958		Canada	Quebec			Payne Bay	59.5499992	-69.339996		
CNCHZ136-09								R. Coyles	15-Aug-1954		Canada	Quebec			Indian House Lake	56.1500015	-64.419998		
CNCHZ137-09								E. F. Cashman	23-Jun-1955		Canada	Quebec			Cartwright	53.4199982	-57.009998		
CNCHZ138-09		S	A					W. E. Beckel	21-Jun-1948		Canada	Quebec			Goose Bay	53.1800003	-60.25		
CNCHZ139-09		S	A					J. R. McGillis	26-Jul-1956		Canada	Quebec			Mistassini Post	50.25	-73.519997		
CNCHZ140-09		S	A					R. Lambert	08-Jul-1953		Canada	Quebec			Cape Rouge	46.4500008	-71.209999		
CNCHZ141-09								J. R. Lonsway	14-Jul-1956		Canada	Quebec			Mistassini Post	50.25	-73.519997		
CNCHZ142-09								W. R. Richards	11-Aug-1954		Canada	Quebec			Indian House Lake	56.1500015	-64.419998		
CNCHZ143-09								W. Mason	09-Aug-1958		Canada	Quebec			Payne Bay	59.5499992	-69.339996		
CNCHZ144-09								W. Mason	31-Jul-1958		Canada	Quebec			Payne Bay	59.5499992	-69.339996		
CNCHZ145-09								B. S. Heming	13-Jun-1961		United States	Alaska			Unalakleet	63.5299988	-160.47		
CNCHZ146-09		S	A			NULL		J. Chillcot	12-Jul-1952		Canada	Manitoba	Churchill		Fort Churchill	58.7340012	-93.82		
CNCHZ147-09								H. Huckel	22-Jul-1954		Canada	Quebec			Sugluk	62.1199989	-75.379997		
CNCHZ148-09								W. Mason	17-Aug-1959		Canada	Quebec			Chimo	58.0600014	-68.230003		
CNCHZ149-09		S	A					J. F. McAlpine	09-Aug-1954		Canada	Newfoundland and Labrador			Hebron	58.1199989	-62.240002		
CNCHZ150-09		S	A					E. E. Sterns	25-Jul-1954		Canada	Newfoundland and Labrador			Nutak	57.2799988	-61.490002		
CNCHZ151-09		S	A					J. F. McAlpine	16-Jul-1954		Canada	Newfoundland and Labrador			Hebron	58.1199989	-62.240002		
CNCHZ152-09		S	A					J. F. McAlpine	24-Jul-1954		Canada	Newfoundland and Labrador			Nutak	57.2799988	-61.490002		
CNCHZ153-09								J. R. Vockeroth	13-Jul-1949		Canada	Quebec			Great Whale River	55.1699982	-77.449997		
CNCHZ154-09								J. R. Vockeroth	05-Aug-1949		Canada	Quebec			Great Whale River	55.1699982	-77.449997		
CNCHZ155-09								S & J. Peck	16-Aug-1984		Canada	Yukon Territory			Chicken to Dawson	64.0500031	-140.5		
CNCHZ156-09								R. Madge	05-Aug-1961		United States	Alaska			Unalakleet	63.5299988	-160.47		
CNCHZ157-09		S	A					J. E. Martin	03-Jul-1959		United States	Alaska			Umiat	69.2200012	-152.09		
CNCHZ158-09		S	A			NULL		D. Whilliams	10-Jul-1951		United States	Alaska			Nome	64.3000031	-165.24001		
CNCHZ159-09								W. Mason	30-Jun-1951		United States	Alaska			Big Delta	64.9000015	-145.48		
CNCHZ160-09		S	A					W. Mason	28-Jun-1952		United States	Alaska			Curry	62.3699989	-150.00999		
CNCHZ161-09								Mason & McGillis	11-Jul-1951		United States	Alaska			Shaw Creek, Richardso	64.1600037	-146.06		
CNCHZ162-09								B. S. Heming	14-Aug-1961		United States	Alaska			Unalakleet	63.5299988	-160.47		
CNCHZ163-09								W. Mason	21-Jul-1952		United States	Alaska			Cold Bay	55.1300011	-162.42999		
CNCHZ164-09								J. Matthews	14-Aug-1968		United States	Alaska			Deering	66.0500031	-162.44		
CNCHZ165-09								R. S. Bigelow	25-Jul-1952		United States	Alaska			Anchorage	61.1300011	-149.53999		
CNCHZ166-09								W. Mason	21-Jul-1952		United States	Alaska			Cold Bay	55.1300011	-162.42999		
CNCHZ167-09								R. Madge	29-Jul-1961		United States	Alaska			Cape Thompson	68.0899963	-165.59		
CNCHZ168-09								W. W. Judd	24-Jun-1949		Canada	Yukon Territory			Dawson	64.0400009	-139.25999		
CNCHZ169-09								P. J. Skitsko	19-Aug-1962		Canada	Yukon Territory			Takhini Hot Springs	60.5299988	-135.21001	730	
CNCHZ170-09								R. E. Leech	16-Aug-1956		Canada	Yukon Territory			Firth River	69	-140		
CNCHZ171-09								J. E. Martin	19-Jul-1951		Canada	Yukon Territory			Rampart House	67.25	-140.59		
CNCHZ172-09								P. J. Skitsko	02-Aug-1962		Canada	Yukon Territory			23 km E of Dawson	64.0400009	-130.00999		
CNCHZ173-09								E. F. Cashman	25-Jul-1956		Canada	Yukon Territory			British Mountains, Firth	69.3000031	-139.3		
CNCHZ174-09								Mason & Hughes	09-Aug-1948		Canada	Yukon Territory			Marsh Lake	60.2599983	-135.14999		
CNCHZ175-09									06-Aug-1973		Canada	Yukon Territory			Dempster Highway, km	64.5899963	-138.12		
CNCHZ176-09								R. E. Morlan	01-Jul-1975		Canada	Yukon Territory			Old Crow	67.3399963	-139.48		
CNCHZ177-09								Mason & Hughes	17-Jul-1948		Canada	British Columbia			Near Chilkat Pass	59.4099998	-136.33	980	
CNCHZ178-09								G & M. Wood	23-Jul-1973		Canada	Yukon Territory			Dempster Highway, km	64.5899963	-138.12		
CNCHZ179-09								R. E. Morlan	01-Jul-1975		Canada	Yukon Territory			Old Crow	67.3399963	-139.48		
CNCHZ180-09								E. F. Cashman	05-Aug-1956		Canada	Yukon Territory			Firth River	69	-140		
CNCHZ181-09								P. J. Skitsko	13-Aug-1962		Canada	Yukon Territory			Gravel Lake, 95 Km E	63.4900017	-137.55		
CNCHZ182-09								W. Mason	27-Jul-1971		Canada	Yukon Territory			Herschel Island	69.3499985	-139.5		
CNCHZ183-09								W. Mason	27-Jul-1971		Canada	Yukon Territory			Herschel Island	69.3499985	-139.5		
CNCHZ184-09								S & J. Peck	16-Jun-1984		Canada	Yukon Territory			Dempster Highway	63.5800018	-138.42999		
CNCHZ185-09		S	A					W. Mason	27-Jul-1971		Canada	Yukon Territory			Herschel Island	69.3499985	-139.5		
CNCHZ186-09		S	A					W. Mason	27-Jul-1971		Canada	Yukon Territory			Herschel Island	69.3499985	-139.5		
CNCHZ187-09		S	A					R. E. Leech	13-Jun-1962		Canada	Yukon Territory			North Fork Pass, Ogilvi	64.6500015	-138.25		
CNCHZ188-09		S	A					J. E. Martin	21-Jun-1960		Canada	Yukon Territory			Ross River	61.5600014	-132.3	900	
CNCHZ189-09								R. E. Morlan	14-Jul-1975		Canada	Yukon Territory			Old Crow	67.3399963	-139.48		
CNCHZ190-09								W. Mason	19-Aug-1958		Canada	Quebec			Payne Bay	59.5499992	-69.339996		
CNCHZ199-09								H. Goulet	19-Sep-2007		Canada	Ontario	Ottawa		Ottawa city garden	45.3560982	-75.707001		
CNCHZ209-09								H. Goulet	30-May-2007		Canada	Ontario	Ottawa		Ottawa city garden	45.3560982	-75.707001		
CNCHZ212-09								H. Goulet	13-Jul-2007		Canada	Ontario	Ottawa		Ottawa city garden	45.3560982	-75.707001		
CNCHZ214-09								H. Goulet	30-Jul-2007		Canada	Ontario	Ottawa		Ottawa city garden	45.3560982	-75.707001		
CNCHZ215-09								H. Goulet	30-Jul-2007		Canada	Ontario	Ottawa		Ottawa city garden	45.3560982	-75.707001		

Table S5: All collection and accession information associated with specimens analysed here.

ProcessID	Sample ID	Field Num	Catalog Num	COI-5P Seq.	COI GenBank	28S Seq. Length	28S GenBan	Sample ID	Phylum	Class	Order	Family	Subfamily	Genus	Species	Identifier	Identifier email	Sex	Reproduction	Life Stage	Extra Info	Notes	Voucher Type	Tissue Type	Collectors	Date Collected	Continent	Country/Ocean	Province	Region	Sector	Exact Site	Lat	Lon	Elev	GPS Source
CNCHZ263-09	CAM0073	CAM0073	CAM0073	657				CAM0073	Arthropoda	Insecta	Hymenoptera	Braconidae	Microgastrinae	Apanteles	Apanteles petrovae	Jose Leonardo Fernandez	jftriana@uoguelph.ca								H. Goulet	26-Jun-2007		Canada	Ontario	Ottawa		Ottawa city garden	45.3560982	-75.707001		
CNCHZ264-09	CAM0074	CAM0074	CAM0074	657				CAM0074	Arthropoda	Insecta	Hymenoptera	Braconidae	Microgastrinae	Apanteles	Apanteles ensiger	Jose Leonardo Fernandez	jftriana@uoguelph.ca								H. Goulet	26-Jun-2007		Canada	Ontario	Ottawa		Ottawa city garden	45.3560982	-75.707001		
CNCHZ281-09	CAM0091	CAM0091	CAM0091	657				CAM0091	Arthropoda	Insecta	Hymenoptera	Braconidae	Microgastrinae	Microplitis	Microplitis varicolor	Jose Leonardo Fernandez	jftriana@uoguelph.ca								H. Goulet	10-Aug-2007		Canada	Ontario	Ottawa		Ottawa city garden	45.3560982	-75.707001		
CNCHZ283-09	CAM0093	CAM0093	CAM0093	657				CAM0093	Arthropoda	Insecta	Hymenoptera	Braconidae	Microgastrinae	Microplitis	Microplitis varicolor	Jose Leonardo Fernandez	jftriana@uoguelph.ca								H. Goulet	10-Aug-2007		Canada	Ontario	Ottawa		Ottawa city garden	45.3560982	-75.707001		
CNCHZ284-09	CAM0094	CAM0094	CAM0094	657				CAM0094	Arthropoda	Insecta	Hymenoptera	Braconidae	Microgastrinae	Microplitis	Microplitis varicolor	Jose Leonardo Fernandez	jftriana@uoguelph.ca								H. Goulet	10-Aug-2007		Canada	Ontario	Ottawa		Ottawa city garden	45.3560982	-75.707001		
CNCHZ285-09	CAM0095	CAM0095	CAM0095	657				CAM0095	Arthropoda	Insecta	Hymenoptera	Braconidae	Microgastrinae	Microplitis	Microplitis varicolor	Jose Leonardo Fernandez	jftriana@uoguelph.ca								H. Goulet	10-Aug-2007		Canada	Ontario	Ottawa		Ottawa city garden	45.3560982	-75.707001		
CNCHZ304-09	CAM0114	CAM0114	CAM0114	657				CAM0114	Arthropoda	Insecta	Hymenoptera	Braconidae	Microgastrinae	Apanteles	Apanteles ensiger	Jose Leonardo Fernandez	jftriana@uoguelph.ca								H. Goulet	10-Aug-2007		Canada	Ontario	Ottawa		Ottawa city garden	45.3560982	-75.707001		
CNCHZ317-09	CAM0127	CAM0127	CAM0127	657				CAM0127	Arthropoda	Insecta	Hymenoptera	Braconidae	Microgastrinae	Apanteles	Apanteles jft28	Jose Leonardo Fernandez	jftriana@uoguelph.ca								H. Goulet	15-Jul-2007		Canada	Ontario	Ottawa		Ottawa city garden	45.3560982	-75.707001		
CNCHZ318-09	CAM0128	CAM0128	CAM0128	657				CAM0128	Arthropoda	Insecta	Hymenoptera	Braconidae	Microgastrinae	Apanteles	Apanteles jft01	Jose Leonardo Fernandez	jftriana@uoguelph.ca								H. Goulet	02-Jul-2007		Canada	Ontario	Ottawa		Ottawa city garden	45.3560982	-75.707001		
CNCHZ344-09	CAM0154	CAM0154	CAM0154	657				CAM0154	Arthropoda	Insecta	Hymenoptera	Braconidae	Microgastrinae	Apanteles	Apanteles ensiger	Jose Leonardo Fernandez	jftriana@uoguelph.ca								H. Goulet	01-Sep-2007		Canada	Ontario	Ottawa		Ottawa city garden	45.3560982	-75.707001		
CNCHZ351-09	CAM0161	CAM0161	CAM0161	657				CAM0161	Arthropoda	Insecta	Hymenoptera	Braconidae	Microgastrinae	Apanteles	Apanteles ensiger	Jose Leonardo Fernandez	jftriana@uoguelph.ca								H. Goulet	01-Sep-2007		Canada	Ontario	Ottawa		Ottawa city garden	45.3560982	-75.707001		
CNCHZ359-09	CAM0169	CAM0169	CAM0169	657				CAM0169	Arthropoda	Insecta	Hymenoptera	Braconidae	Microgastrinae	Microplitis	Microplitis jft09	Jose Leonardo Fernandez	jftriana@uoguelph.ca								H. Goulet	10-Aug-2007		Canada	Ontario	Ottawa		Ottawa city garden	45.3560982	-75.707001		
CNCHZ360-09	CAM0170	CAM0170	CAM0170	657				CAM0170	Arthropoda	Insecta	Hymenoptera	Braconidae	Microgastrinae	Microplitis	Microplitis varicolor	Jose Leonardo Fernandez	jftriana@uoguelph.ca								H. Goulet	10-Aug-2007		Canada	Ontario	Ottawa		Ottawa city garden	45.3560982	-75.707001		
CNCHZ361-09	CAM0171	CAM0171	CAM0171	657				CAM0171	Arthropoda	Insecta	Hymenoptera	Braconidae	Microgastrinae	Microplitis	Microplitis varicolor	Jose Leonardo Fernandez	jftriana@uoguelph.ca								H. Goulet	10-Aug-2007		Canada	Ontario	Ottawa		Ottawa city garden	45.3560982	-75.707001		
CNCHZ362-09	CAM0172	CAM0172	CAM0172	657				CAM0172	Arthropoda	Insecta	Hymenoptera	Braconidae	Microgastrinae	Microplitis	Microplitis varicolor	Jose Leonardo Fernandez	jftriana@uoguelph.ca								H. Goulet	10-Aug-2007		Canada	Ontario	Ottawa		Ottawa city garden	45.3560982	-75.707001		
CNCHZ363-09	CAM0173	CAM0173	CAM0173	657				CAM0173	Arthropoda	Insecta	Hymenoptera	Braconidae	Microgastrinae	Microplitis	Microplitis varicolor	Jose Leonardo Fernandez	jftriana@uoguelph.ca								H. Goulet	10-Aug-2007		Canada	Ontario	Ottawa		Ottawa city garden	45.3560982	-75.707001		
CNCHZ365-09	CAM0175	CAM0175	CAM0175	657				CAM0175	Arthropoda	Insecta	Hymenoptera	Braconidae	Microgastrinae	Microplitis	Microplitis varicolor	Jose Leonardo Fernandez	jftriana@uoguelph.ca								H. Goulet	10-Aug-2007		Canada	Ontario	Ottawa		Ottawa city garden	45.3560982	-75.707001		
CNCHZ366-09	CAM0176	CAM0176	CAM0176	657				CAM0176	Arthropoda	Insecta	Hymenoptera	Braconidae	Microgastrinae	Microplitis	Microplitis varicolor	Jose Leonardo Fernandez	jftriana@uoguelph.ca								H. Goulet	10-Aug-2007		Canada	Ontario	Ottawa		Ottawa city garden	45.3560982	-75.707001		
CNCHZ367-09	CAM0177	CAM0177	CAM0177	657				CAM0177	Arthropoda	Insecta	Hymenoptera	Braconidae	Microgastrinae	Microplitis	Microplitis varicolor	Jose Leonardo Fernandez	jftriana@uoguelph.ca								H. Goulet	10-Aug-2007		Canada	Ontario	Ottawa		Ottawa city garden	45.3560982	-75.707001		
CNCHZ368-09	CAM0178	CAM0178	CAM0178	657				CAM0178	Arthropoda	Insecta	Hymenoptera	Braconidae	Microgastrinae	Microplitis	Microplitis varicolor	Jose Leonardo Fernandez	jftriana@uoguelph.ca								H. Goulet	10-Aug-2007		Canada	Ontario	Ottawa		Ottawa city garden	45.3560982	-75.707001		
CNCHZ370-09	CAM0180	CAM0180	CAM0180	646				CAM0180	Arthropoda	Insecta	Hymenoptera	Braconidae	Microgastrinae	Microplitis	Microplitis varicolor	Jose Leonardo Fernandez	jftriana@uoguelph.ca								H. Goulet	30-Jul-2007		Canada	Ontario	Ottawa		Ottawa city garden	45.3560982	-75.707001		
CNCHZ372-09	CAM0182	CAM0182	CAM0182	657				CAM0182	Arthropoda	Insecta	Hymenoptera	Braconidae	Microgastrinae	Microplitis	Microplitis varicolor	Jose Leonardo Fernandez	jftriana@uoguelph.ca								H. Goulet	30-Jul-2007		Canada	Ontario	Ottawa		Ottawa city garden	45.3560982	-75.707001		
CNCHZ373-09	CAM0183	CAM0183	CAM0183	657				CAM0183	Arthropoda	Insecta	Hymenoptera	Braconidae	Microgastrinae	Microplitis	Microplitis varicolor	Jose Leonardo Fernandez	jftriana@uoguelph.ca								H. Goulet	30-Jul-2007		Canada	Ontario	Ottawa		Ottawa city garden	45.3560982	-75.707001		
CNCHZ375-09	CAM0185	CAM0185	CAM0185	657				CAM0185	Arthropoda	Insecta	Hymenoptera	Braconidae	Microgastrinae	Microplitis	Microplitis varicolor	Jose Leonardo Fernandez	jftriana@uoguelph.ca								H. Goulet	30-Jul-2007		Canada	Ontario	Ottawa		Ottawa city garden	45.3560982	-75.707001		
CNCHZ376-09	CAM0186	CAM0186	CAM0186	657				CAM0186	Arthropoda	Insecta	Hymenoptera	Braconidae	Microgastrinae	Microplitis	Microplitis varicolor	Jose Leonardo Fernandez	jftriana@uoguelph.ca								H. Goulet	30-Jul-2007		Canada	Ontario	Ottawa		Ottawa city garden	45.3560982	-75.707001		
CNCHZ377-09	CAM0187	CAM0187	CAM0187	657				CAM0187	Arthropoda	Insecta	Hymenoptera	Braconidae	Microgastrinae	Microplitis	Microplitis varicolor	Jose Leonardo Fernandez	jftriana@uoguelph.ca								H. Goulet	30-Jul-2007		Canada	Ontario	Ottawa		Ottawa city garden	45.3560982	-75.707001		
CNCHZ378-09	CAM0188	CAM0188	CAM0188	657				CAM0188	Arthropoda	Insecta	Hymenoptera	Braconidae	Microgastrinae	Microplitis	Microplitis varicolor	Jose Leonardo Fernandez	jftriana@uoguelph.ca								H. Goulet	30-Jul-2007		Canada	Ontario	Ottawa		Ottawa city garden	45.3560982	-75.707001		
CNCHZ379-09	CAM0189	CAM0189	CAM0189	657				CAM0189	Arthropoda	Insecta	Hymenoptera	Braconidae	Microgastrinae	Microplitis	Microplitis varicolor	Jose Leonardo Fernandez	jftriana@uoguelph.ca								H. Goulet	30-Jul-2007		Canada	Ontario	Ottawa		Ottawa city garden	45.3560982	-75.707001		
CNCHZ380-09	CAM0190	CAM0190	CAM0190	657				CAM0190	Arthropoda	Insecta	Hymenoptera	Braconidae	Microgastrinae	Microplitis	Microplitis varicolor	Jose Leonardo Fernandez	jftriana@uoguelph.ca								H. Goulet	30-Jul-2007		Canada	Ontario	Ottawa		Ottawa city garden	45.3560982	-75.707001		
CNCHZ381-09	CAM0191	CAM0191	CAM0191	657				CAM0191	Arthropoda	Insecta	Hymenoptera	Braconidae	Microgastrinae	Microplitis	Microplitis varicolor	Jose Leonardo Fernandez	jftriana@uoguelph.ca								H. Goulet	16-Jun-2007		Canada	Ontario	Ottawa		Ottawa city garden	45.3560982	-75.707001		
CNCHZ382-09	CAM0192	CAM0192	CAM0192	657				CAM0192	Arthropoda	Insecta	Hymenoptera	Braconidae	Microgastrinae	Microplitis	Microplitis varicolor	Jose Leonardo Fernandez	jftriana@uoguelph.ca								H. Goulet	19-Sep-2007		Canada	Ontario	Ottawa		Ottawa city garden	45.3560982	-75.707001		
CNCHZ383-09	CAM0193	CAM0193	CAM0193	657				CAM0193	Arthropoda	Insecta	Hymenoptera	Braconidae	Microgastrinae	Microplitis	Microplitis varicolor	Jose Leonardo Fernandez	jftriana@uoguelph.ca								H. Goulet	19-Sep-2007		Canada	Ontario	Ottawa		Ottawa city garden	45.3560982	-75.707001		
CNCHZ384-09	CAM0194	CAM0194	CAM0194	657				CAM0194	Arthropoda	Insecta	Hymenoptera	Braconidae	Microgastrinae	Microplitis	Microplitis varicolor	Jose Leonardo Fernandez	jftriana@uoguelph.ca								H. Goulet	19-Sep-2007		Canada	Ontario	Ottawa		Ottawa city garden	45.3560982	-75.707001		
CNCHZ385-09	CAM0195	CAM0195	CAM0195	657				CAM0195	Arthropoda	Insecta	Hymenoptera	Braconidae	Microgastrinae	Microplitis	Microplitis jft09	Jose Leonardo Fernandez	jftriana@uoguelph.ca								H. Goulet	19-Sep-2007		Canada	Ontario	Ottawa		Ottawa city garden	45.3560982	-75.707001		
CNCHZ386-09	CAM0196	CAM0196	CAM0196	657				CAM0196	Arthropoda	Insecta	Hymenoptera	Braconidae	Microgastrinae	Microplitis	Microplitis varicolor	Jose Leonardo Fernandez	jftriana@uoguelph.ca								H. Goulet	19-Sep-2007		Canada	Ontario	Ottawa		Ottawa city garden	45.3560982	-75.707001		
CNCHZ387-09	CAM0197	CAM0197	CAM0197	657				CAM0197	Arthropoda	Insecta	Hymenoptera	Braconidae	Microgastrinae	Microplitis	Microplitis varicolor	Jose Leonardo Fernandez	jftriana@uoguelph.ca								H. Goulet	19-Sep-2007		Canada	Ontario	Ottawa		Ottawa city garden	45.3560982	-75.707001		
CNCHZ390-09	CAM0200	CAM0200	CAM0200	657				CAM0200	Arthropoda	Insecta	Hymenoptera	Braconidae	Microgastrinae	Microplitis	Microplitis varicolor	Jose Leonardo Fernandez	jftriana@uoguelph.ca								H. Goulet	19-Sep-2007		Canada	Ontario	Ottawa		Ottawa city garden	45.3560982	-75.707001		
CNCHZ391-09	CAM0201	CAM0201	CAM0201	657				CAM0201	Arthropoda	Insecta	Hymenoptera	Braconidae	Microgastrinae	Microplitis	Microplitis varicolor	Jose Leonardo Fernandez	jftriana@uoguelph.ca								H. Goulet	19-Sep-2007		Canada	Ontario	Ottawa		Ottawa city garden	45.3560982	-75.707001		
CNCHZ392-09	CAM0202	CAM0202	CAM0202	577				CAM0202	Arthropoda	Insecta	Hymenoptera	Braconidae	Microgastrinae	Microplitis	Microplitis varicolor	Jose Leonardo Fernandez	jftriana@uoguelph.ca								H. Goulet	19-Sep-2007		Canada	Ontario	Ottawa		Ottawa city garden	45.3560982	-75.707001		
CNCHZ395-09	CAM0205	CAM0205	CAM0205	657				CAM0205	Arthropoda	Insecta	Hymenoptera	Braconidae	Microgastrinae	Microplitis	Microplitis varicolor	Jose Leonardo Fernandez	jftriana@uoguelph.ca								H. Goulet	30-May-2007		Canada	Ontario	Ottawa		Ottawa city garden	45.3560982	-75.707001		
CNCHZ396-09	CAM0206	CAM0206	CAM0206	657				CAM0206	Arthropoda	Insecta	Hymenoptera	Braconidae	Microgastrinae	Microplitis	Microplitis varicolor	Jose Leonardo Fernandez	jftriana@uoguelph.ca								H. Goulet	19-Oct-2007		Canada	Ontario	Ottawa		Ottawa city garden	45.3560982	-75.707001		
CNCHZ397-09	CAM0207	CAM0207	CAM0207	657				CAM0207	Arthropoda	Insecta	Hymenoptera	Braconidae	Microgastrinae	Microplitis	Microplitis varicolor	Jose Leonardo Fernandez	jftriana@uoguelph.ca								H. Goulet	19-Oct-2007		Canada	Ontario	Ottawa		Ottawa city garden	45.3560982	-75.707001		
CNCHZ398-09	CAM0208	CAM0208	CAM0208	657				CAM0208	Arthropoda	Insecta	Hymenoptera	Braconidae	Microgastrinae	Microplitis	Microplitis varicolor	Jose Leonardo Fernandez	jftriana@uoguelph.ca								H. Goulet	19-Oct-2007		Canada	Ontario	Ottawa		Ottawa city garden	45.3560982	-75.707001		
CNCHZ399-09	CAM0209	CAM0209	CAM0209	657				CAM0209	Arthropoda	Insecta	Hymenoptera	Braconidae	Microgastrinae	Microplitis	Microplitis varicolor	Jose Leonardo Fernandez	jftriana@uoguelph.ca								H. Goulet	19-Oct-2007		Canada	Ontario	Ottawa		Ottawa city garden	45.3560982	-75.707001		
CNCHZ401-09	CAM0211	CAM0211	CAM0211	657				CAM0211	Arthropoda	Insecta	Hymenoptera	Braconidae	Microgastrinae	Microplitis	Microplitis varicolor	Jose Leonardo Fernandez	jftriana@uoguelph.ca								H. Goulet	30-May-2007		Canada	Ontario	Ottawa		Ottawa city garden	45.3560982	-75.707001		
CNCHZ406-09	CAM0216	CAM0216	CAM0216	657				CAM0216	Arthropoda	Insecta	Hymenoptera	Braconidae	Microgastrinae	Microplitis	Microplitis varicolor	Jose Leonardo Fernandez	jftriana@uoguelph.ca								H. Goulet	30-May-2007		Canada	Ontario	Ottawa		Ottawa city garden	45.3560982	-75.707001		
CNCHZ408-09	CAM0218	CAM0218	CAM0218	657				CAM0218	Arthropoda	Insecta	Hymenoptera	Braconidae	Microgastrinae	Microplitis	Microplitis varicolor	Jose Leonardo Fernandez	jftriana@uoguelph.ca								H. Goulet	30-May-2007		Canada	Ontario	Ottawa		Ottawa city garden	45.3560982	-75.707001		
CNCHZ410-09	CAM0220	CAM0220	CAM0220	657				CAM0220	Arthropoda	Insecta	Hymenoptera	Braconidae	Microgastrinae	Microplitis	Microplitis varicolor	Jose Leonardo Fernandez	jftriana@uoguelph.ca								H. Goulet	30-May-2007		Canada	Ontario	Ottawa		Ottawa city garden	45.3560982	-75.707001		
CNCHZ412-09	CAM0222	CAM0222	CAM0222	657				CAM0222	Arthropoda	Insecta	Hymenoptera	Braconidae	Microgastrinae	Microplitis	Microplitis varicolor	Jose Leonardo Fernandez	jftriana@uoguelph.ca								H. Goulet	30-May-2007		Canada	Ontario	Ottawa		Ottawa city garden	45.3560982	-75.707001		
CNCHZ413-09	CAM0223	CAM0223	CAM0223	657				CAM0223	Arthropoda	Insecta	Hymenoptera	Braconidae	Microgastrinae	Microplitis	Microplitis varicolor	Jose Leonardo Fernandez	jftriana@uoguelph.ca								H. Goulet	30-May-2007		Canada	Ontario	Ottawa		Ottawa city garden	45.3560982	-75.707001		
CNCHZ417-09	CAM0227	CAM0227	CAM0227	657				CAM0227	Arthropoda	Insecta	Hymenoptera	Braconidae	Microgastrinae	Microplitis	Microplitis varicolor	Jose Leonardo Fernandez	jftriana@uoguelph.ca								H. Goulet	30-May-2007		Canada	Ontario	Ottawa		Ottawa city garden	45.3560982	-75.707001		
CNCHZ423-09	CAM0233	CAM0233	CAM0233	657				CAM0233	Arthropoda	Insecta	Hymenoptera	Braconidae	Microgastrinae	Microplitis	Microplitis varicolor	Jose Leonardo Fernandez	jftriana@uoguelph.ca								H. Goulet	13-Jul-2007		Canada	Ontario	Ottawa		Ottawa city garden	45.3560982	-75.707001		
CNCHZ425-09	CAM0235	CAM0235	CAM0235	646				CAM0235	Arthropoda	Insecta	Hymenoptera	Braconidae	Microgastrinae	Microplitis	Microplitis varicolor	Jose Leonardo Fernandez	jftriana@uoguelph.ca								H. Goulet	30-Jul-2007		Canada	Ontario	Ottawa		Ottawa city garden	45.3560982	-75.707001		
CNCHZ426-09	CAM0236	CAM0236	CAM0236	657				CAM0236	Arthropoda	Insecta	Hymenoptera	Braconidae	Microgastrinae	Microplitis	Microplitis varicolor	Jose Leonardo Fernandez	jftriana@uoguelph.ca								H. Goulet	30-Jul-2007		Canada	Ontario	Ottawa		Ottawa city garden	45.3560982	-75.707001		
CNCHZ427-09	CAM0237	CAM0237	CAM0237	657				CAM0237	Arthropoda	Insecta	Hymenoptera	Braconidae	Microgastrinae	Microplitis	Microplitis varicolor	Jose Leonardo Fernandez	jftriana@uoguelph.ca								H. Goulet	30-Jul-2007		Canada	Ontario	Ottawa		Ottawa city garden	45.3560982	-75.707001		
CNCHZ429-09	CAM0239	CAM0239	CAM0239	657				CAM0239	Arthropoda	Insecta	Hymenoptera	Braconidae	Microgastrinae	Microplitis	Microplitis varicolor	Jose Leonardo Fernandez	jftriana@uoguelph.ca								H. Goulet	30-Jul-2007		Canada	Ontario	Ottawa		Ottawa city garden	45.3560982	-75.707001		
CNCHZ430-09	CAM0240	CAM0240	CAM0240	657				CAM0240	Arthropoda	Insecta	Hymenoptera	Braconidae	Microgastrinae	Microplitis	Microplitis varicolor	Jose Leonardo Fernandez	jftriana@uoguelph.ca								H. Goulet	30-Jul-2007		Canada	Ontario	Ottawa		Ottawa city garden	45.3560982	-75.707001		
CNCHZ431-09	CAM0241	CAM0241	CAM0241	657				CAM0241	Arthropoda	Insecta	Hymenoptera	Braconidae	Microgastrinae	Microplitis	Microplitis varicolor	Jose Leonardo Fernandez	jftriana@uoguelph.ca								H. Goulet	30-Jul-2007		Canada	Ontario	Ottawa		Ottawa city garden	45.3560982	-75.707001		
CNCHZ433-09	CAM0243	CAM0243	CAM0243	657				CAM0243	Arthropoda	Insecta	Hymenoptera	Braconidae	Microgastrinae	Microplitis	Microplitis varicolor	Jose Leonardo Fernandez	jftriana@uoguelph.ca								H. Goulet	30-Jul-2007		Canada	Ontario	Ottawa		Ottawa city garden	45.3560982	-75.707001		
CNCHZ434-09	CAM0244	CAM0244	CAM0244	657				CAM0244	Arthropoda	Insecta	Hymenoptera	Braconidae	Microgastrinae	Microplitis	Microplitis varicolor	Jose Leonardo Fernandez	jftriana@uoguelph.ca								H. Goulet	30-Jul-2007		Canada	Ontario	Ottawa		Ottawa city garden	45.3560982	-75.707001		
CNCHZ438-09	CAM0248	CAM0248	CAM0248	657				CAM0248	Arthropoda	Insecta	Hymenoptera	Braconidae	Microgastrinae	Microplitis	Microplitis varicolor	Jose Leonardo Fernandez	jftriana@uoguelph.ca								H. Goulet	10-Aug-2007		Canada	Ontario	Ottawa		Ottawa city garden	45.3560982	-75.707001		
CNCHZ439-09	CAM0249	CAM0249	CAM0249	657				CAM0249	Arthropoda	Insecta	Hymenoptera	Braconidae	Microgastrinae	Microplitis	Microplitis varicolor	Jose Leonardo Fernandez	jftriana@uoguelph.ca								H. Goulet	16-May-2007		Canada	Ontario	Ottawa		Ottawa city garden	45.3560982	-75.707001		
CNCHZ440-09	CAM0250	CAM0250	CAM0250	657				CAM0250	Arthropoda	Insecta	Hymenoptera	Braconidae	Microgastrinae	Microplitis	Microplitis varicolor	Jose Leonardo Fernandez	jftriana@uoguelph.ca								H. Goulet	16-May-2007		Canada	Ontario	Ottawa		Ottawa city garden	45.3560982	-75.707001		
CNCHZ459-09	CAM0269	CAM0269	CAM0269	657				CAM0269	Arthropoda	Insecta	Hymenoptera	Braconidae	Microgastrinae	Protapanteles	Protapanteles jft02	Jose Leonardo Fernandez	jftriana@uoguelph.ca								H. Goulet	01-Sep-2007		Canada	Ontario	Ottawa		Ottawa city garden	45.3560982	-75.707001		
CNCHZ461-09	CAM0271	CAM0271	CAM0271	657				CAM0271	Arthropoda	Insecta	Hymenoptera	Braconidae	Microgastrinae	Protapanteles	Protapanteles jft02	Jose Leonardo Fernandez	jftriana@uoguelph.ca								H. Goulet	01-Nov-2007		Canada	Ontario	Ottawa		Ottawa city garden	45.3560982	-75.707001		
CNCHZ465-09	CAM0275	CAM0275	CAM0275	657				CAM0275	Arthropoda	Insecta	Hymenoptera	Braconidae	Microgastrinae	Protapanteles	Protapanteles jft02	Jose Leonardo Fernandez	jftriana@uoguelph.ca						NULL		H. Goulet	30-Jul-2007		Canada	Ontario	Ottawa		Ottawa city garden	45.3560982	-75.707001		
CNCHZ468-09	CAM0278	CAM0278	CAM0278	657				CAM0278	Arthropoda	Insecta	Hymenoptera	Braconidae	Microgastrinae	Pholetesor	Pholetesor bedelliae	Jose Leonardo Fernandez	jftriana@uoguelph.ca								H. Goulet	30-May-2007		Canada	Ontario	Ottawa		Ottawa city garden	45.3560982	-75.707001		
CNCHZ476-09	CAM0286	CAM0286	CAM0286	657				CAM0286	Arthropoda	Insecta	Hymenoptera	Braconidae	Microgastrinae	Apanteles	Apanteles ensiger	Jose Leonardo Fernandez	jftriana@uoguelph.ca								Goulet, Boudreault	26-Jul-2008		Canada	Quebec	Belle-Anse		Belle-Anse	48.6249008	-64.178001		
CNCHZ477-09	CAM0287	CAM0287	CAM0287	657				CAM0287	Arthropoda	Insecta	Hymenoptera	Braconidae	Microgastrinae	Dolichogenidea	Dolichogenidea jft07	Jose Leonardo Fernandez	jftriana@uoguelph.ca								Goulet, Boudreault	26-Jul-2008		Canada	Quebec	Belle-Anse		Belle-Anse	48.6249008	-64.178001		
CNCHZ478-09	CAM0288	CAM0288	CAM0288	657				CAM0288	Arthropoda	Insecta	Hymenoptera	Braconidae	Microgastrinae	Apanteles	Apanteles ensiger	Jose Leonardo Fernandez	jftriana@uoguelph.ca								Goulet, Boudreault	26-Jul-2008		Canada	Quebec	Belle-Anse		Belle-Anse	48.6249008	-64.178001		
CNCHZ479-09	CAM0289	CAM0289	CAM0289	657				CAM0289	Arthropoda	Insecta	Hymenoptera	Braconidae	Microgastrinae	Apanteles	Apanteles ensiger	Jose Leonardo Fernandez	jftriana@uoguelph.ca								Goulet, Boudreault	26-Jul-2008		Canada	Quebec	Belle-Anse		Belle-Anse	48.6249008	-64.178001		
CNCHZ480-09	CAM0290	CAM0290	CAM0290	657				CAM0290	Arthropoda	Insecta	Hymenoptera	Braconidae	Microgastrinae	Apanteles	Apanteles ensiger	Jose Leonardo Fernandez	jftriana@uoguelph.ca								Goulet, Boudreault	26-Jul-2008		Canada	Quebec	Belle-Anse		Belle-Anse	48.6249008	-64.178001		
CNCHZ482-09	CAM0292	CAM0292	CAM0292	657				CAM0292	Arthropoda	Insecta	Hymenoptera	Braconidae	Microgastrinae	Dolichogenidea	Dolichogenidea jft09	Jose Leonardo Fernandez	jftriana@uoguelph.ca								Goulet, Boudreault	26-Jul-2008		Canada	Quebec	Belle-Anse		Belle-Anse	48.6249008	-64.178001		
CNCHZ483-09	CAM0293	CAM0293	CAM0293	657				CAM0293	Arthropoda	Insecta	Hymenoptera	Braconidae	Microgastrinae	Dolichogenidea	Dolichogenidea jft09	Jose Leonardo Fernandez	jftriana@uoguelph.ca								Goulet, Boudreault	26-Jul-2008		Canada	Quebec	Belle-Anse		Belle-Anse	48.6249008	-64.178001		
CNCHZ484-09	CAM0294	CAM0294	CAM0294	657				CAM0294	Arthropoda	Insecta	Hymenoptera	Braconidae	Microgastrinae	Dolichogenidea	Dolichogenidea jft09	Jose Leonardo Fernandez	jftriana@uoguelph.ca								Goulet, Boudreault	26-Jul-2008		Canada	Quebec	Belle-Anse		Belle-Anse	48.6249008	-64.178001		
CNCHZ485-09	CAM0295	CAM0295	CAM0295	621				CAM0295	Arthropoda	Insecta	Hymenoptera	Braconidae	Microgastrinae	Dolichogenidea	Dolichogenidea jft09	Jose Leonardo Fernandez	jftriana@uoguelph.ca								Goulet, Boudreault	26-Jul-2008		Canada	Quebec	Belle-Anse		Belle-Anse	48.6249008	-64.178001		
CNCHZ486-09	CAM0296	CAM0296	CAM0296	657				CAM0296	Arthropoda	Insecta	Hymenoptera	Braconidae	Microgastrinae	Dolichogenidea	Dolichogenidea jft09	Jose Leonardo Fernandez	jftriana@uoguelph.ca								Goulet, Boudreault	26-Jul-2008		Canada	Quebec	Belle-Anse		Belle-Anse	48.6249008	-64.178001		
CNCHZ487-09	CAM0297	CAM0297	CAM0297	657				CAM0297	Arthropoda	Insecta	Hymenoptera	Braconidae	Microgastrinae	Dolichogenidea	Dolichogenidea jft09	Jose Leonardo Fernandez	jftriana@uoguelph.ca								Goulet, Boudreault	26-Jul-2008		Canada	Quebec	Belle-Anse		Belle-Anse	48.6249008	-64.178001		
CNCHZ488-09	CAM0298	CAM0298	CAM0298	657				CAM0298	Arthropoda	Insecta	Hymenoptera	Braconidae	Microgastrinae	Dolichogenidea	Dolichogenidea jft09	Jose Leonardo Fernandez	jftriana@uoguelph.ca								Goulet, Boudreault	26-Jul-2008		Canada	Quebec	Belle-Anse		Belle-Anse	48.6249008	-64.178001		
CNCHZ489-09	CAM0299	CAM0299	CAM0299	657				CAM0299	Arthropoda	Insecta	Hymenoptera	Braconidae	Microgastrinae	Dolichogenidea	Dolichogenidea jft09	Jose Leonardo Fernandez	jftriana@uoguelph.ca								Goulet, Boudreault	26-Jul-2008		Canada	Quebec	Belle-Anse		Belle-Anse	48.6249008	-64.178001		
CNCHZ511-09	CAM0321	CAM0321	CAM0321	657				CAM0321	Arthropoda	Insecta	Hymenoptera	Braconidae	Microgastrinae	Microplitis	Microplitis varicolor	Jose Leonardo Fernandez	jftriana@uoguelph.ca								Goulet & Fernandez	29-May-2008		Canada	Ontario	Almonte		5 km NW of Almonte, H	45.2549019	-76.139999		
CNCHZ525-09	CAM0335	CAM0335	CAM0335	657				CAM0335	Arthropoda	Insecta	Hymenoptera	Braconidae	Microgastrinae	Apanteles	Apanteles ensiger	Jose Leonardo Fernandez	jftriana@uoguelph.ca								Goulet & Fernandez	29-May-2008		Canada	Ontario	Almonte		5 km NW of Almonte, H	45.2549019	-76.139999		
CNCHZ533-09	CAM0343	CAM0343	CAM0343	657				CAM0343	Arthropoda	Insecta	Hymenoptera	Braconidae	Microgastrinae	Pholetesor	Pholetesor salicifoliellae	Jose Leonardo Fernandez	jftriana@uoguelph.ca								Goulet & Fernandez	29-May-2008		Canada	Ontario	Almonte		5 km NW of Almonte, H	45.2549019	-76.139999		
CNCHZ538-09	CAM0348	CAM0348	CAM0348	657				CAM0348	Arthropoda	Insecta	Hymenoptera	Braconidae	Microgastrinae	Apanteles	Apanteles ensiger	Jose Leonardo Fernandez	jftriana@uoguelph.ca								Goulet & Fernandez	29-May-2008		Canada	Ontario	Almonte		5 km NW of Almonte, H	45.2549019	-76.139999		
CNCHZ543-09	CAM0353	CAM0353	CAM0353	657				CAM0353	Arthropoda	Insecta	Hymenoptera	Braconidae	Microgastrinae	Apanteles	Apanteles jft01	Jose Leonardo Fernandez	jftriana@uoguelph.ca								Goulet & Fernandez	29-May-2008		Canada	Ontario	Almonte		5 km NW of Almonte, H	45.2549019	-76.139999		
CNCHZ545-09	CAM0355	CAM0355	CAM0355	657				CAM0355	Arthropoda	Insecta	Hymenoptera	Braconidae	Microgastrinae	Pholetesor	Pholetesor salicifoliellae	Jose Leonardo Fernandez	jftriana@uoguelph.ca								Goulet & Fernandez	29-May-2008		Canada	Ontario	Almonte		5 km NW of Almonte, H	45.2549019	-76.139999		
CNCHZ547-09	CAM0357	CAM0357	CAM0357	657				CAM0357	Arthropoda	Insecta	Hymenoptera	Braconidae	Microgastrinae	Apanteles	Apanteles jft01	Jose Leonardo Fernandez	jftriana@uoguelph.ca								Goulet & Fernandez	29-May-2008		Canada	Ontario	Almonte		5 km NW of Almonte, H	45.2549019	-76.139999		
CNCHZ551-09	CAM0361	CAM0361	CAM0361	657				CAM0361	Arthropoda	Insecta	Hymenoptera	Braconidae	Microgastrinae	Apanteles	Apanteles jft01	Jose Leonardo Fernandez	jftriana@uoguelph.ca								Goulet & Fernandez	29-May-2008		Canada	Ontario	Almonte		5 km NW of Almonte, H	45.2549019	-76.139999		
CNCHZ552-09	CAM0362	CAM0362	CAM0362	632				CAM0362	Arthropoda	Insecta	Hymenoptera	Braconidae	Microgastrinae	Pholetesor	Pholetesor salicifoliellae	Jose Leonardo Fernandez	jftriana@uoguelph.ca								Goulet & Fernandez	29-May-2008		Canada	Ontario	Almonte		5 km NW of Almonte, H	45.2549019	-76.139999		
CNCHZ554-09	CAM0364	CAM0364	CAM0364	657				CAM0364	Arthropoda	Insecta	Hymenoptera	Braconidae	Microgastrinae	Apanteles	Apanteles jft01	Jose Leonardo Fernandez	jftriana@uoguelph.ca								Goulet & Fernandez	29-May-2008		Canada	Ontario	Almonte		5 km NW of Almonte, H	45.2549019	-76.139999		
CNCHZ555-09	CAM0365	CAM0365	CAM0365	657				CAM0365	Arthropoda	Insecta	Hymenoptera	Braconidae	Microgastrinae	Pholetesor	Pholetesor viminetorum	Jose Leonardo Fernandez	jftriana@uoguelph.ca								Goulet & Fernandez	29-May-2008		Canada	Ontario	Almonte		5 km NW of Almonte, H	45.2549019	-76.139999		
CNCHZ572-09	CAM0382	CAM0382	CAM0382	657				CAM0382	Arthropoda	Insecta	Hymenoptera	Braconidae	Microgastrinae	Pholetesor	Pholetesor viminetorum	Jose Leonardo Fernandez	jftriana@uoguelph.ca								Boudreault, Goulet	03-Jun-2008		Canada	Ontario	Manitoulin Island		Misery Bay Prov. Park	45.7919006	-82.75	185	
CNCHZ573-09	CAM0383	CAM0383	CAM0383	657				CAM0383	Arthropoda	Insecta	Hymenoptera	Braconidae	Microgastrinae	Pholetesor	Pholetesor viminetorum	Jose Leonardo Fernandez	jftriana@uoguelph.ca								Boudreault, Goulet	03-Jun-2008		Canada	Ontario	Manitoulin Island		Misery Bay Prov. Park	45.7919006	-82.75	185	
CNCHZ574-09	CAM0384	CAM0384	CAM0384	657				CAM0384	Arthropoda	Insecta	Hymenoptera	Braconidae	Microgastrinae	Pholetesor	Pholetesor viminetorum	Jose Leonardo Fernandez	jftriana@uoguelph.ca								Goulet, Boudreault	04-Jun-2008		Canada	Ontario	Manitoulin Island		Little Current prairie Alv	45.9752998	-81.903999		
CNCHZ575-09	CAM0385	CAM0385	CAM0385	657				CAM0385	Arthropoda	Insecta	Hymenoptera	Braconidae	Microgastrinae	Pholetesor	Pholetesor viminetorum	Jose Leonardo Fernandez	jftriana@uoguelph.ca								Goulet, Boudreault	04-Jun-2008		Canada	Ontario	Manitoulin Island		Little Current prairie Alv	45.9752998	-81.903999		
CNCHZ576-09	CAM0386	CAM0386	CAM0386	657				CAM0386	Arthropoda	Insecta	Hymenoptera	Braconidae	Microgastrinae	Pholetesor	Pholetesor viminetorum	Jose Leonardo Fernandez	jftriana@uoguelph.ca								Goulet, Boudreault	04-Jun-2008		Canada	Ontario	Manitoulin Island		Little Current prairie Alv	45.9752998	-81.903999		
CNCHZ577-09	CAM0387	CAM0387	CAM0387	657				CAM0387	Arthropoda	Insecta	Hymenoptera	Braconidae	Microgastrinae	Pholetesor	Pholetesor viminetorum	Jose Leonardo Fernandez	jftriana@uoguelph.ca								Goulet, Boudreault	04-Jun-2008		Canada	Ontario	Manitoulin Island		Little Current prairie Alv	45.9752998	-81.903999		
CNCHZ593-09	CAM0403	CAM0403	CAM0403	657				CAM0403	Arthropoda	Insecta	Hymenoptera	Braconidae	Microgastrinae	Pholetesor	Pholetesor viminetorum	Jose Leonardo Fernandez	jftriana@uoguelph.ca								Boudreault, Goulet	03-Jun-2008		Canada	Ontario	Manitoulin Island		Misery Bay Prov. Park	45.7919006	-82.75	185	
CNCHZ602-09	CAM0412	CAM0412	CAM0412	657				CAM0412	Arthropoda	Insecta	Hymenoptera	Braconidae	Microgastrinae	Pholetesor	Pholetesor viminetorum	Jose Leonardo Fernandez	jftriana@uoguelph.ca								Goulet, Boudreault	04-Jun-2008		Canada	Ontario	Manitoulin Island		Little Current prairie Alv	45.9752998	-81.903999		
CNCHZ604-09	CAM0414	CAM0414	CAM0414	657				CAM0414	Arthropoda	Insecta	Hymenoptera	Braconidae	Microgastrinae	Pholetesor	Pholetesor viminetorum	Jose Leonardo Fernandez	jftriana@uoguelph.ca								Goulet & Fernandez	29-May-2008		Canada	Ontario	Almonte		5 km NW of Almonte, H	45.2549019	-76.139999		
CNCHZ605-09	CAM0415	CAM0415	CAM0415	657				CAM0415	Arthropoda	Insecta	Hymenoptera	Braconidae	Microgastrinae	Pholetesor	Pholetesor viminetorum	Jose Leonardo Fernandez	jftriana@uoguelph.ca								Goulet & Fernandez	29-May-2008		Canada	Ontario	Almonte		5 km NW of Almonte, H	45.2549019	-76.139999		
CNCHZ606-09	CAM0416	CAM0416	CAM0416	657				CAM0416	Arthropoda	Insecta	Hymenoptera	Braconidae	Microgastrinae	Pholetesor	Pholetesor viminetorum	Jose Leonardo Fernandez	jftriana@uoguelph.ca								Goulet & Fernandez	29-May-2008		Canada	Ontario	Almonte		5 km NW of Almonte, H	45.2549019	-76.139999		
CNCHZ607-09	CAM0417	CAM0417	CAM0417	657				CAM0417	Arthropoda	Insecta	Hymenoptera	Braconidae	Microgastrinae	Pholetesor	Pholetesor viminetorum	Jose Leonardo Fernandez	jftriana@uoguelph.ca								Goulet & Fernandez	29-May-2008		Canada	Ontario	Almonte		5 km NW of Almonte, H	45.2549019	-76.139999		
CNCHZ608-09	CAM0418	CAM0418	CAM0418	657				CAM0418	Arthropoda	Insecta	Hymenoptera	Braconidae	Microgastrinae	Pholetesor	Pholetesor viminetorum	Jose Leonardo Fernandez	jftriana@uoguelph.ca								Goulet & Fernandez	29-May-2008		Canada	Ontario	Almonte		5 km NW of Almonte, H	45.2549019	-76.139999		
CNCHZ609-09	CAM0419	CAM0419	CAM0419	657				CAM0419	Arthropoda	Insecta	Hymenoptera	Braconidae	Microgastrinae	Pholetesor	Pholetesor viminetorum	Jose Leonardo Fernandez	jftriana@uoguelph.ca								Goulet & Fernandez	29-May-2008		Canada	Ontario	Almonte		5 km NW of Almonte, H	45.2549019	-76.139999		
CNCHZ610-09	CAM0420	CAM0420	CAM0420	576				CAM0420	Arthropoda	Insecta	Hymenoptera	Braconidae	Microgastrinae	Pholetesor	Pholetesor salicifoliellae	Jose Leonardo Fernandez	jftriana@uoguelph.ca								Goulet & Fernandez	29-May-2008		Canada	Ontario	Almonte		5 km NW of Almonte, H	45.2549019	-76.139999		
CNCHZ611-09	CAM0421	CAM0421	CAM0421	657				CAM0421	Arthropoda	Insecta	Hymenoptera	Braconidae	Microgastrinae	Pholetesor	Pholetesor viminetorum	Jose Leonardo Fernandez	jftriana@uoguelph.ca								Goulet & Fernandez	29-May-2008		Canada	Ontario	Almonte		5 km NW of Almonte, H	45.2549019	-76.139999		
CNCHZ612-09	CAM0422	CAM0422	CAM0422	657				CAM0422	Arthropoda	Insecta	Hymenoptera	Braconidae	Microgastrinae	Pholetesor	Pholetesor viminetorum	Jose Leonardo Fernandez	jftriana@uoguelph.ca								Goulet & Fernandez	29-May-2008		Canada	Ontario	Almonte		5 km NW of Almonte, H	45.2549019	-76.139999		
CNCHZ613-09	CAM0423	CAM0423	CAM0423	657				CAM0423	Arthropoda	Insecta	Hymenoptera	Braconidae	Microgastrinae	Pholetesor	Pholetesor viminetorum	Jose Leonardo Fernandez	jftriana@uoguelph.ca								Goulet & Fernandez	29-May-2008		Canada	Ontario	Almonte		5 km NW of Almonte, H	45.2549019	-76.139999		
CNCHZ614-09	CAM0424	CAM0424	CAM0424	548				CAM0424	Arthropoda	Insecta	Hymenoptera	Braconidae	Microgastrinae	Pholetesor	Pholetesor ornigis	Jose Leonardo Fernandez	jftriana@uoguelph.ca								Goulet & Fernandez	29-May-2008		Canada	Ontario	Almonte		5 km NW of Almonte, H	45.2549019	-76.139999		
CNCHZ615-09	CAM0425	CAM0425	CAM0425	618				CAM0425	Arthropoda	Insecta	Hymenoptera	Braconidae	Microgastrinae	Pholetesor	Pholetesor salicifoliellae	Jose Leonardo Fernandez	jftriana@uoguelph.ca								Goulet & Fernandez	29-May-2008		Canada	Ontario	Almonte		5 km NW of Almonte, H	45.2549019	-76.139999		
CNCHZ616-09	CAM0426	CAM0426	CAM0426	657				CAM0426	Arthropoda	Insecta	Hymenoptera	Braconidae	Microgastrinae	Pholetesor	Pholetesor viminetorum	Jose Leonardo Fernandez	jftriana@uoguelph.ca								Goulet & Fernandez	29-May-2008		Canada	Ontario	Almonte		5 km NW of Almonte, H	45.2549019	-76.139999		
CNCHZ617-09	CAM0427	CAM0427	CAM0427	657				CAM0427	Arthropoda	Insecta	Hymenoptera	Braconidae	Microgastrinae	Pholetesor	Pholetesor viminetorum	Jose Leonardo Fernandez	jftriana@uoguelph.ca								Goulet & Fernandez	29-May-2008		Canada	Ontario	Almonte		5 km NW of Almonte, H	45.2549019	-76.139999		
CNCHZ618-09	CAM0428	CAM0428	CAM0428	657				CAM0428	Arthropoda	Insecta	Hymenoptera	Braconidae	Microgastrinae	Pholetesor	Pholetesor viminetorum	Jose Leonardo Fernandez	jftriana@uoguelph.ca								Goulet & Fernandez	29-May-2008		Canada	Ontario	Almonte		5 km NW of Almonte, H	45.2549019	-76.139999		
CNCHZ619-09	CAM0429	CAM0429	CAM0429	657				CAM0429	Arthropoda	Insecta	Hymenoptera	Braconidae	Microgastrinae	Pholetesor	Pholetesor viminetorum	Jose Leonardo Fernandez	jftriana@uoguelph.ca								Goulet & Fernandez	29-May-2008		Canada	Ontario	Almonte		5 km NW of Almonte, H	45.2549019	-76.139999		
CNCHZ620-09	CAM0430	CAM0430	CAM0430	657				CAM0430	Arthropoda	Insecta	Hymenoptera	Braconidae	Microgastrinae	Pholetesor	Pholetesor viminetorum	Jose Leonardo Fernandez	jftriana@uoguelph.ca								Goulet & Fernandez	29-May-2008		Canada	Ontario	Almonte		5 km NW of Almonte, H	45.2549019	-76.139999		
CNCHZ621-09	CAM0431	CAM0431	CAM0431	657				CAM0431	Arthropoda	Insecta	Hymenoptera	Braconidae	Microgastrinae	Pholetesor	Pholetesor viminetorum	Jose Leonardo Fernandez	jftriana@uoguelph.ca								Goulet & Fernandez	29-May-2008		Canada	Ontario	Almonte		5 km NW of Almonte, H	45.2549019	-76.139999		
CNCHZ622-09	CAM0432	CAM0432	CAM0432	657				CAM0432	Arthropoda	Insecta	Hymenoptera	Braconidae	Microgastrinae	Pholetesor	Pholetesor viminetorum	Jose Leonardo Fernandez	jftriana@uoguelph.ca								Goulet & Fernandez	29-May-2008		Canada	Ontario	Almonte		5 km NW of Almonte, H	45.2549019	-76.139999		
CNCHZ623-09	CAM0433	CAM0433	CAM0433	606				CAM0433	Arthropoda	Insecta	Hymenoptera	Braconidae	Microgastrinae	Pholetesor	Pholetesor ornigis	Jose Leonardo Fernandez	jftriana@uoguelph.ca								Goulet & Fernandez	29-May-2008		Canada	Ontario	Almonte		5 km NW of Almonte, H	45.2549019	-76.139999		
CNCHZ624-09	CAM0434	CAM0434	CAM0434	613				CAM0434	Arthropoda	Insecta	Hymenoptera	Braconidae	Microgastrinae	Pholetesor	Pholetesor viminetorum	Jose Leonardo Fernandez	jftriana@uoguelph.ca								Goulet & Fernandez	29-May-2008		Canada	Ontario	Almonte		5 km NW of Almonte, H	45.2549019	-76.139999		
CNCHZ625-09	CAM0435	CAM0435	CAM0435	657				CAM0435	Arthropoda	Insecta	Hymenoptera	Braconidae	Microgastrinae	Pholetesor	Pholetesor viminetorum	Jose Leonardo Fernandez	jftriana@uoguelph.ca								Goulet & Fernandez	29-May-2008		Canada	Ontario	Almonte		5 km NW of Almonte, H	45.2549019	-76.139999		
CNCHZ627-09	CAM0437	CAM0437	CAM0437	657				CAM0437	Arthropoda	Insecta	Hymenoptera	Braconidae	Microgastrinae	Pholetesor	Pholetesor bedelliae	Jose Leonardo Fernandez	jftriana@uoguelph.ca								Goulet & Fernandez	29-May-2008		Canada	Ontario	Almonte		5 km NW of Almonte, H	45.2549019	-76.139999		
CNCHZ628-09	CAM0438	CAM0438	CAM0438	657				CAM0438	Arthropoda	Insecta	Hymenoptera	Braconidae	Microgastrinae	Pholetesor	Pholetesor viminetorum	Jose Leonardo Fernandez	jftriana@uoguelph.ca								Goulet & Fernandez	29-May-2008		Canada	Ontario	Almonte		5 km NW of Almonte, H	45.2549019	-76.139999		
CNCHZ635-09	CAM0445	CAM0445	CAM0445	657				CAM0445	Arthropoda	Insecta	Hymenoptera	Braconidae	Microgastrinae	Apanteles	Apanteles ensiger	Jose Leonardo Fernandez	jftriana@uoguelph.ca								S. Peck	01-Oct-2008		Canada	Ontario	Leeds		Leeds-Granville Co., fo	44.6287994	-76.359001		
CNCHZ636-09	CAM0446	CAM0446	CAM0446	657				CAM0446	Arthropoda	Insecta	Hymenoptera	Braconidae	Microgastrinae	Apanteles	Apanteles ensiger	Jose Leonardo Fernandez	jftriana@uoguelph.ca								S. Peck	01-Oct-2008		Canada	Ontario	Leeds		Leeds-Granville Co., fo	44.6287994	-76.359001		
CNCHZ637-09	CAM0447	CAM0447	CAM0447	657				CAM0447	Arthropoda	Insecta	Hymenoptera	Braconidae	Microgastrinae	Apanteles	Apanteles ensiger	Jose Leonardo Fernandez	jftriana@uoguelph.ca								S. Peck	01-Oct-2008		Canada	Ontario	Leeds		Leeds-Granville Co., fo	44.6287994	-76.359001		
CNCHZ638-09	CAM0448	CAM0448	CAM0448	657				CAM0448	Arthropoda	Insecta	Hymenoptera	Braconidae	Microgastrinae	Apanteles	Apanteles ensiger	Jose Leonardo Fernandez	jftriana@uoguelph.ca								S. Peck	01-Oct-2008		Canada	Ontario	Leeds		Leeds-Granville Co., fo	44.6287994	-76.359001		
CNCHZ791-09	MIC 000599	MIC 000599	MIC 000599	657				MIC 000599	Arthropoda	Insecta	Hymenoptera	Braconidae	Microgastrinae	Apanteles	Apanteles jft01	Jose Leonardo Fernandez	jftriana@uoguelph.ca								L. Masner	06-Aug-2008		Canada	Ontario			Woodlawn				
CNCHZ793-09	MIC 000601	MIC 000601	MIC 000601	657				MIC 000601	Arthropoda	Insecta	Hymenoptera	Braconidae	Microgastrinae	Apanteles	Apanteles jft01	Jose Leonardo Fernandez	jftriana@uoguelph.ca								L. Masner	06-Aug-2008		Canada	Ontario			Woodlawn				
CNCHZ812-09	MIC 000620	MIC 000620	MIC 000620	657				MIC 000620	Arthropoda	Insecta	Hymenoptera	Braconidae	Microgastrinae	Microplitis	Microplitis varicolor	Jose Leonardo Fernandez	jftriana@uoguelph.ca								L. Masner	06-Aug-2008		Canada	Ontario			Woodlawn				
CNCHZ837-09	MIC 000645	MIC 000645	MIC 000645	632				MIC 000645	Arthropoda	Insecta	Hymenoptera	Braconidae	Microgastrinae	Microplitis	Microplitis jft07	Jose Leonardo Fernandez	jftriana@uoguelph.ca								Goulet, Boudreault	26-Jul-2008		Canada	Quebec	Cloridorme		Cloridorme, fallow field	49.1869011	-64.853996		
CNCHZ838-09	MIC 000646	MIC 000646	MIC 000646	657				MIC 000646	Arthropoda	Insecta	Hymenoptera	Braconidae	Microgastrinae	Microplitis	Microplitis jft07	Jose Leonardo Fernandez	jftriana@uoguelph.ca								Goulet, Boudreault	26-Jul-2008		Canada	Quebec	Cloridorme		Cloridorme, fallow field	49.1869011	-64.853996		
CNCHZ839-09	MIC 000647	MIC 000647	MIC 000647	657				MIC 000647	Arthropoda	Insecta	Hymenoptera	Braconidae	Microgastrinae	Microplitis	Microplitis jft07	Jose Leonardo Fernandez	jftriana@uoguelph.ca								Goulet, Boudreault	26-Jul-2008		Canada	Quebec	Cloridorme		Cloridorme, fallow field	49.1869011	-64.853996		
CNCHZ841-09	MIC 000649	MIC 000649	MIC 000649	657				MIC 000649	Arthropoda	Insecta	Hymenoptera	Braconidae	Microgastrinae	Dolichogenidea	Dolichogenidea jft02	Jose Leonardo Fernandez	jftriana@uoguelph.ca								Goulet, Boudreault	26-Jul-2008		Canada	Quebec	Cloridorme		Cloridorme, fallow field	49.1869011	-64.853996		
DSWAS071-06	06-PROBE-1477	06-PROBE-1477	06-PROBE-1477	0		0		06-PROBE-1477	Arthropoda	Insecta	Hymenoptera	Braconidae	Microgastrinae	Microplitis	Microplitis varicolor	Jose Leonardo Fernandez	jftriana@uoguelph.ca					<VERBATIM LOCALITY>|Burn Site|58.618|-93.8			J.Sones	23-Aug-2006		Canada	Manitoba	Churchill	26 km SE Churchill, Tw		58.618	-93.829002		
DSWAS736-07	06-PROBE-3435	06-PROBE-3435	06-PROBE-3435	302	GU141268			06-PROBE-3435	Arthropoda	Insecta	Hymenoptera	Braconidae	Microgastrinae	Microplitis	Microplitis jft04	Jose Leonardo Fernandez	jftriana@uoguelph.ca					<VERBATIM LOCALITY>|Churchill|58.769|-94.1						Canada	Manitoba	Churchill	unspecified locality		58.7400017	-93.82		
DSWAS744-07	HYM00000801	HYM00000801	HYM00000801	267	GU141376			HYM00000801	Arthropoda	Insecta	Hymenoptera	Braconidae	Microgastrinae	Pholetesor	Pholetesor viminetorum	Jose Leonardo Fernandez	jftriana@uoguelph.ca					<VERBATIM LOCALITY>|Fort Churchill|||</VER			J.G.Chillcot	07-Aug-1952		Canada	Manitoba	Churchill	23 km E Churchill, Fort		58.7340012	-93.789001	27	
DSWAS745-07	HYM00000802	HYM00000802	HYM00000802	0		0		HYM00000802	Arthropoda	Insecta	Hymenoptera	Braconidae	Microgastrinae	Pholetesor	Pholetesor viminetorum	Jose Leonardo Fernandez	jftriana@uoguelph.ca					<VERBATIM LOCALITY>|Fort Churchill|||</VER			J.G.Chillcot	04-Aug-1952		Canada	Manitoba	Churchill	23 km E Churchill, Fort		58.7340012	-93.789001	27	
DSWAS746-07	HYM00000803	HYM00000803	HYM00000803	0		0		HYM00000803	Arthropoda	Insecta	Hymenoptera	Braconidae	Microgastrinae	Pholetesor	Pholetesor viminetorum	Jose Leonardo Fernandez	jftriana@uoguelph.ca					<VERBATIM LOCALITY>|Fort Churchill|||</VER			J.G.Chillcot	11-Jul-1952		Canada	Manitoba	Churchill	23 km E Churchill, Fort		58.7340012	-93.789001	27	
DSWAS747-07	HYM00000804	HYM00000804	HYM00000804	0		0		HYM00000804	Arthropoda	Insecta	Hymenoptera	Braconidae	Microgastrinae	Pholetesor	Pholetesor viminetorum	Jose Leonardo Fernandez	jftriana@uoguelph.ca					<VERBATIM LOCALITY>|Fort Churchill|||</VER			J.G.Chillcot	21-Jul-1952		Canada	Manitoba	Churchill	23 km E Churchill, Fort		58.7340012	-93.789001	27	
DSWAS748-07	HYM00000805	HYM00000805	HYM00000805	0		0		HYM00000805	Arthropoda	Insecta	Hymenoptera	Braconidae	Microgastrinae	Pholetesor	Pholetesor viminetorum	Jose Leonardo Fernandez	jftriana@uoguelph.ca					<VERBATIM LOCALITY>|Fort Churchill|||</VER			J.G.Chillcot	16-Jul-1952		Canada	Manitoba	Churchill	23 km E Churchill, Fort		58.7340012	-93.789001	27	
DSWAS749-07	HYM00000806	HYM00000806	HYM00000806	0		0		HYM00000806	Arthropoda	Insecta	Hymenoptera	Braconidae	Microgastrinae	Pholetesor	Pholetesor viminetorum	Jose Leonardo Fernandez	jftriana@uoguelph.ca					<VERBATIM LOCALITY>|Fort Churchill|||</VER			J.G.Chillcot	18-Jul-1952		Canada	Manitoba	Churchill	23 km E Churchill, Fort		58.7340012	-93.789001	27	
DSWAS750-07	HYM00000807	HYM00000807	HYM00000807	0		0		HYM00000807	Arthropoda	Insecta	Hymenoptera	Braconidae	Microgastrinae	Pholetesor	Pholetesor viminetorum	Jose Leonardo Fernandez	jftriana@uoguelph.ca					<VERBATIM LOCALITY>|Fort Churchill|||</VER			J.G.Chillcot	23-Jul-1952		Canada	Manitoba	Churchill	23 km E Churchill, Fort		58.7340012	-93.789001	27	
DSWAS751-07	HYM00000808	HYM00000808	HYM00000808	0		0		HYM00000808	Arthropoda	Insecta	Hymenoptera	Braconidae	Microgastrinae	Pholetesor	Pholetesor viminetorum	Jose Leonardo Fernandez	jftriana@uoguelph.ca					<VERBATIM LOCALITY>|Fort Churchill|||</VER			J.G.Chillcot	18-Jul-1952		Canada	Manitoba	Churchill	23 km E Churchill, Fort		58.7340012	-93.789001	27	
DSWAS760-07	HYM00000817	HYM00000817	HYM00000817	0		0		HYM00000817	Arthropoda	Insecta	Hymenoptera	Braconidae	Microgastrinae	Pholetesor	Pholetesor viminetorum	Jose Leonardo Fernandez	jftriana@uoguelph.ca					<VERBATIM LOCALITY>|Fort Churchill|||</VER			J.G.Chillcot	09-Jul-1952		Canada	Manitoba	Churchill	23 km E Churchill, Fort		58.7340012	-93.789001	27	
DSWAS761-07	HYM00000818	HYM00000818	HYM00000818	293	GU141335			HYM00000818	Arthropoda	Insecta	Hymenoptera	Braconidae	Microgastrinae	Pholetesor	Pholetesor bedelliae	Jose Leonardo Fernandez	jftriana@uoguelph.ca					<VERBATIM LOCALITY>|Burn Site|58.371|-93.4				19-Aug-2006		Canada	Manitoba	Churchill	26 km SE Churchill, Tw		58.6170006	-93.811996	38	
DSWAS762-07	HYM00000819	HYM00000819	HYM00000819	283	GU141334			HYM00000819	Arthropoda	Insecta	Hymenoptera	Braconidae	Microgastrinae	Pholetesor	Pholetesor bedelliae	Jose Leonardo Fernandez	jftriana@uoguelph.ca					<VERBATIM LOCALITY>|Burn Site|58.371|-93.4				19-Aug-2006		Canada	Manitoba	Churchill	26 km SE Churchill, Tw		58.6170006	-93.811996	38	
DSWAS766-07	HYM00000823	HYM00000823	HYM00000823	293	GU141267			HYM00000823	Arthropoda	Insecta	Hymenoptera	Braconidae	Microgastrinae	Microplitis	Microplitis jft04	Jose Leonardo Fernandez	jftriana@uoguelph.ca					<VERBATIM LOCALITY>|Burn Site|58.371|-93.4				19-Aug-2006		Canada	Manitoba	Churchill	26 km SE Churchill, Tw		58.6170006	-93.811996	38	
DSWAS767-07	HYM00000824	HYM00000824	HYM00000824	293	GU141266			HYM00000824	Arthropoda	Insecta	Hymenoptera	Braconidae	Microgastrinae	Microplitis	Microplitis jft04	Jose Leonardo Fernandez	jftriana@uoguelph.ca					<VERBATIM LOCALITY>|Burn Site|58.371|-93.4				17-Aug-2006		Canada	Manitoba	Churchill	26 km SE Churchill, Tw		58.6170006	-93.811996	38	

Table S5: All collection and accession information associated with specimens analysed here.

ProcessID	Sample ID	Field Num	Catalog Num	COI-5P Seq.	COI GenBank	28S Seq. Length	28S GenBan	Sample ID	Phylum	Class	Order	Family	Subfamily	Genus
DSWAS768-07	HYM00000825	HYM00000825	HYM00000825	288	GU141265			HYM00000825	Arthropoda	Insecta	Hymenoptera	Braconidae	Microgastrinae	Microplitis
DSWAS769-07	HYM00000826	HYM00000826	HYM00000826	290	GU141264			HYM00000826	Arthropoda	Insecta	Hymenoptera	Braconidae	Microgastrinae	Microplitis
DSWAS770-07	HYM00000827	HYM00000827	HYM00000827	290	GU141263			HYM00000827	Arthropoda	Insecta	Hymenoptera	Braconidae	Microgastrinae	Microplitis
DSWAS771-07	HYM00000828	HYM00000828	HYM00000828	288	GU141262			HYM00000828	Arthropoda	Insecta	Hymenoptera	Braconidae	Microgastrinae	Microplitis
DSWAS772-07	HYM00000829	HYM00000829	HYM00000829	290	GU141261			HYM00000829	Arthropoda	Insecta	Hymenoptera	Braconidae	Microgastrinae	Microplitis
DSWAS773-07	HYM00000830	HYM00000830	HYM00000830	293	GU141260			HYM00000830	Arthropoda	Insecta	Hymenoptera	Braconidae	Microgastrinae	Microplitis
DSWAS778-07	HYM00000835	HYM00000835	HYM00000835	281	GU141307			HYM00000835	Arthropoda	Insecta	Hymenoptera	Braconidae	Microgastrinae	Microplitis
DSWAS779-07	HYM00000836	HYM00000836	HYM00000836	293	GU141306			HYM00000836	Arthropoda	Insecta	Hymenoptera	Braconidae	Microgastrinae	Microplitis
DSWAS782-07	HYM00000839	HYM00000839	HYM00000839	384	GU141305			HYM00000839	Arthropoda	Insecta	Hymenoptera	Braconidae	Microgastrinae	Microplitis
DSWAS805-07	HYM00000862	HYM00000862	HYM00000862	291	GU141206			HYM00000862	Arthropoda	Insecta	Hymenoptera	Braconidae	Microgastrinae	Glyptapanteles
DSWAS806-07	HYM00000863	HYM00000863	HYM00000863	290	GU141205			HYM00000863	Arthropoda	Insecta	Hymenoptera	Braconidae	Microgastrinae	Glyptapanteles
DSWAS807-07	HYM00000864	HYM00000864	HYM00000864	290	GU141204			HYM00000864	Arthropoda	Insecta	Hymenoptera	Braconidae	Microgastrinae	Glyptapanteles
DSWAS811-07	HYM00000868	HYM00000868	HYM00000868	594	GU141187			HYM00000868	Arthropoda	Insecta	Hymenoptera	Braconidae	Microgastrinae	Glyptapanteles
DSWAS813-07	HYM00000870	HYM00000870	HYM00000870	589	GU141186			HYM00000870	Arthropoda	Insecta	Hymenoptera	Braconidae	Microgastrinae	Glyptapanteles
DSWAS815-07	HYM00000872	HYM00000872	HYM00000872	590	GU141185			HYM00000872	Arthropoda	Insecta	Hymenoptera	Braconidae	Microgastrinae	Glyptapanteles
DSWAS816-07	HYM00000873	HYM00000873	HYM00000873	293	GU141203			HYM00000873	Arthropoda	Insecta	Hymenoptera	Braconidae	Microgastrinae	Glyptapanteles
DSWAS817-07	HYM00000874	HYM00000874	HYM00000874	293	GU141202			HYM00000874	Arthropoda	Insecta	Hymenoptera	Braconidae	Microgastrinae	Glyptapanteles
DSWAS818-07	HYM00000875	HYM00000875	HYM00000875	285	GU141201			HYM00000875	Arthropoda	Insecta	Hymenoptera	Braconidae	Microgastrinae	Glyptapanteles
DSWAS819-07	HYM00000876	HYM00000876	HYM00000876	594	GU141184			HYM00000876	Arthropoda	Insecta	Hymenoptera	Braconidae	Microgastrinae	Glyptapanteles
DSWAS820-07	HYM00000877	HYM00000877	HYM00000877	574	GU141183			HYM00000877	Arthropoda	Insecta	Hymenoptera	Braconidae	Microgastrinae	Glyptapanteles
DSWAS821-07	HYM00000878	HYM00000878	HYM00000878	592	GU141182			HYM00000878	Arthropoda	Insecta	Hymenoptera	Braconidae	Microgastrinae	Glyptapanteles
DSWAS822-07	HYM00000879	HYM00000879	HYM00000879	293	GU141200			HYM00000879	Arthropoda	Insecta	Hymenoptera	Braconidae	Microgastrinae	Glyptapanteles
DSWAS823-07	HYM00000880	HYM00000880	HYM00000880	594	GU141181			HYM00000880	Arthropoda	Insecta	Hymenoptera	Braconidae	Microgastrinae	Glyptapanteles
DSWAS824-07	HYM00000881	HYM00000881	HYM00000881	290	GU141199			HYM00000881	Arthropoda	Insecta	Hymenoptera	Braconidae	Microgastrinae	Glyptapanteles
DSWAS825-07	HYM00000882	HYM00000882	HYM00000882	290	GU141198			HYM00000882	Arthropoda	Insecta	Hymenoptera	Braconidae	Microgastrinae	Glyptapanteles
DSWAS826-07	HYM00000883	HYM00000883	HYM00000883	589	GU141180			HYM00000883	Arthropoda	Insecta	Hymenoptera	Braconidae	Microgastrinae	Glyptapanteles
DSWAS827-07	HYM00000884	HYM00000884	HYM00000884	594	GU141179			HYM00000884	Arthropoda	Insecta	Hymenoptera	Braconidae	Microgastrinae	Glyptapanteles
DSWAS828-07	HYM00000885	HYM00000885	HYM00000885	573	GU141178			HYM00000885	Arthropoda	Insecta	Hymenoptera	Braconidae	Microgastrinae	Glyptapanteles
DSWAS829-07	HYM00000886	HYM00000886	HYM00000886	290	GU141197			HYM00000886	Arthropoda	Insecta	Hymenoptera	Braconidae	Microgastrinae	Glyptapanteles
DSWAS830-07	HYM00000887	HYM00000887	HYM00000887	574	GU141177			HYM00000887	Arthropoda	Insecta	Hymenoptera	Braconidae	Microgastrinae	Glyptapanteles
DSWAS841-07	HYM00000898	HYM00000898	HYM00000898	292	GU141225			HYM00000898	Arthropoda	Insecta	Hymenoptera	Braconidae	Microgastrinae	Illidops
DSWAS843-07	HYM00000900	HYM00000900	HYM00000900	0		0		HYM00000900	Arthropoda	Insecta	Hymenoptera	Braconidae	Microgastrinae	Dolichogenidea
DSWAS848-07	HYM00000905	HYM00000905	HYM00000905	290	GU141145			HYM00000905	Arthropoda	Insecta	Hymenoptera	Braconidae	Microgastrinae	Dolichogenidea
DSWAS854-07	HYM00000911	HYM00000911	HYM00000911	291	GU141224			HYM00000911	Arthropoda	Insecta	Hymenoptera	Braconidae	Microgastrinae	Illidops
DSWAS857-07	HYM00000914	HYM00000914	HYM00000914	283	GU141229			HYM00000914	Arthropoda	Insecta	Hymenoptera	Braconidae	Microgastrinae	Microgaster
DSWAS858-07	HYM00000915	HYM00000915	HYM00000915	275	GU141228			HYM00000915	Arthropoda	Insecta	Hymenoptera	Braconidae	Microgastrinae	Microgaster
DSWAS859-07	HYM00000916	HYM00000916	HYM00000916	288	GU141227			HYM00000916	Arthropoda	Insecta	Hymenoptera	Braconidae	Microgastrinae	Microgaster
DSWAS863-07	HYM00000920	HYM00000920	HYM00000920	596	GU141333			HYM00000920	Arthropoda	Insecta	Hymenoptera	Braconidae	Microgastrinae	Pholetesor
DSWAS865-07	HYM00000922	HYM00000922	HYM00000922	0		0		HYM00000922	Arthropoda	Insecta	Hymenoptera	Braconidae	Microgastrinae	Pholetesor
DSWAS866-07	HYM00000923	HYM00000923	HYM00000923	283	GU141248			HYM00000923	Arthropoda	Insecta	Hymenoptera	Braconidae	Microgastrinae	Microplitis
DSWAS867-07	HYM00000924	HYM00000924	HYM00000924	284	GU141254			HYM00000924	Arthropoda	Insecta	Hymenoptera	Braconidae	Microgastrinae	Microplitis
DSWAS868-07	HYM00000925	HYM00000925	HYM00000925	393	GU141259			HYM00000925	Arthropoda	Insecta	Hymenoptera	Braconidae	Microgastrinae	Microplitis
DSWAS869-07	HYM00000926	HYM00000926	HYM00000926	617	GU141258			HYM00000926	Arthropoda	Insecta	Hymenoptera	Braconidae	Microgastrinae	Microplitis
DSWAS902-07	HYM00000931	HYM00000931	HYM00000931	657				HYM00000931	Arthropoda	Insecta	Hymenoptera	Braconidae	Microgastrinae	Pholetesor
DSWAS903-07	HYM00000932	HYM00000932	HYM00000932	657				HYM00000932	Arthropoda	Insecta	Hymenoptera	Braconidae	Microgastrinae	Pholetesor
DSWAS905-07	HYM00000934	HYM00000934	HYM00000934	657				HYM00000934	Arthropoda	Insecta	Hymenoptera	Braconidae	Microgastrinae	Pholetesor
DSWAS906-07	HYM00000935	HYM00000935	HYM00000935	657				HYM00000935	Arthropoda	Insecta	Hymenoptera	Braconidae	Microgastrinae	Pholetesor
DSWAS907-07	HYM00000936	HYM00000936	HYM00000936	657				HYM00000936	Arthropoda	Insecta	Hymenoptera	Braconidae	Microgastrinae	Pholetesor
DSWAS909-07	HYM00000938	HYM00000938	HYM00000938	657				HYM00000938	Arthropoda	Insecta	Hymenoptera	Braconidae	Microgastrinae	Pholetesor
DSWAS911-07	HYM00000940	HYM00000940	HYM00000940	657				HYM00000940	Arthropoda	Insecta	Hymenoptera	Braconidae	Microgastrinae	Pholetesor
DSWAS912-07	HYM00000941	HYM00000941	HYM00000941	657				HYM00000941	Arthropoda	Insecta	Hymenoptera	Braconidae	Microgastrinae	Pholetesor
DSWAS913-07	HYM00000942	HYM00000942	HYM00000942	657				HYM00000942	Arthropoda	Insecta	Hymenoptera	Braconidae	Microgastrinae	Pholetesor
DSWAS914-07	HYM00000943	HYM00000943	HYM00000943	657				HYM00000943	Arthropoda	Insecta	Hymenoptera	Braconidae	Microgastrinae	Pholetesor
DSWAS915-07	HYM00000944	HYM00000944	HYM00000944	657				HYM00000944	Arthropoda	Insecta	Hymenoptera	Braconidae	Microgastrinae	Pholetesor
DSWAS916-07	HYM00000945	HYM00000945	HYM00000945	657				HYM00000945	Arthropoda	Insecta	Hymenoptera	Braconidae	Microgastrinae	Pholetesor
DSWAS919-07	HYM00000948	HYM00000948	HYM00000948	657				HYM00000948	Arthropoda	Insecta	Hymenoptera	Braconidae	Microgastrinae	Pholetesor
DSWAS921-07	HYM00000950	HYM00000950	HYM00000950	657				HYM00000950	Arthropoda	Insecta	Hymenoptera	Braconidae	Microgastrinae	Pholetesor
DSWAS922-07	HYM00000951	HYM00000951	HYM00000951	657				HYM00000951	Arthropoda	Insecta	Hymenoptera	Braconidae	Microgastrinae	Pholetesor
DSWAS924-07	HYM00000953	HYM00000953	HYM00000953	601				HYM00000953	Arthropoda	Insecta	Hymenoptera	Braconidae	Microgastrinae	Pholetesor
DSWAS926-07	HYM00000955	HYM00000955	HYM00000955	657				HYM00000955	Arthropoda	Insecta	Hymenoptera	Braconidae	Microgastrinae	Pholetesor
DSWAS928-07	HYM00000957	HYM00000957	HYM00000957	654				HYM00000957	Arthropoda	Insecta	Hymenoptera	Braconidae	Microgastrinae	Pholetesor
DSWAS929-07	HYM00000958	HYM00000958	HYM00000958	657				HYM00000958	Arthropoda	Insecta	Hymenoptera	Braconidae	Microgastrinae	Pholetesor
DSWAS933-07	HYM00000962	HYM00000962	HYM00000962	657				HYM00000962	Arthropoda	Insecta	Hymenoptera	Braconidae	Microgastrinae	Pholetesor
DSWAS935-07	HYM00000964	HYM00000964	HYM00000964	657				HYM00000964	Arthropoda	Insecta	Hymenoptera	Braconidae	Microgastrinae	Pholetesor
DSWAS939-07	HYM00000968	HYM00000968	HYM00000968	657				HYM00000968	Arthropoda	Insecta	Hymenoptera	Braconidae	Microgastrinae	Pholetesor
DSWAS940-07	HYM00000969	HYM00000969	HYM00000969	614				HYM00000969	Arthropoda	Insecta	Hymenoptera	Braconidae	Microgastrinae	Pholetesor
DSWAS941-07	HYM00000970	HYM00000970	HYM00000970	657				HYM00000970	Arthropoda	Insecta	Hymenoptera	Braconidae	Microgastrinae	Pholetesor
DSWAS942-07	HYM00000971	HYM00000971	HYM00000971	657				HYM00000971	Arthropoda	Insecta	Hymenoptera	Braconidae	Microgastrinae	Pholetesor
DSWAS943-07	HYM00000972	HYM00000972	HYM00000972	614				HYM00000972	Arthropoda	Insecta	Hymenoptera	Braconidae	Microgastrinae	Pholetesor
DSWAS944-07	HYM00000973	HYM00000973	HYM00000973	657				HYM00000973	Arthropoda	Insecta	Hymenoptera	Braconidae	Microgastrinae	Pholetesor
DSWAS945-07	HYM00000974	HYM00000974	HYM00000974	657				HYM00000974	Arthropoda	Insecta	Hymenoptera	Braconidae	Microgastrinae	Pholetesor
DSWAS946-07	HYM00000975	HYM00000975	HYM00000975	657				HYM00000975	Arthropoda	Insecta	Hymenoptera	Braconidae	Microgastrinae	Pholetesor
DSWAS947-07	HYM00000976	HYM00000976	HYM00000976	657				HYM00000976	Arthropoda	Insecta	Hymenoptera	Braconidae	Microgastrinae	Pholetesor
DSWAS952-07	HYM00000981	HYM00000981	HYM00000981	657				HYM00000981	Arthropoda	Insecta	Hymenoptera	Braconidae	Microgastrinae	Pholetesor
DSWAS953-07	HYM00000982	HYM00000982	HYM00000982	657				HYM00000982	Arthropoda	Insecta	Hymenoptera	Braconidae	Microgastrinae	Pholetesor
DSWAS955-07	HYM00000984	HYM00000984	HYM00000984	631				HYM00000984	Arthropoda	Insecta	Hymenoptera	Braconidae	Microgastrinae	Pholetesor
DSWAS956-07	HYM00000985	HYM00000985	HYM00000985	657				HYM00000985	Arthropoda	Insecta	Hymenoptera	Braconidae	Microgastrinae	Pholetesor
DSWAS957-07	HYM00000986	HYM00000986	HYM00000986	657				HYM00000986	Arthropoda	Insecta	Hymenoptera	Braconidae	Microgastrinae	Pholetesor
DSWAS958-07	HYM00000987	HYM00000987	HYM00000987	657				HYM00000987	Arthropoda	Insecta	Hymenoptera	Braconidae	Microgastrinae	Pholetesor
DSWAS959-07	HYM00000988		HYM00000988	657				HYM00000988	Arthropoda	Insecta	Hymenoptera	Braconidae	Microgastrinae	Dolichogenidea
DSWAS960-07	HYM00000989	HYM00000989	HYM00000989	657				HYM00000989	Arthropoda	Insecta	Hymenoptera	Braconidae	Microgastrinae	Pholetesor
DSWAS961-07	HYM00000990	HYM00000990	HYM00000990	657				HYM00000990	Arthropoda	Insecta	Hymenoptera	Braconidae	Microgastrinae	Pholetesor
DSWAS963-07	HYM00000992	HYM00000992	HYM00000992	656				HYM00000992	Arthropoda	Insecta	Hymenoptera	Braconidae	Microgastrinae	Pholetesor
DSWAS964-07	HYM00000993	HYM00000993	HYM00000993	379				HYM00000993	Arthropoda	Insecta	Hymenoptera	Braconidae	Microgastrinae	Protapanteles
DSWAS965-07	HYM00000994	HYM00000994	HYM00000994	657				HYM00000994	Arthropoda	Insecta	Hymenoptera	Braconidae	Microgastrinae	Pholetesor
DSWAS967-07	HYM00000996	HYM00000996	HYM00000996	657				HYM00000996	Arthropoda	Insecta	Hymenoptera	Braconidae	Microgastrinae	Pholetesor
DSWAS988-07	HYM00000669	HYM00000669	HYM00000669	395	GU141144			HYM00000669	Arthropoda	Insecta	Hymenoptera	Braconidae	Microgastrinae	Dolichogenidea
TTHYB348-09	08BBHYM-0772	L#OEC-001		657				08BBHYM-0772	Arthropoda	Insecta	Hymenoptera	Braconidae	Microgastrinae	Cotesia
TTHYB415-09	08BBHYM-0839	L#08WATL-020		657				08BBHYM-0839	Arthropoda	Insecta	Hymenoptera	Braconidae	Microgastrinae	Cotesia
TTHYB423-09	08BBHYM-0847	L#08RIDMO-137		657				08BBHYM-0847	Arthropoda	Insecta	Hymenoptera	Braconidae	Microgastrinae	Cotesia
TTHYW1151-09	08BBHYM-1022	L#08WATL-007		657				08BBHYM-1022	Arthropoda	Insecta	Hymenoptera	Braconidae	Microgastrinae	Cotesia
TTHYW990-09	08BBHYM-0861	L#08GRAS-054	08BBHYM-0861	657				08BBHYM-0861	Arthropoda	Insecta	Hymenoptera	Braconidae	Microgastrinae	Microgaster
WSPAH043-06	CHU05-WSP-043	CHU05-WSP-043	CHU05-WSP-043	650	GU141195			CHU05-WSP-043	Arthropoda	Insecta	Hymenoptera	Braconidae	Microgastrinae	Glyptapanteles

ProcessID	Species	Identifier	Identifier email	Sex	Reproduction	Life Stage	Extra Info	Notes	Voucher Type	Tissue Type
DSWAS768-07	Microplitis jft04	Jose Leonardo Fernandez	jftriana@uoguelph.ca					<VERBATIM LOCALITY>|Burn Site|58.371|-93.4		
DSWAS769-07	Microplitis jft04	Jose Leonardo Fernandez	jftriana@uoguelph.ca					<VERBATIM LOCALITY>|Burn Site|58.371|-93.4		
DSWAS770-07	Microplitis jft04	Jose Leonardo Fernandez	jftriana@uoguelph.ca					<VERBATIM LOCALITY>|Burn Site|58.371|-93.4		
DSWAS771-07	Microplitis jft04	Jose Leonardo Fernandez	jftriana@uoguelph.ca					<VERBATIM LOCALITY>|Burn Site|58.371|-93.4		
DSWAS772-07	Microplitis jft04	Jose Leonardo Fernandez	jftriana@uoguelph.ca					<VERBATIM LOCALITY>|Burn Site|58.371|-93.4		
DSWAS773-07	Microplitis jft04	Jose Leonardo Fernandez	jftriana@uoguelph.ca					<VERBATIM LOCALITY>|Burn Site|58.371|-93.4		
DSWAS778-07	Microplitis varicolor	Jose Leonardo Fernandez	jftriana@uoguelph.ca					<VERBATIM LOCALITY>|Burn Site|58.371|-93.4		
DSWAS779-07	Microplitis varicolor	Jose Leonardo Fernandez	jftriana@uoguelph.ca					<VERBATIM LOCALITY>|Burn Site|58.371|-93.4		
DSWAS782-07	Microplitis varicolor	Jose Leonardo Fernandez	jftriana@uoguelph.ca					<VERBATIM LOCALITY>|Burn Site|58.371|-93.4		
DSWAS805-07	Glyptapanteles jft05	Jose Leonardo Fernandez	jftriana@uoguelph.ca					<VERBATIM LOCALITY>|Burn Site|58.371|-93.4		
DSWAS806-07	Glyptapanteles jft05	Jose Leonardo Fernandez	jftriana@uoguelph.ca					<VERBATIM LOCALITY>|Burn Site|58.371|-93.4		
DSWAS807-07	Glyptapanteles jft05	Jose Leonardo Fernandez	jftriana@uoguelph.ca					<VERBATIM LOCALITY>|Burn Site|58.371|-93.4		
DSWAS811-07	Glyptapanteles jft01	Jose Leonardo Fernandez	jftriana@uoguelph.ca					<VERBATIM LOCALITY>|Burn Site|58.371|-93.4		
DSWAS813-07	Glyptapanteles jft01	Jose Leonardo Fernandez	jftriana@uoguelph.ca					<VERBATIM LOCALITY>|Burn Site|58.371|-93.4		
DSWAS815-07	Glyptapanteles jft01	Jose Leonardo Fernandez	jftriana@uoguelph.ca					<VERBATIM LOCALITY>|Burn Site|58.371|-93.4		
DSWAS816-07	Glyptapanteles jft05	Jose Leonardo Fernandez	jftriana@uoguelph.ca					<VERBATIM LOCALITY>|Burn Site|58.371|-93.4		
DSWAS817-07	Glyptapanteles jft05	Jose Leonardo Fernandez	jftriana@uoguelph.ca					<VERBATIM LOCALITY>|Burn Site|58.371|-93.4		
DSWAS818-07	Glyptapanteles jft05	Jose Leonardo Fernandez	jftriana@uoguelph.ca					<VERBATIM LOCALITY>|Burn Site|58.371|-93.4		
DSWAS819-07	Glyptapanteles jft01	Jose Leonardo Fernandez	jftriana@uoguelph.ca					<VERBATIM LOCALITY>|Burn Site|58.371|-93.4		
DSWAS820-07	Glyptapanteles jft01	Jose Leonardo Fernandez	jftriana@uoguelph.ca					<VERBATIM LOCALITY>|Burn Site|58.371|-93.4		
DSWAS821-07	Glyptapanteles jft01	Jose Leonardo Fernandez	jftriana@uoguelph.ca					<VERBATIM LOCALITY>|Burn Site|58.371|-93.4		
DSWAS822-07	Glyptapanteles jft05	Jose Leonardo Fernandez	jftriana@uoguelph.ca					<VERBATIM LOCALITY>|Burn Site|58.371|-93.4		
DSWAS823-07	Glyptapanteles jft01	Jose Leonardo Fernandez	jftriana@uoguelph.ca					<VERBATIM LOCALITY>|Burn Site|58.371|-93.4		
DSWAS824-07	Glyptapanteles jft05	Jose Leonardo Fernandez	jftriana@uoguelph.ca					<VERBATIM LOCALITY>|Burn Site|58.371|-93.4		
DSWAS825-07	Glyptapanteles jft05	Jose Leonardo Fernandez	jftriana@uoguelph.ca					<VERBATIM LOCALITY>|Burn Site|58.371|-93.4		
DSWAS826-07	Glyptapanteles jft01	Jose Leonardo Fernandez	jftriana@uoguelph.ca					<VERBATIM LOCALITY>|Burn Site|58.371|-93.4		
DSWAS827-07	Glyptapanteles jft01	Jose Leonardo Fernandez	jftriana@uoguelph.ca					<VERBATIM LOCALITY>|Burn Site|58.371|-93.4		
DSWAS828-07	Glyptapanteles jft01	Jose Leonardo Fernandez	jftriana@uoguelph.ca					<VERBATIM LOCALITY>|Burn Site|58.371|-93.4		
DSWAS829-07	Glyptapanteles jft05	Jose Leonardo Fernandez	jftriana@uoguelph.ca					<VERBATIM LOCALITY>|Burn Site|58.371|-93.4		
DSWAS830-07	Glyptapanteles jft01	Jose Leonardo Fernandez	jftriana@uoguelph.ca					<VERBATIM LOCALITY>|Burn Site|58.371|-93.4		
DSWAS841-07	Illidops jft03	Jose Leonardo Fernandez	jftriana@uoguelph.ca					<VERBATIM LOCALITY>|Fort Churchill|||</VER	NULL	
DSWAS843-07	Dolichogenidea jft01	Jose Leonardo Fernandez	jftriana@uoguelph.ca					<VERBATIM LOCALITY>|Fort Churchill|||</VER		
DSWAS848-07	Dolichogenidea jft01	Jose Leonardo Fernandez	jftriana@uoguelph.ca					<VERBATIM LOCALITY>|Fort Churchill|||</VER		
DSWAS854-07	Illidops jft03	Jose Leonardo Fernandez	jftriana@uoguelph.ca					<VERBATIM LOCALITY>|Fort Churchill|||</VER	NULL	
DSWAS857-07	Microgaster jft10	Jose Leonardo Fernandez	jftriana@uoguelph.ca					<VERBATIM LOCALITY>|Burn Site|58.371|-93.4		
DSWAS858-07	Microgaster jft10	Jose Leonardo Fernandez	jftriana@uoguelph.ca					<VERBATIM LOCALITY>|Burn Site|58.371|-93.4		
DSWAS859-07	Microgaster jft01	Jose Leonardo Fernandez	jftriana@uoguelph.ca					<VERBATIM LOCALITY>|Warkworth Creek nr. (	NULL	
DSWAS863-07	Pholetesor bedelliae	Jose Leonardo Fernandez	jftriana@uoguelph.ca					<VERBATIM LOCALITY>|Burn Site|58.371|-93.4		
DSWAS865-07	Pholetesor viminetorum	Jose Leonardo Fernandez	jftriana@uoguelph.ca					<VERBATIM LOCALITY>|Fort Churchill|||</VER		
DSWAS866-07	Microplitis jft02	Jose Leonardo Fernandez	jftriana@uoguelph.ca					<VERBATIM LOCALITY>|Burn Site|58.371|-93.4		
DSWAS867-07	Microplitis jft03	Jose Leonardo Fernandez	jftriana@uoguelph.ca					<VERBATIM LOCALITY>|Fort Churchill|||</VER		
DSWAS868-07	Microplitis jft04	Jose Leonardo Fernandez	jftriana@uoguelph.ca					<VERBATIM LOCALITY>|Burn Site|58.371|-93.4		
DSWAS869-07	Microplitis jft04	Jose Leonardo Fernandez	jftriana@uoguelph.ca					<VERBATIM LOCALITY>|Burn Site|58.371|-93.4		
DSWAS902-07	Pholetesor bedelliae	Jose Leonardo Fernandez	jftriana@uoguelph.ca					HYM000489		
DSWAS903-07	Pholetesor viminetorum	Jose Leonardo Fernandez	jftriana@uoguelph.ca					HYM000490		
DSWAS905-07	Pholetesor bedelliae	Jose Leonardo Fernandez	jftriana@uoguelph.ca					HYM000492		
DSWAS906-07	Pholetesor bedelliae	Jose Leonardo Fernandez	jftriana@uoguelph.ca					HYM000493		
DSWAS907-07	Pholetesor bedelliae	Jose Leonardo Fernandez	jftriana@uoguelph.ca					HYM000494		
DSWAS909-07	Pholetesor bedelliae	Jose Leonardo Fernandez	jftriana@uoguelph.ca					HYM000496		
DSWAS911-07	Pholetesor bedelliae	Jose Leonardo Fernandez	jftriana@uoguelph.ca					HYM000498		
DSWAS912-07	Pholetesor bedelliae	Jose Leonardo Fernandez	jftriana@uoguelph.ca					HYM000499		
DSWAS913-07	Pholetesor bedelliae	Jose Leonardo Fernandez	jftriana@uoguelph.ca					HYM000500		
DSWAS914-07	Pholetesor bedelliae	Jose Leonardo Fernandez	jftriana@uoguelph.ca					HYM000501		
DSWAS915-07	Pholetesor viminetorum	Jose Leonardo Fernandez	jftriana@uoguelph.ca					HYM000502		
DSWAS916-07	Pholetesor viminetorum	Jose Leonardo Fernandez	jftriana@uoguelph.ca					HYM000503		
DSWAS919-07	Pholetesor bedelliae	Jose Leonardo Fernandez	jftriana@uoguelph.ca					HYM000506		
DSWAS921-07	Pholetesor bedelliae	Jose Leonardo Fernandez	jftriana@uoguelph.ca					HYM000508		
DSWAS922-07	Pholetesor bedelliae	Jose Leonardo Fernandez	jftriana@uoguelph.ca					HYM000509		
DSWAS924-07	Pholetesor bedelliae	Jose Leonardo Fernandez	jftriana@uoguelph.ca					HYM000511		
DSWAS926-07	Pholetesor bedelliae	Jose Leonardo Fernandez	jftriana@uoguelph.ca					HYM000513		
DSWAS928-07	Pholetesor salicifoliellae	Jose Leonardo Fernandez	jftriana@uoguelph.ca					HYM000515		
DSWAS929-07	Pholetesor bedelliae	Jose Leonardo Fernandez	jftriana@uoguelph.ca					HYM000516		
DSWAS933-07	Pholetesor viminetorum	Jose Leonardo Fernandez	jftriana@uoguelph.ca					HYM000520		
DSWAS935-07	Pholetesor viminetorum	Jose Leonardo Fernandez	jftriana@uoguelph.ca					HYM000522		
DSWAS939-07	Pholetesor bedelliae	Jose Leonardo Fernandez	jftriana@uoguelph.ca					HYM000526		
DSWAS940-07	Pholetesor bedelliae	Jose Leonardo Fernandez	jftriana@uoguelph.ca					HYM000527		
DSWAS941-07	Pholetesor bedelliae	Jose Leonardo Fernandez	jftriana@uoguelph.ca					HYM000528		
DSWAS942-07	Pholetesor bedelliae	Jose Leonardo Fernandez	jftriana@uoguelph.ca					HYM000529		
DSWAS943-07	Pholetesor bedelliae	Jose Leonardo Fernandez	jftriana@uoguelph.ca					HYM000530		
DSWAS944-07	Pholetesor bedelliae	Jose Leonardo Fernandez	jftriana@uoguelph.ca					HYM000531		
DSWAS945-07	Pholetesor bedelliae	Jose Leonardo Fernandez	jftriana@uoguelph.ca					HYM000532		
DSWAS946-07	Pholetesor bedelliae	Jose Leonardo Fernandez	jftriana@uoguelph.ca					HYM000533		
DSWAS947-07	Pholetesor bedelliae	Jose Leonardo Fernandez	jftriana@uoguelph.ca					HYM000534		
DSWAS952-07	Pholetesor viminetorum	Jose Leonardo Fernandez	jftriana@uoguelph.ca					HYM000539		
DSWAS953-07	Pholetesor viminetorum	Jose Leonardo Fernandez	jftriana@uoguelph.ca					HYM000540		
DSWAS955-07	Pholetesor ornigis	Jose Leonardo Fernandez	jftriana@uoguelph.ca					HYM000542		
DSWAS956-07	Pholetesor bedelliae	Jose Leonardo Fernandez	jftriana@uoguelph.ca					HYM000543		
DSWAS957-07	Pholetesor bedelliae	Jose Leonardo Fernandez	jftriana@uoguelph.ca					HYM000544		
DSWAS958-07	Pholetesor bedelliae	Jose Leonardo Fernandez	jftriana@uoguelph.ca					HYM000545		
DSWAS959-07	Dolichogenidea jft03	Jose Leonardo Fernandez	jftriana@uoguelph.ca					HYM000546		
DSWAS960-07	Pholetesor viminetorum	Jose Leonardo Fernandez	jftriana@uoguelph.ca					HYM000547		
DSWAS961-07	Pholetesor bedelliae	Jose Leonardo Fernandez	jftriana@uoguelph.ca					HYM000548		
DSWAS963-07	Pholetesor salicifoliellae	Jose Leonardo Fernandez	jftriana@uoguelph.ca					HYM000550		
DSWAS964-07	Protapanteles jft03	Jose Leonardo Fernandez	jftriana@uoguelph.ca					HYM000551		
DSWAS965-07	Pholetesor bedelliae	Jose Leonardo Fernandez	jftriana@uoguelph.ca					HYM000552		
DSWAS967-07	Pholetesor bedelliae	Jose Leonardo Fernandez	jftriana@uoguelph.ca					HYM000554		
DSWAS988-07	Dolichogenidea jft01	Jose Leonardo Fernandez	jftriana@uoguelph.ca					<VERBATIM LOCALITY>|Landing Lake|58.71|-9		
TTHYB348-09	Cotesia xylina	Jose Leonardo Fernandez	jftriana@uoguelph.ca		S	A		daytime netting and free hand	NULL	
TTHYB415-09	Cotesia xylina	Jose Leonardo Fernandez	jftriana@uoguelph.ca		S	A	Waterton	daytime netting	NULL	
TTHYB423-09	Cotesia jft06	Jose Leonardo Fernandez	jftriana@uoguelph.ca		S	A	Riding Mountain	Night UV sheet	NULL	
TTHYW1151-09	Cotesia jft28	Jose Leonardo Fernandez	jftriana@uoguelph.ca		S	A	Waterton	free-hand sampling	NULL	
TTHYW990-09	Microgaster canadensis	Jose Leonardo Fernandez	jftriana@uoguelph.ca		S	A	Grasslands	Daytime netting	NULL	
WSPAH043-06	Glyptapanteles jft03	Jose Leonardo Fernandez	jftriana@uoguelph.ca					<VERBATIM LOCALITY>|Landing lake|58.71|-9		

ProcessID	Collectors	Date Collected	Continent	Country/Ocean	Province	Region	Sector	Exact Site	Lat	Lon	Elev	GPS Source
DSWAS768-07		19-Aug-2006		Canada	Manitoba	Churchill	26 km SE Churchill, Tw		58.6170006	-93.811996	38	
DSWAS769-07		19-Aug-2006		Canada	Manitoba	Churchill	26 km SE Churchill, Tw		58.6170006	-93.811996	38	
DSWAS770-07		17-Aug-2006		Canada	Manitoba	Churchill	26 km SE Churchill, Tw		58.6170006	-93.811996	38	
DSWAS771-07		19-Aug-2006		Canada	Manitoba	Churchill	26 km SE Churchill, Tw		58.6170006	-93.811996	38	
DSWAS772-07		17-Aug-2006		Canada	Manitoba	Churchill	26 km SE Churchill, Tw		58.6170006	-93.811996	38	
DSWAS773-07		17-Aug-2006		Canada	Manitoba	Churchill	26 km SE Churchill, Tw		58.6170006	-93.811996	38	
DSWAS778-07		17-Aug-2006		Canada	Manitoba	Churchill	26 km SE Churchill, Tw		58.6170006	-93.811996	38	
DSWAS779-07		17-Aug-2006		Canada	Manitoba	Churchill	26 km SE Churchill, Tw		58.6170006	-93.811996	38	
DSWAS782-07		17-Aug-2006		Canada	Manitoba	Churchill	26 km SE Churchill, Tw		58.6170006	-93.811996	38	
DSWAS805-07		17-Aug-2006		Canada	Manitoba	Churchill	26 km SE Churchill, Tw		58.6170006	-93.811996	38	
DSWAS806-07		17-Aug-2006		Canada	Manitoba	Churchill	26 km SE Churchill, Tw		58.6170006	-93.811996	38	
DSWAS807-07		17-Aug-2006		Canada	Manitoba	Churchill	26 km SE Churchill, Tw		58.6170006	-93.811996	38	
DSWAS811-07		17-Aug-2006		Canada	Manitoba	Churchill	26 km SE Churchill, Tw		58.6170006	-93.811996	38	
DSWAS813-07		17-Aug-2006		Canada	Manitoba	Churchill	26 km SE Churchill, Tw		58.6170006	-93.811996	38	
DSWAS815-07		17-Aug-2006		Canada	Manitoba	Churchill	26 km SE Churchill, Tw		58.6170006	-93.811996	38	
DSWAS816-07		17-Aug-2006		Canada	Manitoba	Churchill	26 km SE Churchill, Tw		58.6170006	-93.811996	38	
DSWAS817-07		17-Aug-2006		Canada	Manitoba	Churchill	26 km SE Churchill, Tw		58.6170006	-93.811996	38	
DSWAS818-07		17-Aug-2006		Canada	Manitoba	Churchill	26 km SE Churchill, Tw		58.6170006	-93.811996	38	
DSWAS819-07		17-Aug-2006		Canada	Manitoba	Churchill	26 km SE Churchill, Tw		58.6170006	-93.811996	38	
DSWAS820-07		17-Aug-2006		Canada	Manitoba	Churchill	26 km SE Churchill, Tw		58.6170006	-93.811996	38	
DSWAS821-07		17-Aug-2006		Canada	Manitoba	Churchill	26 km SE Churchill, Tw		58.6170006	-93.811996	38	
DSWAS822-07		17-Aug-2006		Canada	Manitoba	Churchill	26 km SE Churchill, Tw		58.6170006	-93.811996	38	
DSWAS823-07		17-Aug-2006		Canada	Manitoba	Churchill	26 km SE Churchill, Tw		58.6170006	-93.811996	38	
DSWAS824-07		17-Aug-2006		Canada	Manitoba	Churchill	26 km SE Churchill, Tw		58.6170006	-93.811996	38	
DSWAS825-07		17-Aug-2006		Canada	Manitoba	Churchill	26 km SE Churchill, Tw		58.6170006	-93.811996	38	
DSWAS826-07		17-Aug-2006		Canada	Manitoba	Churchill	26 km SE Churchill, Tw		58.6170006	-93.811996	38	
DSWAS827-07		17-Aug-2006		Canada	Manitoba	Churchill	26 km SE Churchill, Tw		58.6170006	-93.811996	38	
DSWAS828-07		17-Aug-2006		Canada	Manitoba	Churchill	26 km SE Churchill, Tw		58.6170006	-93.811996	38	
DSWAS829-07		17-Aug-2006		Canada	Manitoba	Churchill	26 km SE Churchill, Tw		58.6170006	-93.811996	38	
DSWAS830-07		17-Aug-2006		Canada	Manitoba	Churchill	26 km SE Churchill, Tw		58.6170006	-93.811996	38	
DSWAS841-07	J.G.Chillcot	04-Jun-1952		Canada	Manitoba	Churchill	23 km E Churchill, Fort		58.7340012	-93.789001	27	
DSWAS843-07	J.G.Chillcot	08-Mar-1952		Canada	Manitoba	Churchill	23 km E Churchill, Fort		58.7340012	-93.789001	27	
DSWAS848-07	J.G.Chillcot	04-Jun-1952		Canada	Manitoba	Churchill	23 km E Churchill, Fort		58.7340012	-93.789001	27	
DSWAS854-07	J.G.Chillcot	03-Aug-1952		Canada	Manitoba	Churchill	23 km E Churchill, Fort		58.7340012	-93.789001	27	
DSWAS857-07		17-Aug-2006		Canada	Manitoba	Churchill	26 km SE Churchill, Tw		58.6170006	-93.811996	38	
DSWAS858-07		17-Aug-2006		Canada	Manitoba	Churchill	26 km SE Churchill, Tw		58.6170006	-93.811996	38	
DSWAS859-07	J.G.Chillcot	29-Jun-1952		Canada	Manitoba	Churchill	unspecified locality	Warkworth Creek nr. Cl	58.7000008	-94.099998		
DSWAS863-07		17-Aug-2006		Canada	Manitoba	Churchill	26 km SE Churchill, Tw		58.6170006	-93.811996	38	
DSWAS865-07	J.G.Chillcot	11-Jul-1952		Canada	Manitoba	Churchill	23 km E Churchill, Fort		58.7340012	-93.789001	27	
DSWAS866-07		17-Aug-2006		Canada	Manitoba	Churchill	26 km SE Churchill, Tw		58.6170006	-93.811996	38	
DSWAS867-07	J.G.Chillcot	07-Aug-1952		Canada	Manitoba	Churchill	23 km E Churchill, Fort		58.7340012	-93.789001	27	
DSWAS868-07		17-Aug-2006		Canada	Manitoba	Churchill	26 km SE Churchill, Tw		58.6170006	-93.811996	38	
DSWAS869-07		17-Aug-2006		Canada	Manitoba	Churchill	26 km SE Churchill, Tw		58.6170006	-93.811996	38	
DSWAS902-07	Goulet & Boudreault	15-Jul-2006		Canada	Yukon Territory			Pelly Crossing	62.8310013	-136.58501	495	
DSWAS903-07	Goulet & Boudreault	15-Jul-2006		Canada	Yukon Territory			Pelly Crossing	62.8310013	-136.58501	495	
DSWAS905-07	Goulet & Boudreault	15-Jul-2006		Canada	Yukon Territory			Pelly Crossing	62.8310013	-136.58501	495	
DSWAS906-07	Goulet & Boudreault	15-Jul-2006		Canada	Yukon Territory			Pelly Crossing	62.8310013	-136.58501	495	
DSWAS907-07	Goulet & Boudreault	15-Jul-2006		Canada	Yukon Territory			Pelly Crossing	62.8310013	-136.58501	495	
DSWAS909-07	Goulet & Boudreault	08-Jul-2006		Canada	Yukon Territory			Champagne	60.7900009	-136.437	733	
DSWAS911-07	Goulet & Boudreault	08-Jul-2006		Canada	Yukon Territory			Champagne	60.7900009	-136.437	733	
DSWAS912-07	Goulet & Boudreault	08-Jul-2006		Canada	Yukon Territory			Champagne	60.7900009	-136.437	733	
DSWAS913-07	Goulet & Boudreault	08-Jul-2006		Canada	Yukon Territory			Champagne	60.7900009	-136.437	733	
DSWAS914-07	Goulet & Boudreault	08-Jul-2006		Canada	Yukon Territory			Champagne	60.7900009	-136.437	733	
DSWAS915-07	Goulet & Boudreault	15-Jul-2006		Canada	Yukon Territory			Pelly Crossing	62.8310013	-136.58501	495	
DSWAS916-07	Goulet & Boudreault	15-Jul-2006		Canada	Yukon Territory			Pelly Crossing	62.8310013	-136.58501	495	
DSWAS919-07	Goulet & Boudreault	15-Jul-2006		Canada	Yukon Territory			Pelly Crossing	62.8310013	-136.58501	495	
DSWAS921-07	Goulet & Boudreault	15-Jul-2006		Canada	Yukon Territory			Pelly Crossing	62.8310013	-136.58501	495	
DSWAS922-07	Goulet & Boudreault	15-Jul-2006		Canada	Yukon Territory			Pelly Crossing	62.8310013	-136.58501	495	
DSWAS924-07	Goulet & Boudreault	15-Jul-2006		Canada	Yukon Territory			Pelly Crossing	62.8310013	-136.58501	495	
DSWAS926-07	Goulet & Boudreault	15-Jul-2006		Canada	Yukon Territory			Pelly Crossing	62.8310013	-136.58501	495	
DSWAS928-07	Goulet & Boudreault	08-Jul-2006		Canada	Yukon Territory			Champagne	60.7900009	-136.437	733	
DSWAS929-07	Goulet & Boudreault	05-Jul-2006		Canada	Yukon Territory			Fish Lake road	60.6879997	-135.23801	1107	
DSWAS933-07	Goulet & Boudreault	06-Jul-2006		Canada	Yukon Territory			Tahkini River road	60.875	-135.386	693	
DSWAS935-07	Goulet & Boudreault	06-Jul-2006		Canada	Yukon Territory			Tahkini River road	60.875	-135.386	693	
DSWAS939-07	Goulet & Boudreault	08-Jul-2006		Canada	Yukon Territory			Champagne	60.7900009	-136.437	733	
DSWAS940-07	Goulet & Boudreault	08-Jul-2006		Canada	Yukon Territory			Champagne	60.7900009	-136.437	733	
DSWAS941-07	Goulet & Boudreault	08-Jul-2006		Canada	Yukon Territory			Champagne	60.7900009	-136.437	733	
DSWAS942-07	Goulet & Boudreault	08-Jul-2006		Canada	Yukon Territory			Champagne	60.7900009	-136.437	733	
DSWAS943-07	Goulet & Boudreault	08-Jul-2006		Canada	Yukon Territory			Champagne	60.7900009	-136.437	733	
DSWAS944-07	Goulet & Boudreault	08-Jul-2006		Canada	Yukon Territory			Champagne	60.7900009	-136.437	733	
DSWAS945-07	Goulet & Boudreault	08-Jul-2006		Canada	Yukon Territory			Champagne	60.7900009	-136.437	733	
DSWAS946-07	Goulet & Boudreault	08-Jul-2006		Canada	Yukon Territory			Champagne	60.7900009	-136.437	733	
DSWAS947-07	Goulet & Boudreault	08-Jul-2006		Canada	Yukon Territory			Champagne	60.7900009	-136.437	733	
DSWAS952-07	Goulet & Boudreault	06-Jul-2006		Canada	Yukon Territory			Tahkini River road	60.875	-135.386	693	
DSWAS953-07	Goulet & Boudreault	06-Jul-2006		Canada	Yukon Territory			Tahkini River road	60.875	-135.386	693	
DSWAS955-07	Goulet & Boudreault	06-Jul-2006		Canada	Yukon Territory			Tahkini River road	60.875	-135.386	693	
DSWAS956-07	Goulet & Boudreault	08-Jul-2006		Canada	Yukon Territory			Champagne	60.7900009	-136.437	733	
DSWAS957-07	Goulet & Boudreault	08-Jul-2006		Canada	Yukon Territory			Champagne	60.7900009	-136.437	733	
DSWAS958-07	Goulet & Boudreault	08-Jul-2006		Canada	Yukon Territory			Champagne	60.7900009	-136.437	733	
DSWAS959-07	Goulet & Boudreault	15-Jul-2006		Canada	Yukon Territory			Pelly Crossing	62.8310013	-136.58501	495	
DSWAS960-07	Goulet & Boudreault	05-Jul-2006		Canada	Yukon Territory			Fish Lake road	60.6879997	-135.23801	1107	
DSWAS961-07	Goulet & Boudreault	15-Jul-2006		Canada	Yukon Territory			Pelly Crossing	62.8310013	-136.58501	495	
DSWAS963-07	Goulet & Boudreault	06-Jul-2006		Canada	Yukon Territory			Tahkini River road	60.875	-135.386	693	
DSWAS964-07	Goulet & Boudreault	06-Jul-2006		Canada	Yukon Territory			Tahkini River road	60.875	-135.386	693	
DSWAS965-07	Goulet & Boudreault	16-Jul-2006		Canada	Yukon Territory			Pelly Crossing	62.8310013	-136.58501	495	
DSWAS967-07	Goulet & Boudreault	16-Jul-2006		Canada	Yukon Territory			Pelly Crossing	62.8310013	-136.58501	495	
DSWAS988-07		29-Jul-2005		Canada	Manitoba	Churchill	10 km SE Churchill, Fa		58.7099991	-94.050003		
TTHYB348-09	J.Cossey, J.Sones,	25-May-2008		Canada	Ontario	Kawartha	Oliver Ecological Centr	fallow field	44.5699997	-78.5	250	
TTHYB415-09	J.Straka	23-Jul-2008		Canada	Alberta	Waterton	Waterton Lakes Nat. Pa	Subalpine forest/meadc	49.0559998	-114.042	1912	
TTHYB423-09	J.Straka,J.Cossey	13-Aug-2008		Canada	Manitoba	Riding Mountain	Riding Mountain Nat. P	Oak/mixed deciduous fc	50.7820015	-99.639	400	
TTHYW1151-09	J.Straka	22-Jul-2008		Canada	Alberta	Waterton	Waterton Lakes Nat. Pa	Rocky creekside/meadc	49.0779991	-113.968	1540	
TTHYW990-09	J.Cossey, N.Jeffery	19-Jul-2008		Canada	Saskatchewan	Grasslands	Belza`s Ranch Grasslai	Sage/willow grass valley	49.1529999	-107.517	773	
WSPAH043-06	C.Sheffield	29-Jul-2005		Canada	Manitoba	Churchill	10 km SE Churchill, Fa	Landing lake	58.7099991	-94.050003		
